# Supplementary material for: Visible-light photoredox-catalyzed C–O bond cleavage of diaryl ethers by acridinium photocatalysts at room temperature
Source: Nat Commun. 2020 Nov 30;11:6126. doi: 10.1038/s41467-020-19944-x (PMC7705023; doi:10.1038/s41467-020-19944-x)
Supplement: Supplementary file 1 — Supplementary Information [file 41467_2020_19944_MOESM1_ESM.pdf]

Supplementary Information

Visible-Light Photoredox-Catalyzed C–O Bond Cleavage of Diaryl Ethers by Acridinium Photocatalysts at Room Temperature

Fang-Fang Tan,[1] Xiao-Ya He,[1] Wan-Fa Tian[1] and Yang Li[1,2] ✉

[1]Frontier Institute of Science and Technology and State Key Laboratory of Multiphase Flow in Power Engineering, Xi'an Jiaotong University, Xi'an, Shaanxi, 710054, P. R. China
[2]State Key Laboratory of Elemento-Organic Chemistry, Nankai University, Tianjin 300071, P. R. China
✉email: liyang79@mail.xjtu.edu.cn

Table of Contents

General Information 3
Synthetic Procedures 3
Preparation of Acridinium Photocatalysts 3
Preparation of Diaryl Ethers 7
Supplementary Table 1 Lewis Acid and Solvent Screening 11
General Procedure for the C-O Bond Cleavage of Diaryl Ethers 11
C-O bond Cleavage of an Aryl Ether by a One-pot, Two-step Reaction 21
The Application of C-O Bond Cleavage to the Model of 4-O-5 Lignin Linkages 21
X-ray Crystallography Studies of PC 9 22
Mechanistic Investigation 23
Quantum Yield Measurements: 23
UV-Vis Absorption of 1a, 2a, PC 9 and the Reaction Mixture 24
Emission Quenching Experiments: 24
Deducing the Possible Intermediate B 28
Redox Properties and the Fluorescence Lifetime of Acridinium Salts 31
Supplementary Table 3 Redox Potentials 39
^{1}H NMR and ^{13}C NMR Spectra 40
Supplementary References 91

General Information

Acetonitrile (CH_3CN) was freshly distilled over CaH_2. If no special indicated, other reagents and solvents were used as commercially available without further purification. Flash column chromatography was accomplished using 200-300 mesh silica gel. NMR spectra were measured on a Bruker Avance-400 spectrometer in the solvents indicated; chemical shifts are reported in units (ppm) by assigning TMS resonance in the ^1H spectrum as 0.00 ppm, $CDCl_3$ resonance in the ^{13}C spectrum as 77.00 ppm. Coupling constants are reported in Hz with multiplicities denoted as br (broad), s (singlet), d (doublet), t (triplet), q (quartet) and m (multiple). Mass spectra (GC-MS) were detected at Thermo Trace 1300 gas chromatograph mass spectrometer and a TR-5MS column (0.25 mm × 30 m, Film: 0.25 μm). Infrared spectra were collected on a Thermo Fisher Nicolet 6700 FT-IR spectrometer using ATR (Attenuated Total Reflectance) method. Absorption maxima (v max) are reported in wavenumbers (cm^{-1}). HRMS were performed a Bruker Daltonics APEX II 47e FT-ICR mass spectrometer or on a Fisons VG Autospec double focusing sector-field instrument by using electrospray ionization (ESI) techniques. UV-vis absorption spectrum, the quantum yield measurement and steady-state emission spectra were recorded using a PerkinElmer Lambda or a HITACHI F-4500 Fluorescence Spectrometer. The lifetime of fluorescence of photoredox catalysts were determined by Steady-State Transient Fluorescence Spectrometer (Edinburgh FLS9). Cyclic voltammetry experiments were determined on a CHI660E Electrochemical Workstation equipped with the conventional three electrode system under argon atmosphere. The measurements were performed in CH_3CN containing 0.1 M NBu_4PF_6 using ferrocene/ferrocenium (Fc^+/Fc^0) as an internal reference. The working electrode was a gold plate electrode (d = 0.3 cm). The auxiliary and reference electrode consisted of a Pt tablets (or reticulated platinum) and an $Ag/AgNO_3$ (0.1 M in CH_3CN), respectively. All the photoredox catalyzed reactions were carried out in reaction tubes with Wattecs Blue LEDs Irradiation Parallel Reactor (WP-MSAR-620A) or polytetrafluoroethylene (PTFE) tubing under argon atmosphere by a flow reactor, the inner reaction temperature was determined as 19-23 °C.

Synthetic Procedures

Preparation of Acridinium Photocatalysts

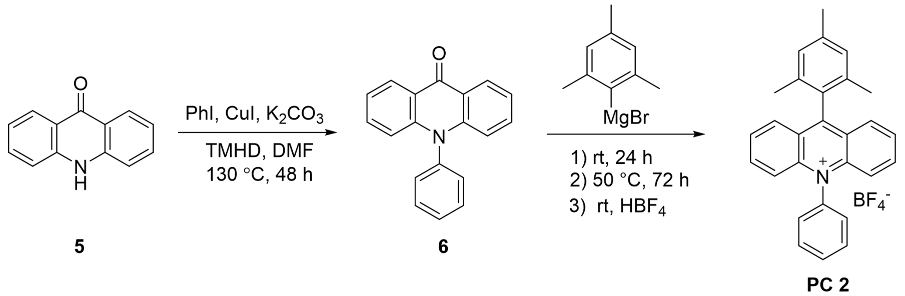

According to the literature,[1] to a 50 mL flame dried three-necked round bottom flask with a stirring bar, acridin-9(10H)-one (**5**, 0.50 g, 2.56 mmol, 1 equiv), CuI (49 mg, 0.256 mmol, 0.1 equiv) and K_2CO_3 (0.71 g, 5.12 mmol, 2.0 equiv) were added. The flask was evacuated and filled with argon three times. Iodobenzene (0.314 mL, 2.82 mmol, 1.1 equiv), 2,2,6,6-tetramethylheptane-3,5-dione (TMHD) (0.107 mL, 0.512 mmol, 0.2 equiv) and dry DMF (12 mL) were added under argon atmosphere. The solution was heated to 130 °C for 48 h. After cooling to room temperature, the reaction was quenched with 3 M HCl (10 mL). The aqueous solution was extracted with dichloromethane (DCM) (3 x 50 mL). The combined organic layers were washed with sodium bicarbonate (50 mL), ammonium chloride (50 mL), brine, dried over Na_2SO_4, concentrated in vacuo and purified on silica-gel column chromatography (petroleum ether/EtOAc = 5/1) to give **6** as a yellow solid (0.417 g, 60% yield). Compound **6** was accumulated by several batches.

According to the literature,[1] to a 50 mL flame dried three-necked round bottom flask with a stirring bar, compound **6** (0.53 g, 1.96 mmol, 1.0 equiv) was added. The flask was evacuated and filled with argon three times. Then dry THF (21 mL) was added under argon atmosphere. Freshly prepared mesityl magnesium bromide in THF (~ 2.5 equiv)[1] was added dropwise and the solution was stirred at room temperature for 24 h. The solution was then heated to 50 °C for 72 h. After cooling to room temperature, the reaction mixture was quenched with sodium bicarbonate (15 mL). The aqueous layer was extracted with DCM (3 x 20 mL) and followed by a brine wash,

dried over Na_2SO_4 and concentrated in vacuo to afford a red oil. The oil was then dissolved in ether (8 mL) and tetrafluoroboric acid diethyl ether complex (0.32 mL, 1.2 equiv) in ether was added dropwise and the solution was stirred for 1 h until the precipitate quickly appeared. The yellow precipitate was collected by filtration and washed with ether (10 mL) to afford **PC 2** (0.578 g, 64% yield). ^{1}H NMR (400 MHz, $CDCl_3$) δ 8.26-8.07 (m, 2H), 7.97-7.84 (m, 5H), 7.84-7.76 (m, 2H), 7.73 (d, *J* = 6.8 Hz, 2H), 7.61 (d, *J* = 9.2 Hz, 2H), 7.18 (s, 2H), 2.50 (s, 3H), 1.84 (s, 6H). ^{13}C NMR (100 MHz, $CDCl_3$) δ 164.6, 141.8 (2C), 140.4, 139.0 (2C), 136.7, 136.0 (2C), 131.9, 131.6 (2C), 129.1, 129.0 (2C), 128.7 (2C), 128.6 (2C), 127.9 (2C), 125.8 (2C), 120.1 (2C), 21.2, 20.1 (2C). HRMS: Calculated for $(M\text{-}BF_4)^+$: 374.1903; found: 374.1908.

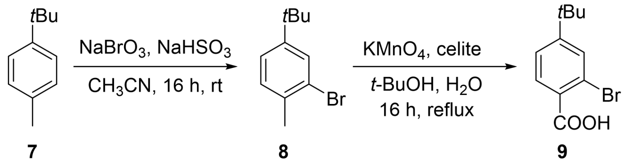

According to the literature,[2] to a 500 mL flame dried three-necked round bottom flask with a stirring bar, compound **7** (5.0 mL, 29.0 mmol), $NaBrO_3$ (13.2 g, 87.0 mmol), CH_3CN (60 mL) and H_2O (45 mL) were added. $NaHSO_3$ (9.0 g, 87.0 mmol) was dissolved in H_2O (90 mL), then was added dropwise into the solution in 30 minutes. The reaction mixture was stirred for 16 h at room temperature. After the reaction, the solution was extracted with Et_2O (3 x 100 mL). The combined organic layers were washed with 10% aq $Na_2S_2O_3$ (2 x 100 mL) and dried over Na_2SO_4. The solvents were removed in vacuo to give compound **8** as a yellow oil (6.46 g, 98% crude yield), which was directed used for the next step. Compound **8** (6.46 g, 28.4 mmol) was dissolved in *t*-BuOH (50 mL) and H_2O (50 mL). $KMnO_4$ (9.2 g, 58.0 mmol) and celite (10.0 g) were added and the reaction mixture was refluxed for 16 h. The reaction mixture was cooled to room temperature and filtered through a pad of celite. The filtrate was acidified with HCl and extracted with methyl tert-butyl ether (3 x 75 mL). The combined organic layers were washed with 5% aq NaOH (3 x 50 mL), acidified with HCl, extracted with methyl tert-butyl ether (3 x 75 mL), and dried over Na_2SO_4. The solvents were removed in vacuo to give compound **9** as a white solid (5.25 g, 72% crude yield), which was used directly for the next step. Compound **9** was accumulated by several batches.

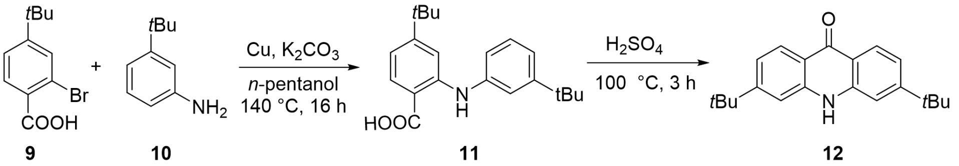

According to the literature,[1] to a 250 mL flame dried three-neck round bottom flask with a stirring bar, K_2CO_3 (6.0 g, 43.5 mmol, 1.4 equiv), Cu (0.37 g, 5.36 mmol, 0.2 equiv) and compound **9** (8.0 g, 31.3 mmol, 1.0 equiv) were added. The flask was evacuated and filled with argon three times. Compound **10** (6.90 mL, 43.5 mmol, 1.4 equiv) and *n*-pentanol (48 mL) were both mixed and bubbled with argon for 0.25 h, then were added to the reaction flask under argon atmosphere. The mixture was heated to 140 °C for 16 h. After cooling to room temperature, it was diluted with water (100 mL), washed with 3 M HCl (100 mL) and extracted with DCM (3 x 100 mL). The organic layer was washed with saturated aqueous solution of NH_4Cl (100 mL), brine (10 mL), dried over Na_2SO_4, and concentrated in vacuo to give a brown solid. The crude product was recrystallized in methanol to afford compound **11** (6.15 g, 60% yield).

According to the literature,[1] to a 100 mL flame dried three-necked round bottom flask with a stirring bar, concentrated H_2SO_4 (46 mL) was added and heated to 100 °C. Compound **11** (6.15 g, 18.9 mmol) was added to the sulfuric acid in portions and stirred for 3 h at 100 °C. The solution was cooled to room temperature and the acidic solution was then slowly poured into water at 0 °C to form a yellow precipitate. Ammonium hydroxide was added to the mixture to neutralize the added H_2SO_4. The resulted yellow precipitate was then filtered to afford the desired product along with the undesired regio-isomer. The mixture was dissolved in hot MeOH. After cooling to room temperature, the undissolved solid was removed by filtration. The filtrate was concentrated in vacuo to give a yellow solid, which was recrystallized in DCM and MeOH to afford the desired compound **12** (3.75 g, 65% yield).

According to the literature,[1] compound **12** (3.75 g, 12.2 mmol, 1 equiv), CuI (232 mg, 1.22 mmol, 0.1 equiv) and K_2CO_3 (3.37 g, 24.4 mmol, 2.0 equiv) were added to a dried three-necked round bottom flask. The flask was evacuated and filled with argon three times. DMF (62 mL) was added under argon atmosphere, followed by iodobenzene (13.3 mmol, 1.1 equiv) or 1-(tert-butyl)-4-iodobenzene (13.3 mmol, 1.1 equiv), and 2,2,6,6-tetramethylheptane-3,5-dione (TMHD) (0.51 mL, 2.44 mmol, 0.2 equiv). The solution was heated at 130 °C for 48 h. The solution was then cooled to room temperature and quenched with 3 M HCl (50

mL), extracted with DCM (3 x 100 mL). The combined organic layers were washed with sodium bicarbonate (150 mL), ammonium chloride (150 mL), brine, dried over Na_2SO_4 and concentrated in vacuo. The final pale yellow solid compound **13a** (2.6 g, 56% yield) and **13b** (3.0 g, 56% yield) were obtained after flash chromatography (petroleum ether/EtOAc = 5/1), respectively.

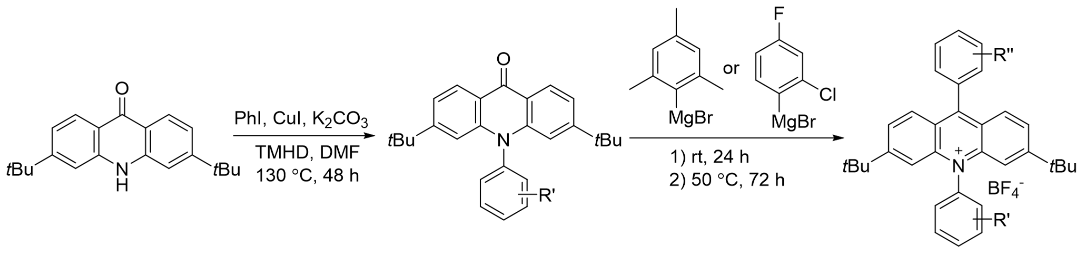

12

13a R'=H
13b R'=4-*t*Bu

PC 3, R'=H, R''=2,4,6-tri-CH_3
PC 10, R'=4-*t*Bu, R''=2-Cl-4-F
PC 12, R'=H, R''=2-Cl-4-F

According to the literature,[1] compound **13** (0.56 mmol, 1.0 equiv) was added to a three-necked round bottom flask. The flask was evacuated and filled with argon three times. Dry THF (15 mL, 0.035 M) was added under argon atmosphere. Freshly prepared (2-chloro-4-fluorophenyl)magnesium bromide (~2.5 equiv) or mesityl magnesium bromide (~2.5 equiv) was added dropwise and the solution was stirred at room temperature for 24 h. Then the solution was then heated to 50 °C for 72 h. The red solution was cooled and quenched with sodium bicarbonate (0.04 M). The aqueous layer was extracted with DCM followed by a brine wash, dried over Na_2SO_4 and concentration in vacuo to afford a red oil. The oil dissolved in diethyl ether. Tetrafluoroboric acid diethyl ether complex (1.2 equiv) in ether was added dropwise and the solution was stirred for 1 h until a precipitate quickly appeared. The yellow precipitate was collected by filtration and washed with ether to afford **PC 3** (275 mg, 86% yield), **PC 10** (290 mg, 81 % yield), **PC 12** (280 mg, 86% yield), respectively. **PC 3**: ^1H NMR (400 MHz, $CDCl_3$) δ 8.02-7.94 (m, 2H), 7.94-7.87 (m, 1H), 7.79 (s, 4H), 7.764-7.68 (m, 2H), 7.41 (s, 2H), 7.17 (s, 2H), 2.49 (s, 3H), 1.86 (s, 6H), 1.29 (s, 18H). ^{13}C NMR (100 MHz, $CDCl_3$) δ 163.6, 162.3, 142.0 (2C), 140.2, 136.7, 136.0 (2C), 131.8 (2C), 131.61 (2C), 129.2, 128.9 (2C), 128.3 (2C), 127.9 (2C), 127.5 (2C), 124.0 (2C), 115.0 (2C), 36.6 (2C), 30.2 (6C), 21.2, 20.2 (2C). HRMS: Calculated for $(M-BF_4)^+$: 486.3155; found: 486.3168. **PC 10**: ^1H NMR (400 MHz, $CDCl_3$) δ 7.97-7.91 (m, 1H), 7.91-7.83 (m, 3H), 7.83-7.71 (m, 4H), 7.50-7.43 (m, 2H), 7.43-7.32 (m, 3H), 1.51 (s, 9H), 1.28 (s, 18H). ^{13}C NMR (100 MHz, $CDCl_3$) δ 163.6 (d, J = 252.9 Hz), 162.9, 156.6, 155.3, 142.5 (2C), 134.5, 134.1 (d, J = 9.3 Hz), 133.6 (d, J = 10.0 Hz), 128.8 (2C), 128.4 (2C), 127.4 (2C), 127.2 (2C), 126.5 (2C), 124.4 (2C), 117.4 (d, J = 24.8 Hz), 115.7 (d, J = 21.3 Hz), 114.8 (2C), 36.5 (2C), 35.3, 31.3 (3C), 30.1 (6C). IR (cm^{-1}): ν 3070.14, 2962.59, 2870.79, 1610.11, 1579.77, 1503.54, 1416.94, 1399.64, 1267.91, 1226.38, 1197.52, 1055.13, 924.30, 873.14, 836.73, 733.57, 684.73. HRMS: Calculated for $(M-BF_4)^+$: 552.2828; found: 552.2827. **PC 12**: ^1H NMR (400 MHz, $CDCl_3$) δ 8.05-7.91 (m, 2H), 7.92-7.84 (m, 3H), 7.84-7.72 (m, 4H), 7.61-7.51 (m, 1H), 7.47 (dd, J = 8.0, 2.4 Hz, 1H), 7.43-7.30 (m, 3H), 1.28 (s, 18H). ^{13}C NMR (100 MHz, $CDCl_3$) δ 163.6 (d, J = 253.1 Hz), 163.2, 156.8, 142.3 (2C), 137.0 (2C), 134.0 (d, J = 9.0 Hz), 133.7 (d, J = 10.2 Hz), 131.9, 131.7, 130.9 (2C), 128.8, 128.5 (2C), 128.2 (d, J = 3.8 Hz), 127.3 (2C), 127.1, 124.3, 117.4 (d, J = 24.8 Hz), 115.6 (d, J =21.1 Hz), 114.7 (2C), 36.6 (2C), 30.1 (6C). IR (cm^{-1}): ν 3223.08, 3069.08, 2964.37, 2872.09, 1611.79, 1578.39, 1455.06, 1417.19, 1385.91, 1254.69, 1226.60, 1050.76, 923.36, 872.79, 837.64, 779.13, 732.82, 702.88. HRMS: Calculated for $(M-BF_4)^+$: 496.2202; found: 496.2200.

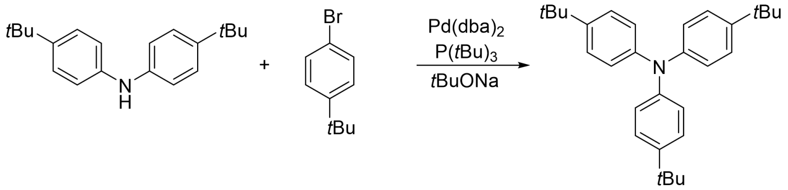

14 **15** **16**

According to the literature,[3] compound **14** (5.00 g, 17.76 mmol), $Pd(dba)_2$ (204.24 mg, 0.02 mmol, 2.0 mol%), and *t*-BuONa (2.56 g, 26.6 mmol, 1.5 equiv) were added to a dried three-necked round bottom flask. The flask was evacuated and filled with argon for three times. Dry toluene (34 mL) was added under argon atmosphere, followed by 1-bromo-4-(tert-butyl)benzene **15** (3.82 g, 17.9 mmol, 1.01 equiv), $P(tBu)_3$ (66.8 uL, 0.28 mmol, 1.6 mol%). The flask was stirred for 24 h at room temperature. After the reaction, Et_2O (75 mL) was added and the organic layer was washed with H_2O (50 mL). The aqueous layer was extracted with Et_2O (3 x 100 mL). The combined organic layers were washed with brine, dried over Na_2SO_4, concentrated in vacuo and the residue was purified via silica-gel column chromatography to give **16** as a white solid (6.0 g, 82% yield).

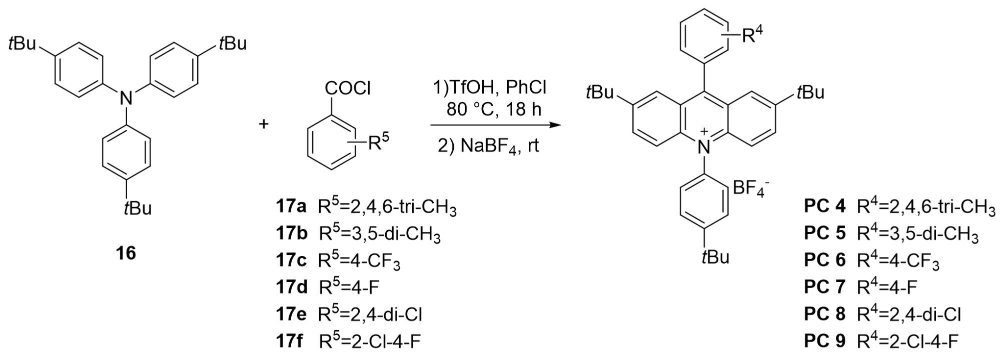

According to the literature,[4] compound **16** (2.00 g, 4.8 mmol) was added to a dried reaction tube. The flask was evacuated and filled with argon three times. Chlorobenzene (16 mL) and compound **17a** (9.6 mmol, 1.69 mL) was added under argon atmosphere, followed by triflic acid (0.43 mL, 4.8 mmol). The flask was sealed and stirred for 18 h at 80 °C. After cooling to room temperature, $NaBF_4$ (0.2 M, 3 × 10 mL) was added to the reaction mixture and stirred for a while, then washed with H_2O (2 × 30 mL). Methyl *tert*-butyl ether (120 mL) was added slowly until a precipitate started to form and another batch of methyl *tert*-butyl ether (150 mL) was added slowly again. This mixture was stirred for 0.5 h. The precipitate was collected by filtration, washed with MTBE and dried in vacuum for 0.5 h, **PC 4** (2.10 g) was obtained as a bright orange solid in 69% yield. **PC 5-9** were prepared according to the same procedure**. PC 4:** ^{1}H NMR (400 MHz, $CDCl_3$) δ 8.28-8.13 (m, 2H), 7.90 (d, *J* = 8.0 Hz, 2H), 7.71 (s, 2H), 7.66-7.49 (m, 4H), 7.20 (s, 2H), 2.52 (s, 3H), 1.84 (s, 6H), 1.51 (s, 9H), 1.31 (s, 18H). ^{13}C NMR (100 MHz, $CDCl_3$) δ 162.5, 155.5, 152.0 (2C), 140.2, 140.1 (2C), 137.8 (2C), 135.7 (2C), 134.0, 129.1, 129.0 (2C), 128.5 (2C), 127.3 (2C), 125.8 (2C), 122.6 (2C), 119.9 (2C), 35.3, 35.2 (2C), 31.3 (3C), 30.5 (6C), 21.3, 20.2 (2C). IR (cm^{-1}): ν 2964.10, 2871.42, 1610.75, 1542.94, 1495.14, 1366.66, 1264.05, 1222.74, 1150.08, 1057.65, 1030.93, 988.34, 828.83, 732.97. HRMS: Calculated for $(M\text{-}BF_4)^+$: 542.3781; found: 542.3783. **PC 5** (1.80 g, 61% yield): ^{1}H NMR (400 MHz, $CDCl_3$) δ 8.16 (dd, *J* = 9.6, 2.0 Hz, 2H), 7.96 (d, *J* =1.2 Hz, 2H), 7.88 (d, *J* = 8.4 Hz, 2H), 7.57 (d, *J* = 6.8 Hz, 2H), 7.52 (d, *J* = 9.6 Hz, 2H), 7.37 (s, 1H), 7.23 (s, 2H), 2.52 (s, 6H), 1.50 (s, 9H), 1.32 (s, 18H). ^{13}C NMR (100 MHz, $CDCl_3$) δ 161.9, 155.4, 150.9 (2C), 140.3 (2C), 138.6 (2C), 137.5 (2C), 134.2, 132.9, 132.1, 128.4 (2C), 127.7 (2C), 127.3 (2C), 125.8 (2C), 124.1 (2C), 119.4 (2C), 35.3, 31.3 (2C), 30.4 (9C), 21.3 (2C). IR (cm^{-1}): ν 2963.27, 2870.26, 1586.77, 1546.55, 1508.33, 1392.97, 1365.72, 1306.80, 1263.98, 1222.66, 1149.76, 1030.95, 992.21, 922.82, 845.24, 826.28, 731.49. HRMS: Calculated for $(M\text{-}BF_4)^+$: 528.3630; found: 528.3632. **PC 6** (2.04 g, 65% yield): ^{1}H NMR (400 MHz, $CDCl_3$) δ 8.14 (dd, *J* = 9.6, 2.0 Hz, 2H), 8.03 (d, *J* = 8.0 Hz, 2H), 7.93-7.79 (m, 4H), 7.74 (d, *J* = 2.0 Hz, 2H), 7.66 (d, *J* = 8.4 Hz, 2H), 7.54 (d, *J* = 9.6 Hz, 2H), 1.50 (s, 9H), 1.30 (s, 18H). ^{13}C NMR (100 MHz, $CDCl_3$) δ 159.0, 155.3, 151.2 (2C), 140.5 (2C), 137.4 (2C), 137.2, 134.4 (2C), 132.2 (q, *J* = 32.8 Hz), 130.5 (2C), 128.3 (2C), 127.4 (2C), 125.9 (2C), 125.9 (q, *J* = 3.7 Hz), 123.7 (q, *J* =270.9 Hz), 123.2 (2C), 119.6 (2C), 35.3 (2C), 35.3, 31.3 (3C), 30.4 (6C). IR (cm^{-1}): ν 3086.59, 2958.57, 2871.52, 1579.61, 1508.07, 1365.81, 1325.55, 1266.77, 1223.03, 1141.99, 1113.98, 1065.12, 1030.30, 1012.83, 956.55, 862.59, 827.30, 750.87, 739.83, 729.30. HRMS: Calculated for $(M\text{-}BF_4)^+$: 568.3191; found: 568.3188. **PC 7** (2.05 g, 71% yield): ^{1}H NMR (400 MHz, $CDCl_3$) δ 8.14 (dd, *J* = 9.6, 2.0 Hz, 2H), 7.90-7.80 (m, 4H), 7.73-7.64 (m, 2H), 7.60 (d, *J* = 8.4 Hz, 2H), 7.52 (d, *J* = 9.6 Hz, 2H), 7.50-7.42 (m, 2H), 1.50 (s, 9H), 1.31 (s, 18H). ^{13}C NMR (100 MHz, $CDCl_3$) δ 163.70 (d, *J* = 249.8 Hz), 160.1, 155.3, 151.0 (2C), 140.5 (2C), 137.4 (2C), 134.5, 132.1 (d, *J* =8.3 Hz, 2C), 129.2 (d, *J* = 3.5 Hz), 128.3 (2C), 127.4 (2C), 126.2 (2C), 123.6 (2C), 119.5 (2C), 116.3 (d, *J* = 21.7, 2C), 35.3, 31.3 (2C), 30.4 (9C). IR (cm^{-1}): ν 3065.97, 2963.47, 2871.14, 1602.19, 1582.38, 1543.73, 1492.14, 1386.23, 1366.83, 1263.40, 1223.31, 1151.37, 1030.82, 992.05, 957.80, 916.91, 826.42, 732.79. HRMS: Calculated for $(M\text{-}BF_4)^+$: 518.3218; found: 518.3218. **PC 8** (1.98 g, 63% yield): ^{1}H NMR (400 MHz, $CDCl_3$) δ 8.28 (dd, *J* = 9.6, 2.4 Hz, 2H), 7.92 (d, *J* = 8.4 Hz, 2H), 7.78 (s, 2H), 7.67 (dd, *J* = 9.6, 1.6 Hz, 2H), 7.64-7.55 (m, 5H), 1.51 (s, 9H), 1.36 (s, 18H). ^{13}C NMR (100 MHz, $CDCl_3$) δ 156.5, 155.3, 151.4 (2C), 140.5 (2C), 137.4 (2C), 137.2, 134.4, 133.6, 133.4, 132.2, 130.9, 129.7, 128.9, 128.4 (2C), 127.5, 126.4, 125.9, 122.8 (2C), 119.7 (2C), 35.3, 31.3 (2C), 30.5 (9C). IR (cm^{-1}): ν 3058.00, 2960.60, 2927.49, 2870.53, 1594.95, 1544.23, 1488.92, 1387.04, 1264.69, 1152.30, 1090.54, 1031.00, 957.71, 822.92, 734.16. HRMS: Calculated for $(M\text{-}BF_4)^+$: 568.2532; found: 568.2523. **PC 9** (1.60 g, 52% yield): ^{1}H NMR (400 MHz, $CDCl_3$) δ 8.15 (d, *J* = 9.5 Hz, 2H), 7.94 -7.78 (m, 4H), 7.67 (d, *J* = 1.0 Hz, 2H), 7.59-7.49 (m, 3H), 7.48-7.40 (m, 2H), 1.50 (s, 9H), 1.32 (s, 18H). ^{13}C NMR (100 MHz, $CDCl_3$) δ 163.5 (d, *J* = 253.1 Hz), 156.9, 155.3, 151.4 (2C), 140.5 (2C), 137.4 (2C), 134.4, 134.1 (d, *J* = 7.6 Hz), 133.7 (d, *J* = 10.3 Hz), 128.9 (2C), 128.4 (d, *J* = 3.5 Hz), 128.3, 127.5, 126.5, 126.1, 122.8 (2C), 119.7 (2C), 117.4 (d, *J* = 24.9 Hz), 115.7 (d, *J* = 21.2 Hz), 35.3, 31.3 (2C), 30.4 (9C). IR (cm^{-1}): ν 3063.16, 2963.12, 2871.03, 1602.13, 1581.34, 1545.21, 1489.16, 1390.25, 1263.96, 1221.81, 1153.17, 1058.96, 1030.93, 959.67, 919.54, 826.24, 732.65. HRMS: Calculated for $(M\text{-}BF_4)^+$: 552.2827; found: 552.2828.

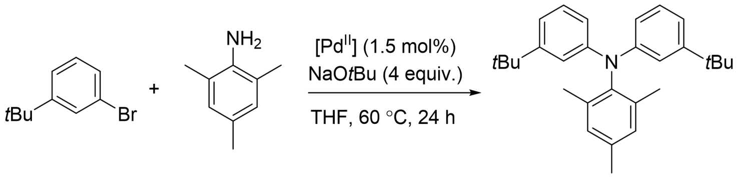

18 **19** **20**

According to the reference,[4] chloro[(tri-tert-butylphosphine)-2-(2-aminobiphenyl)]palladium(II) (38 mg, 0.08 mmol, 1.5 mol%) and NaO*t*Bu (1.92 g, 20 mmol, 4.0 equiv) were added to a dried three-necked flask. The flask was evacuated and filled with argon three times. Dry THF (25 mL, 0.2 M) was added under argon atmosphere, followed by 1-bromo-3-(tert-butyl)benzene **18** (1.7 mL, 10 mmol, 2.0 equiv) and compound **19** (0.70 mL, 5 mmol, 1.0 equiv). The reaction mixture was heated to 60 °C for 24 h. After cooling to room temperature, Et_2O (75 mL) and H_2O (50 mL) were added and stirred for a while. The separated aqueous layer was extracted with Et_2O (50 mL x 3). The combined organic layers were washed with brine, dried over Na_2SO_4, concentrated in vacuo and the residue was purified on silica-gel column chromatography (petroleum ether:EtOAc = 5:1) to give compound **20** as a colorless oil (1.60 g, 80% yield).

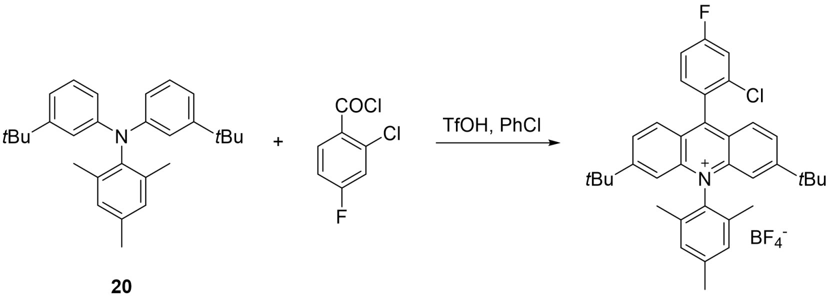

20

PC 11

PC 11 (1.05 g, 67%) were prepared according to the same procedure of **PC 4** from **20** (1.00 g, 2.50 mmol) and 2-chloro-4-fluorobenzoyl chloride (0.67 mL, 2.0 equiv). ^{1}H NMR (400 MHz, $CDCl_3$) δ 8.07-7.75 (m, 5H), 7.63-7.45 (m, 2H), 7.38 (s, 1H), 7.37-7.31 (m, 3H), 2.58 (s, 3H), 1.87 (s, 3H), 1.67 (s, 3H), 1.32 (s, 18H). ^{13}C NMR (100 MHz, $CDCl_3$) δ 165.0, 163.7 (d, J = 253.7 Hz), 157.2 (2C), 142.3 (2C), 141.0 (2C), 133.8, 133.6, 132.0, 131.0 (2C), 130.8, 129.2 (2C), 127.9 (2C), 127.6, 124.3 (2C), 117.6 (d, J = 25.2 Hz), 115.9 (d, J = 21.6 Hz), 112.8 (2C), 36.7 (2C), 30.3 (6C), 21.4, 17.1, 16.7. IR (cm^{-1}): v 3088.44, 2963.27, 2871.77, 1606.98, 1578.24, 1501.57, 1442.27, 1384.66, 1266.66, 1224.12, 1198.12, 1198.76, 1150.53, 1055.03, 1030.61, 991.41, 916.51, 871.84, 857.97, 732.93. HRMS: Calculated for $(M-BF_4)^+$: 538.2671; found: 538.2680.

Preparation of Diaryl Ethers

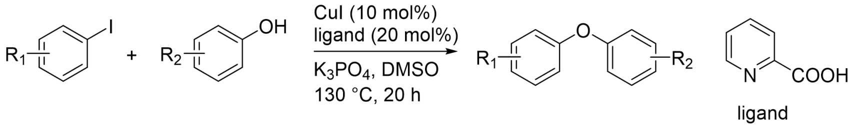

According to the literature,[5] to a 50 mL flame dried three-necked round bottom flask with a stirring bar, CuI (174 mg, 0.94 mmol, 10 mol%), pyridine-2-carboxylic acid (225 mg, 1.88 mmol, 20 mol%), aryl iodide (9.42 mmol) and potassium phosphate (5.91g, 27.84 mmol, 3 equiv) were added. The flask was evacuated and filled with argon three times. DMSO (18 mL) and phenol (11.46 mmol) were added under argon atmosphere. The reaction mixture was stirred at 130 °C for 20 h. The reaction mixture was cooled to room temperature and diluted with water (150 mL). The crude product was extracted with EtOAc (3 x 100 mL). The combined organic layers was washed with brine, dried over Na_2SO_4, concentrated in vacuo and the residue was purified on silica-gel column chromatography (petroleum ether to petroleum ether/EtOAc = 100/1) to give the diaryl ethers.

Methyl 4-phenoxybenzoate (1b)[6]

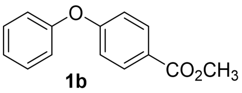

1b

According to the general procedure, the reaction of methyl-4-iodobenzoate (2.47 g, 9.42 mmol) with phenol (1.08 g, 11.42 mmol) produced **1b** as a white solid (0.84 g) in 39% yield. ^{1}H NMR (400 MHz, $CDCl_3$) δ 8.07-7.94 (m, 2H), 7.44-7.34 (m, 2H), 7.23-7.14 (m, 1H), 7.10-7.04 (m, 2H), 7.02-6.94 (m, 2H), 3.90 (s, 3H). ^{13}C NMR (100 MHz, $CDCl_3$) δ 166.6, 161.8, 155.6, 131.7 (2C), 130.0 (2C), 124.5, 124.4, 120.1 (2C), 117.2 (2C), 52.0. MS: m/z: $[M]^+$, 228.08.

4-Phenoxybenzonitrile (1e)[7]

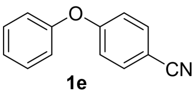

1e

According to the general procedure, the reaction of 4-iodobenzonitrile (2.20 g, 9.42 mmol) with phenol (1.08 g, 11.42 mmol) produced **1e** as a yellow solid (1.30 g) in 71% yield. ^{1}H NMR (400 MHz, $CDCl_3$) δ 7.60 (d, *J* = 8.8 Hz, 2H), 7.46-7.36 (m, 2H), 7.30-7.15 (m, 1H), 7.11-7.05 (d, *J* = 8.0 Hz, 2H), 7.00 (d, *J* = 8.8 Hz, 2H). ^{13}C NMR (100 MHz, $CDCl_3$) δ 161.6, 154.7, 134.1 (2C), 130.2 (2C), 125.1, 120.4 (2C), 118.8, 117.9 (2C), 105.8. MS: m/z: $[M]^+$, 195.06.

1-(4-phenoxyphenyl)ethan-1-one (1f)[7]

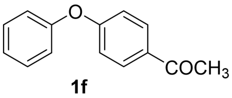

1f

According to the general procedure, the reaction of iodobenzene (1.92 g, 9.42 mmol) with 4-hydroxyacetophenone (1.56 g, 11.42 mmol) produced **1f** as a white solid (0.65 g) in 33% yield. ^{1}H NMR (400 MHz, $CDCl_3$) δ 7.99 -7.89 (m, 2H), 7.46-7.34 (m, 2H), 7.24-7.18 (m, 1H), 7.07 (d, *J* = 8.0 Hz, 2H), 7.04-6.96 (m, 2H), 2.57 (s, 3H). ^{13}C NMR (100 MHz, $CDCl_3$) δ 196.7, 162.0, 155.4, 131.8, 130.6 (2C), 130.0 (2C), 124.6, 120.2 (2C), 117.2 (2C), 26.5. MS: m/z: $[M]^+$, 212.07.

4-phenoxy-1,1'-biphenyl (1h)[6]

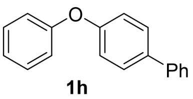

1h

According to the general procedure, the reaction of iodobenzene (1.92 g, 9.42 mmol) with 4-phenylphenol (1.95 g, 11.42 mmol) produced **1h** as a white solid (1.52 g) in 65% yield. ^{1}H NMR (400 MHz, $CDCl_3$) δ 7.64-7.52 (m, 4H), 7.48-7.40 (m, 2H), 7.40-7.31 (m, 3H), 7.18-7.00 (m, 1H), 7.10-6.98 (m, 4H). ^{13}C NMR (100 MHz, $CDCl_3$) δ 157.1, 156.8, 140.5, 136.2, 129.8 (2C), 128.8 (2C), 128.4 (2C), 127.0, 126.9 (2C), 123.4, 119.0 (4C). MS: m/z: $[M]^+$, 246.09.

1-methyl-2-phenoxybenzene (1i)[8]

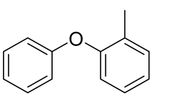

1i

According to the general procedure, the reaction of iodobenzene (1.92 g, 9.42 mmol) with *o*-cresol (1.24 g, 11.42 mmol) produced **1i** as a colorless oil (1.32 g) in 76% yield. ^{1}H NMR (400 MHz, $CDCl_3$) δ 7.36-7.21 (m, 3H), 7.20-7.12 (m, 1H), 7.11-6.99 (m, 2H), 6.96-6.85 (m, 3H), 2.24 (s, 3H). ^{13}C NMR (100 MHz, $CDCl_3$) δ157.9, 154.4, 131.4, 130.0, 129.6 (2C), 127.1, 124.0, 122.3, 119.8, 117.3 (2C), 16.2. MS: m/z: $[M]^+$, 184.07.

1,3-dimethyl-5-phenoxybenzene (1j)[9]

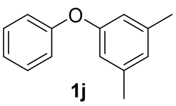

1j

According to the general procedure, the reaction of iodobenzene (1.92 g, 9.42 mmol) with 3,5-dimethylphenol (1.40 g, 11.42 mmol) produced **1j** as a colorless oil (1.49 g) in 80% yield. ^{1}H NMR (400 MHz, $CDCl_3$) δ 7.38-7.27 (m, 2H), 7.13-7.02 (m, 1H), 6.99 (d, *J* = 8.4 Hz, 2H), 6.74 (s, 1H), 6.63 (s, 2H), 2.28 (s, 6H). ^{13}C NMR (100 MHz, $CDCl_3$) δ 157.4, 157.1, 139.6 (2C), 129.6 (2C), 125.0, 122.9, 118.8 (2C), 116.6 (2C), 21.3 (2C). MS: m/z: $[M]^+$, 198.09.

1-methoxy-3-phenoxybenzene (1k)[8]

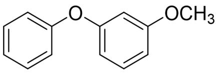

1k

According to the general procedure, the reaction of iodobenzene (1.92 g, 9.42 mmol) with 3-methoxyphenol (1.42 g, 11.42 mmol) produced **1k** as a colorless oil (1.40 g) in 74% yield. ^{1}H NMR (400 MHz, $CDCl_3$) δ 7.39-7.28 (m, 2H), 7.27-7.17 (m, 1H), 7.15-7.05 (m, 1H), 7.06-6.97 (m, 2H), 6.65 (dd, *J* = 9.2, 2.0 Hz, 1H), 6.62-6.53 (m, 2H), 3.77 (s, 3H). ^{13}C NMR (100 MHz, $CDCl_3$) δ 160.9, 158.5, 156.9, 130.1, 129.7 (2C), 123.3, 119.0 (2C), 110.9, 108.8, 104.8, 55.3. MS: m/z: $[M]^+$, 200.08.

3,3'-oxybis(methylbenzene) (1l)[5]

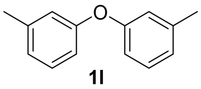

1l

According to the general procedure, the reaction of 3-iodotoluene (2.05 g, 9.42 mmol) with m-cresol (1.24 g, 11.42 mmol) produced **1l** as a light yellow oil (1.66 g) in 89% yield. ^{1}H NMR (400 MHz, $CDCl_3$) δ 7.26-7.13 (m, 2H), 6.90 (d, *J* = 7.6 Hz, 2H), 6.85-6.74 (m, 4H), 2.32 (s, 6H). ^{13}C NMR (100 MHz, $CDCl_3$) δ 157.3 (2C), 139.8 (2C), 129.4 (2C), 123.9 (2C), 119.5 (2C), 115.8 (2C), 21.4 (2C). MS: m/z: $[M]^+$, 198.09.

3,3'-oxybis(methoxybenzene) (1m)[5]

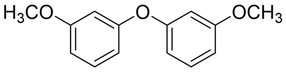

1m

According to the general procedure, the reaction of 3-iodoanisole (2.20 g, 9.42 mmol) with 3-methoxyphenol (1.42 g, 11.42 mmol) produced **1m** as a colorless oil (1.84 g) in 85% yield. ^{1}H NMR (400 MHz, $CDCl_3$) δ 7.28-7.17 (m, 2H), 6.76-6.64 (m, 2H), 6.64-6.54 (m, 4H), 3.78 (s, 6H). ^{13}C NMR (100 MHz, $CDCl_3$) δ 160.9 (2C), 158.2 (2C), 130.1 (2C), 111.1 (2C), 109.0 (2C), 105.0 (2C), 55.3 (2C). MS: m/z: $[M]^+$, 230.08.

4,4'-oxybis(chlorobenzene) (1o)[10]

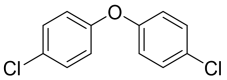

1o

According to the general procedure, the reaction of 1-chloro-4-iodobenzene (2.25 g, 9.42 mmol) with phenol (1.47 g, 11.42 mmol) produced **1o** as a light yellow oil (1.65 g) in 73% yield. ^{1}H NMR (400 MHz, $CDCl_3$) δ 7.35-7.26 (m, 4H), 7.00-6.85 (m, 4H). ^{13}C NMR (100 MHz, $CDCl_3$) δ 155.5 (2C), 129.8 (4C), 128.6 (2C), 120.1 (4C). MS: m/z: $[M]^+$, 237.99.

Methyl 4-(o-tolyloxy)benzoate (1q)[7]

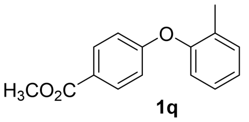

1q

According to the general procedure, the reaction of methyl 4-iodobenzoate (2.46 g, 9.42 mmol) with o-cresol (1.26 g, 11.42 mmol) produced **1q** as a colorless oil (1.80 g) in 79% yield. ^{1}H NMR (400 MHz, $CDCl_3$) δ 7.98 (d, *J* = 8.8 Hz, 2H), 7.27 (d, *J* = 7.6 Hz, 1H), 7.25-7.18 (m, 1H), 7.16-7.10 (m, 1H), 6.98 (d, *J* = 8.0 Hz, 1H), 6.87 (d, *J* = 8.8 Hz, 2H), 3.88 (s, 3H), 2.18 (s, 3H). ^{13}C NMR (100 MHz, $CDCl_3$) δ 166.6, 162.0, 153.0, 131.7 (3C), 130.5, 127.4, 125.1, 123.7, 120.9, 115.9 (2C), 51.9, 16.0. MS: m/z: $[M]^+$, 242.10.

5-(m-tolyloxy)benzonitrile (1r)[11]

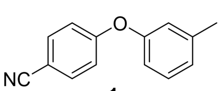

1r

According to the general procedure, the reaction of 4-iodobenzonitrile (2.20 g, 9.42 mmol) with m-cresol (1.24 g, 11.42 mmol) produce **1r** as a colorless oil (1.77 g) in 90% yield. ^{1}H NMR (400 MHz, $CDCl_3$) δ 7.59 (d, J = 8.0 Hz, 2H), 7.35-7.22 (m, 1H), 7.04 (d, J = 7.6 Hz, 1H), 6.99 (d, J = 8.4 Hz, 2H), 6.87-6.83 (m, 2H), 2.37 (s, 3H). ^{13}C NMR (100 MHz, $CDCl_3$) δ 161.8, 154.7, 140.5, 134.1 (2C), 129.9, 125.9, 121.0, 118.9, 117.8 (2C), 117.3, 105.6, 21.3. MS: m/z: $[M]^+$, 209.09.

1-methoxy-2-(4-(trifluoromethyl)phenoxy)benzene (1s)

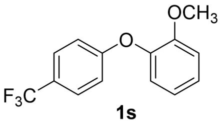

1s

According to the general procedure, the reaction of 4-iodobenzotrifluoride (2.56 g, 9.42 mmol) with guaiacol (1.40 g, 11.42 mmol) produced **1s** as a colorless oil (0.78 g) in 31% yield. ^{1}H NMR (400 MHz, $CDCl_3$) δ 7.53 (d, J = 8.8 Hz, 2H), 7.25-7.16 (m, 1H), 7.11-7.01 (m, 2H), 7.01-6.91 (m, 3H), 3.81 (s, 3H). ^{13}C NMR (100 MHz, $CDCl_3$) δ 160.9, 151.7, 143.3, 126.9 (q, J = 3.8 Hz, 2C), 126.1, 124.1 (q, J = 32.4 Hz), 123.8 (q, J = 269.6 Hz), 122.3, 121.3, 116.2 (2C), 112.9, 55.9. MS: m/z: $[M]^+$, 268.07.

1-methoxy-2-phenoxybenzene (1t)[5]

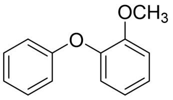

1t

According to the general procedure, the reaction of iodobenzene (1.92 g, 9.42 mmol) with guaiacol (1.42 g, 11.42 mmol) produced **1t** as a white solid (1.30 g) in 69% yield. ^{1}H NMR (400 MHz, $CDCl_3$) δ 7.33-7.24 (m, 2H), 7.20-7.08 (m, 1H), 7.07-6.81 (m, 6H), 3.82 (s, 3H). ^{13}C NMR (100 MHz, $CDCl_3$) δ 157.9, 151.4, 145.0, 129.5 (2C), 124.7, 122.4, 121.0, 121.0, 117.2 (2C), 112.7, 55.9. MS: m/z: $[M]^+$, 200.10.

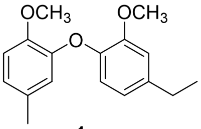

1y

According to the general procedure, ^{1}H NMR (400 MHz, $CDCl_3$) δ 6.91-6.79 (m, 3H), 6.76 (d, J = 8.0 Hz, 1H), 6.69 (dd, J = 8.0, 1.6 Hz, 1H), 6.59 (d, J = 1.6 Hz, 1H), 3.85 (s, 3H), 3.84 (s, 3H), 2.64 (q, J = 7.6 Hz, 2H), 2.20 (s, 3H), 1.25 (t, J = 7.6 Hz, 3H). ^{13}C NMR (100 MHz, $CDCl_3$) δ 150.41, 148.19, 146.20, 143.64, 140.11, 130.49, 123.57, 119.80, 119.07, 118.96, 112.45, 112.35, 56.18, 55.96, 28.62, 20.63, 15.58. MS: m/z: $[M]^+$, 272.14.

Supplementary Table 1 Lewis Acid and Solvent Screening.

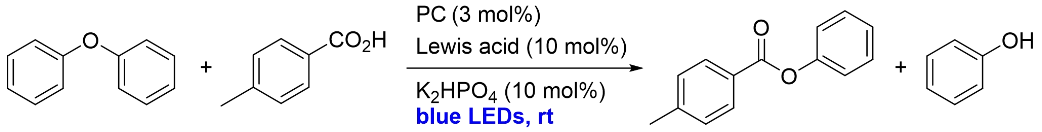

1a **2a** (1.2 equiv) **3a** **4a**

Entry	Lewis acid	Solvent	Wavelength (nm)	**3a** yield(%)	**4a** yield(%)[b]
1	$Cu(OAc)_2$	CH_3CN	450-455	9	6
2	$Cu(acac)_2$	CH_3CN	450-455	10	9
3	$Cu(OTf)_2$	CH_3CN	450-455	—	—
4	$Ni(acac)_2$	CH_3CN	450-455	6	7
5	$Fe(acac)_2$	CH_3CN	450-455	7	<5
6	$Zn(acac)_2$	CH_3CN	450-455	—	—
7	$Cu(TMHD)_2$	MeOH	425-430	—	—
8	$Cu(TMHD)_2$	DCE	425-430	—	—
9	$Cu(TMHD)_2$	EtOAc	425-430	—	—
10	$Cu(TMHD)_2$	acetone	425-430	<5	<5

[a]Reaction conditions: **1a** (0.24 mmol), **2a** (0.2 mmol), Acr^+-Mes ClO_4^- (**PC 1**) (3.0 mol %), Lewis acid (10 mol %), solvent (2.0 mL), irradiation with blue LEDs for 30 h, ^1H NMR yields of **3a** and **4a** were reported by using $Cl_2CHCHCl_2$ as an internal standard.
[b]Due to the volatility of phenol (during work-up), phenol was obtained in slightly lower yields than **3a**.

General Procedure for the C-O Bond Cleavage of Diaryl Ethers

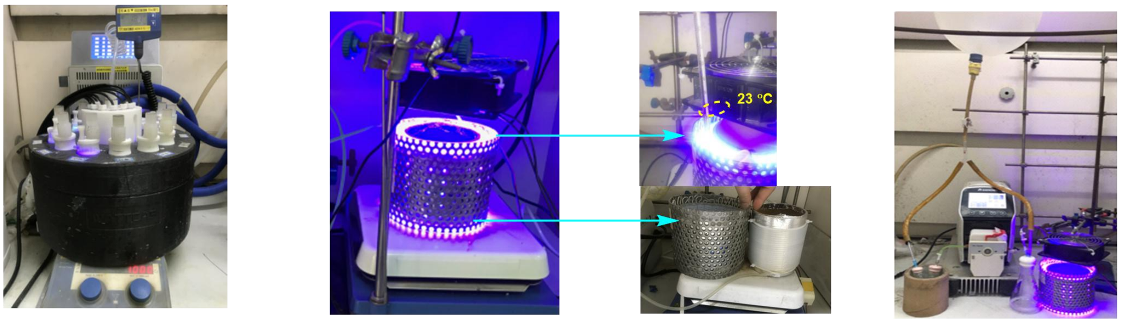

Wattecs Parallel Reactor
(a)

Stop-Flow Reactor
(b)

Continuous-Flow Reactor
(c)

Supplementary Figure 1 Photoreaction equipments. a Wattecs Parallel Reactor; **b** Stop-Flow Reactor; **c** Continuous-Flow Reactor.

Condition A: To a quartz tube equipped with a magnetic stirring bar, **PC 9** (0.015 mmol, 3.0 mol%, 9.60 mg), compound **1** (0.50 mmol), compound **2** (0.60 mmol), bis(2,2,6,6-tetra-Methyl-3,5-heptanedionato)copper(II) ($Cu(TMHD)_2$) (0.05 mmol, 10 mol%, 21.5 mg) were added. The tube was evacuated and filled with argon three times with each cycle in 15 minutes. Freshly distilled CH_3CN (5.0 mL) was then added into the tube via a syringe under argon atmosphere, then stirred and irradiated with 425-430 nm blue LEDs at ambient temperature (19-21 °C) in a Wattecs Parallel Reactor (Supplementary Figure 1 (a)) for 30-60 h. After the reaction, the solvent was removed in vacuo and the residue was purified by column chromatography (petroleum ether/EtOAc = 200/1 - 5/1) to afford compound **3** and **4**.

Condition B: To a quartz tube equipped with a magnetic stirring bar were added **PC 9** (0.015 mmol, 3.0 mol%, 9.60 mg), compound **1** (0.50 mmol), compound **2** (0.60 mmol), $Cu(TMHD)_2$ (0.05 mmol, 10 mol%, 21.5 mg). The tube was evacuated and filled with argon three times with a cycle in 15 minutes. Freshly distilled CH_3CN (5.0 mL) and $PhCF_3$ (2.5 mL) were then added into the tube via a syringe under argon atmosphere, then stirred and irradiated with 425-430 nm blue LEDs at ambient temperature (19-21 °C) in a Wattecs Parallel Reactor for 60 h. After the reaction, the solvent was removed in vacuo and the residue was purified by column chromatography(petroleum ether/EtOAc = 200/1 - 5/1) to afford compounds **3** and **4**.

Condition C: Under argon atmosphere, a solution of compound **1** (0.20 mmol), compound **2** (0.24 mmol), **PC 9** (0.006 mmol, 3.0 mol%), and $Cu(TMHD)_2$ (0.02 mmol, 10 mol%) in anhydrous CH_3CN (2.0 mL) and $PhCF_3$ (1 mL) was added into a sealed vial. Then the solution was pumped into a stop-flow micro tube (total volume 3.0 mL), which was made of PTFE tubing (O.D. 1/16", I.D. 0.03", 1000 cm), via a syringe. The valves were closed and the tube irradiated with 420-430 nm 25 W blue LEDs in a Stop-Flow micro tubing

reactor (Supplementary Figure 1 (b)) at ambient temperature (23 °C) for 60 h. After the reaction, the solvent was removed in vacuo and the residue was purified by column chromatography (petroleum ether/EtOAc = 200/1 - 5/1) to afford compound **3** and **4**.

The reaction of diphenyl ether (1a) with 4-methylbenzoic acid (2a) produced phenyl 4-methylbenzoate (3a)[12] **and phenol (4a)**

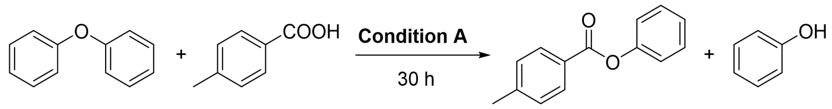

1a **2a** **3a** **4a**

According to the condition A, **3a** was obtained as a white solid (80.6 mg) in 76% yield, **4a** was obtained as a solid (32.4 mg) in 69% yield. **3a,** ^{1}H NMR (400 MHz, $CDCl_3$) δ 8.10 (d, *J* = 8.1 Hz, 2H), 7.47-7.38 (m, 2H), 7.31 (d, *J* = 8.0 Hz, 2H), 7.27-7.24 (m, 1H), 7.23-7.16 (m, 2H), 2.45 (s, 3H). ^{13}C NMR (100 MHz, $CDCl_3$) δ 165.2, 151.0, 144.4, 130.2 (2C), 129.4 (2C), 129.2 (2C), 126.7, 125.7, 121.7 (2C), 21.7. MS: m/z: $[M]^+$, 212.08. **4a**, ^{1}H NMR (400 MHz, $CDCl_3$) δ 7.32-7.20 (m, 2H), 7.02-6.92 (m, 1H), 6.86 (d, *J* = 8.4 Hz, 2H), 4.76 (br s, 1H). ^{13}C NMR (100 MHz, $CDCl_3$) δ 155.3, 129.7 (2C), 120.8, 115.3 (2C). MS: m/z: $[M]^+$, 94.05.

The reaction of diphenyl ether (1a) with 4-methoxybenzoic acid (2b) produced phenyl 4-methoxybenzoate (3ab)[12] **and phenol (4a)**

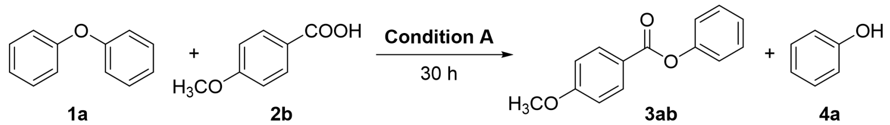

1a **2b** **3ab** **4a**

According to the condition A, **3ab** was obtained as a white solid (69.6 mg) in 61% yield, **4a** was obtained as a colorless solid (28.2 mg) in 60% yield. **3ab**, ^{1}H NMR (400 MHz, $CDCl_3$) δ 8.22-8.10 (m, 2H), 7.49-7.36 (m, 2H), 7.29 -7.23 (m, 1H), 7.23-7.17 (m, 2H), 7.04-6.93 (m, 2H), 3.88 (s, 3H). ^{13}C NMR (100 MHz, $CDCl_3$) δ 164.9, 163.8, 151.0, 132.2 (2C), 129.4 (2C), 125.7, 121.8, 121.7 (2C), 113.7 (2C), 55.5. MS: m/z: $[M]^+$, 228.09.

The reaction of diphenyl ether (1a) with 4-(*tert*-butyl)benzoic acid (2c) produced phenyl 4-(*tert*-butyl)benzoate (3ac)[13] **and phenol (4a)**

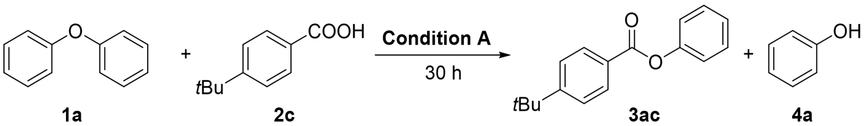

1a **2c** **3ac** **4a**

According to the condition A, **3ac** was obtained as a white solid (82.7 mg) in 65% yield, **4a** was obtained as a colorless solid (26.3 mg) in 56% yield. **3ac**, ^{1}H NMR (400 MHz, $CDCl_3$) δ 8.14 (d, *J* = 8.4 Hz, 2H), 7.53 (d, *J* = 8.4 Hz, 2H), 7.47-7.37 (m, 2H), 7.31-7.24 (m, 1H), 7.20 (d, *J* = 8.4 Hz, 2H), 1.37 (s, 9H). ^{13}C NMR (100 MHz, $CDCl_3$) δ 165.2, 157.4, 151.0, 130.1 (2C), 129.4 (2C), 126.7, 125.8, 125.6 (2C), 121.8 (2C), 35.2, 31.1 (3C). MS: m/z: $[M]^+$, 254.15.

The reaction of diphenyl ether (1a) with benzoic acid (2d) produced phenyl benzoate (3ad)[13] **and phenol (4a)**

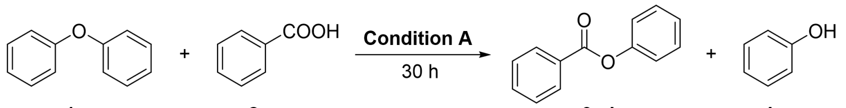

1a **2c** **3ad** **4a**

According to the condition A, **3ad** was obtained as a white solid (54.5 mg) in 55% yield, **4a** was obtained as a colorless solid (23.5 mg) in 50% yield. **3ad**, ^{1}H NMR (400 MHz, $CDCl_3$) δ 8.21 (d, *J* = 7.6 Hz, 2H), 7.68-7.60 (m, 1H), 7.57-7.47 (m, 2H), 7.47-7.39 (m, 2H), 7.32-7.25 (m, 1H), 7.22 (d, *J* = 7.8 Hz, 2H). ^{13}C NMR (100 MHz, $CDCl_3$) δ 165.2, 150.9, 133.6, 130.2 (2C), 129.5, 129.4 (2C), 128.6 (2C), 125.9, 121.7 (2C). MS: m/z: $[M]^+$, 198.07.

The reaction of diphenyl ether (1a) with 4-fluorobenzoic acid (2e) produced phenyl 4-fluorobenzoate (3ae)[12] **and phenol (4a)**

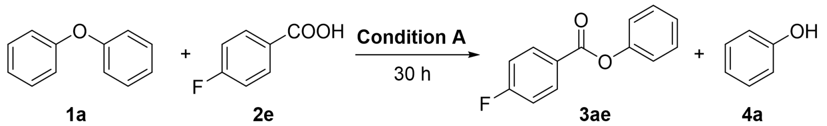

1a **2e** **3ae** **4a**

According to the condition A, **3ae** was obtained as a colorless crystal (97.3 mg) in 90% yield, **4a** was obtained as a colorless solid (39.6 mg) in 84% yield. **3ae**, ^{1}H NMR (400 MHz, $CDCl_3$) δ 8.30-8.18 (m, 2H), 7.49-7.40 (m, 2H), 7.33-7.26 (m, 1H), 7.24-7.14 (m, 4H). ^{13}C NMR (100 MHz, $CDCl_3$) δ 166.1 (d, J = 255.1 Hz), 164.2, 150.8, 132.8 (d, J = 10.0 Hz, 2C), 129.5 (2C), 126.0, 125.8 (d, J = 2.9 Hz), 121.6 (2C), 115.8 (d, J = 22.0 Hz, 2C). MS: m/z: $[M]^+$, 216.06.

The reaction of diphenyl ether (1a) with 4-chlorobenzoic acid (2f) produced phenyl 4-chlorobenzoate (3af)[12] and phenol (4a)

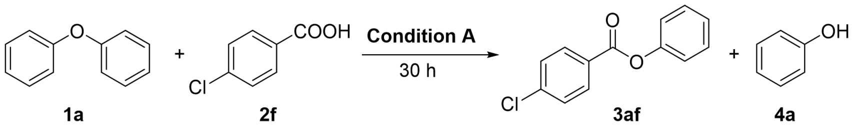

1a **2f** **3af** **4a**

According to the condition A, **3af** was obtained as a white solid (105.9 mg) in 91% yield, **4a** was obtained as a colorless solid (39.5 mg) in 84% yield. **3af**, ^{1}H NMR (400 MHz, $CDCl_3$) δ 8.18-8.09 (m, 2H), 7.52-7.46 (m, 2H), 7.46 -7.39 (m, 2H), 7.32-7.25 (m, 1H), 7.24-7.17 (m, 2H). ^{13}C NMR (100 MHz, $CDCl_3$) δ 164.3, 150.7, 140.1, 131.5 (2C), 129.5 (2C), 128.9 (2C), 128.0, 126.0, 121.6 (2C). MS: m/z: $[M]^+$, 232.01.

The reaction of diphenyl ether (1a) with 4-bromobenzoic acid (2g) produced phenyl 4-bromobenzoate (3ag)[14] and phenol (4a)

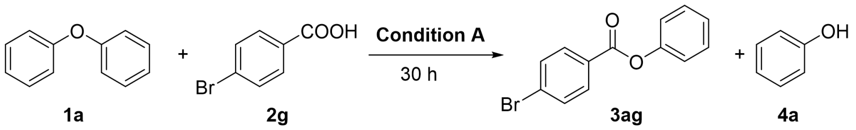

1a **2g** **3ag** **4a**

According to the condition A, **3ag** was obtained as a white solid (120.6 mg) in 87% yield, **4a** was obtained as a colorless solid (38.5 mg) in 82% yield. **3ag**, ^{1}H NMR (400 MHz, $CDCl_3$) δ 8.10-8.02 (m, 2H), 7.72-7.61 (m, 2H), 7.48 -7.38 (m, 2H), 7.32-7.25 (m, 1H), 7.23-7.16 (m, 2H). ^{13}C NMR (100 MHz, $CDCl_3$) δ 164.5, 150.7, 131.9 (2C), 131.6 (2C), 129.5 (2C), 128.8, 128.4, 126.0, 121.6 (2C). MS: m/z: $[M]^+$, 275.95.

The reaction of diphenyl ether (1a) with 4-nitrobenzoic acid (2h) produced phenyl 4-nitrobenzoate (3ah)[12] and phenol (4a)

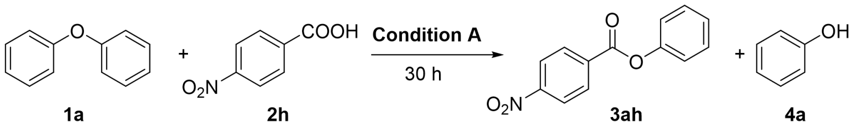

1a **2h** **3ah** **4a**

According to the condition A, **3ah** was obtained as a white solid (93.6 mg) in 77% yield, **4a** was obtained as a colorless solid (32.9 mg) in 70% yield. **3ah**, ^{1}H NMR (400 MHz, $CDCl_3$) δ 8.47-8.30 (m, 4H), 7.53-7.40 (m, 2H), 7.35-7.28 (m, 1H), 7.24 (d, J = 8.0 Hz, 2H).^{13}C NMR (100 MHz, $CDCl_3$) δ 163.3, 150.9, 150.5, 134.9, 131.3 (2C), 129.7 (2C), 126.4, 123.7 (2C), 121.4 (2C). MS: m/z: $[M]^+$, 243.06.

The reaction of diphenyl ether (1a) with 4-formylbenzoic acid (2i) produced phenyl 4-formylbenzoate (3ai)[15] and phenol (4a)

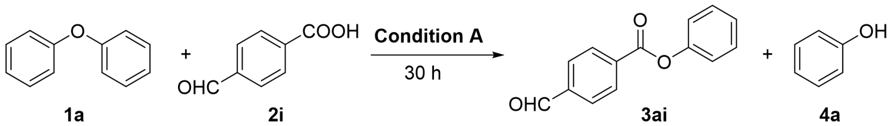

1a **2i** **3ai** **4a**

According to the condition A, **3ai** was obtained as a white solid (80.3 mg) in 71% yield, **4a** was obtained as a colorless solid (30.5 mg) in 65% yield. **3ai**, ^{1}H NMR (400 MHz, $CDCl_3$) δ 10.14 (s, 1H), 8.37 (d, J = 8.3 Hz, 2H), 8.02 (d, J = 8.3 Hz, 2H), 7.51-7.40 (m, 2H), 7.34-7.27 (m, 1H), 7.25-7.18 (m, 2H). ^{13}C NMR (100 MHz, $CDCl_3$) δ 191.5, 164.1, 150.6, 139.5, 134.4, 130.7 (2C), 129.6 (2C), 129.5 (2C), 126.2, 121.5 (2C). MS: m/z: $[M]^+$, 226.04.

The reaction of diphenyl ether (1a) with 3-methylbenzoic acid (2j) produced phenyl 3-methylbenzoate (3aj)[12] and phenol (4a)

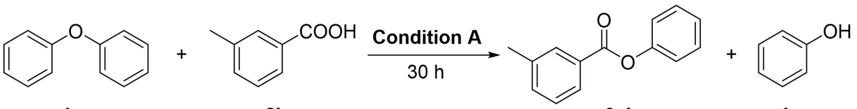

1a 2j 3aj 4a

According to the condition A, **3aj** was obtained as a white solid (64.7 mg) in 61% yield, **4a** was obtained as a colorless solid (28.2 mg) in 60% yield. **3aj**, ^{1}H NMR (400 MHz, $CDCl_3$) δ 8.01 (d, *J* = 8.3 Hz, 2H), 7.50-7.37 (m, 4H), 7.31-7.24 (m, 1H), 7.21 (d, *J* = 8.1 Hz, 2H), 2.45 (s, 3H). ^{13}C NMR (100 MHz, $CDCl_3$) δ 165.3, 151.0, 138.4, 134.3, 130.7, 129.5 (2C), 128.4, 127.3, 125.8, 121.7 (2C), 21.3. MS: m/z: $[M]^+$, 212.07.

The reaction of diphenyl ether (1a) with 3-fluorobenzoic acid (2k) produced phenyl 3-fluorobenzoate (3ak)[12] and phenol (4a)

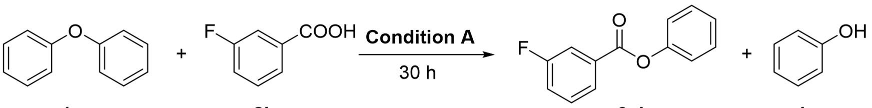

1a 2k 3ak 4a

According to the condition A, **3ak** was obtained as a white crystal (73.5 mg) in 81% yield, **4a** was obtained as a colorless solid (30.7 mg) in 74% yield. **3ak**, ^{1}H NMR (400 MHz, $CDCl_3$) δ 8.01 (d, *J* = 7.8 Hz, 1H), 7.93-7.81 (m, 1H), 7.55-7.47 (m, 1H), 7.47-7.39 (m, 2H), 7.39-7.27 (m, 2H), 7.21 (d, *J* = 8.0 Hz, 2H). ^{13}C NMR (100 MHz, $CDCl_3$) δ 164.0 (d, *J* = 3.0 Hz), 162.5 (d, *J* = 246.0 Hz), 150.7, 131.7 (d, *J* = 8.0 Hz), 130.2 (d, *J* = 8.0 Hz), 129.5 (2C), 126.1, 125.9 (d, *J* = 3.0 Hz), 121.5 (2C), 120.6 (d, *J* = 21.0 Hz), 117.0 (d, *J* = 23.0 Hz). MS: m/z: $[M]^+$, 216.07.

The reaction of diphenyl ether (1a) with 3-chlorobenzoic acid (2l) produced phenyl 3-chlorobenzoate (3al)[12] and phenol (4a)

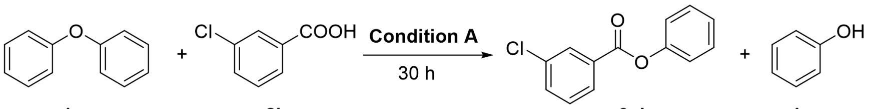

1a 2l 3al 4a

According to the condition A, **3al** was obtained as a white solid (99.9 mg) in 86% yield, **4a** was obtained as a colorless solid (35.7 mg) in 76% yield. **3al**, ^{1}H NMR (400 MHz, $CDCl_3$) δ 8.23-8.15 (m, 1H), 8.13-8.05 (m, 1H), 7.64-7.55 (m, 1H), 7.49-7.39 (m, 3H), 7.33-7.25 (m, 1H), 7.23-7.16 (m, 2H). ^{13}C NMR (100 MHz, $CDCl_3$) δ 163.9, 150.7, 134.7, 133.6, 131.3, 130.1, 129.9, 129.5 (2C), 128.2, 126.1, 121.5 (2C). MS: m/z: $[M]^+$, 232.04.

The reaction of diphenyl ether (1a) with 3-bromobenzoic acid (2m) produced phenyl 3-bromobenzoate (3am)[13] and phenol (4a)

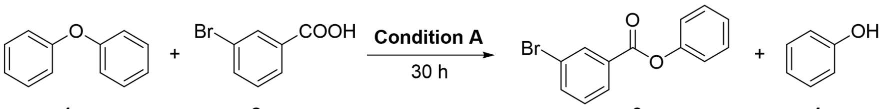

1a 2m 3am 4a

According to the condition A, **3am** was obtained as a white solid (80.6 mg) in 76% yield, **4a** was obtained as a colorless solid (35.7 mg) in 76% yield. **3am**, ^{1}H NMR (400 MHz, $CDCl_3$) δ 8.38-8.31 (m, 1H), 8.14 (d, *J* = 7.6 Hz, 1H), 7.83-7.72 (m, 1H), 7.50-7.37 (m, 3H), 7.33-7.26 (m, 1H), 7.21 (d, *J* = 8.0 Hz, 2H). ^{13}C NMR (100 MHz, $CDCl_3$) δ 163.9, 150.7, 136.5, 133.1, 131.5, 130.1, 129.5 (2C), 128.7, 126.1, 122.6, 121.6 (2C). MS: m/z: $[M]^+$, 275.98.

The reaction of diphenyl ether (1a) with 3-iodobenzoic acid (2n) produced phenyl 3-iodobenzoate (3an)[13] and phenol (4a)

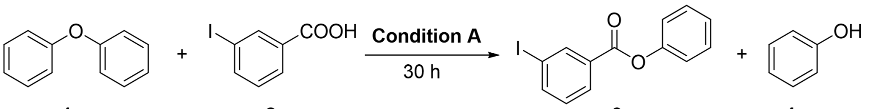

1a 2n 3an 4a

According to the condition A, **3an** was obtained as a white crystal (116.9 mg) in 72% yield, **4a** was obtained as a colorless solid (31.9 mg) in 68% yield. **3an**, ^{1}H NMR (400 MHz, $CDCl_3$) δ 8.60-8.49 (m, 1H), 8.19-8.13 (m, 1H), 8.00-7.90 (m, 1H), 7.49-7.39 (m, 2H), 7.35-7.15 (m, 4H). ^{13}C NMR (100 MHz, $CDCl_3$) δ 163.7, 150.6, 142.3, 138.9, 131.4, 130.2, 129.5 (2C), 129.3, 126.1, 121.5 (2C), 93.9. MS: m/z: $[M]^+$, 323.99.

The reaction of diphenyl ether (1a) with 3-nitrobenzoic acid (2o) produced phenyl 3-nitrobenzoate (3ao)[16] and phenol (4a)

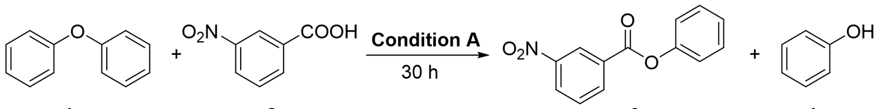

1a **2o** **3ao** **4a**

According to the condition A, **3ao** was obtained as a white solid (86.9 mg) in 71% yield, **4a** was obtained as a colorless solid (32.9 mg) in 70% yield. **3ao**, ^{1}H NMR (400 MHz, $CDCl_3$) δ 9.08-8.99 (m, 1H), 8.57-8.42 (m, 2H), 7.80-7.66 (m, 1H), 7.54-7.40 (m, 2H), 7.35-7.27 (m, 1H), 7.27-7.16 (m, 2H). ^{13}C NMR (100 MHz, $CDCl_3$) δ 163.0, 150.4, 148.3, 135.7, 131.3, 129.8, 129.6 (2C), 127.9, 126.3, 125.0, 121.4 (2C). MS: m/z: $[M]^+$, 243.06.

The reaction of diphenyl ether (1a) with 2-fluorobenzoic acid (2p) produced phenyl 2-fluorobenzoate (3ap)[12] and phenol (4a)

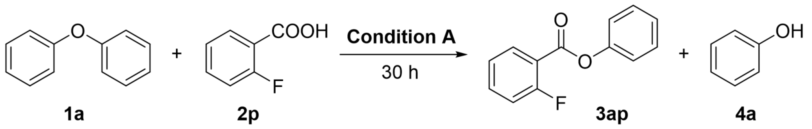

1a **2p** **3ap** **4a**

According to the condition A, **3ap** was obtained as a white solid (68.1 mg) in 63% yield, **4a** was obtained as a colorless solid (28.2 mg) in 60% yield. **3ap**, ^{1}H NMR (400 MHz, $CDCl_3$) δ 8.15-8.03 (m, 1H), 7.68-7.53 (m, 1H), 7.47-7.35 (m, 2H), 7.31-7.11 (m, 5H). ^{13}C NMR (100 MHz, $CDCl_3$) δ 162.7 (d, J = 4.0 Hz), 162.3 (d, J = 259.8 Hz), 150.6, 135.2 (d, J = 9.0 Hz), 132.5, 129.5 (2C), 126.0, 124.1 (d, J = 4.0 Hz), 121.6 (2C), 118.1 (d, J = 9.4 Hz), 117.2 (d, J = 22.2 Hz). MS: m/z: $[M]^+$, 216.07.

The reaction of diphenyl ether (1a) with thiophene-2-carboxylic acid (2q) produced phenyl thiophene-2-carboxylate (3aq)[12] and phenol (4a)

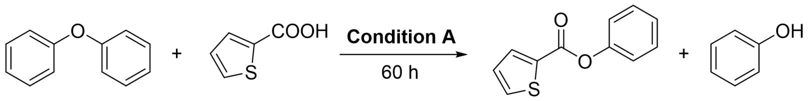

1a **2q** **3aq** **4a**

According to the condition A, **3aq** was obtained as a white solid (55.0 mg) in 54% yield, **4a** was obtained as a colorless solid (21.2 mg) in 54% yield. **3aq**, ^{1}H NMR (400 MHz, $CDCl_3$) δ 7.98 (dd, J = 4.0, 0.8 Hz, 1H), 7.66 (dd, J = 4.8, 1.2 Hz, 1H), 7.51-7.37 (m, 2H), 7.33-7.04 (m, 4H). ^{13}C NMR (100 MHz, $CDCl_3$) δ 160.6, 150.6, 134.7, 133.5, 132.9, 129.5 (2C), 128.0, 126.0, 121.6 (2C). MS: m/z: $[M]^+$, 204.00.

The reaction of diphenyl ether (1a) with thiophene-3-carboxylic acid (2r) produced phenyl thiophene-3-carboxylate (3ar)[12] and phenol (4a)

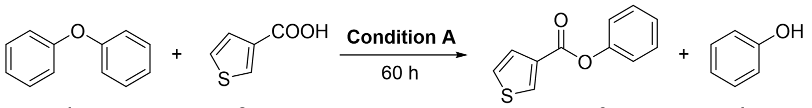

1a **2r** **3ar** **4a**

According to the condition A, **3ar** was obtained as a white solid (50.0 mg) in 49% yield, **4a** was obtained as a colorless solid (18.8 mg) in 40% yield. **3ar**, ^{1}H NMR (400 MHz, $CDCl_3$) δ 8.31 (dd, J = 3.2, 1.2 Hz, 1H), 7.67 (dd, J = 4.8, 1.2 Hz, 1H), 7.48-7.34 (m, 3H), 7.31-7.23 (m, 1H), 7.23-7.15 (m, 2H). ^{13}C NMR (100 MHz, $CDCl_3$) δ 161.0, 150.7, 134.0, 132.9, 129.5 (2C), 128.2, 126.3, 125.9, 121.7 (2C). MS: m/z: $[M]^+$, 204.00.

The reaction of methyl 4-phenoxybenzoate (1b) with 4-methylbenzoic acid (2a) produced phenyl 4-methylbenzoate (3a)[12] and methyl 4-hydroxybenzoate (4b)

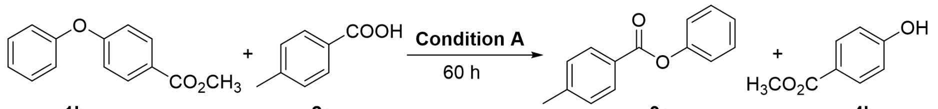

1b **2a** **3a** **4b**

According to the condition A, **3a** was obtained as a white solid (74.3 mg) in 70% yield, **4b** was obtained as a white solid (54.0 mg) in 71% yield. **4b**, ^{1}H NMR (400 MHz, $CDCl_3$) δ 7.96 (d, J = 8.7 Hz, 2H), 6.89 (d, J = 8.7 Hz, 2H), 6.43 (br s, 1H), 3.90 (s, 3H). ^{13}C NMR

(100 MHz, $CDCl_3$) δ 167.0, 159.8, 131.9 (2C), 122.7, 115.2 (2C), 52.0. MS: m/z: $[M]^+$, 152.06.

The reaction of 1-phenoxy-4-(trifluoromethyl)benzene (1c) with 4-methylbenzoic acid (2a) produced phenyl 4-methylbenzoate (3a)[12] and 4-(trifluoromethyl)phenol (4c)

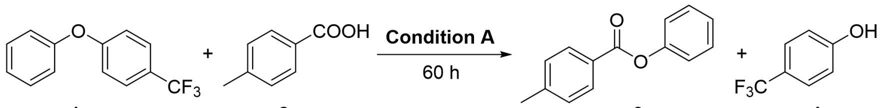

1c 2a 3a 4c

According to the condition A, **3a** was obtained as a white solid (78.5 mg) in 74% yield, **4c** was obtained as a white solid (55.9 mg) in 69% yield. **4c**, ^{1}H NMR (400 MHz, $CDCl_3$) δ 7.51 (d, J = 8.4 Hz, 2H), 6.90 (d, J = 8.5 Hz, 2H), 5.13 (s, 1H). ^{13}C NMR (100 MHz, $CDCl_3$) δ 158.0, 127.2 (q, J = 3.8 Hz), 124.3 (q, J = 269.6 Hz), 123.0 (q, J = 32.6 Hz), 115.4. MS: m/z: $[M]^+$, 162.06.

The reaction of 1-nitro-4-phenoxybenzene (1d) with 4-methylbenzoic acid (2a) produced phenyl 4-methylbenzoate (3a)[12] and 4-nitrophenol (4d)

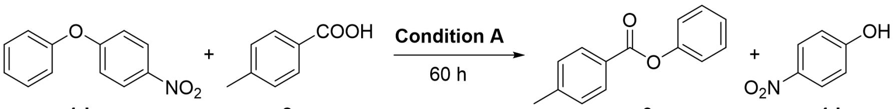

1d 2a 3a 4d

According to the condition A, **3a** was obtained as a white solid (70.0 mg) in 66% yield, **4d** was obtained as a white solid (43.8 mg) in 63% yield. **4d**, ^{1}H NMR (400 MHz, $CDCl_3$) δ 8.19 (d, J = 9.0 Hz, 2H), 6.94 (d, J = 9.0 Hz, 2H), 5.91 (s, 1H). ^{13}C NMR (100 MHz, $CDCl_3$) δ 161.1, 141.8, 126.3 (2C), 115.6 (2C). MS: m/z: $[M]^+$, 139.06.

The reaction of 4-phenoxybenzonitrile (1e) with 4-methylbenzoic acid (2a) produced phenyl 4-methylbenzoate (3a)[12] and 4-hydroxybenzonitrile (4e)

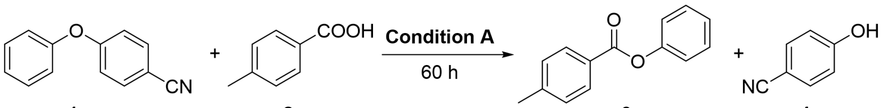

1e 2a 3a 4e

According to the condition A, **3a** was obtained as a white solid (77.5 mg) in 73% yield, **4e** was obtained as a white solid (42.9 mg) in 72% yield. **4e**, ^{1}H NMR (400 MHz, $CDCl_3$) δ 7.79-7.42 (m, 2H), 7.09-6.82 (m, 2H), 6.42 (s, 1H). ^{13}C NMR (100 MHz, $CDCl_3$) δ 160.0, 134.3 (2C), 119.2, 116.4 (2C), 103.2. MS: m/z: $[M]^+$, 119.04.

The reaction of 1-(4-phenoxyphenyl)ethan-1-one (1f) with 4-methylbenzoic acid (2a) produced phenyl 4-methylbenzoate (3a)[12] and 1-(4-hydroxyphenyl)ethan-1-one (4f)

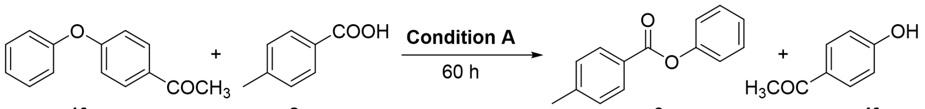

1f 2a 3a 4f

According to the condition A, **3a** was obtained as a white solid (68.9 mg) in 65% yield, **4f** was obtained as a white solid (42.2 mg) in 62% yield. **4f**, ^{1}H NMR (400 MHz, $CDCl_3$) δ 7.92 (d, J = 8.6 Hz, 2H), 7.50 (s, 1H), 6.95 (d, J = 8.6 Hz, 2H), 2.59 (s, 3H). ^{13}C NMR (100 MHz, $CDCl_3$) δ 198.3, 161.0, 131.2 (2C), 129.7, 115.5 (2C), 26.3. MS: m/z: $[M]^+$, 136.06.

The reaction of 1-bromo-4-phenoxybenzene (1g) with 4-methylbenzoic acid (2a) produced 4-bromophenyl 4-methylbenzoate (3as),[17] phenyl 4-methylbenzoate (3a),[12] phenol (4a) and 4-bromophenol (4g)

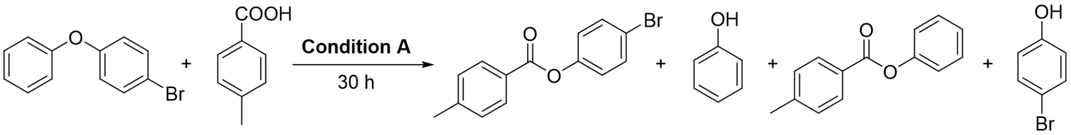

1g 2a 3as 4a 3a 4g

According to the condition A, **3as** and **3a** were obtained as a mixture (116.6 mg). **4a** and **4d** were also obtained as a mixture (43.5

mg). The yields of them were determined according to integration of the peaks assigned to colored H in the mixed ^{1}HNMR as **3as** (64% yield), **3a** (22% yield), **4a** (54% yield), **4g** (21% yield).

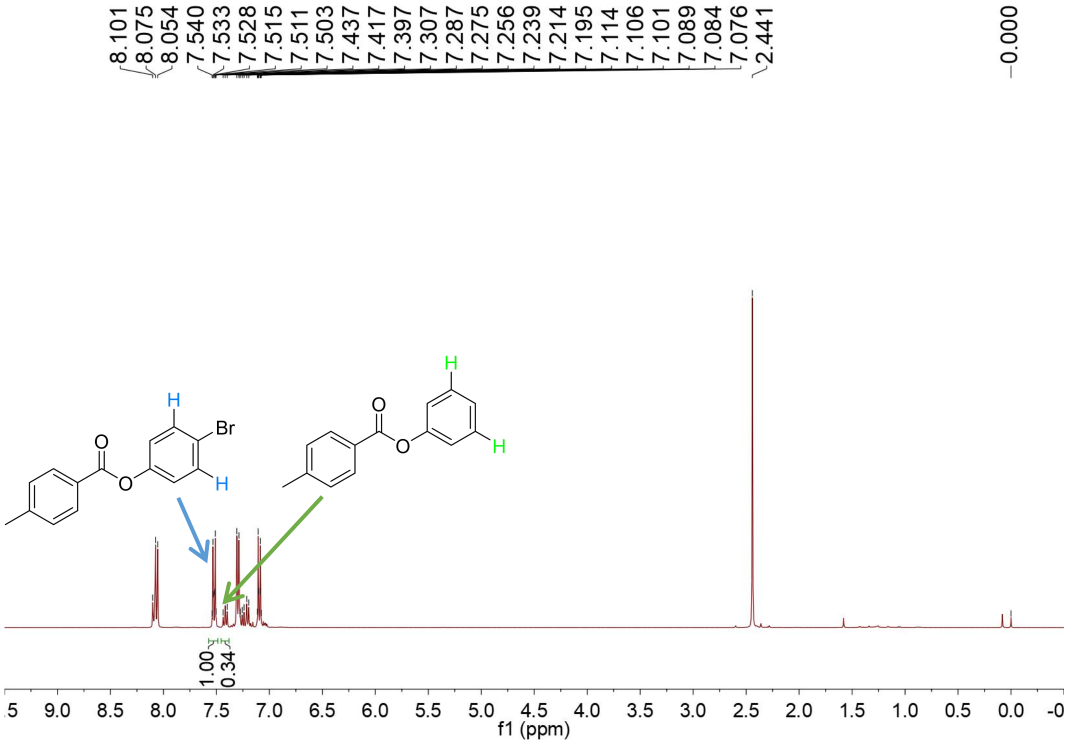

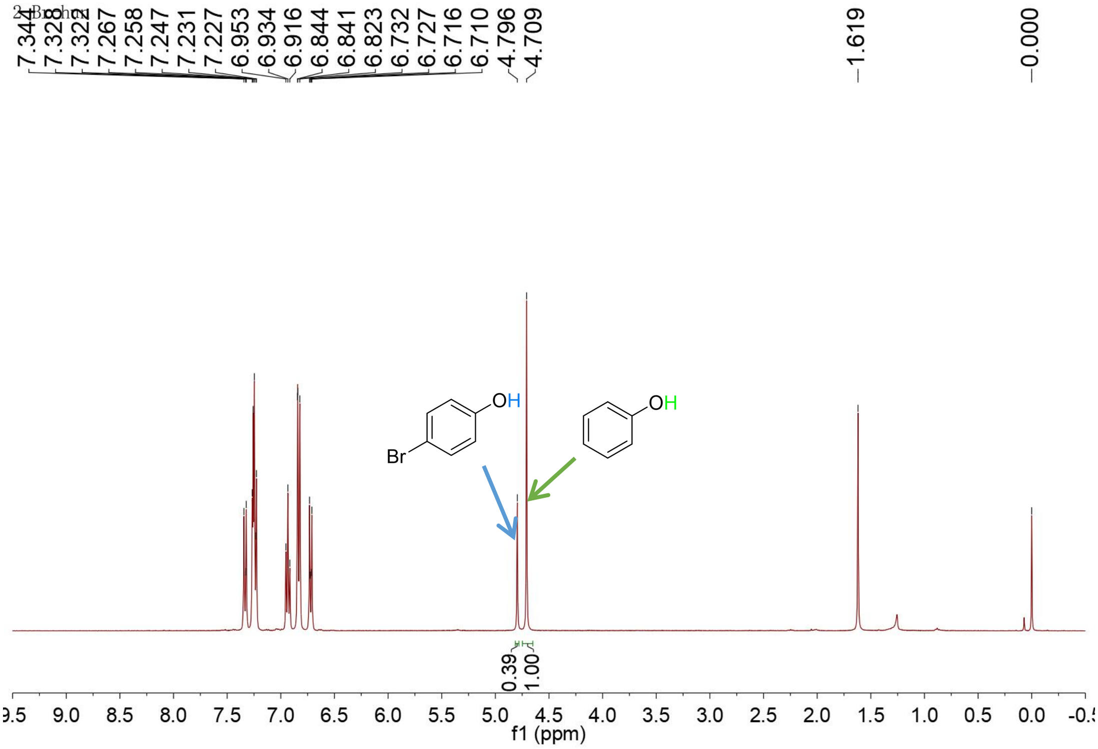

The reaction of 4-phenoxy-1,1'-biphenyl (1h) with 4-methylbenzoic acid (2a) produced [1,1'-biphenyl]-4-yl 4-methylbenzoate (3at)[17] and phenol (4a)

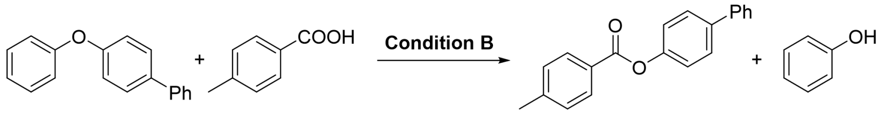

1h **2a** **3at** **4a**

According to the condition B, **3at** was obtained as a white solid (121.1 mg) in 84% yield, **4a** was obtained as a colorless solid (39.4

mg) in 84% yield. **3at**, ^{1}H NMR (400 MHz, $CDCl_3$) δ 8.12 (d, *J* = 8.0 Hz, 2H), 7.69-7.55 (m, 4H), 7.50-7.40 (m, 2H), 7.41-7.26 (m, 5H), 2.47 (s, 3H). ^{13}C NMR (100 MHz, $CDCl_3$) δ 165.2, 150.4, 144.4, 140.4, 138.9, 130.2 (2C), 129.3 (2C), 128.7 (2C), 128.1 (2C), 127.3, 127.1 (2C), 126.7, 122.0 (2C), 21.7. MS: m/z: $[M]^+$, 288.08.

The reaction of 1-methoxy-3-phenoxybenzene (1i) with 4-methylbenzoic acid (2a) produced p-tolyl 4-methylbenzoate (3au)[17] and phenol (4a)

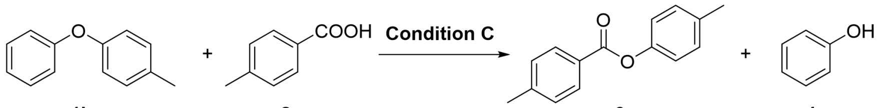

1i 2a 3au 4a

According to the condition C, **3au** was obtained as a colorless solid (28.9 mg) in 64% yield, **4a** was obtained as a colorless solid (10.2 mg) in 54% yield. **3au**, ^{1}H NMR (400 MHz, $CDCl_3$) δ 8.21-7.99 (m, 2H), 7.37-7.27 (m, 2H), 7.27-7.16 (m, 2H), 7.13-7.05 (m, 2H), 2.45 (s, 3H), 2.37 (s, 3H). ^{13}C NMR (100 MHz, $CDCl_3$) δ 165.4, 148.7, 144.2, 135.3, 130.2 (2C), 129.9 (2C), 129.2 (2C), 126.9, 121.4 (2C), 21.7, 20.9. MS: m/z: $[M]^+$, 226.09.

The reaction of 1-methoxy-3-phenoxybenzene (1j) with 4-methylbenzoic acid (2a) produced 4-methoxyphenyl 4-methylbenzoate (3av)[17] and phenol (4a)

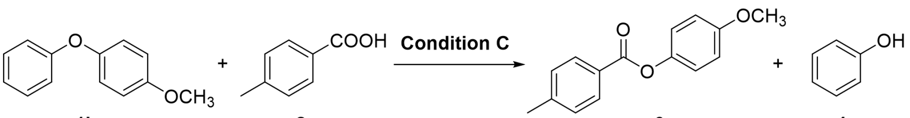

1j 2a 3av 4a

According to the condition C, **3av** was obtained as a colorless solid (11.6 mg) in 24% yield, **4a** was obtained as a colorless solid (1.9 mg) in 10% yield. **3av**, ^{1}H NMR (400 MHz, $CDCl_3$) δ 8.08 (d, *J* = 8.0 Hz, 2H), 7.29 (d, *J* = 8.0 Hz, 2H), 7.12 (d, *J* = 9.2 Hz, 2H), 6.93 (d, *J* = 9.2 Hz, 2H), 3.81 (s, 3H), 2.44 (s, 3H). ^{13}C NMR (100 MHz, $CDCl_3$) δ 165.6, 157.2, 144.5, 144.3, 130.1 (2C), 129.2 (2C), 126.9, 122.5 (2C), 114.5 (2C), 55.6, 21.8. MS: m/z: $[M]^+$, 242.08.

The reaction of 1-methyl-2-phenoxybenzene (1k) with 4-methylbenzoic acid (2a) produced o-tolyl 4-methylbenzoate (3aw)[18] and phenol (4a)

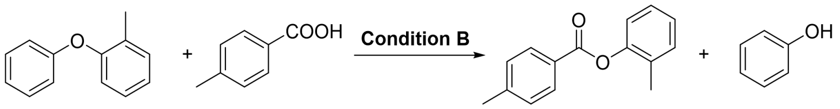

1k 2a 3aw 4a

According to the condition B, **3aw** was obtained as a colorless solid (90.5 mg) in 80% yield, **4a** was obtained as a colorless solid (33.8 mg) in 72% yield. **3aw**, ^{1}H NMR (400 MHz, $CDCl_3$) δ 8.11 (d, *J* = 8.4 Hz, 2H), 7.31 (d, *J* = 8.0 Hz, 2H), 7.28-7.20 (m, 2H), 7.21-7.08 (m, 2H), 2.44 (s, 3H), 2.23 (s, 3H). ^{13}C NMR (100 MHz, $CDCl_3$) 164.9, 149.5, 144.4, 131.1, 130.3, 130.2 (2C), 129.3 (2C), 126.9, 126.6, 125.9, 122.0, 21.7, 16.2. MS: m/z: $[M]^+$, 226.09.

The reaction of 1,3-dimethyl-5-phenoxybenzene (1l) with 4-methylbenzoic acid (2a) produced 3,5-dimethylphenyl 4-methylbenzoate (3ax)[17] and phenol (4a)

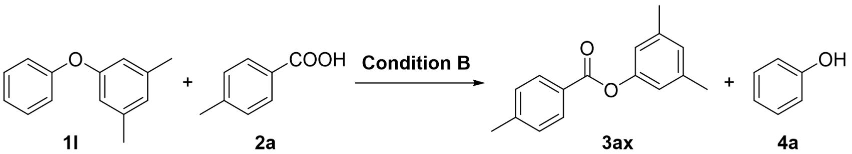

1l 2a 3ax 4a

According to the condition B, **3ax** was obtained as a white solid (85.3 mg) in 71% yield, **4a** was obtained as a colorless oil (33.3 mg) in 71% yield. **3ax**, ^{1}H NMR (400 MHz, $CDCl_3$) δ 8.07 (d, *J* = 8.4 Hz, 2H), 7.28 (d, *J* = 8.0 Hz, 2H), 6.89 (s, 1H), 6.82 (s, 2H), 2.43 (s, 3H), 2.33 (s, 6H). ^{13}C NMR (100 MHz, $CDCl_3$) δ 165.4, 150.8, 144., 139.3 (2C), 130.1 (2C), 129.2 (2C), 127.5, 126.9, 119.3 (2C), 21.7, 21.2 (2C). MS: m/z: $[M]^+$, 240.13.

The reaction of 1-methoxy-3-phenoxybenzene (1m) with 4-methylbenzoic acid (2a) produced 3-methoxyphenyl

4-methylbenzoate (3ay)[17] and phenol (4a)

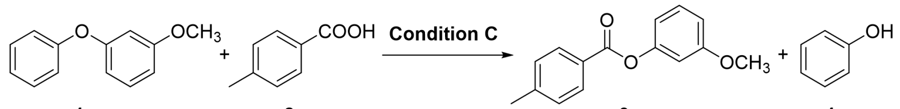

1m 2a 3ay 4a

According to the condition C, **3ay** was obtained as a colorless solid (32.9 mg) in 68% yield, **4a** was obtained as a colorless solid (11.5 mg) in 61% yield. **3ay**, ^{1}H NMR (400 MHz, $CDCl_3$) δ 8.19-8.00 (m, 2H), 7.51-7.09 (m, 3H), 7.07-6.26 (m, 3H), 3.81 (s, 3H), 2.45 (s, 3H). ^{13}C NMR (100 MHz, $CDCl_3$) δ 165.1, 160.5, 152.0, 144.4, 130.2 (2C), 129.8, 129.3 (2C), 126.7, 114.0, 111.8, 107.7, 55.4, 21.7. MS: m/z: $[M]^+$, 242.08.

The reaction of 3,3'-oxybis(methylbenzene) (1n) with 4-methylbenzoic acid (2a) produced m-tolyl 4-methylbenzoate (3az)[19] and phenol (4h)

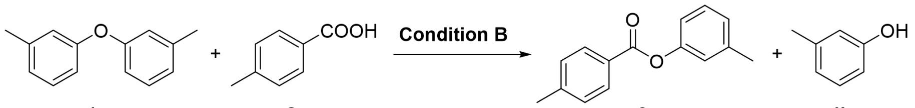

1n 2a 3az 4h

According to the condition A, **3az** was obtained as a white solid (78.0 mg) in 69% yield, **4h** was obtained as a colorless oil (34.1 mg) in 63% yield. **3az**, ^{1}H NMR (400 MHz, $CDCl_3$) δ 8.09 (d, J = 8.4 Hz, 2H), 7.35-7.27 (m, 3H), 7.08 (d, J = 7.6 Hz, 1H), 7.06-6.94 (m, 2H), 2.45 (s, 3H), 2.39 (s, 3H). ^{13}C NMR (100 MHz, $CDCl_3$) δ 165.3, 150.9, 144.3, 139.6, 130.1 (2C), 129.2 (2C), 129.1, 126.8, 126.6, 122.3, 118.6, 21.7, 21.3. MS: m/z: $[M]^+$, 226.03. **4h**, ^{1}H NMR (400 MHz, $CDCl_3$) δ 7.15-7.05 (m, 1H), 6.75 (d, J = 7.2 Hz, 1H), 6.69-6.58 (m, 2H), 5.19 (br s, 1H), 2.29 (s, 3H). ^{13}C NMR (100 MHz, $CDCl_3$) δ 155.3, 139.8, 129.4, 121.6, 116.0, 112.2, 21.3. MS: m/z: $[M]^+$, 108.07.

The reaction of 3,3'-oxybis(methoxybenzene) (1o) with 4-methylbenzoic acid (2a) produced 3-methoxyphenyl 4-methylbenzoate (3ay)[17] and 3-methoxyphenol (4i)

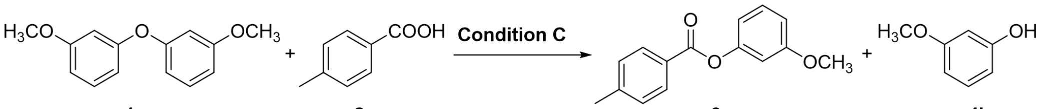

1o 2a 3ay 4i

According to the condition C, **3ay** was obtained as a colorless solid (25.2 mg) in 52% yield, **4i** was obtained as a pink oil (12.4 mg) in 50% yield. **4i**, ^{1}H NMR (400 MHz, $CDCl_3$) δ 7.20-7.00 (m, 1H), 6.59-6.29 (m, 3H), 3.76 (s, 3H). ^{13}C NMR (100 MHz, $CDCl_3$) δ 160.7, 156.6, 130.2, 107.9, 106.4, 101.5, 55.3. MS: m/z: $[M]^+$, 124.05.

The reaction of 4,4'-oxybis(bromobenzene) (1p) with 4-methylbenzoic acid (2a) produced 4-bromophenyl 4-methylbenzoate (3as)[17] and 4-bromophenol (4g)

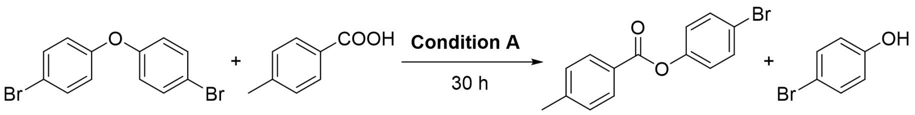

1p 2a 3as 4g

According to the condition A, **3as** was obtained as a white solid (119.4 mg) in 82% yield, **4g** was obtained as a white solid (69.2 mg) in 80% yield. **3as**, ^{1}H NMR (400 MHz, $CDCl_3$) δ 8.07 (d, J = 8.2 Hz, 2H), 7.61-7.47 (m, 2H), 7.31 (d, J = 8.0 Hz, 2H), 7.17-7.05 (m, 2H), 2.46 (s, 3H). ^{13}C NMR (100 MHz, $CDCl_3$) δ 164.9, 150.0, 144.7, 132.5 (2C), 130.2 (2C), 129.3 (2C), 126.4, 123.6 (2C), 118.9, 21.8. MS: m/z: $[M]^+$, 289.97. **4g**, ^{1}H NMR (400 MHz, $CDCl_3$) δ 7.33 (d, J = 8.4 Hz, 2H), 6.72 (d, J = 8.8 Hz, 2H), 4.85 (br s, 1H). ^{13}C NMR (100 MHz, $CDCl_3$) δ 154.3, 132.5 (2C), 117.2 (2C), 112.9. MS: m/z: $[M]^+$, 171.96.

The reaction of 4,4'-oxybis(chlorobenzene) (1q) with 4-methylbenzoic acid (2a) produced 4-chlorophenyl 4-methylbenzoate (3aA)[20] and 4-chlorophenol (4j)

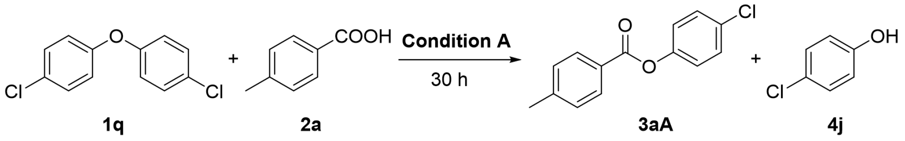

According to the condition A, **3aA** was obtained as a white solid (108.4 mg) in 88% yield, **4j** was obtained as a colorless solid (54.5 mg) in 85% yield. **3aA**, ^1H NMR (400 MHz, $CDCl_3$) δ 8.07 (d, J = 8.0 Hz, 2H), 7.38 (d, J = 8.8 Hz, 2H), 7.31 (d, J = 8.0 Hz, 2H), 7.16 (d, J = 8.4 Hz, 2H), 2.45 (s, 3H). ^{13}C NMR (100 MHz, $CDCl_3$) δ 165.0, 149.4, 144.7, 131.1, 130.2 (2C), 129.5 (2C), 129.3 (2C), 126.4, 123.1 (2C), 21.8. MS: m/z: $[M]^+$, 246.03. **4j**, ^1H NMR (400 MHz, $CDCl_3$) δ 7.23-7.15 (m, 2H), 6.82-6.73 (m, 2H), 5.04 (br s, 1H). ^{13}C NMR (100 MHz, $CDCl_3$) δ 154.0, 129.5 (2C), 125.6, 116.6 (2C). MS: m/z: $[M]^+$, 128.01.

The reaction of 1-chloro-3-(4-chlorophenoxy)benzene (1r) with 4-methylbenzoic acid (2a) produced 4-chlorophenyl 4-methylbenzoate (3aA)[20] and 3-chlorophenol (4k)

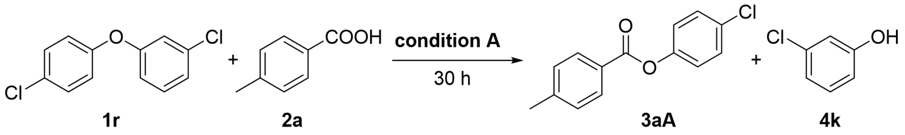

According to the condition A, **3aA** was obtained as a white solid (111.0 mg) in 90% yield, **4k** was obtained as a brown oil (55.2 mg) in 86% yield. **4k**, ^1H NMR (400 MHz, $CDCl_3$) δ 7.20-7.05 (m, 1H), 6.98-6.89 (m, 1H), 6.88-6.78 (m, 1H), 6.76-6.66 (m, 1H), 6.21 (br s, 1H). ^{13}C NMR (100 MHz, $CDCl_3$) δ 155.8, 134.8, 130.5, 121.21, 115.8, 113.8. MS: m/z: $[M]^+$, 128.00.

The reaction of methyl 4-(o-tolyloxy)benzoate (1s) with 4-methylbenzoic acid (2a) produced o-tolyl 4-methylbenzoate (3aw)[18] and methyl 4-hydroxybenzoate (4b)

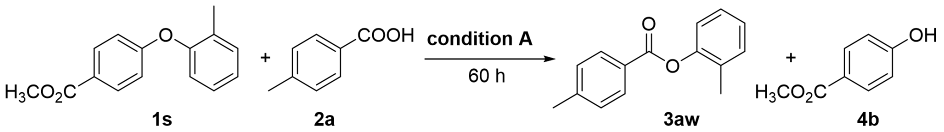

According to the condition A, **3aw** was obtained as a white solid (92.7 mg) in 82% yield, **4b** was obtained as a white solid (62.4 mg) in 82% yield.

The reaction of 4-(m-tolyloxy)benzonitrile (1t) with 4-methylbenzoic acid (2a) produced (3-((4-methylbenzoyl)oxy)phenyl)methylium (3az)[19] and 4-hydroxybenzonitrile (4e)

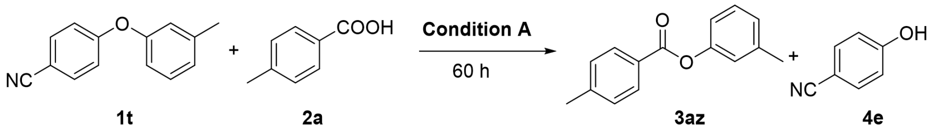

According to the condition A, **3az** was obtained as a white solid (85.9 mg) in 76% yield, **4i** was obtained as a white solid (44.0 mg) in 74% yield.

The reaction of 1-methoxy-2-(4-(trifluoromethyl)phenoxy)benzene (1u) with 4-methylbenzoic acid (2a) produced 2-methoxyphenyl 4-methylbenzoate (3aB)[21] and 4-(trifluoromethyl)phenol (4c)

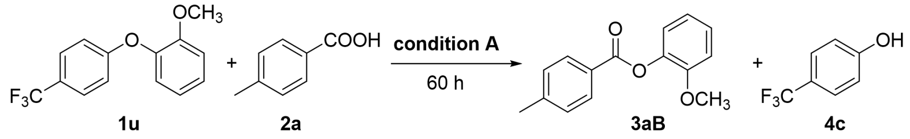

According to the condition A, **3aB** was obtained as a white solid (87.2 mg) in 72% yield, **4c** was obtained as a white solid (56.7 mg) in 70% yield. **3aB**, ^1H NMR (400 MHz, $CDCl_3$) δ 8.11 (d, J = 8.0 Hz, 2H), 7.30 (d, J = 8.0 Hz, 2H), 7.27-7.20 (m, 1H), 7.15 (dd, J = 7.8, 1.6 Hz, 1H), 7.06-6.93 (m, 2H), 3.81 (s, 3H), 2.45 (s, 3H). ^{13}C NMR (100 MHz, $CDCl_3$) δ 164.8, 151.4, 144.2, 140.0, 130.3 (2C), 129.2 (2C), 126.9, 126.7, 123.0, 120.8, 112.5, 55.9, 21.8. MS: m/z: $[M]^+$, 242.08.

C-O bond Cleavage of an Aryl Ether by a One-pot, Two-step Reaction

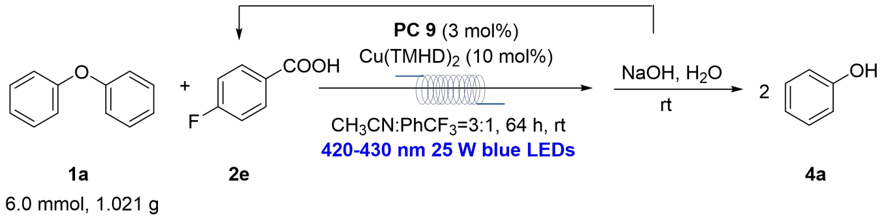

Reaction Conditions: Under argon atmosphere, a solution of compound **1a** (6.0 mmol), compound **2e** (7.2 mmol), **PC 9** (3 mol%, 76.8 mg), and Cu(TMHD)$_2$ (10 mol%, 258 mg) in anhydrous CH_3CN (60 mL) and $PhCF_3$ (20 mL) was introduced into a round bottom flask. As shown in Supplementary Figure 1 (c), an Asian pump was connected with the solution and the flow tube (FEP PTFE tubing O.D. 1/16", I.D. 0.03", 1000 cm). The flow apparatus was set up with flow rate = 21.0 μL/min. Then the tube was irradiated under a 420-430 nm 25 W blue LEDs for 64 h. After the reaction, the solvent was partially evaporated. Then NaOH (12.0 mmol, 2.0 equiv) in 20 mL H_2O was added and stirred for another 24 h at room temperature. The reaction mixture was then acidified with 2.1 equiv concentrated HCl, extracted with Et_2O (15 mL × 3). The organic layer was washed with brine, dried over anhydrous Na_2SO_4. After purification by flash chromatography on silica gel (petroleum ether: EtOAc = 20:1-3:1), **4a** (903 mg) was obtained in 80% yield and **2e** (887 mg) was recovered in 88% recovery rate, respectively.

The Application of C-O Bond Cleavage to the Model of 4-O-5 Lignin Linkages

Reaction Conditions: Under argon atmosphere, a solution of compound **1v** (0.20 mmol), compound **2e** (0.24 mmol, 40.05 mg), **PC 9** (0.006 mmol, 3.0 mol%), and Cu(TMHD)$_2$ (0.02 mmol, 10 mol%) in anhydrous CH_3CN (2.0 mL) and $PhCF_3$ (1 mL) was added into a sealed vial. Then the solution was transformed into a Stop-Flow micro tube (total volume 3.0 mL), which was made of PTFE tubing (O.D. 1/16", I.D. 0.03", 1000 cm), via a syringe. The valves were closed, and the Static-Flow micro tubing reactor (Supplementary Figure 1 (b)) was irradiated with 420-430 nm 25 W blue LEDs at ambient temperature (23 °C) for 100 h. After the reaction, the solvent was removed in vacuo and the residue was purified by column chromatography (petroleum ether/EtOAc = 200/1 - 5/1) to afford compounds **3et** (81%, 119.3 mg) and **4a** (71%, 39.9 mg). To a 50 mL round bottom flask with a stirring bar, **3et** (119.3 mg, 0.49 mmol), NaOH (0.97 mmol, 2.0 equiv) in 2.0 mL CH_3CN and 2.0 mL H_2O was added and stirred for another 24 h at room temperature. The reaction mixture was then acidified with 2.1 equiv concentrated HCl, extracted with Et_2O (10 mL x 3). The organic layer was washed with brine, dried over anhydrous Na_2SO_4. After purification by flash chromatography on silica gel (petroleum ether: EtOAc = 50:1-10:1), **4l** (55.9 mg) was obtained in 75% yield in two steps.

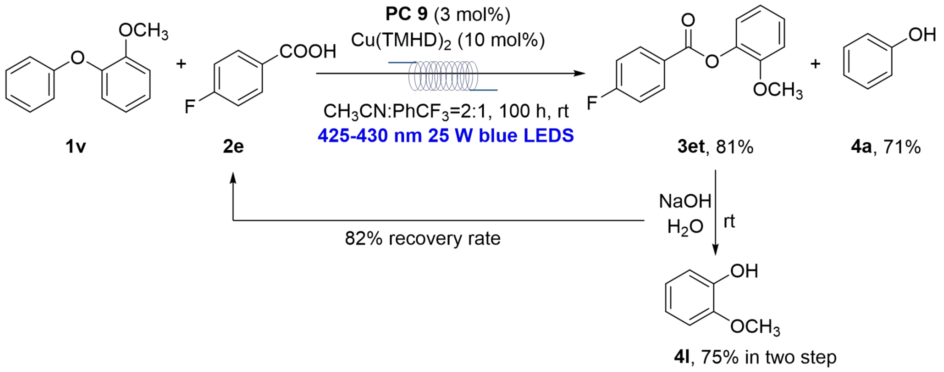

Due to the difficult purification for the isolated yields of **4a** and **4l**, after the acidolysis, the reaction was worked-up to afford **3et** and **4a** in 81% and 71% yields, respectively. The yields of **3et, 4a**, and **4l**, and the recovery rate of **2e** were determined by two parallel reactions.

Under argon atmosphere, a solution of compound **1v** (0.20 mmol), compound **2e** (0.24 mmol, 40.05 mg), **PC 9** (0.006 mmol, 3.0 mol%), and Cu(TMHD)$_2$ (0.02 mmol, 10 mol%) in anhydrous CH_3CN (2.0 mL) and $PhCF_3$ (1 mL) was added into a sealed vial. Then the solution was pumped into a Stop-Flow micro tubing reactor (total volume 3.0 mL), which was made of PTFE tubing (O.D. 1/16", I.D. 0.03", 1000 cm), via a syringe. The valves were closed, and the Stop-Flow micro tubing reactor (Supplementary Figure 1 (b)) was

irradiated with 420-430 nm 25 W blue LEDs at ambient temperature (23 °C) for 100 h. Then NaOH (2.0 equiv) in 2.0 mL H_2O was added and stirred for another 24 h at room temperature. The reaction mixture was then acidified with 2.1 equiv concentrated HCl, extracted with Et_2O (5 mL × 3). The organic layer was washed with brine, dried over anhydrous Na_2SO_4. After purification by flash chromatography on silica gel (petroleum ether: EtOAc = 20:1-3:1), **2e** was obtained in 82% (27.5 mg) recovery rate.

X-ray Crystallography Studies of PC 9

Single crystals suitable for X-ray diffraction were obtained by slow evaporation of a saturated solution of **PC 9** in chloroform and cyclohexane in a loosely capped vial. H atoms and BF_4^- have been omitted for clarity.

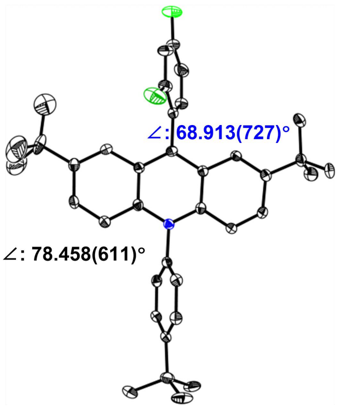

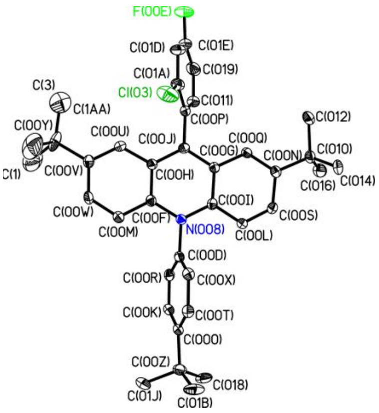

Summary of Data CCDC 2004336

Compound Name: **PC9**

Formula: $C_{37}H_{40}BClF_5N$

Unit Cell Parameters: a 10.550(2) b 17.612(4) c 17.707(4) P-1

Mechanistic Investigation

Quantum Yield Measurements:

Determination the Light Intensity at 402 nm:

According to the procedure of Yoon,[22] the photon flux of the spectrophotometer was determined by standard ferrioxalate actinometry.[23,24] A 0.15 M solution of ferrioxalate was prepared by dissolving potassium ferrioxalate hydrate (1.47 g) in H_2SO_4 (20 mL of a 0.05 M solution). A buffered solution of 1,10-phenanthroline was prepared by dissolving phenanthroline (25 mg) and sodium acetate (5.63 g) in H_2SO_4 (25 mL of a 0.5 M solution). Both solutions were stored in the dark. To determine the photon flux of the spectrophotometer, the ferrioxalate solution (2.0 mL) was placed in a cuvette and irradiated for 90 seconds at λ=402 nm. After in the dark for 1 hour to allow the ferrous ions to completely coordinate to the phenanthroline. The absorbance of the solution was measured at 510 nm. A non-irradiated sample was also prepared and the absorbance at 510 nm was measured. Conversion was calculated using eq S1.

$$\text{mol Fe}^{2+} = \frac{V \cdot \Delta A}{l \cdot \varepsilon} = \frac{0.00235\,\text{L} \times 1.774}{1.000\,\text{cm} \times 11{,}100\,\text{L} \cdot \text{mol}^{-1}\text{cm}^{-1}} = 3.76 \times 10^{-7}\,\text{mol} \quad \text{(eq S1)}$$

Where V is the total volume (0.00235 L) of the solution after addition of phenanthroline, ΔA is the difference in absorbance at 510 nm between the irradiated and non-irradiated solutions, l is the path length (1.000 cm), and ε is the molar absorptivity at 510 nm (11,100 L $mol^{-1}cm^{-1}$). The photon flux can be calculated using eq S2.

$$\text{Photon flux} = \frac{\text{mol Fe}^{2+}}{\phi \cdot t \cdot f} = \frac{3.76 \times 10^{-7}}{1.07 \times 90\,\text{s} \times 0.99356} = 3.93 \times 10^{-9}\,\text{einstein s}^{-1} \quad \text{(eq S2)}$$

Where Φ is the quantum yield for the ferrioxalate actinometer (1.07 for a 0.15 M solution at λ = 402 nm),[20] t is the time (90.0 s), and f is the fraction of light absorbed at λ = 402 nm.

Determination of Fraction of Light Absorbed at 402 nm for the Ferrioxalate Solution:

The fraction of light absorbed (f) by this solution was calculated using eq S3, where A is the measured absorbance of the above ferrioxalate solution at 402 nm which was measured to be 2.1909 (Supplementary Figure 2), indicating the fraction of light absorbed (f) is 0.99356. Finally, the photon flux was calculated to be 3.93×10^{-9} einstein s^{-1}.

$$f = 1 - 10^{-A} = 0.99356 \quad \text{(eq S3)}$$

Quantum Yield Calculation:

A cuvette was charged with **1a** (0.2 mmol), **2a** (0.24 mmol), **I** (0.006 mmol), Cu(TMHD)$_2$ (0.02 mmol), and 2.0 mL degassed CH_3CN in glovebox under Ar atmosphere. The cuvette was then capped with a PTFE stopper. The sample was irradiated (λ = 402 nm, slit width = 10.0 nm) for 43,200 s (12 h). After irradiation, the yield of product formed was determined by ^1H NMR to be 17% using 1,1,2,2-tetrachloroethane as an internal standard. The quantum yield was determined as 0.20 as shown in eq S4.

$$\phi = \frac{\text{mol prod}}{\text{flux} \cdot t \cdot f} = \frac{0.17 \times 0.2 \times 10^{-3}\,\text{mol}}{3.93 \times 10^{-9} \times 43{,}200 \times 0.99228} = 0.20 \quad \text{(eq S4)}$$

Absorbance of Catalyst:

The absorbance of **PC 9** in CH_3CN was measured at the reaction concentration of 2×10^{-3} M (Supplementary Figure 3). The absorbance at 402 nm is 2.1125, indicating the fraction of light absorbed (f) is 0.99228.

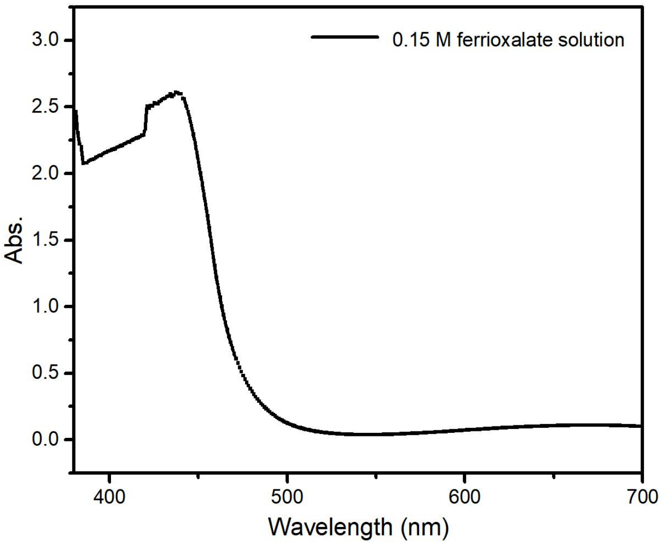

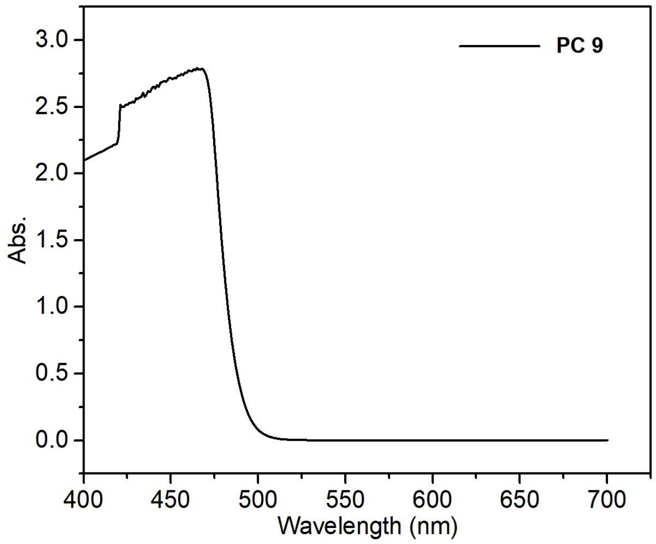

Supplementary Figure 2 UV-vis spectrum of 0.15 M ferrioxalate solution. The data represents the absorption intensity of ferrioxalate solution at each wavelengths. Source data are provided in a Source Data file.

Supplementary Figure 3 UV-vis absorption of 2 × 10^{-3} M **PC 9**. The data represents represents the absorption intensity of **PC 9** at each wavelengths.Source data are provided in a Source Data file.

UV-Vis Absorption of 1a, 2a, PC 9 and the Reaction Mixture

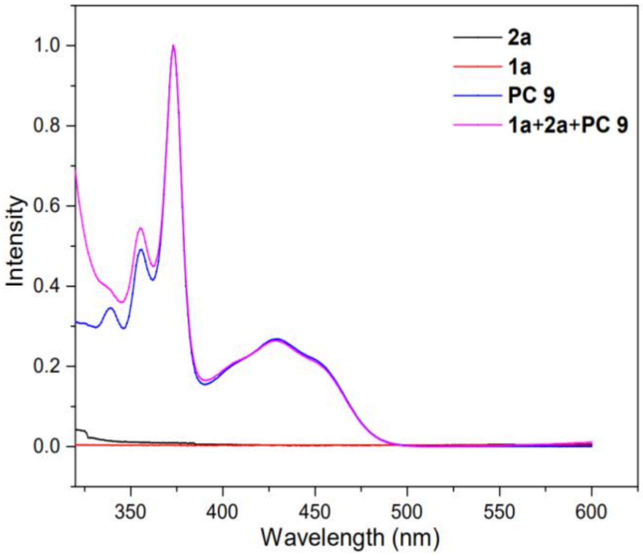

Supplementary Figure 4 UV-vis absorption of **1a**, **2a**, **PC 9** and the reaction mixture. Source data are provided in a Source Data file.

Emission Quenching Experiments:

Emission intensities were recorded using a spectrofluorimeter. **PC 9** solution was excited at 402 nm and the emission intensity at 508 nm was observed. CH_3CN was selected as solvent, and the solvent was degassed with a stream of argon for 30 min. In a typical experiment, the sample was degassed with a stream of argon for 15 minutes, then the emission spectrum of the sample was collected. First, the emission spectrum of a 5×10^{-5} M solution of **PC 9** in CH_3CN was collected. Then, appropriate amount of quencher was added to the measured solution and the emission spectrum of the sample was collected.

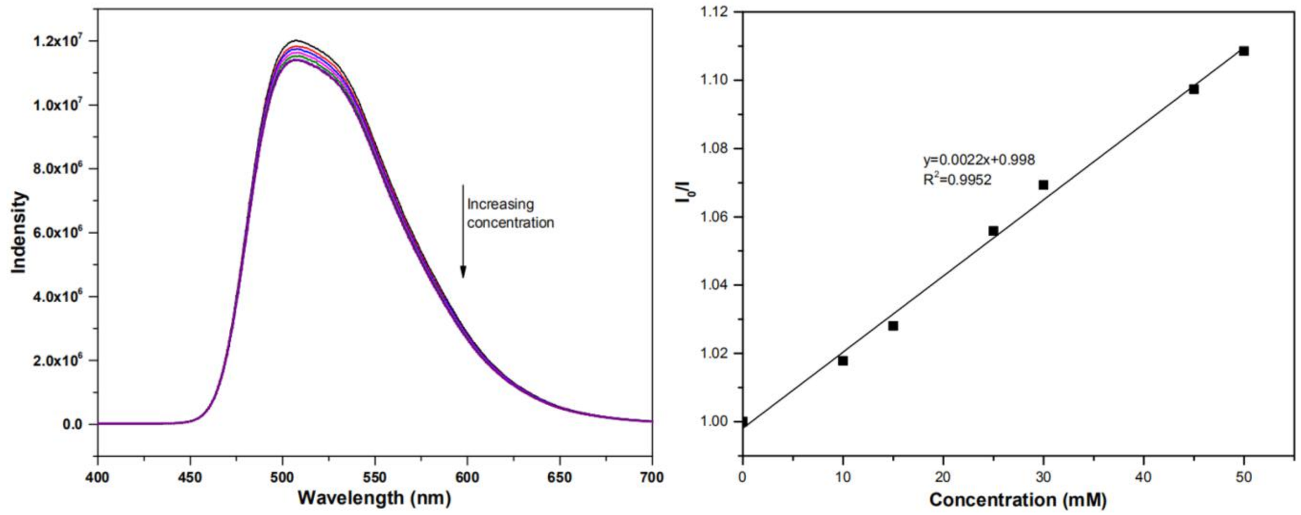

Supplementary Figure 5 PC 9 emission quenching by 4-methylbenzoic acid (**2a**). The data represents that as the concentration of **2a** increases, the fluorescence emission intensity of **PC 9** gradually decreases. Source data are provided in a Source Data file.

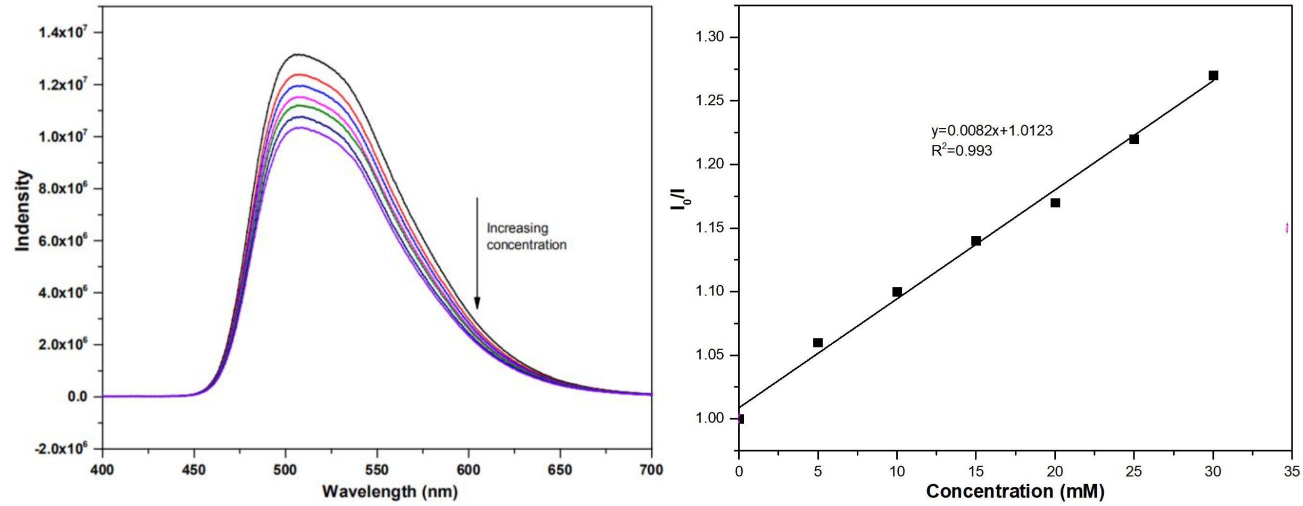

Supplementary Figure 6 PC 9 emission quenching by diphenyl ether (**1a**). The data represents that as the concentration of **1a** increases, the fluorescence emission intensity of **PC 9** gradually decreases. Source data are provided in a Source Data file.

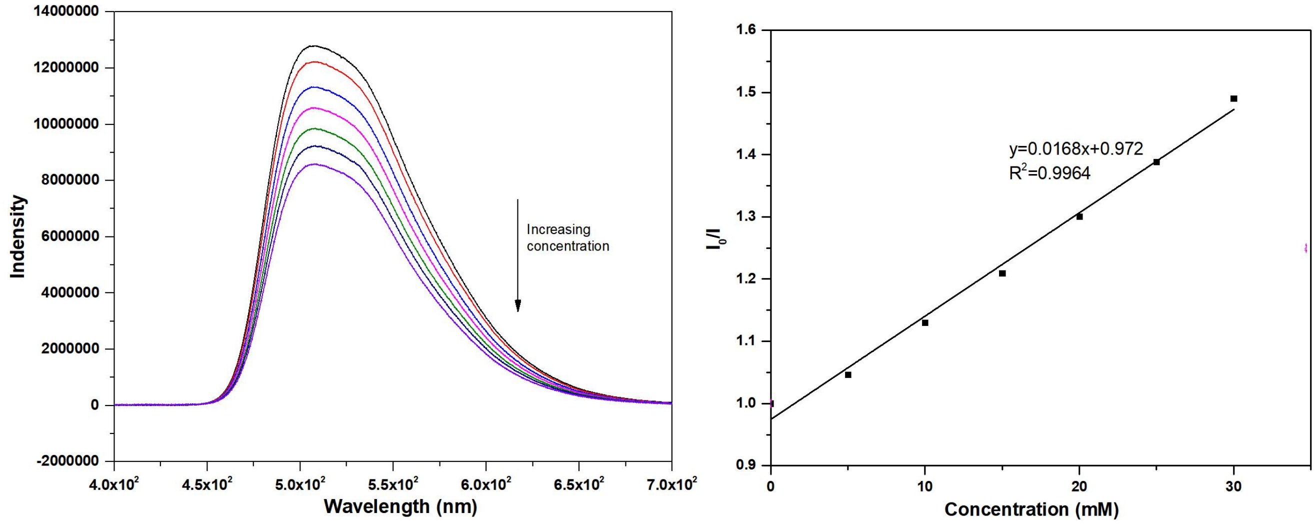

Supplementary Figure 7 PC 9 emission quenching by tetrabutylammonium 4-methylbenzoate (4-Me-$PhCO_2N$(*n*-Bu)$_4$). The data represents that as the concentration of 4-Me-$PhCO_2N$(*n*-Bu)$_4$ increases, the fluorescence emission intensity of **PC 9** decreases. Source data are provided in a Source Data file.

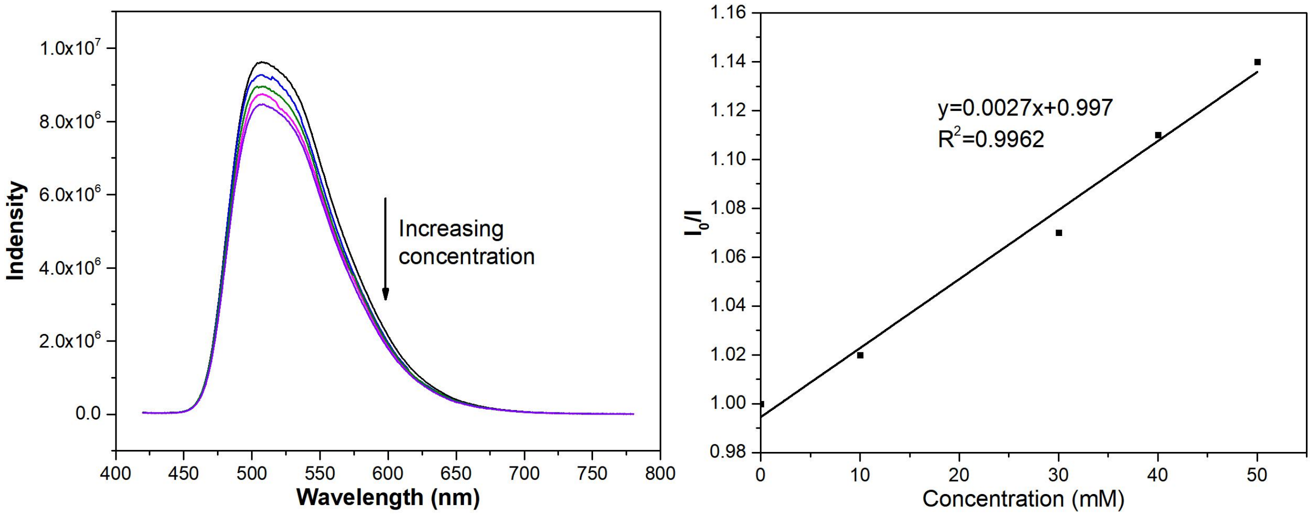

Supplementary Figure 8 PC 9 emission quenching by Cu(TMHD)$_2$. The data represents that as the concentration of Cu(TMHD)$_2$ increases, the fluorescence emission intensity of **PC 9** decreases. Source data are provided in a Source Data file.

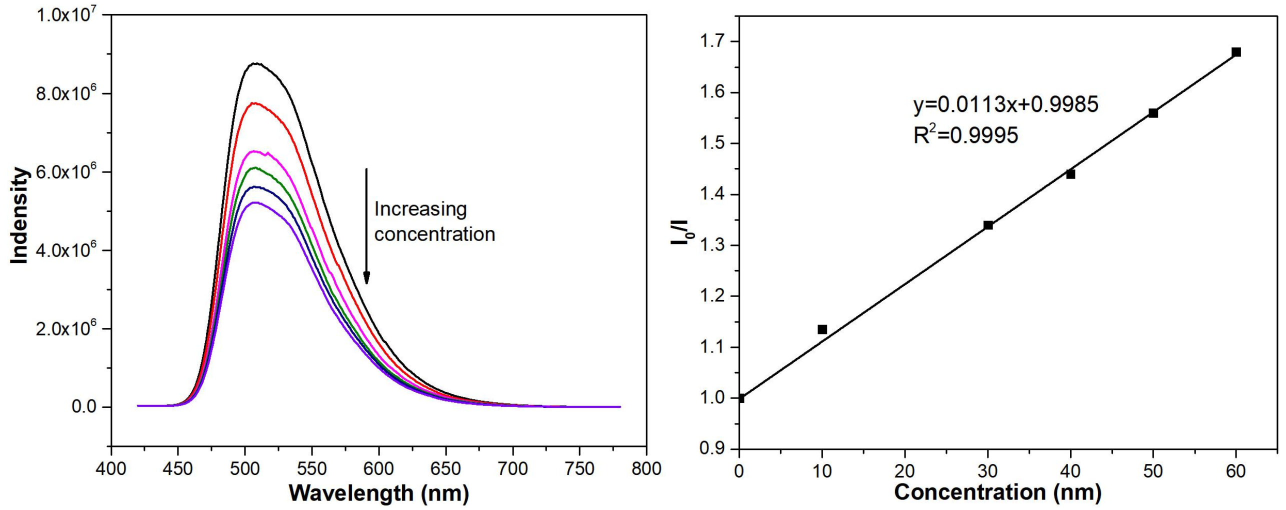

Supplementary Figure 9 PC 9 emission quenching by 4-Me-PhCO$_2$N(n-Bu)$_4$ and Cu(TMHD)$_2$. The data represents that as the concentration of 4-Me-PhCO$_2$N(n-Bu)$_4$ and Cu(TMHD)$_2$ increases, the fluorescence emission intensity of **PC 9** decreases. Source data are provided in a Source Data file.

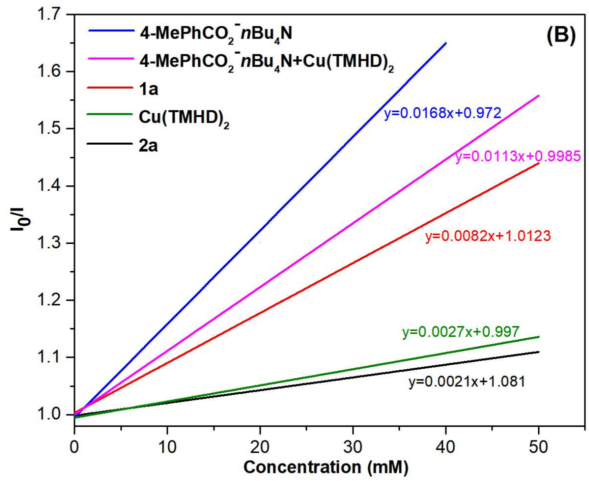

Supplementary Figure 10 quenching curve. **PC 9** emission quenching curve by tetrabutylammonium 4-methylbenzoate, **1a**, **2a,** 4-Me-PhCO$_2$N(n-Bu)$_4$ and Cu(TMHD)$_2$.

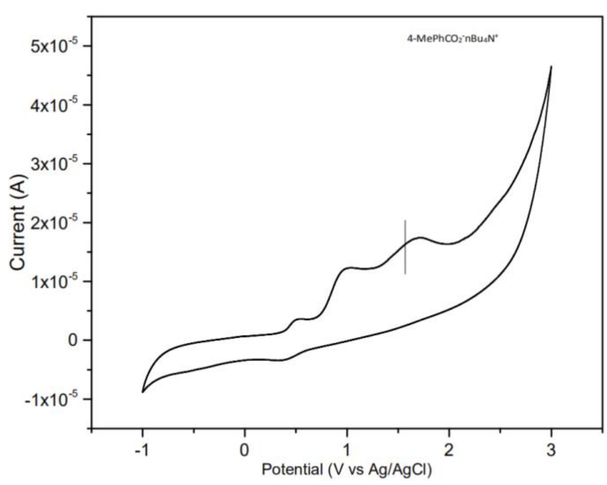

Supplementary Figure 11 Redox potential of 4-MePhCO$_2^-$$nBu_4N^+$ ($E^{\text{4-MePhCO2}^\bullet/\text{4-MePhCO2}^-}$ = +1.45 V vs SCE). It was converted from internal standard Fc$^+$/Fc^0. Source data are provided in a Source Data file.

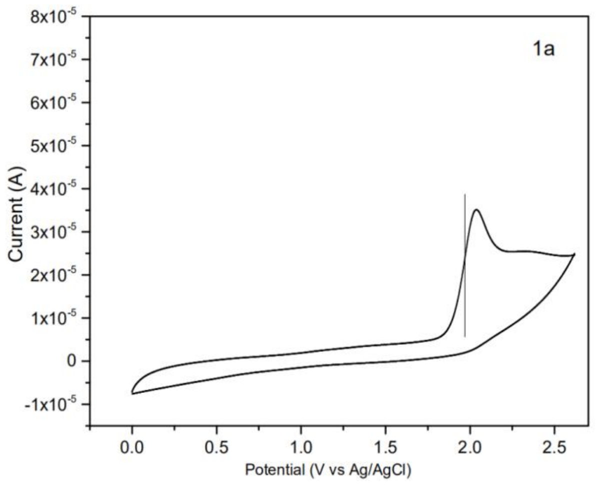

Supplementary Figure 12 Redox potential of **1a** ($E^{1a^{+\bullet}/1a}$ = +1.86 V vs SCE). It was converted from internal standard Fc$^+$/Fc^0. Source data are provided in a Source Data file.

Deducing the Possible Intermediate B

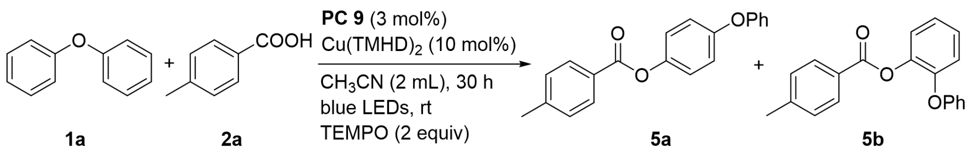

To a quartz tube equipped with a magnetic stirring bar, **PC 9** (3.0 mol%, 3.8 mg), **1a** (0.20 mmol), **2a** (0.24 mmol), bis(2,2,6,6-tetra-Methyl-3,5-heptanedionato)copper(II) (Cu(TMHD)$_2$) (10 mol%, 8.6 mg) and TEMPO (2 equiv) were added. The tube was evacuated and filled with argon three times with each cycle in 15 minutes. Freshly distilled CH_3CN (2.0 mL) was then added into the tube via a syringe under argon atmosphere, then stirred and irradiated with 425-430 nm blue LEDs at ambient temperature (19-21 °C) in a Wattecs Parallel Reactor (Supplementary Figure 1 (a)) for 30 h. After the reaction, the solvent was removed in vacuo and the residue was purified by column chromatography (petroleum ether/EtOAc = 200/1-100/1) to afford the total yield of **5a** and **5b** (22.5 mg) in 37% yield with the ratio of 5.6 :1.0. The standard compounds of **5a** and **5b** were synthesized according to the reported literature.[25]

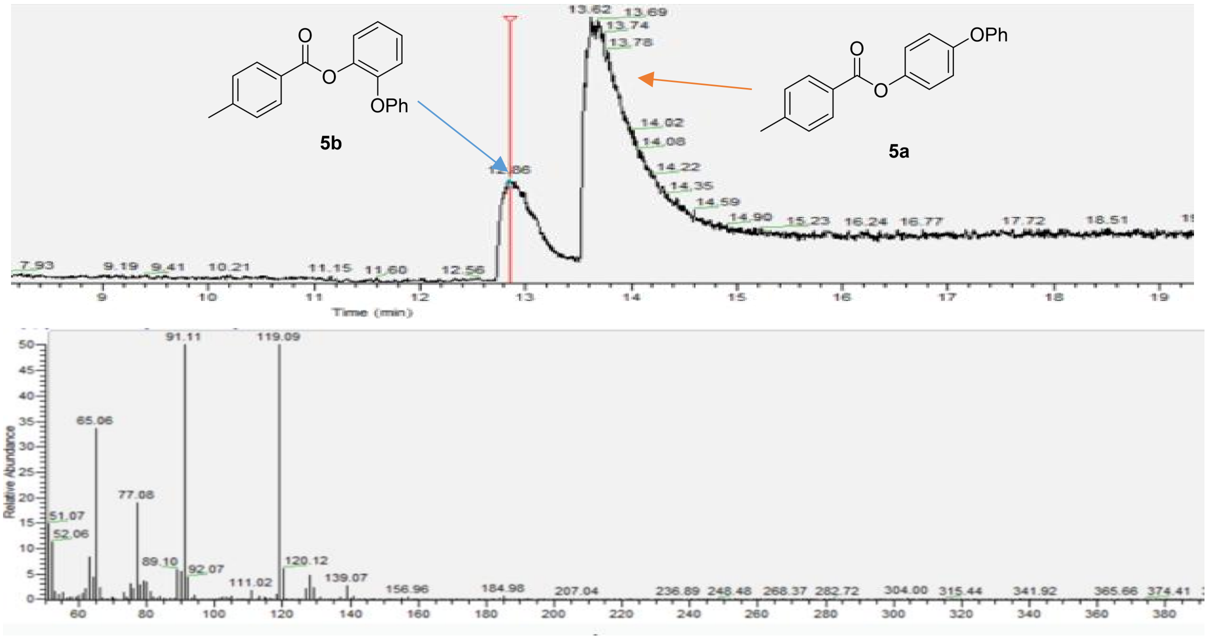

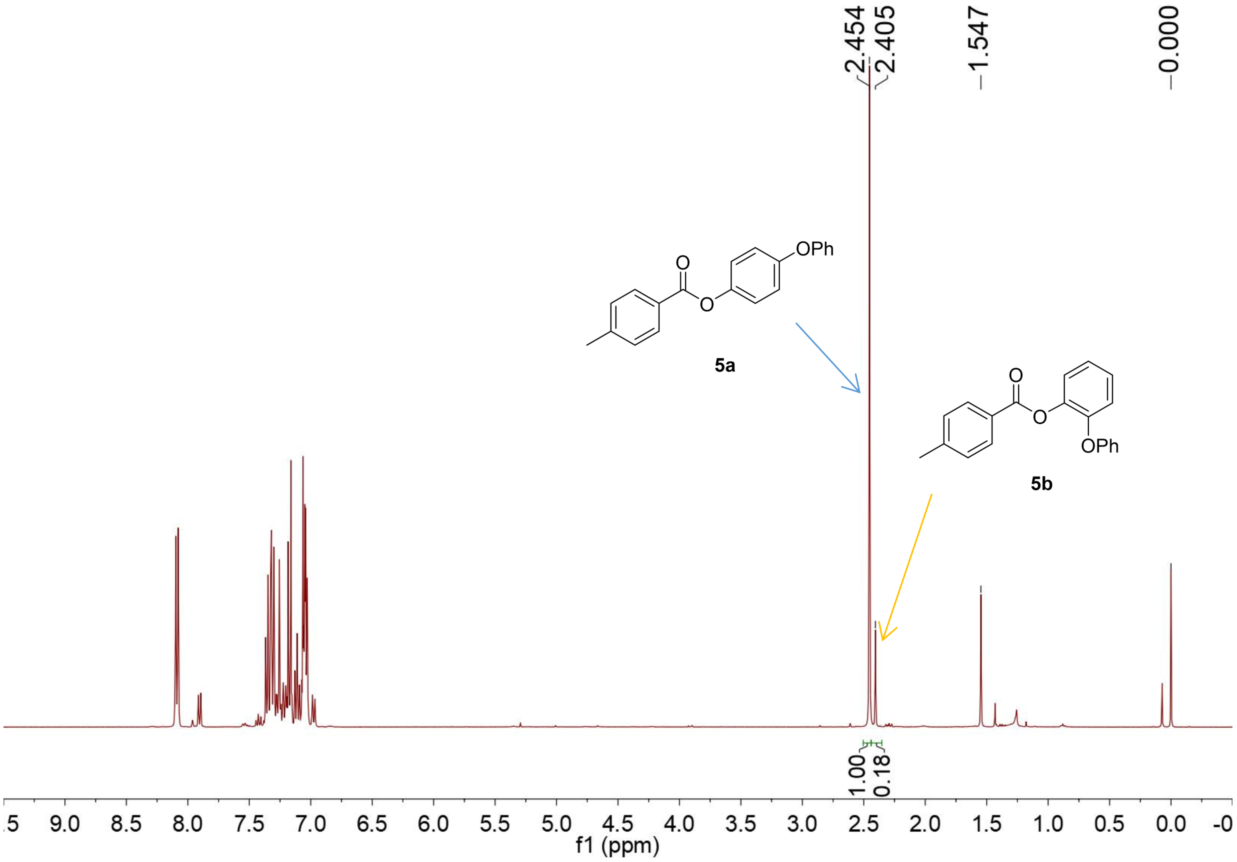

Supplementary Figure 13 The mixed ^{1}H NMR spectra of **5a** and **5b.**

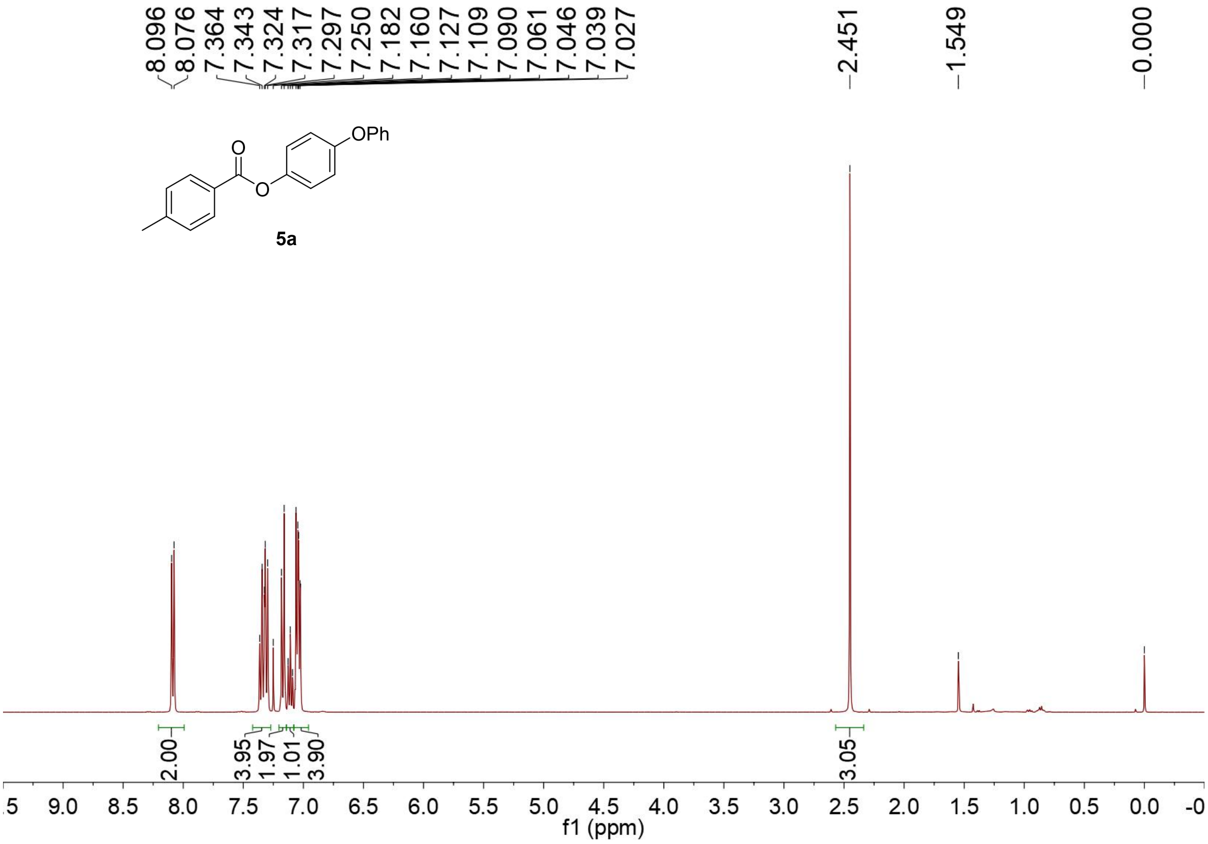

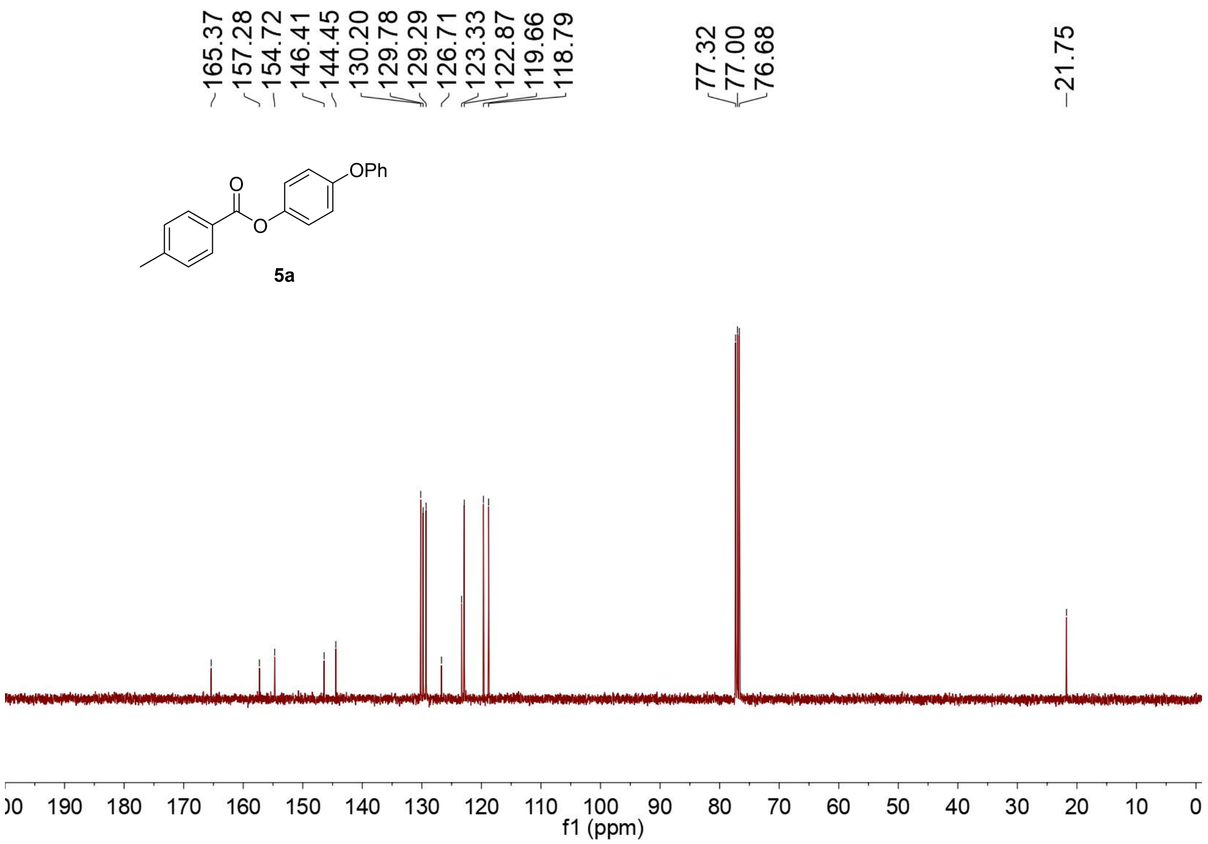
165.37
157.28
154.72
146.41
144.45
130.20
129.78
129.29
126.71
123.33
122.87
119.66
118.79
77.32
77.00
76.68
21.75
OPh
O
O
190
180
170
160
150
140
130
120
110
100
90
80
70
60
50
40
30
20
10
0
f1 (ppm)

5a

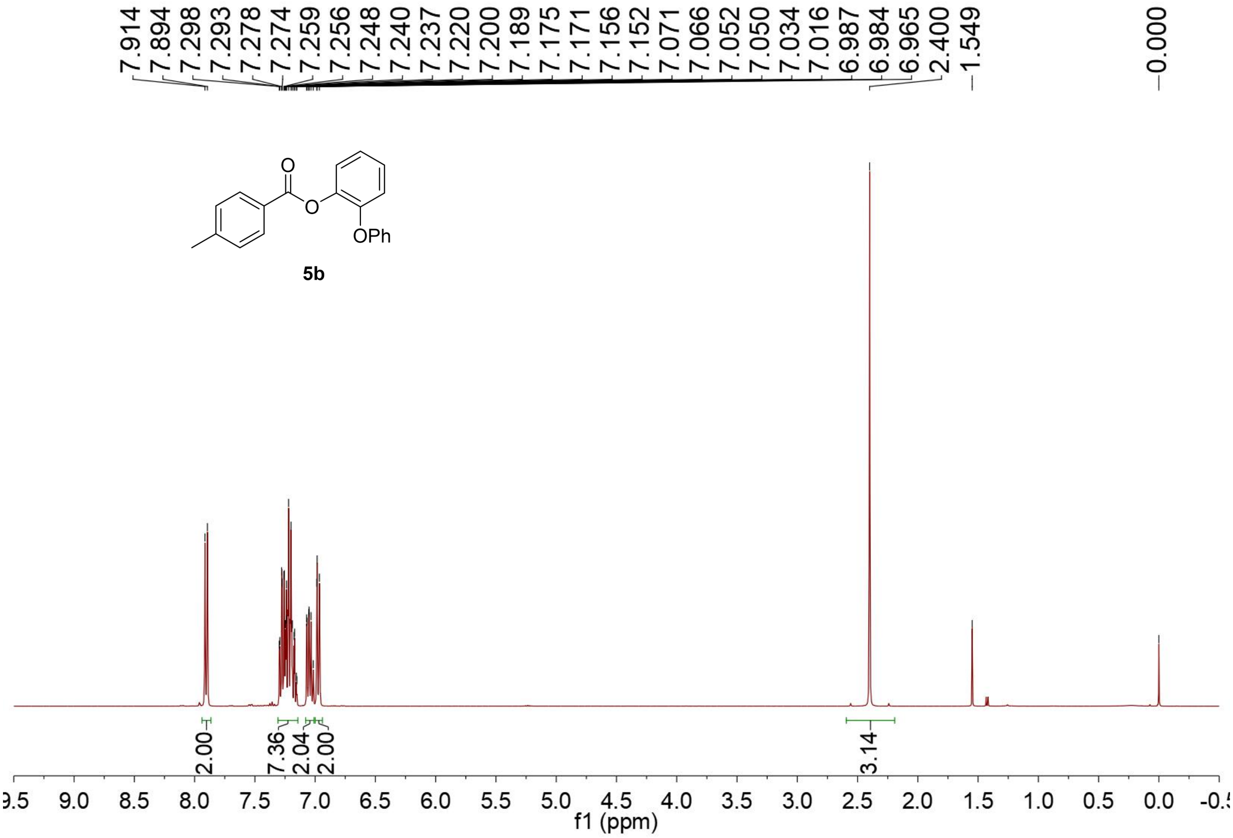
7.914
7.894
7.298
7.293
7.278
7.274
7.259
7.256
7.248
7.240
7.237
7.220
7.200
7.189
7.175
7.171
7.156
7.152
7.071
7.066
7.052
7.050
7.034
7.016
6.987
6.984
6.965
2.400
1.549
0.000
O
O
OPh
2.00
7.36
2.04
2.00
3.14
9.0
8.5
8.0
7.5
7.0
6.5
6.0
5.5
5.0
4.5
4.0
3.5
3.0
2.5
2.0
1.5
1.0
0.5
0.0
f1 (ppm)

5b

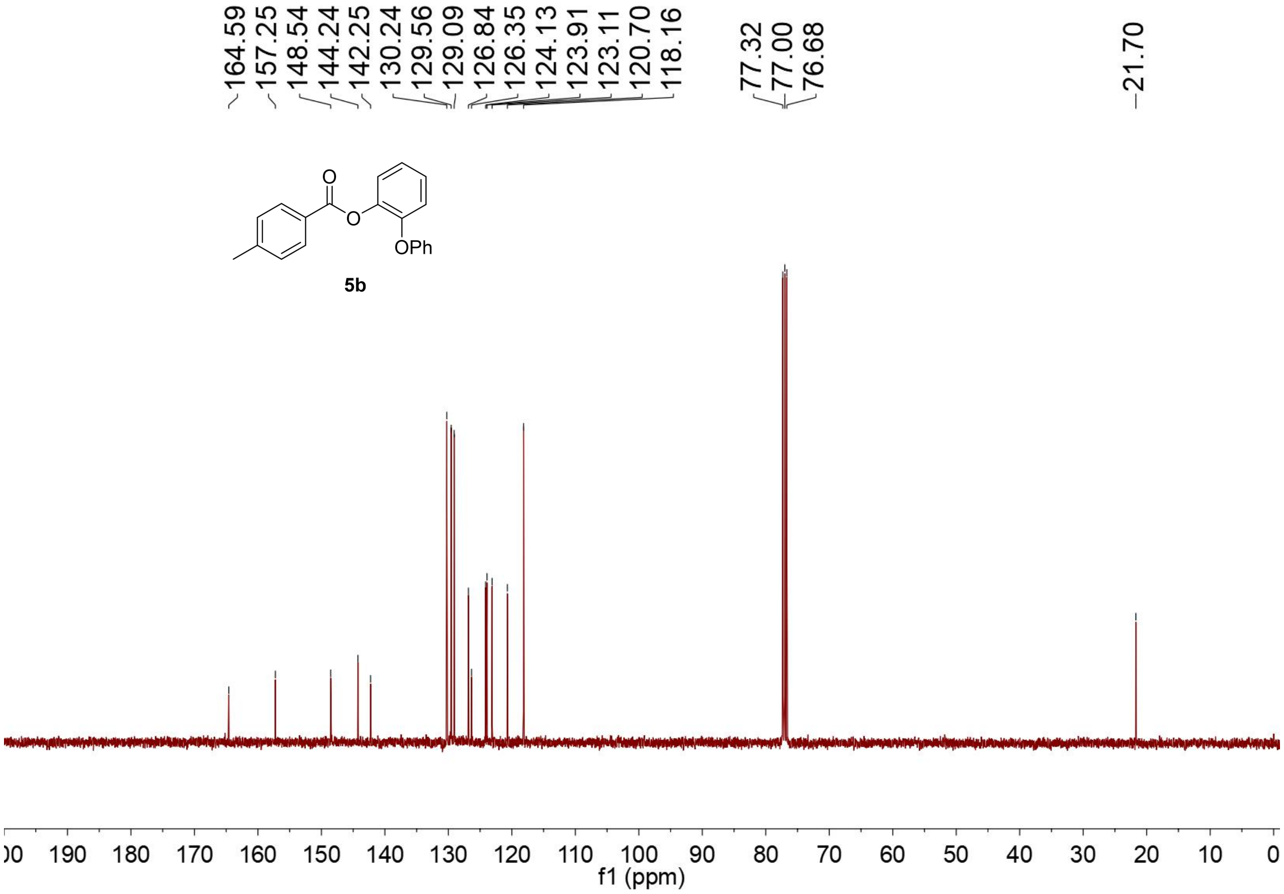

Supplementary Figure 14 The standard NMR spectra of **5a** and **5b**.

Redox Properties and the Fluorescence Lifetime of Acridinium Salts

Cyclic voltammetry experiments were studied on a CHI660E Electrochemical Workstation equipped with the conventional three electrode system under argon atmosphere. The measurements were performed in CH_3CN containing 0.1 M NBu_4PF_6 and 3 mM photo-catalyst using ferrocene/ferrocenium (Fc^+/Fc^0) as an internal reference. The working electrode was a gold plate electrode (d = 0.3 cm). The auxiliary and reference electrodes consisted of a Pt tablets (or reticulated platinum) and an Ag/$AgNO_3$ (0.1 M in $AgNO_3$), respectively. The scan rate was set as 50 mV/s. Data was analyzed with Origin by determining the potential ($E_{p/2}$) at half maximum current value ($C_{p/2}$). The obtained value was referenced to Fc^+/Fc^0 and converted to SCE by adding 0.4 V. Excited state reduction potentials ($E_{p/2*}$) were calculated by subtracting the ground-state reduction potential ($E_{p/2}$), obtained by cyclic voltammetry, from the excitation energy ($E_{0,0}$); $E_{0,0}$ is determined by calculating the energy of the wavelength at which the substrate's UV-Vis absorption and emission spectra overlap.

Samples for the fluorescence lifetime were prepared in a argon-filled glovebox using anhydrous acetonitrile, which was freshly distilled from CaH_2. The solution of the photo-catalyst was diluted to a concentration of 15 μM with a total volume of 3 mL before being transferred to a 3 mL quartz cell and sealed with a PTFE-lined screw cap. The solvent absorbance background was subtracted. Emission spectra (1 nm step size, 14 nm bandwidth) are fully set for the spectral response of the instrument. The photoluminescence quantum efficiency, time-resolved emission spectra and lifetime were obtained using Edinburgh FLSP980 fluorescence spectrophotometer equipped with a xenon lamp (Xe900), a picosecond pulsed laser (EPL-450), a microsecond flash-lamp (μF900) and an integrating sphere, respectively.

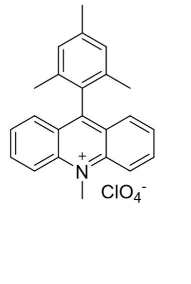

PC 1 (Acr-Mes$^+$ClO$_4^-$)

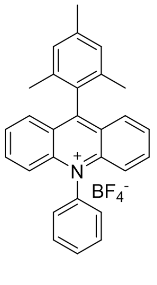

PC 2

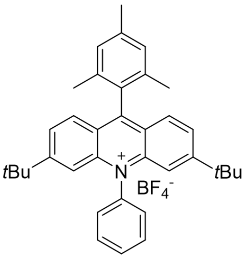

PC 3

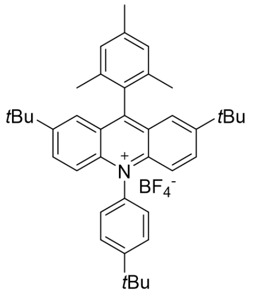

PC 4

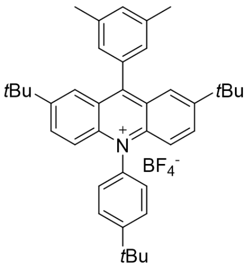

PC 5

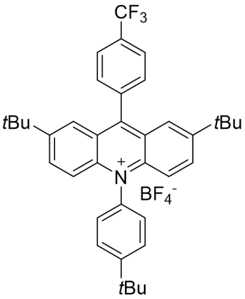

PC 6

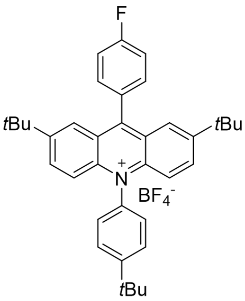

PC 7

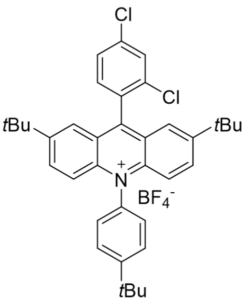

PC 8

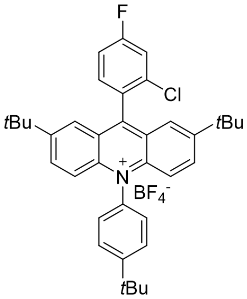

PC 9

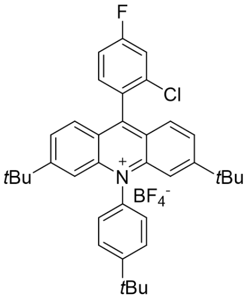

PC 10

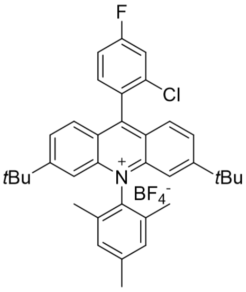

PC 11

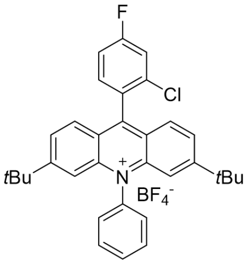

PC 12

Supplementary Figure 15 Structure of photocatalysts.

Supplementary Table 2 Physical Properties of Photocatalysts.

entry	PC	$E_{0,0}$ (eV)[a]	$E_{1/2}$ (C/C$^-$)[b]	$E_{1/2}$ (C*/C$^-$)[c]	τ (ns)[d]	Absorption λ_{max} (nm)	emission λ_{max} (nm)	**3-3a** yield (%)[e]	**3-4a** yield (%)[e]
1	**PC 1**	2.71	-0.57	2.14	8.3	422	503	15	10
2	**PC 2**	2.70	-0.56	2.14	11.2	427	501	29	22
3	**PC 3**	2.70	-0.55	2.15	13.4	421	495	55	49
4	**PC 4**	2.66	-0.48	2.18	12.1	427	498	46	41
5	**PC 5**	2.61	-0.56	2.05	8.8	430	510	61	48
6	**PC 6**	2.62	-0.56	2.06	11.3	430	514	67	62
7	**PC 7**	2.61	-0.60	2.01	9.1	431	509	74	65
8	**PC 8**	2.61	-0.46	2.15	12.5	430	506	78	66
9	**PC 9**	2.63	-0.47	2.16	13.3	429	502	80	71
10	**PC 10**	2.66	-0.41	2.25	6.3	422	507	63	59
11	**PC 11**	2.69	-0.37	2.32	28.6	423	492	50	48
12	**PC 12**	2.67	-0.47	2.20	14.5	422	495	71	69

Scope and photophysical properties of acridinium photocatalysts.
[a]Excited-state reduction potentials were estimated from the ground-state reduction potentials and excited-state energies (E0,0).
[b]Ground-state reduction potentials were determined by cyclic voltammetry (reported vs.SCE).
[c]Excited-state energies were determined from the point of intersection of the normalized absorption and emission spectra.
[d]Fluorescence lifetimes were determined from time-correlated single-photon counting.
[e]Reaction condtions: **1a** (0.24 mmol), **2a** (0.2 mmol), **PC** (3.0 mol %), Lewis acid (10 mol %), CH_3CN (2.0 mL), irradiation with blue LEDs for 30 h, **3a** and **4a** were reported by using $Cl_2CHCHCl_2$ as an internal standard.

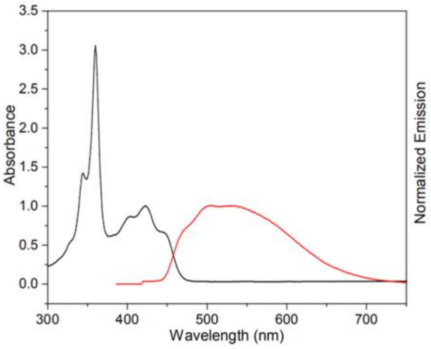

Supplementary Figure 16 Absorption and emission spectra of **PC 1**. The black line is the ultraviolet absorption spectrum, and the red line is the fluorescence emission spectrum. Source data are provided in a Source Data file.

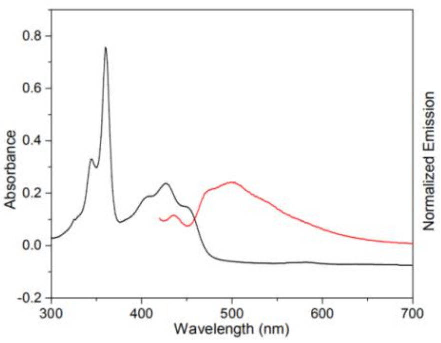

Supplementary Figure 17 Absorption and emission spectra of **PC 2**. The black line is the ultraviolet absorption spectrum, and the red line is the fluorescence emission spectrum. Source data are provided in a Source Data file.

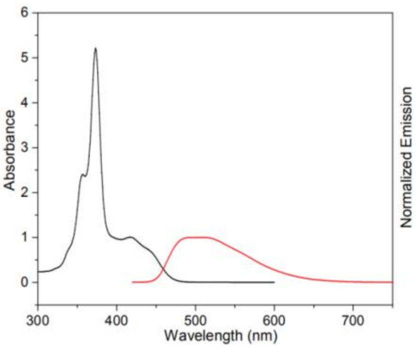

Supplementary Figure 18 Absorption and emission spectra of **PC 3**.The black line is the ultraviolet absorption spectrum, and the red line is the fluorescence emission spectrum. Source data are provided in a Source Data file.

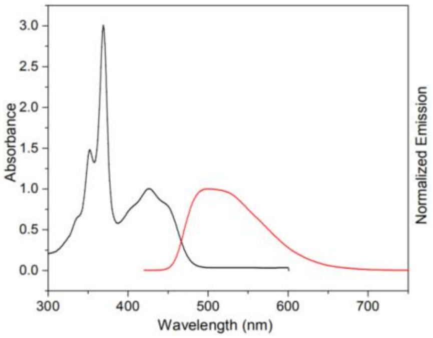

Supplementary Figure 19 Absorption and emission spectra of **PC 4**. The black line is the ultraviolet absorption spectrum, and the red line is the fluorescence emission spectrum. Source data are provided in a Source Data file.

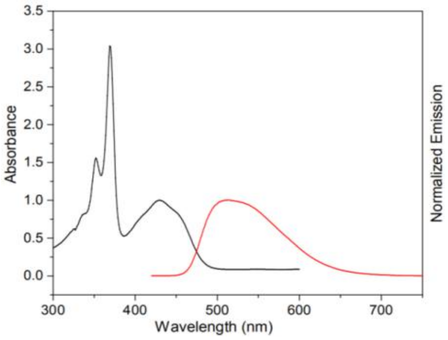

Supplementary Figure 20 Absorption and emission spectra of **PC 5**. The black line is the ultraviolet absorption spectrum, and the red line is the fluorescence emission spectrum. Source data are provided in a Source Data file.

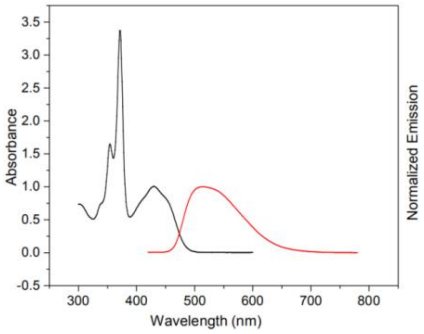

Supplementary Figure 21 Absorption and emission spectra of **PC 6**. The black line is the ultraviolet absorption spectrum, and the red line is the fluorescence emission spectrum. Source data are provided in a Source Data file.

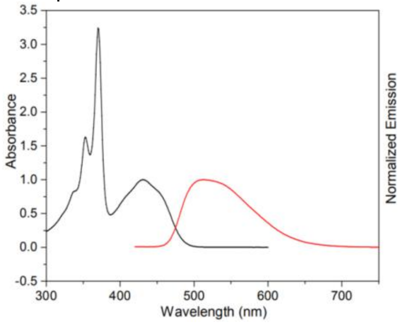

Supplementary Figure 22 Absorption and emission spectra of **PC 7**. The black line is the ultraviolet absorption spectrum, and the red line is the fluorescence emission spectrum. Source data are provided in a Source Data file.

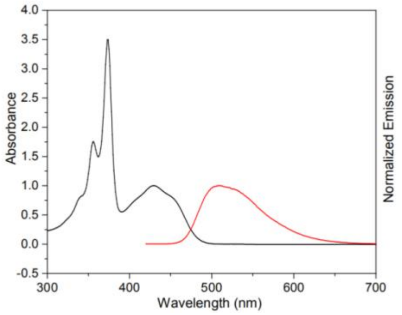

Supplementary Figure 23 Absorption and emission spectra of **PC 8**. The black line is the ultraviolet absorption spectrum, and the red line is the fluorescence emission spectrum. Source data are provided in a Source Data file.

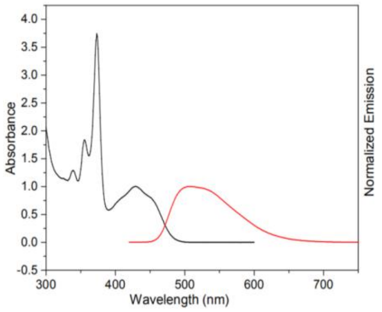

Supplementary Figure 24 Absorption and emission spectra of **PC 9**. The black line is the ultraviolet absorption spectrum, and the red line is the fluorescence emission spectrum. Source data are provided in a Source Data file.

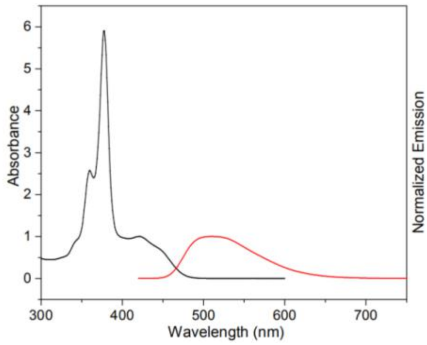

Supplementary Figure 25 Absorption and emission spectra of **PC 10**. The black line is the ultraviolet absorption spectrum, and the red line is the fluorescence emission spectrum. Source data are provided in a Source Data file.

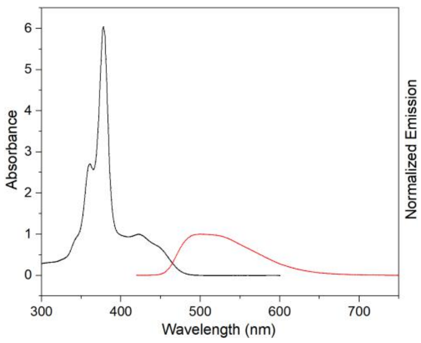

Supplementary Figure 26 Absorption and emission spectra of **PC 11**. The black line is the ultraviolet absorption spectrum, and the red line is the fluorescence emission spectrum. Source data are provided in a Source Data file.

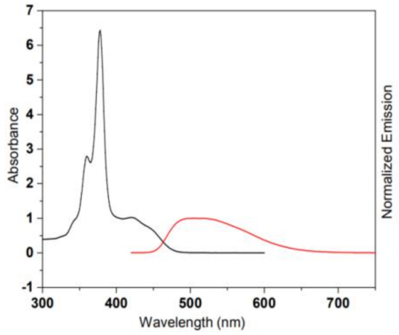

Supplementary Figure 27 Absorption and emission spectra of **PC 12**. The black line is the ultraviolet absorption spectrum, and the red line is the fluorescence emission spectrum. Source data are provided in a Source Data file.

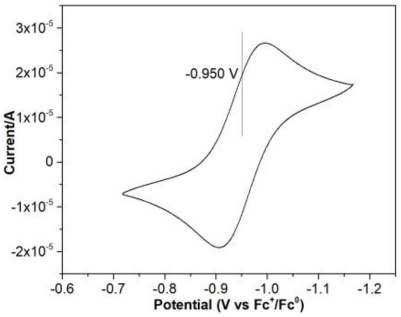

Supplementary Figure 28 The oxidation potention $E^{PC 1/PC 1-\bullet}$ (-0.569 V vs SCE). It was converted from internal standard Fc^+/Fc^0.Source data are provided in a Source Data file.

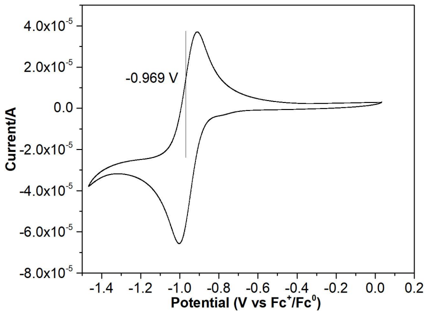

Supplementary Figure 29 The oxidation potention $E^{PC 2/PC 2-\bullet}$ (-0.564 V vs SCE). It was converted from internal standard Fc^+/Fc^0.Source data are provided in a Source Data file.

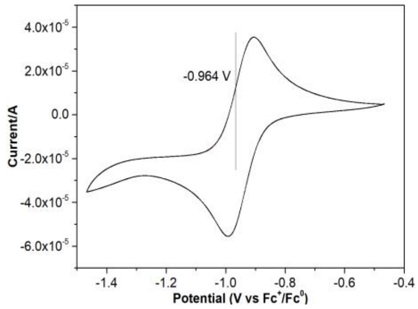

Supplementary Figure 30 The oxidation potention $E^{PC 3/PC 3-\bullet}$ (-0.550 V vs SCE). It was converted from internal standard Fc^+/Fc^0.Source data are provided in a Source Data file.

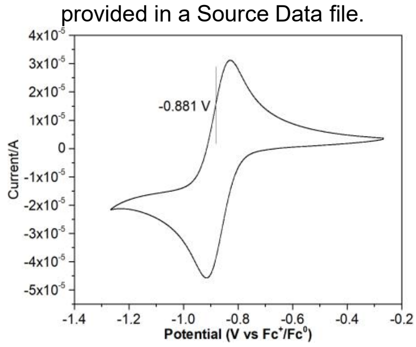

Supplementary Figure 31 The oxidation potention $E^{PC 4/PC 4-\bullet}$ (-0.481 V vs SCE). It was converted from internal standard Fc^+/Fc^0.Source data are provided in a Source Data file.

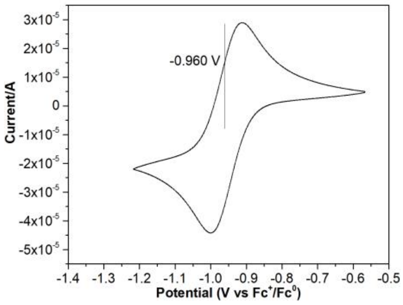

Supplementary Figure 32 The oxidation potention $E^{PC 5/PC 5-\bullet}$ (-0.560 V vs SCE). It was converted from internal standard Fc^+/Fc^0.Source data are provided in a Source Data file.

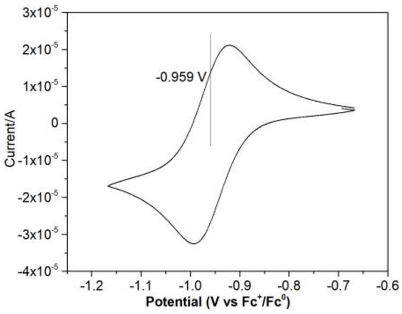

Supplementary Figure 33 The oxidation potention $E^{PC 6/PC 6-\bullet}$ (-0.559 V vs SCE). It was converted from internal standard Fc^+/Fc^0.Source data are provided in a Source Data file.

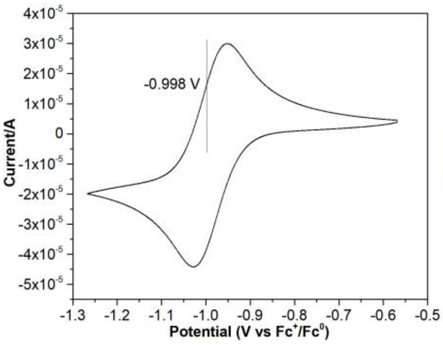

Supplementary Figure 34 The oxidation potention $E^{PC\ 7/PC\ 7-\bullet}$ (-0.598 V vs SCE). It was converted from internal standard Fc^+/Fc^0.Source data are provided in a Source Data file.

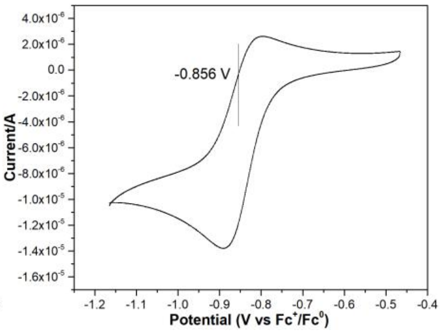

Supplementary Figure 35 The oxidation potention $E^{PC\ 8/PC\ 8-\bullet}$ (-0.456 V vs SCE). It was converted from internal standard Fc^+/Fc^0.Source data are provided in a Source Data file.

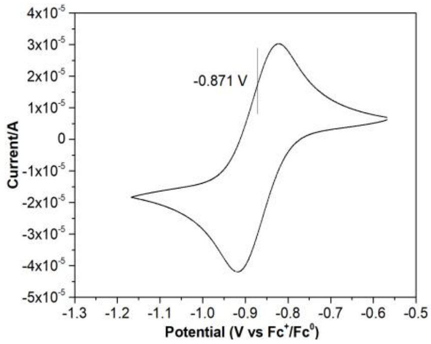

Supplementary Figure 36 The oxidation potention $E^{PC\ 9/PC\ 9-\bullet}$ (-0.471 V vs SCE). It was converted from internal standard Fc^+/Fc^0.Source data are provided in a Source Data file.

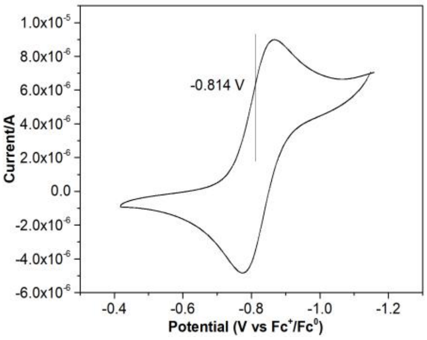

Supplementary Figure 37 The oxidation potention $E^{PC\ 10/PC\ 10-\bullet}$ (-0.414 V vs SCE). It was converted from internal standard Fc^+/Fc^0.Source data are provided in a Source Data file.

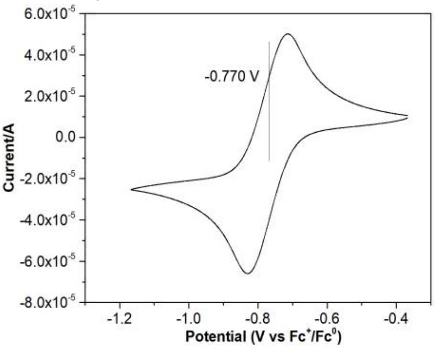

Supplementary Figure 38 The oxidation potention $E^{PC\ 11/PC\ 11-\bullet}$ (-0.370 V vs SCE). It was converted from internal standard Fc^+/Fc^0.Source data are provided in a Source Data file.

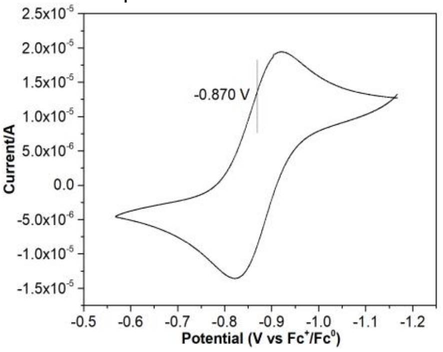

Supplementary Figure 39 The oxidation potention $E^{PC\ 12/PC\ 12-\bullet}$ (-0.470 V vs SCE). It was converted from internal standard Fc^+/Fc^0.Source data are provided in a Source Data file.

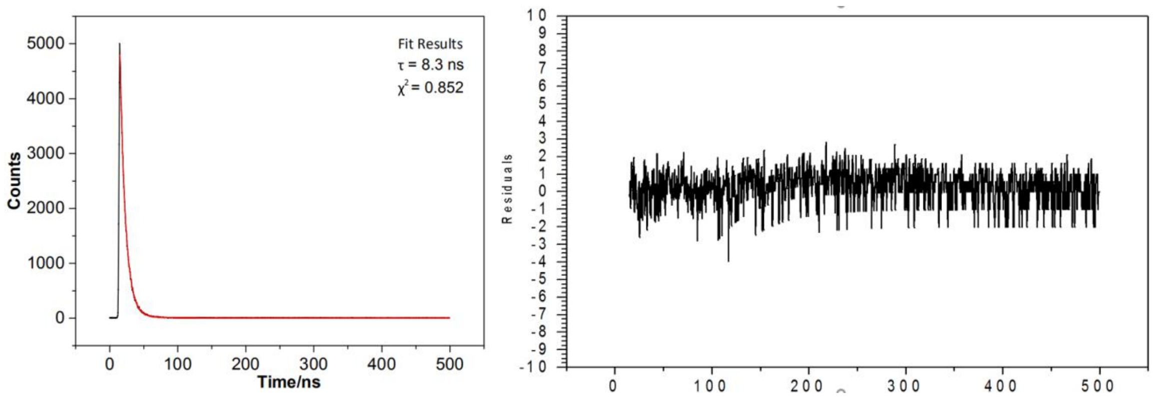

Supplementary Figure 40 Emission decay of **PC 1** in CH_3CN at 298 K. The fluorescence emission bands (left image). Weighted residuals (right image). Source data are provided in a Source Data file.

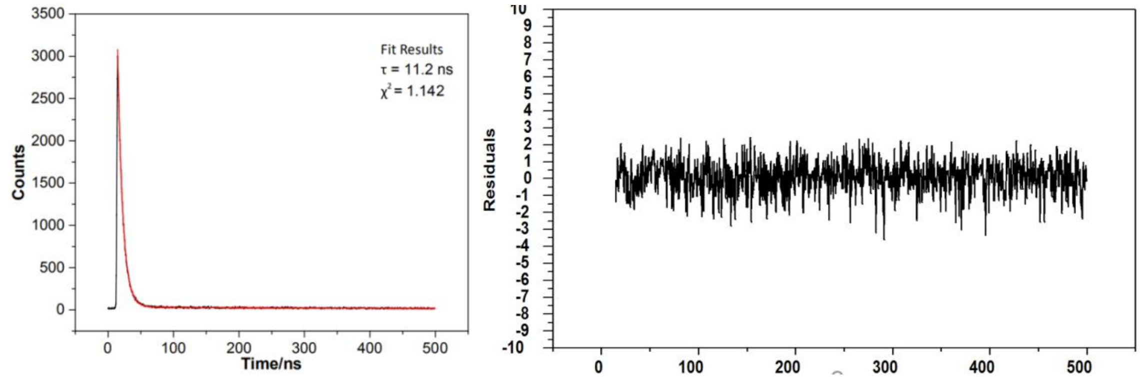

Supplementary Figure 41 Emission decay of **PC 2** in CH_3CN at 298 K. The fluorescence emission bands (left image). Weighted residuals (right image). Source data are provided in a Source Data file.

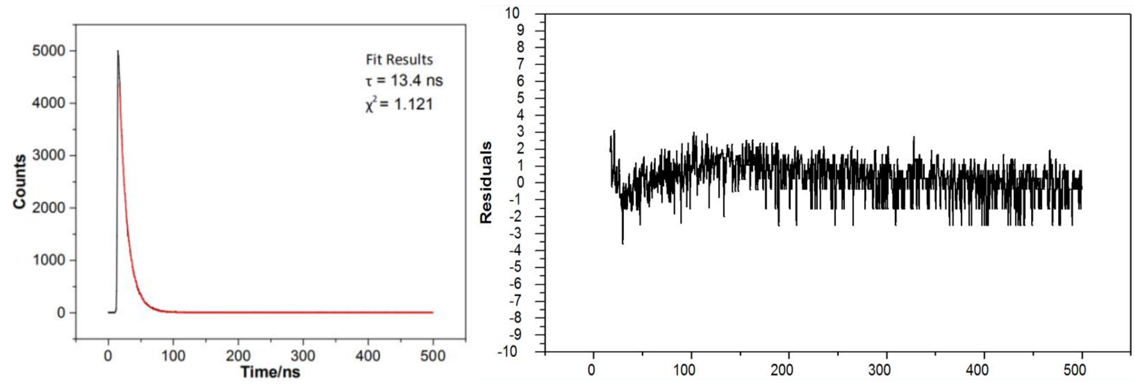

Supplementary Figure 42 Emission decay of **PC 3** in CH_3CN at 298 K. The fluorescence emission bands (left image). Weighted residuals (right image). Source data are provided in a Source Data file.

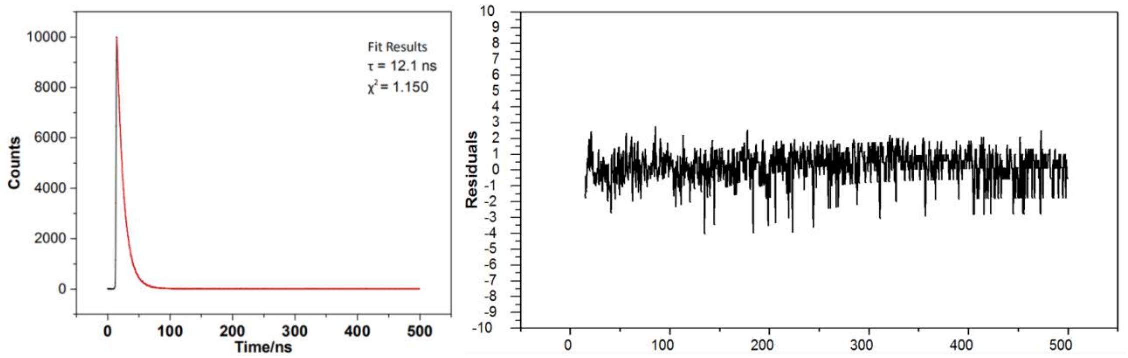

Supplementary Figure 43 Emission decay of **PC 4** in CH_3CN at 298 K. The fluorescence emission bands (left image). Weighted residuals (right image). Source data are provided in a Source Data file.

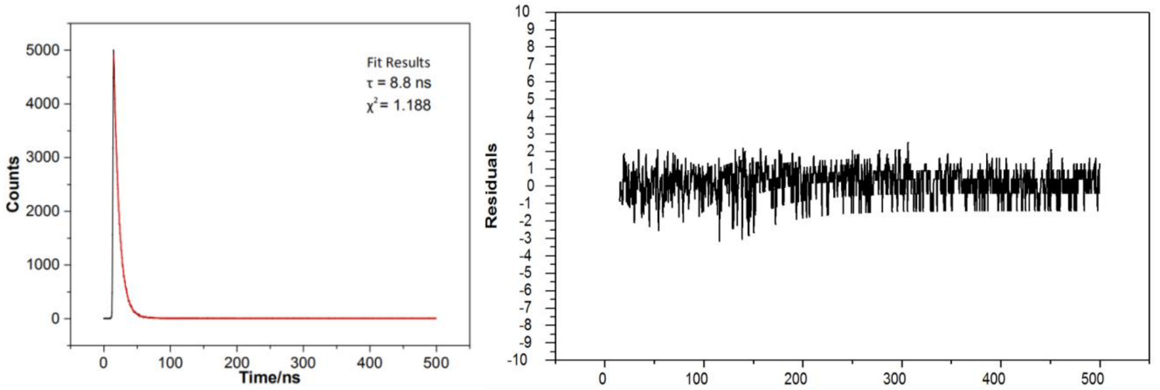

Supplementary Figure 44 Emission decay of **PC 5** in CH_3CN at 298 K. The fluorescence emission bands (left image). Weighted residuals (right image). Source data are provided in a Source Data file.

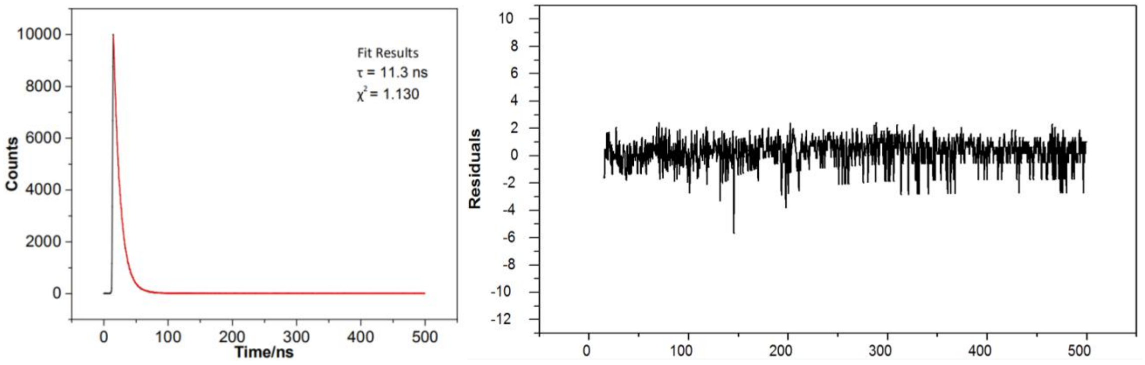

Supplementary Figure 45 Emission decay of **PC 6** in CH_3CN at 298 K.The fluorescence emission bands (left image). Weighted residuals (right image). Source data are provided in a Source Data file.

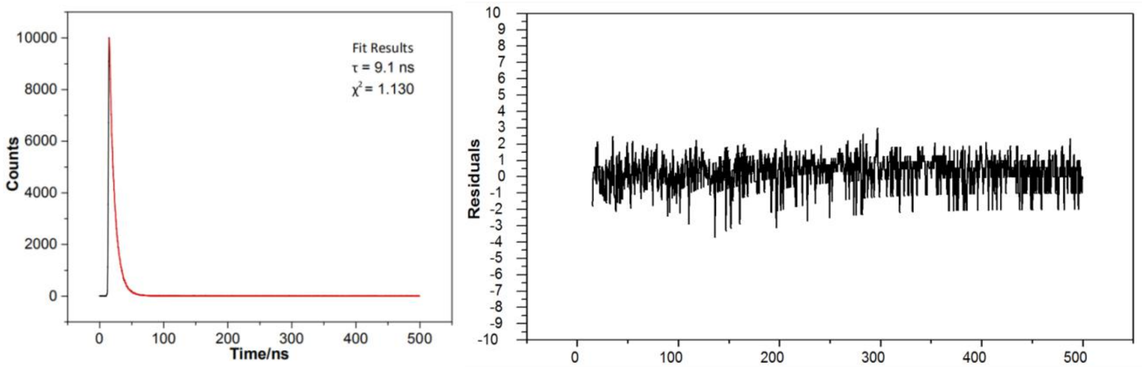

Supplementary Figure 46 Emission decay of **PC 7** in CH_3CN at 298 K. The fluorescence emission bands (left image). Weighted residuals (right image). Source data are provided in a Source Data file.

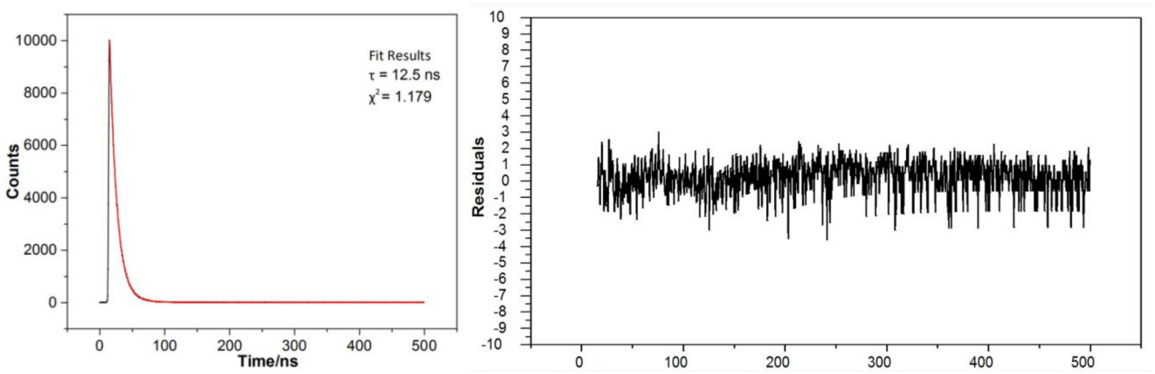

Supplementary Figure 47 Emission decay of **PC 8** in CH_3CN at 298 K. The fluorescence emission bands (left image). Weighted residuals (right image). Source data are provided in a Source Data file.

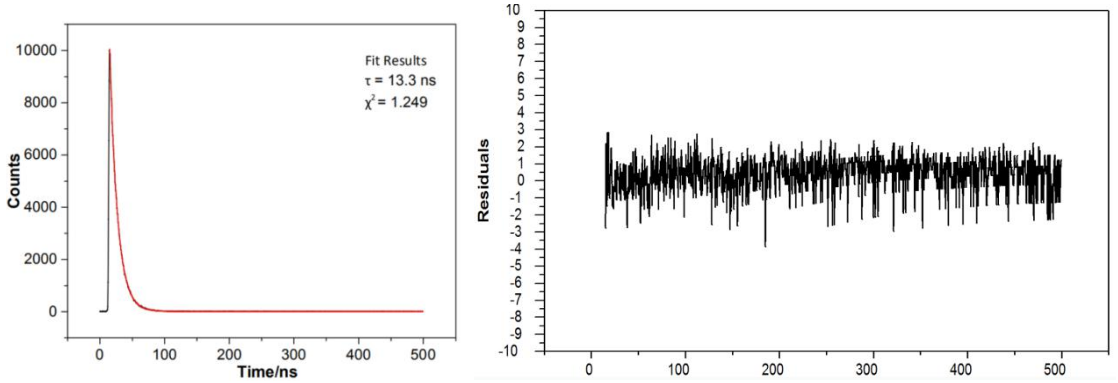

Supplementary Figure 48 Emission decay of **PC 9** in CH_3CN at 298 K. The fluorescence emission bands (left image). Weighted residuals (right image). Source data are provided in a Source Data file.

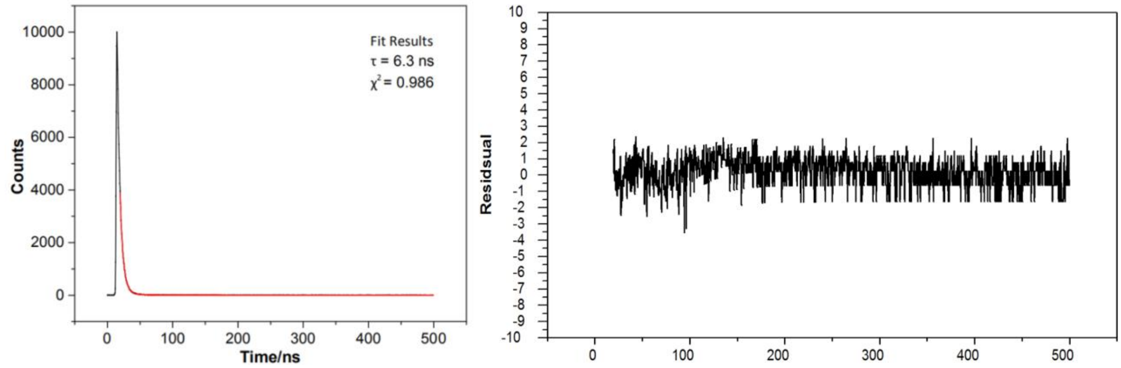

Supplementary Figure 49 Emission decay of **PC 10** in CH_3CN at 298 K. The fluorescence emission bands (left image). Weighted residuals (right image). Source data are provided in a Source Data file.

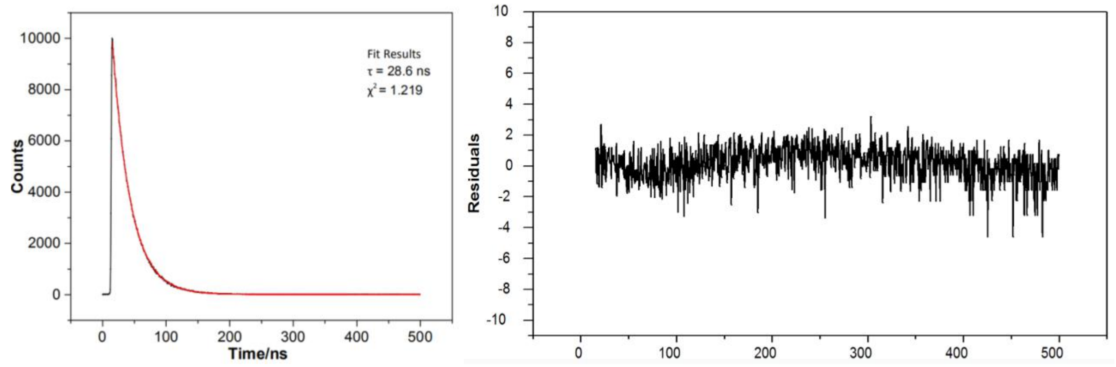

Supplementary Figure 50 Emission decay of **PC 11** in CH_3CN at 298 K. The fluorescence emission bands (left image). Weighted residuals (right image). Source data are provided in a Source Data file.

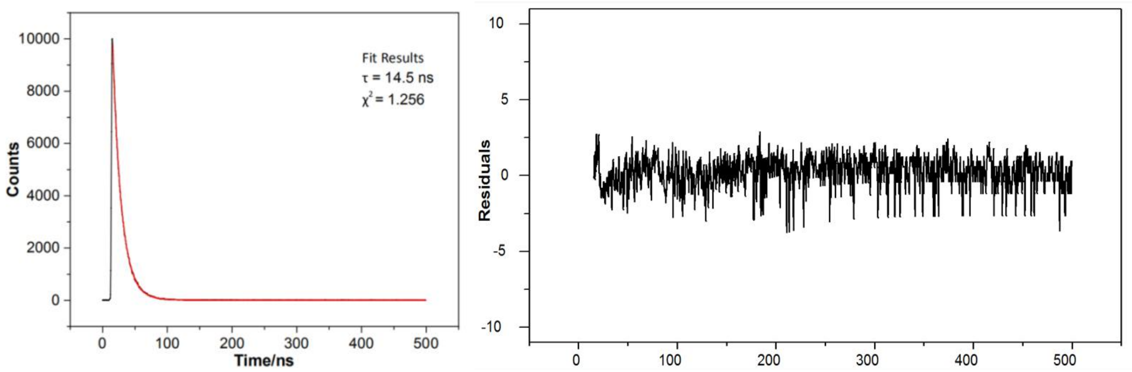

Supplementary Figure 51 Emission decay of **PC 12** in CH_3CN at 298 K. The fluorescence emission bands (left image). Weighted residuals (right image). Source data are provided in a Source Data file.

Supplementary Table 3 Redox Potentials

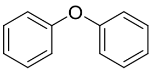<br>**1a**<br>$E^{1a^{+\bullet}/1a}$ = 1.86 V vs SCE	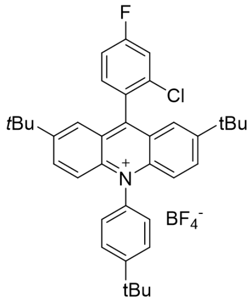<br><br>**PC 9**<br>$E^{PC\ 9^{*}/PC\ 9^{-\bullet}}$ = 2.16 V vs SCE	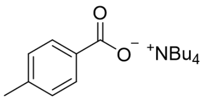<br><br>**2a'**<br>$E^{4\text{-}CH_3PhCO_2\bullet/4\text{-}CH_3PhCO_2^-}$ = 1.45 V vs SCE
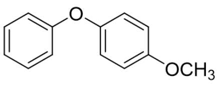<br><br>**1j**<br>$E^{1j^{+\bullet}/1j}$ = 1.39 V vs SCE	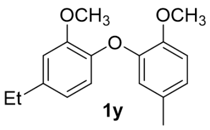<br><br>**1y**<br>$E^{1y^{+\bullet}/1y}$ = 1.22 V vs SCE	

Cyclic voltammetry experiments were studied on a CHI660E Electrochemical Workstation equipped with the conventional three electrode system under argon atmosphere. The measurements were performed in CH_3CN containing 0.1 M NBu_4PF_6 and 3 mM compound using ferrocene/ferrocenium (Fc^+/Fc^0) as an internal reference. The working electrode was a glassy carbon electrode (d = 0.3 cm). The auxiliary and reference electrodes consisted of a Pt tablets and an Ag/AgCl (0.1 M in AgCl), respectively. The scan rate was set as 50 mV/s. Data was analyzed with Origin by determining the potential ($E_{p/2}$) at half maximum current value ($C_{p/2)}$. The obtained value was referenced to Fc^+/Fc^0 (0.511 V) and converted to SCE by adding 0.4 V.

^{1}H NMR and ^{13}C NMR Spectra

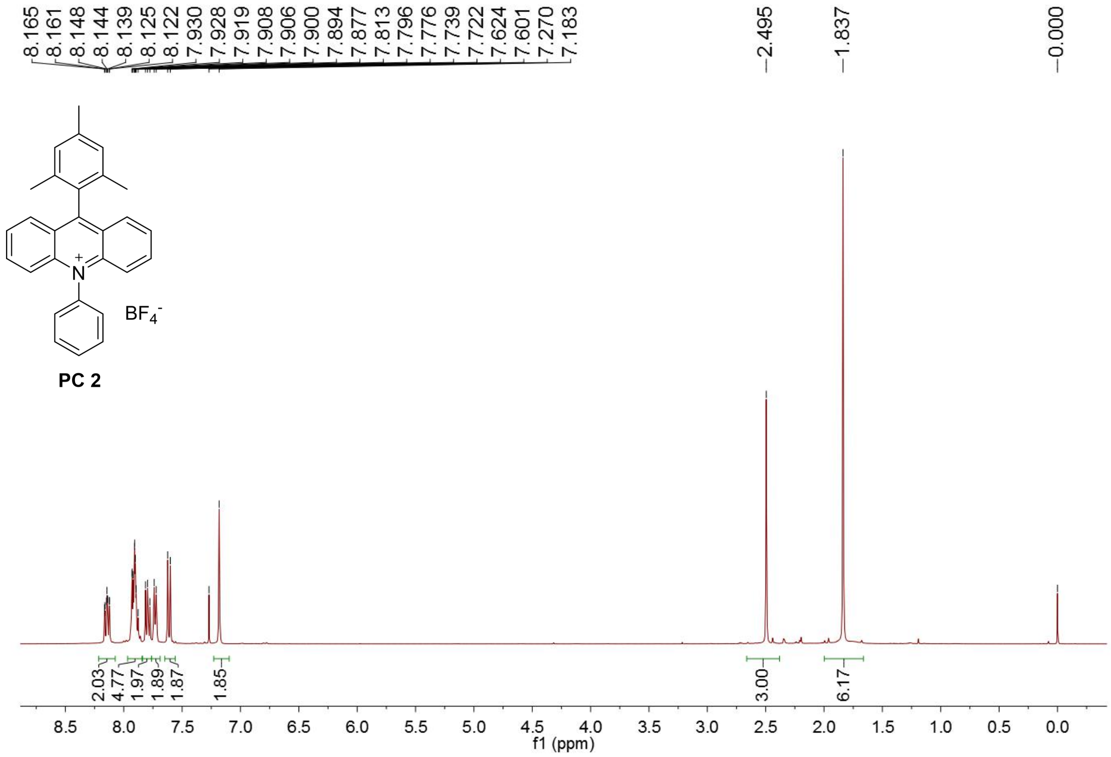

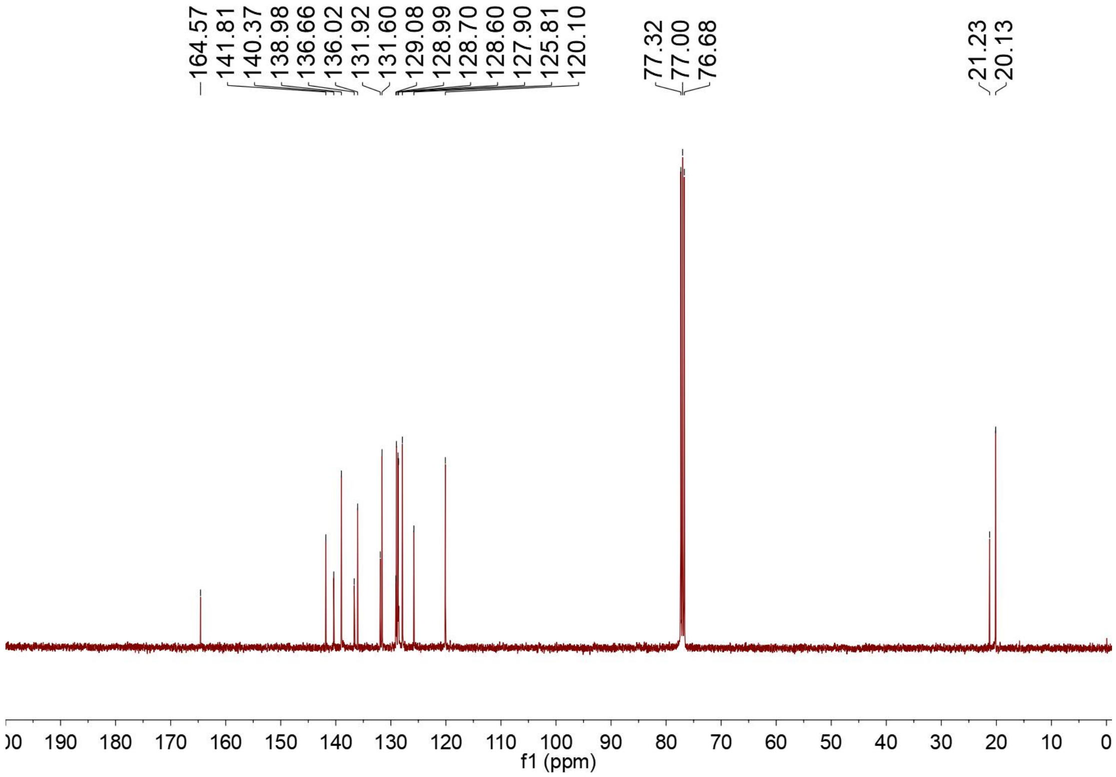

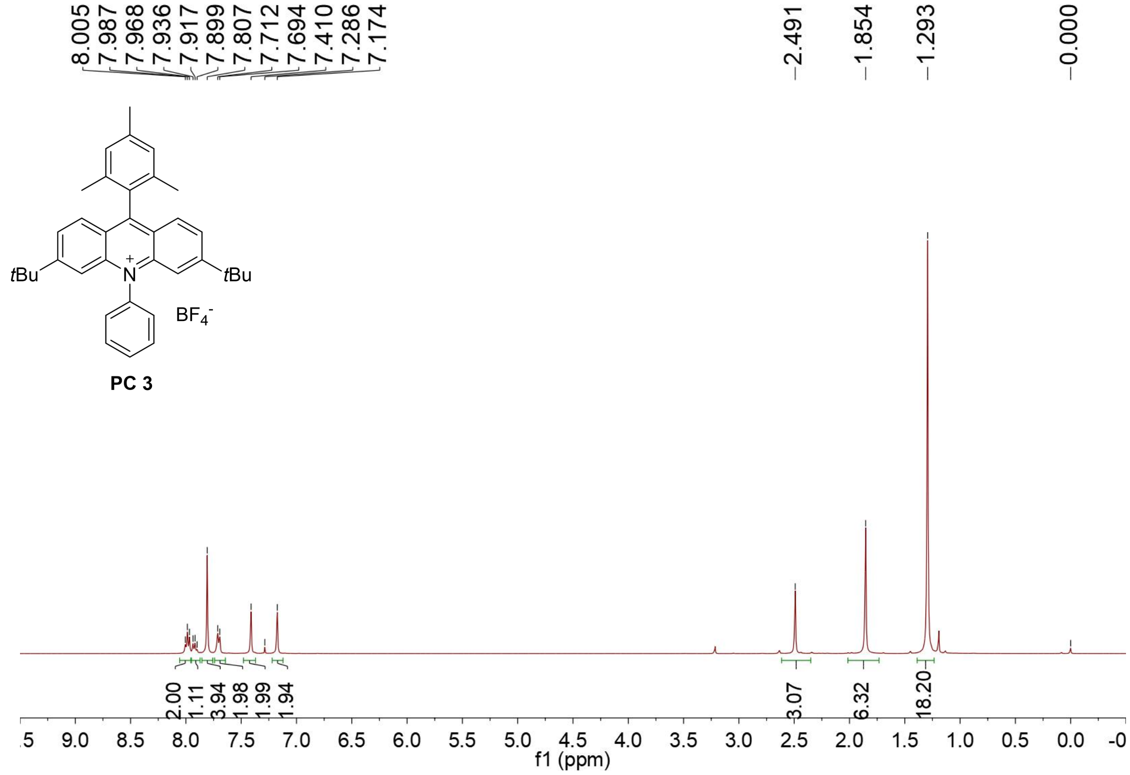
8.005
7.987
7.968
7.936
7.917
7.899
7.807
7.712
7.694
7.410
7.286
7.174
2.491
1.854
1.293
0.000
tBu
tBu
BF_4^-
PC 3
2.00
1.11
3.94
1.98
1.99
1.94
3.07
6.32
18.20
f1 (ppm)

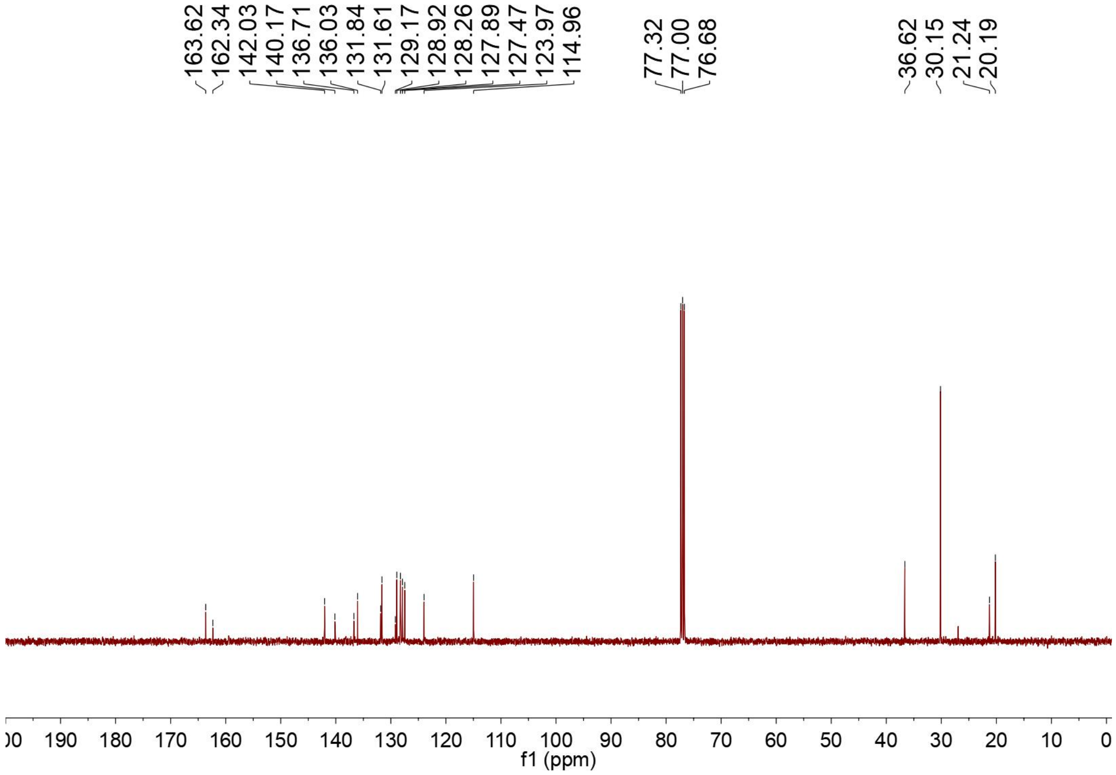
163.62
162.34
142.03
140.17
136.71
136.03
131.84
131.61
129.17
128.92
128.26
127.89
127.47
123.97
114.96
77.32
77.00
76.68
36.62
30.15
21.24
20.19
f1 (ppm)

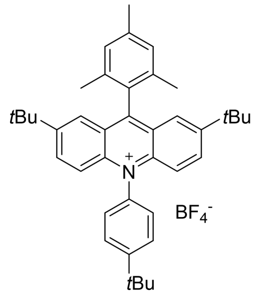
tBu
tBu
N
BF4-
tBu

PC 4

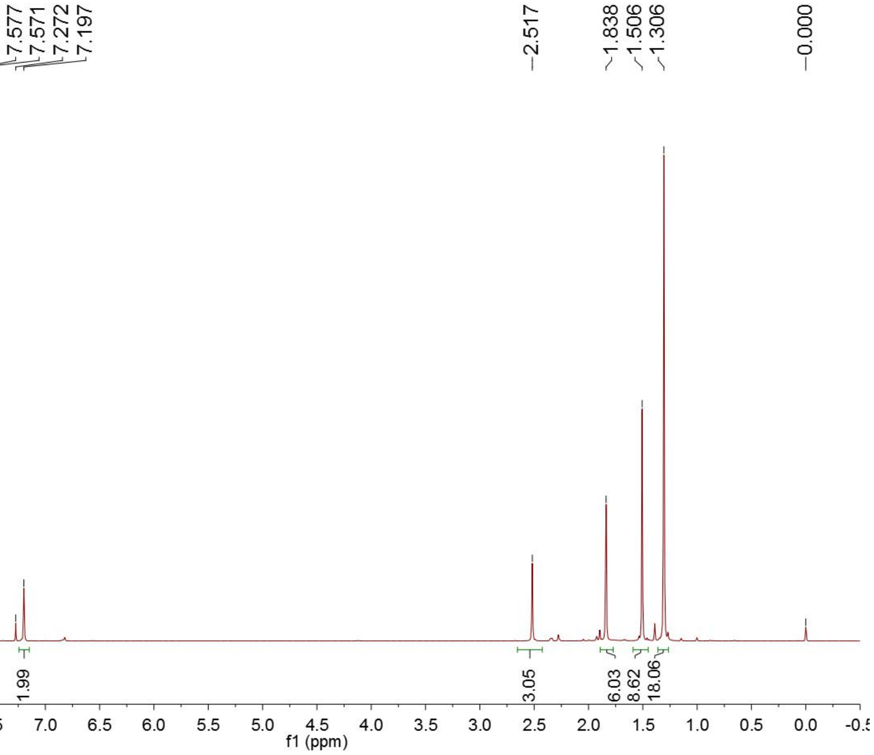
8.212
8.208
8.188
7.912
7.892
7.712
7.592
7.577
7.571
7.272
7.197
2.517
1.838
1.506
1.306
0.000
2.00
1.96
2.02
3.73
1.99
3.05
6.03
8.62
18.06
9.5 9.0 8.5 8.0 7.5 7.0 6.5 6.0 5.5 5.0 4.5 4.0 3.5 3.0 2.5 2.0 1.5 1.0 0.5 0.0
f1 (ppm)

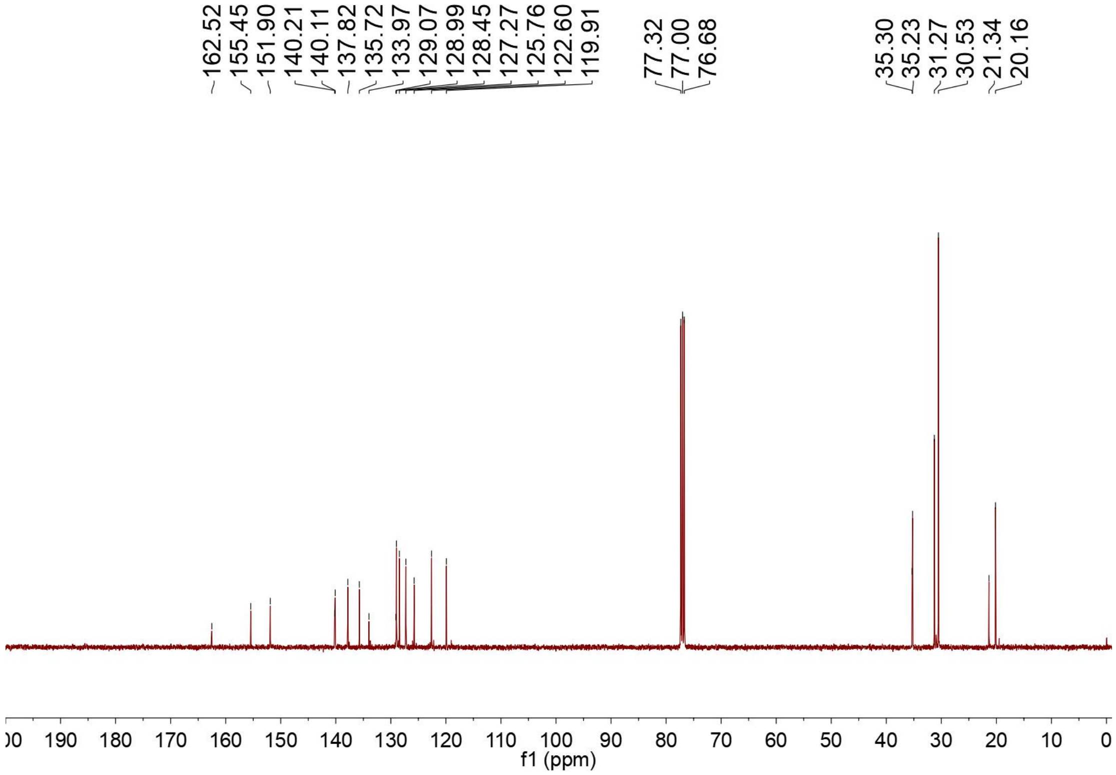
162.52
155.45
151.90
140.21
140.11
137.82
135.72
133.97
129.07
128.99
128.45
127.27
125.76
122.60
119.91
77.32
77.00
76.68
35.30
35.23
31.27
30.53
21.34
20.16
190 180 170 160 150 140 130 120 110 100 90 80 70 60 50 40 30 20 10 0
f1 (ppm)

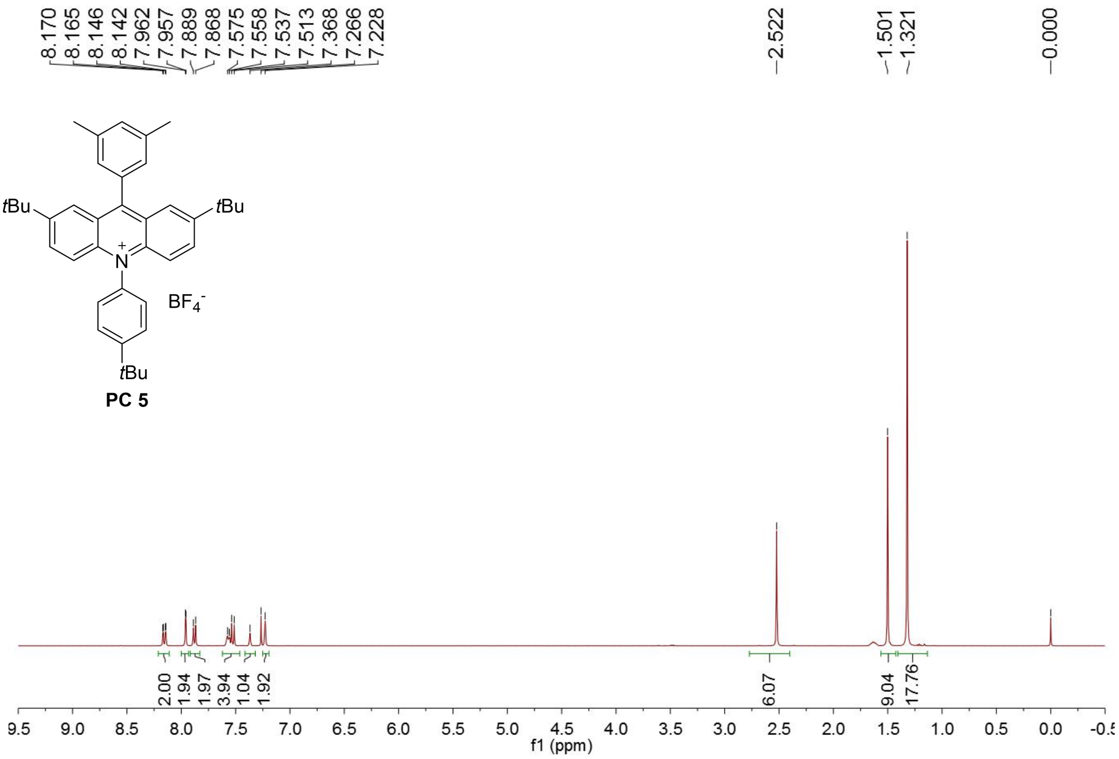
8.170 8.165 8.146 8.142 7.962 7.957 7.889 7.868 7.575 7.558 7.537 7.513 7.368 7.266 7.228
2.522
1.501 1.321
0.000
tBu
tBu
N
BF4-
tBu
PC 5
2.00 1.94 1.97 3.94 1.04 1.92
6.07
9.04 17.76
f1 (ppm)

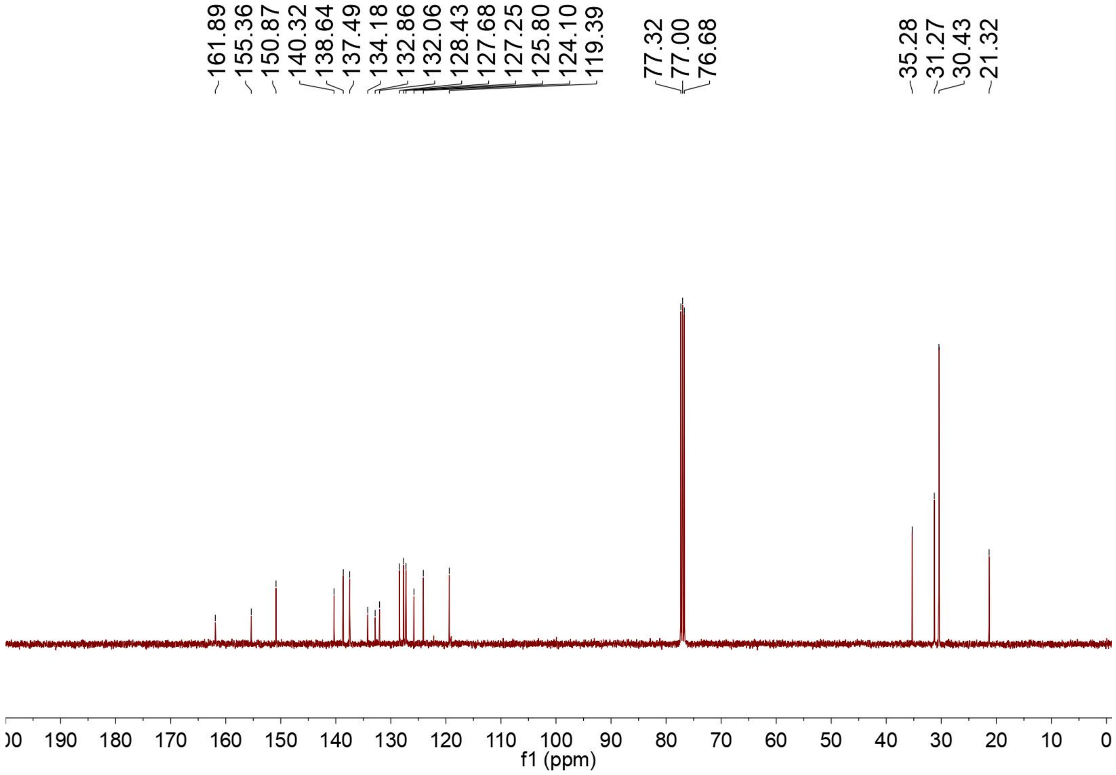
161.89 155.36 150.87 140.32 138.64 137.49 134.18 132.86 132.06 128.43 127.68 127.25 125.80 124.10 119.39
77.32 77.00 76.68
35.28 31.27 30.43 21.32
f1 (ppm)

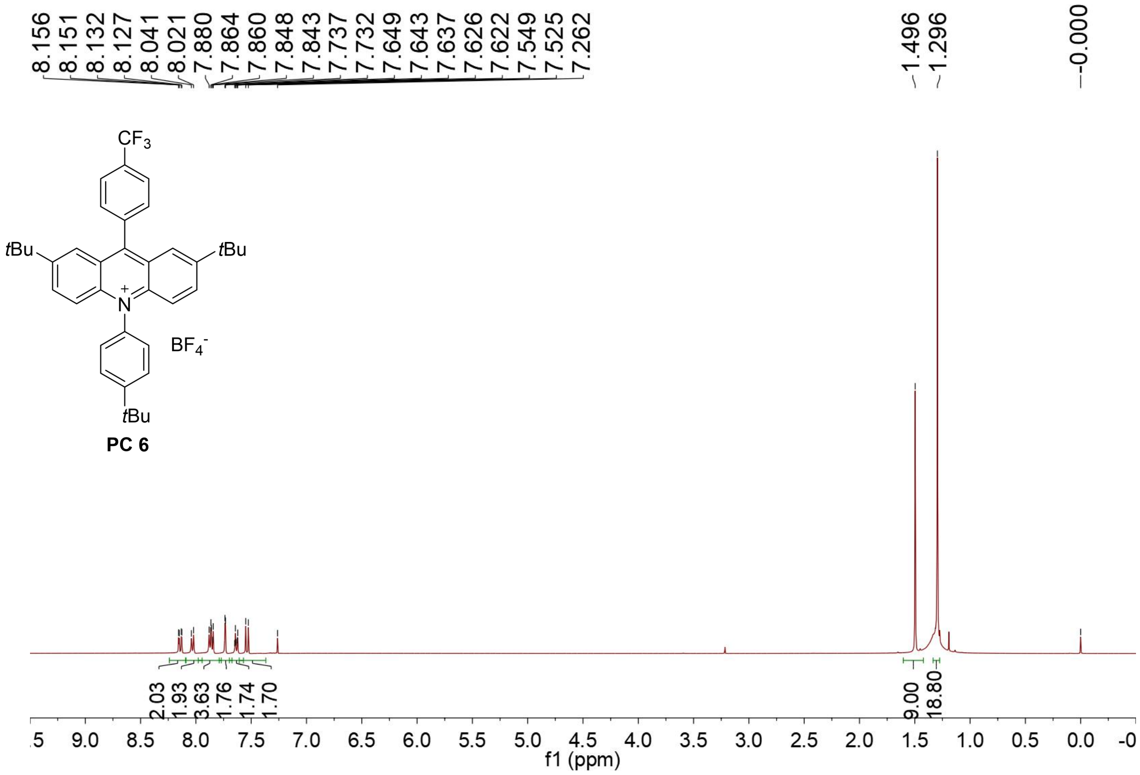
8.156
8.151
8.132
8.127
8.041
8.021
7.880
7.864
7.860
7.848
7.843
7.737
7.732
7.732
7.649
7.643
7.637
7.626
7.622
7.549
7.525
7.262
1.496
1.296
0.000
CF3
tBu
tBu
N
BF4-
tBu
PC 6
2.03
1.93
3.63
1.76
1.74
1.70
9.00
18.80
f1 (ppm)

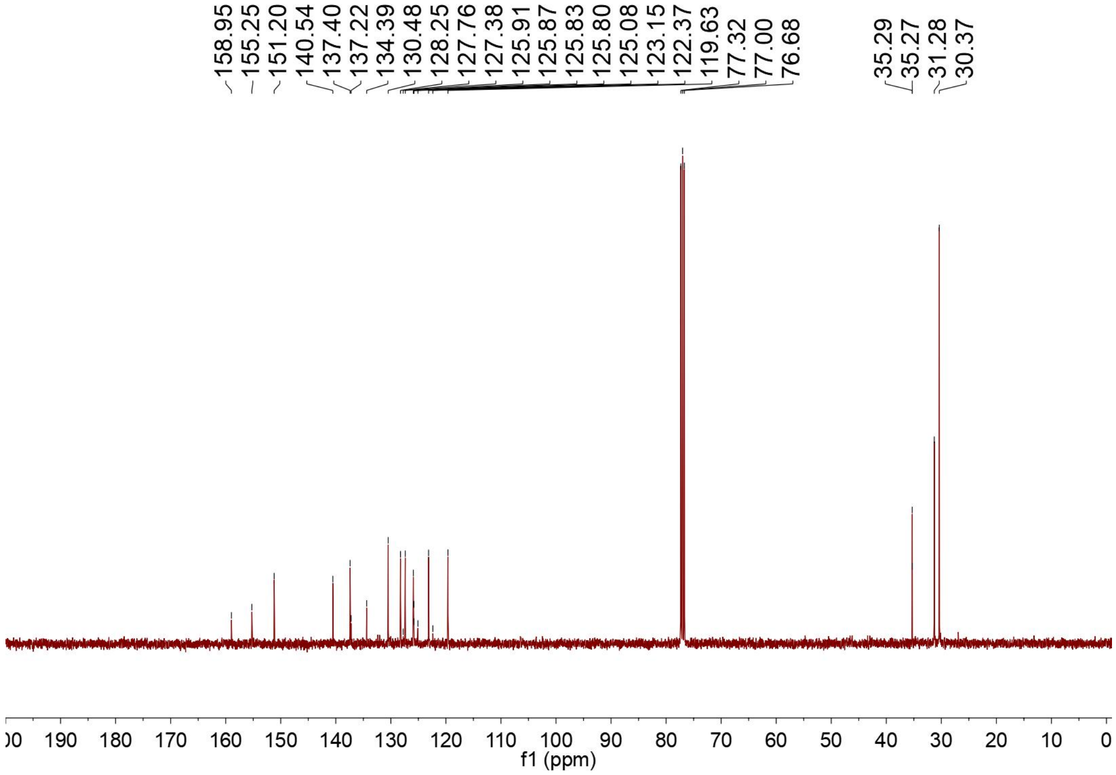
158.95
155.25
151.20
140.54
137.40
137.22
134.39
130.48
128.25
127.76
127.38
125.91
125.87
125.83
125.80
125.08
123.15
122.37
119.63
77.32
77.00
76.68
35.29
35.27
31.28
30.37
f1 (ppm)

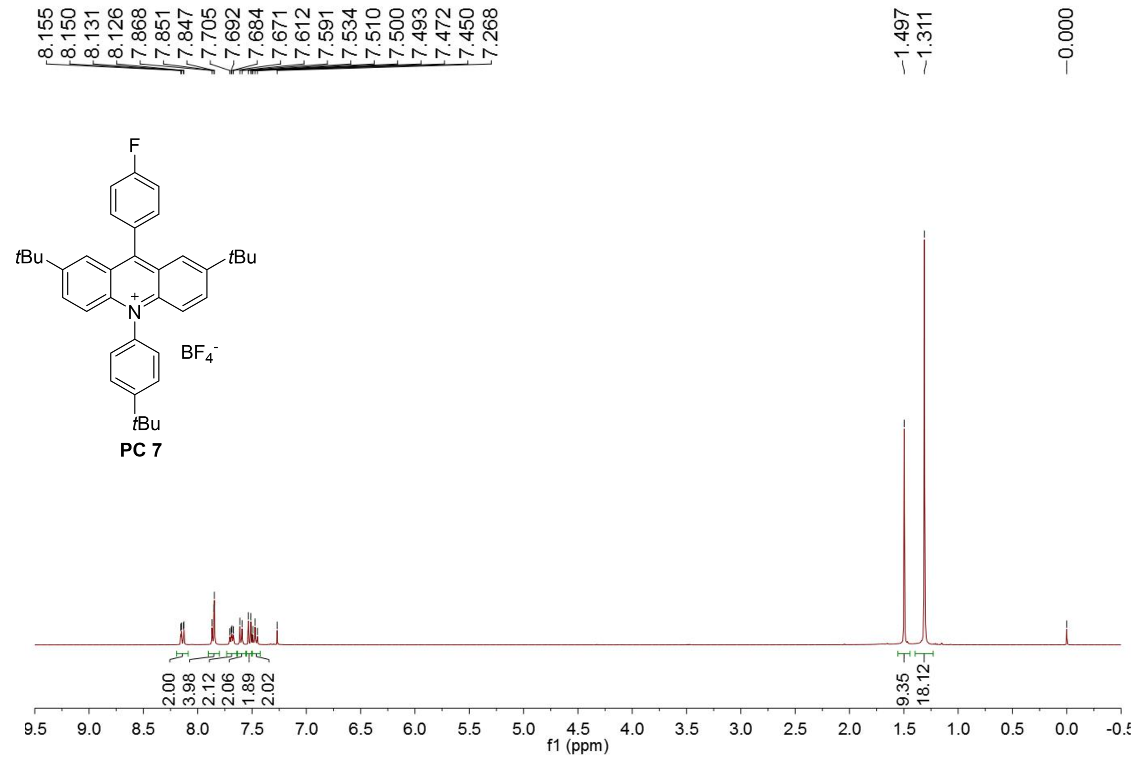
8.155
8.150
8.131
8.126
7.868
7.851
7.847
7.705
7.692
7.684
7.671
7.612
7.591
7.534
7.510
7.500
7.493
7.472
7.450
7.268
1.497
1.311
0.000
F
tBu
tBu
N
BF4-
tBu
PC 7
2.00
3.98
2.12
2.06
1.89
2.02
9.35
18.12
f1 (ppm)

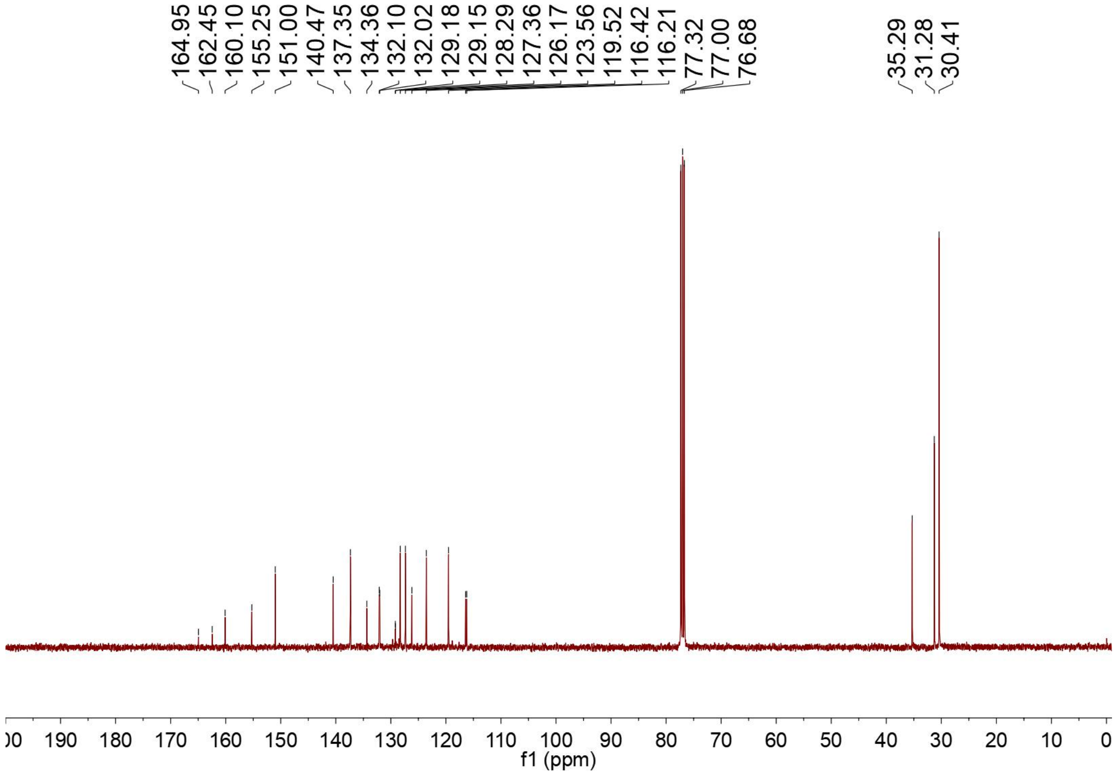
164.95
162.45
160.10
155.25
151.00
140.47
137.35
134.36
132.10
132.02
129.18
129.15
128.29
127.36
126.17
123.56
119.52
116.42
116.21
77.32
77.00
76.68
35.29
31.28
30.41
f1 (ppm)

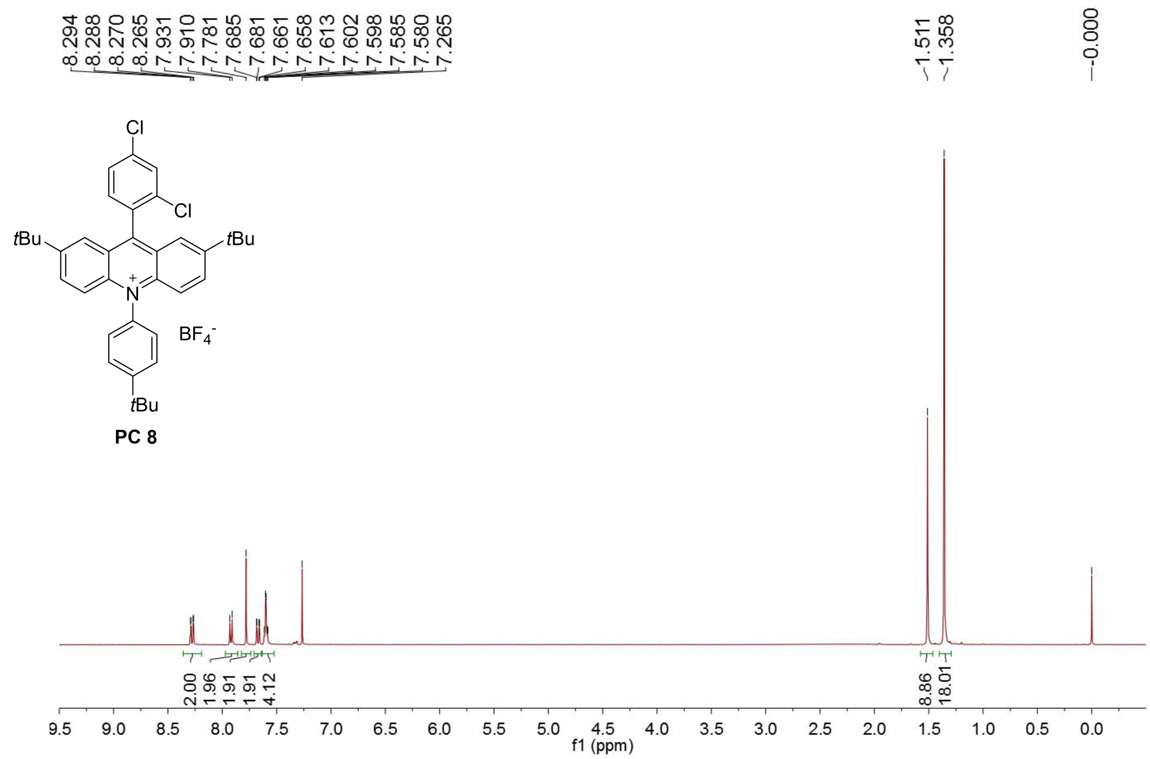
8.294
8.288
8.270
8.265
7.931
7.910
7.781
7.685
7.681
7.661
7.658
7.613
7.602
7.598
7.585
7.580
7.265
1.511
1.358
0.000
Cl
Cl
tBu
tBu
N
BF4-
tBu
PC 8
2.00
1.96
1.91
1.91
4.12
8.86
18.01
f1 (ppm)

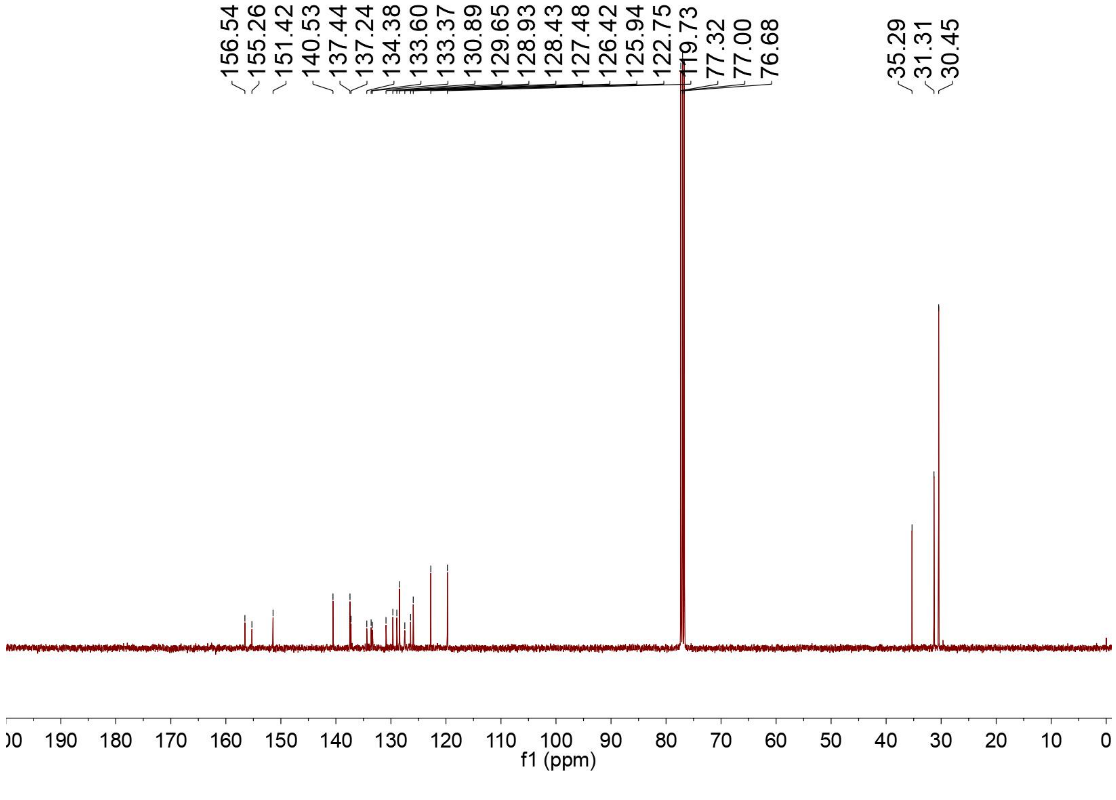
156.54
155.26
151.42
140.53
137.44
137.24
134.38
133.60
133.37
130.89
129.65
128.93
128.43
127.48
126.42
125.94
122.75
119.73
77.32
77.00
76.68
35.29
31.31
30.45
f1 (ppm)

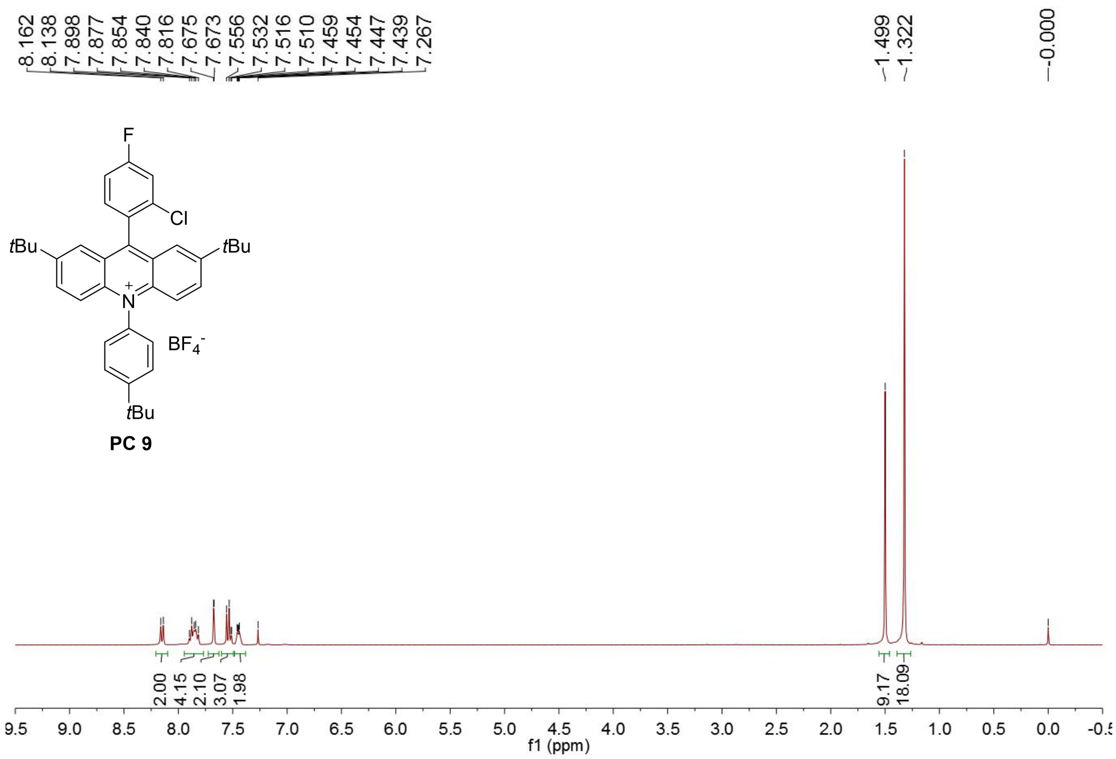
8.162
8.138
7.898
7.877
7.854
7.840
7.816
7.675
7.673
7.556
7.532
7.516
7.510
7.459
7.454
7.447
7.439
7.267
1.499
1.322
0.000
F
Cl
tBu
tBu
N
BF4-
tBu
PC 9
2.00
4.15
2.10
3.07
1.98
9.17
18.09
f1 (ppm)

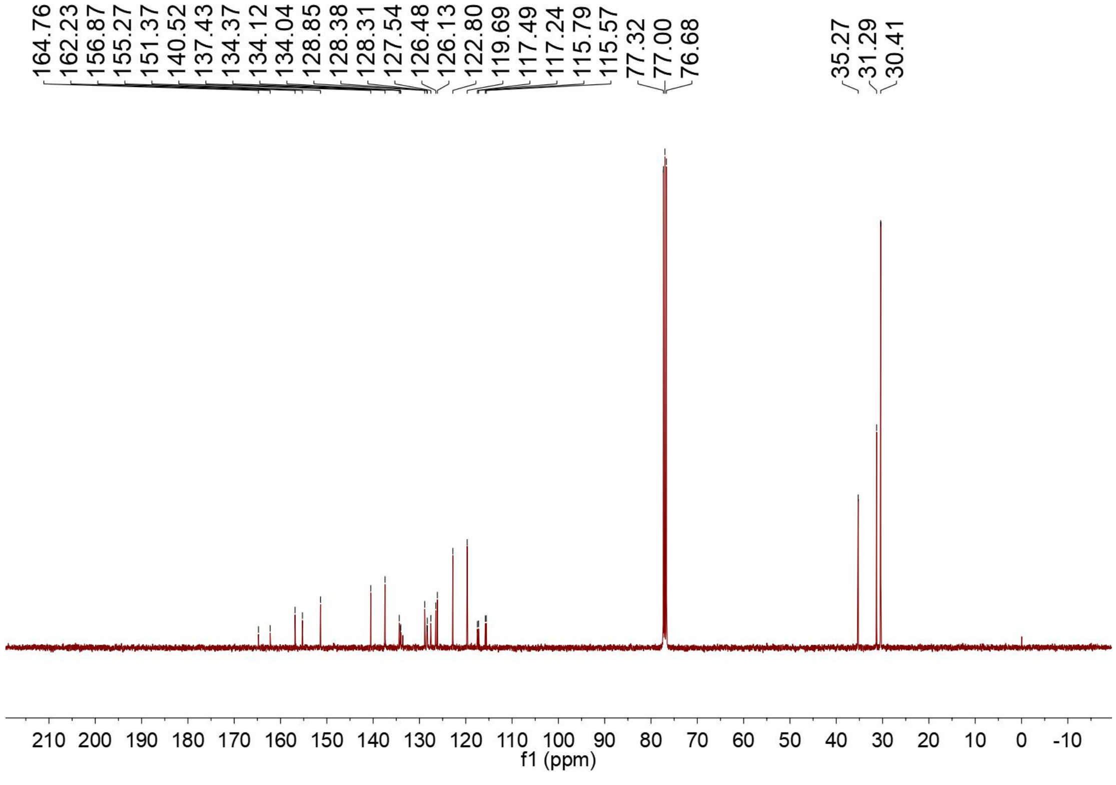
164.76
162.23
156.87
155.27
151.37
140.52
137.43
134.37
134.12
134.04
128.85
128.38
128.31
127.54
126.48
126.13
122.80
119.69
117.49
117.24
115.79
115.57
77.32
77.00
76.68
35.27
31.29
30.41
f1 (ppm)

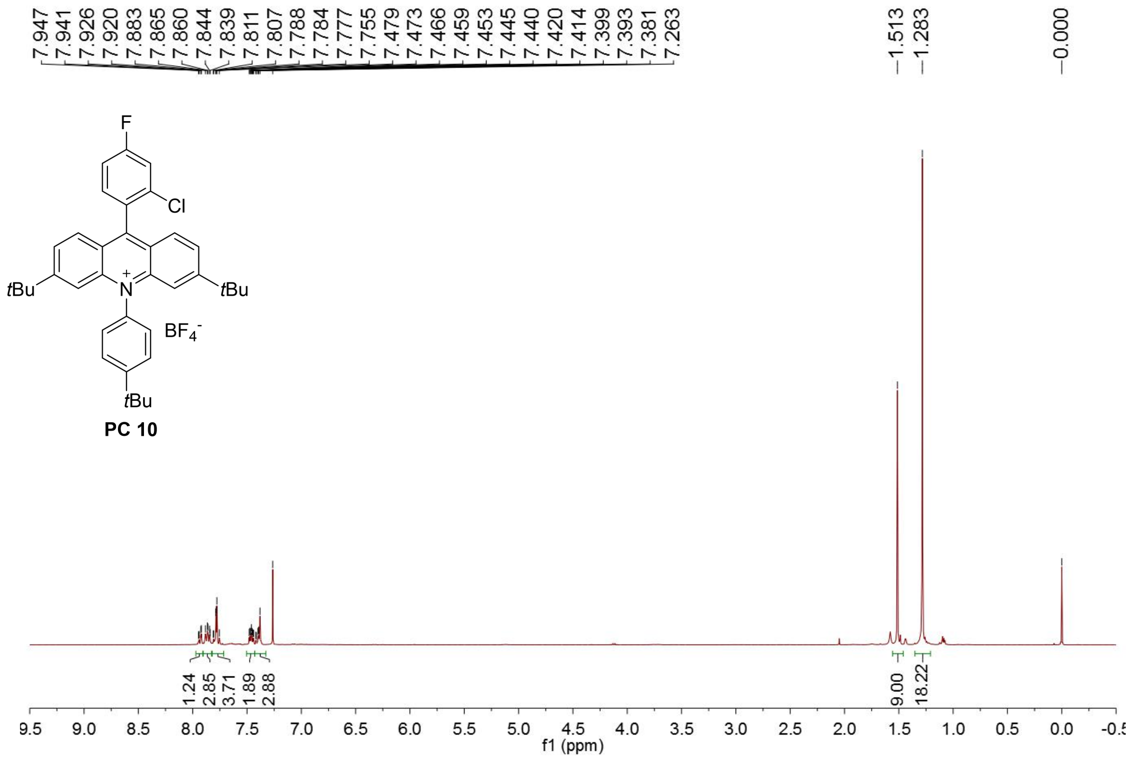
7.947 7.941 7.926 7.920 7.883 7.865 7.860 7.844 7.839 7.811 7.807 7.788 7.784 7.777 7.755 7.479 7.473 7.466 7.459 7.453 7.445 7.440 7.420 7.414 7.399 7.393 7.381 7.263
1.513
1.283
0.000
F
Cl
tBu
N
tBu
BF4-
tBu
PC 10
1.24 2.85 3.71 1.89 2.88
9.00 18.22
9.5 9.0 8.5 8.0 7.5 7.0 6.5 6.0 5.5 5.0 4.5 4.0 3.5 3.0 2.5 2.0 1.5 1.0 0.5 0.0 -0.5
f1 (ppm)

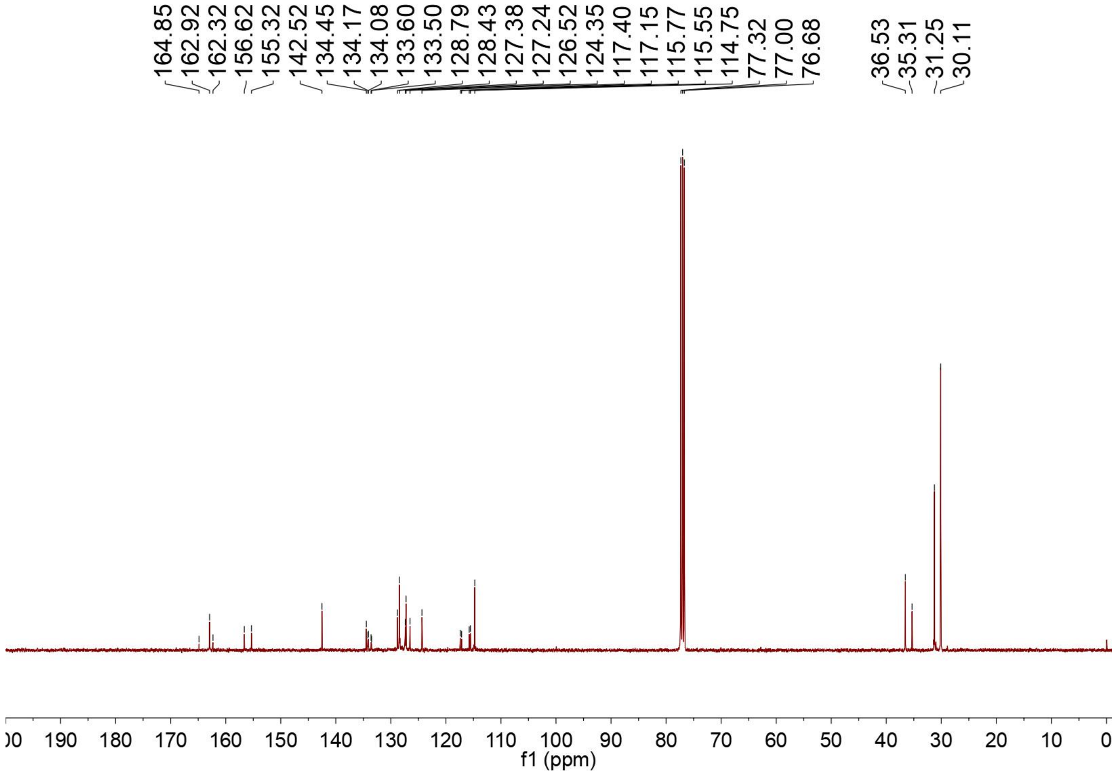
164.85 162.92 162.32 156.62 155.32 142.52 134.45 134.17 134.08 133.60 133.50 128.79 128.43 127.38 127.24 126.52 124.35 117.40 117.15 115.77 115.55 114.75 77.32 77.00 76.68
36.53 35.31 31.25 30.11
190 180 170 160 150 140 130 120 110 100 90 80 70 60 50 40 30 20 10 0
f1 (ppm)

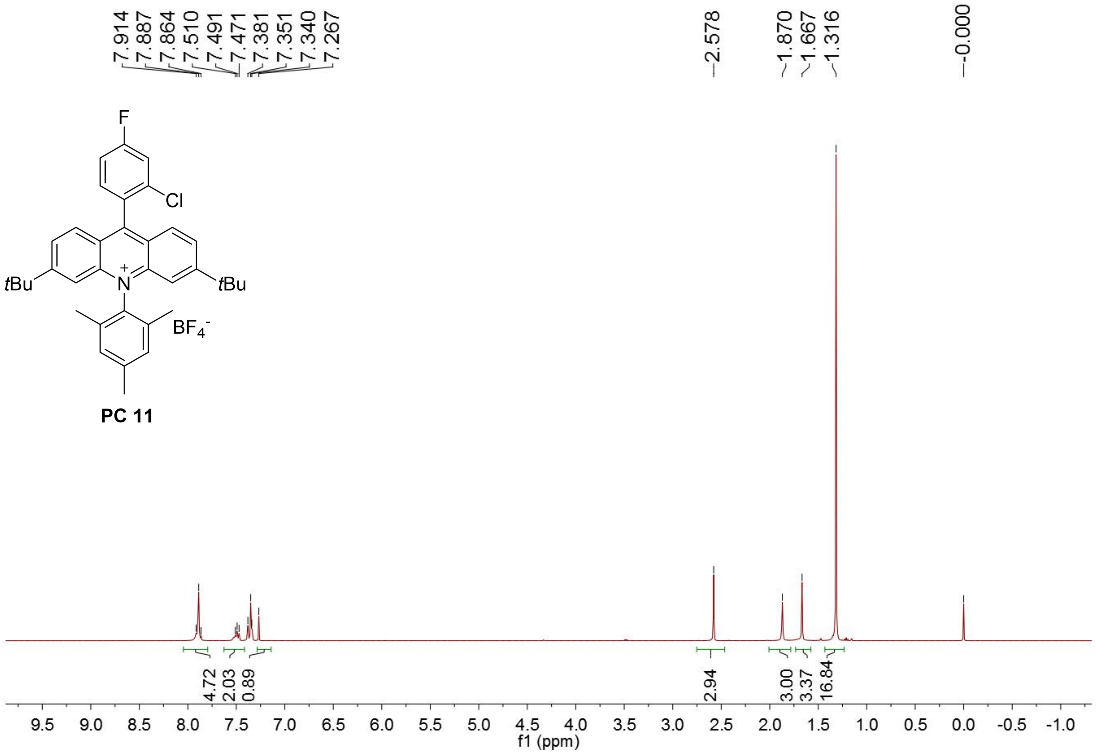
7.914
7.887
7.864
7.510
7.491
7.471
7.381
7.351
7.340
7.267
2.578
1.870
1.667
1.316
0.000
F
Cl
tBu
tBu
N
BF4-
PC 11
4.72
2.03
0.89
2.94
3.00
3.37
16.84
9.5 9.0 8.5 8.0 7.5 7.0 6.5 6.0 5.5 5.0 4.5 4.0 3.5 3.0 2.5 2.0 1.5 1.0 0.5 0.0 -0.5 -1.0
f1 (ppm)

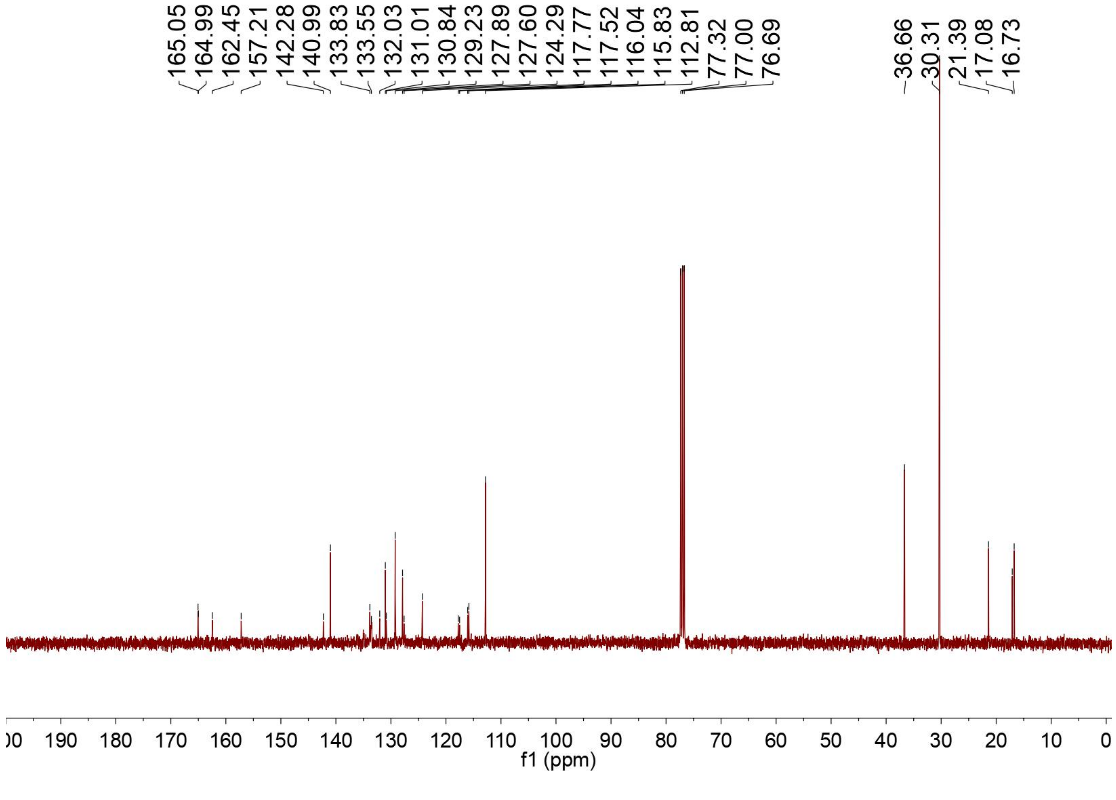
165.05
164.99
162.45
157.21
142.28
140.99
133.83
133.55
132.03
131.01
130.84
129.23
127.89
127.60
124.29
117.77
117.52
116.04
115.83
112.81
77.32
77.00
76.69
36.66
30.31
21.39
17.08
16.73
190 180 170 160 150 140 130 120 110 100 90 80 70 60 50 40 30 20 10 0
f1 (ppm)

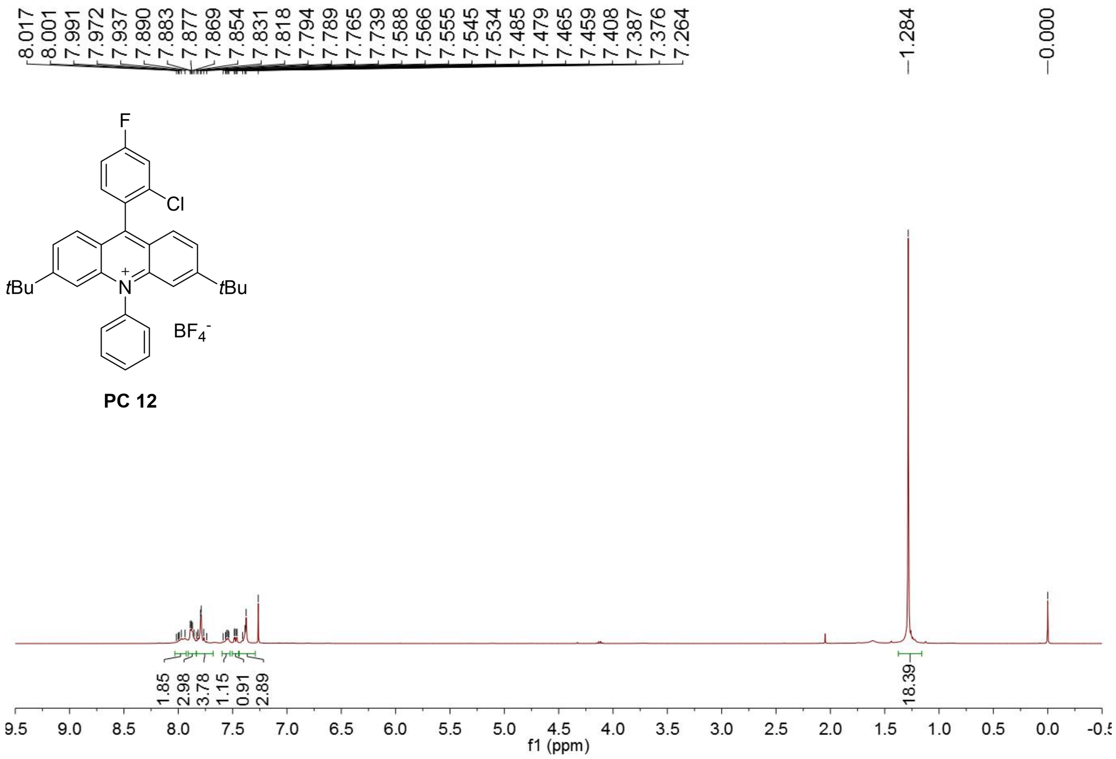
8.017
8.001
7.991
7.972
7.937
7.890
7.883
7.877
7.869
7.854
7.831
7.818
7.794
7.789
7.765
7.739
7.588
7.566
7.555
7.545
7.534
7.485
7.479
7.465
7.459
7.408
7.387
7.376
7.264
1.284
0.000
F
Cl
tBu
tBu
N
BF_4^-
PC 12
1.85
2.98
3.78
1.15
0.91
2.89
18.39
f1 (ppm)

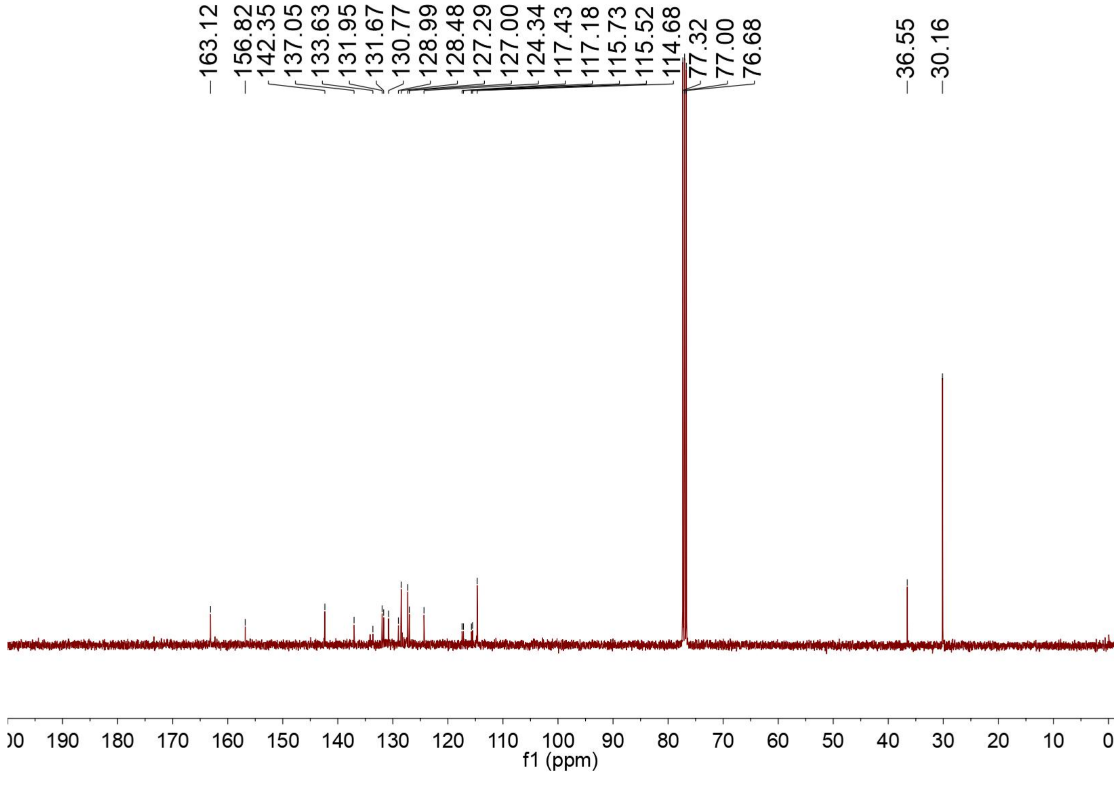
163.12
156.82
142.35
137.05
133.63
131.95
131.67
130.77
128.99
128.48
127.29
127.00
124.34
117.43
117.18
115.73
115.52
114.68
77.32
77.00
76.68
36.55
30.16
f1 (ppm)

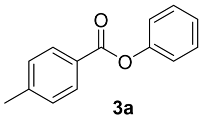

3a

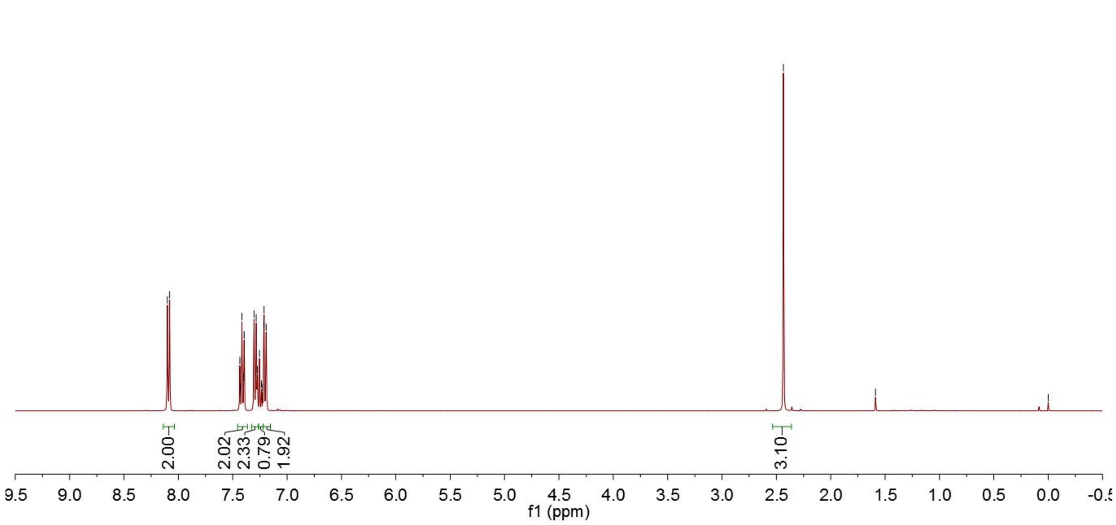

8.101
8.081
7.435
7.430
7.415
7.399
7.395
7.304
7.284
7.273
7.254
7.235
7.225
7.214
7.212
7.193
2.435
1.588
0.000
2.00
2.02
2.33
0.79
1.92
3.10
9.5 9.0 8.5 8.0 7.5 7.0 6.5 6.0 5.5 5.0 4.5 4.0 3.5 3.0 2.5 2.0 1.5 1.0 0.5 0.0
f1 (ppm)

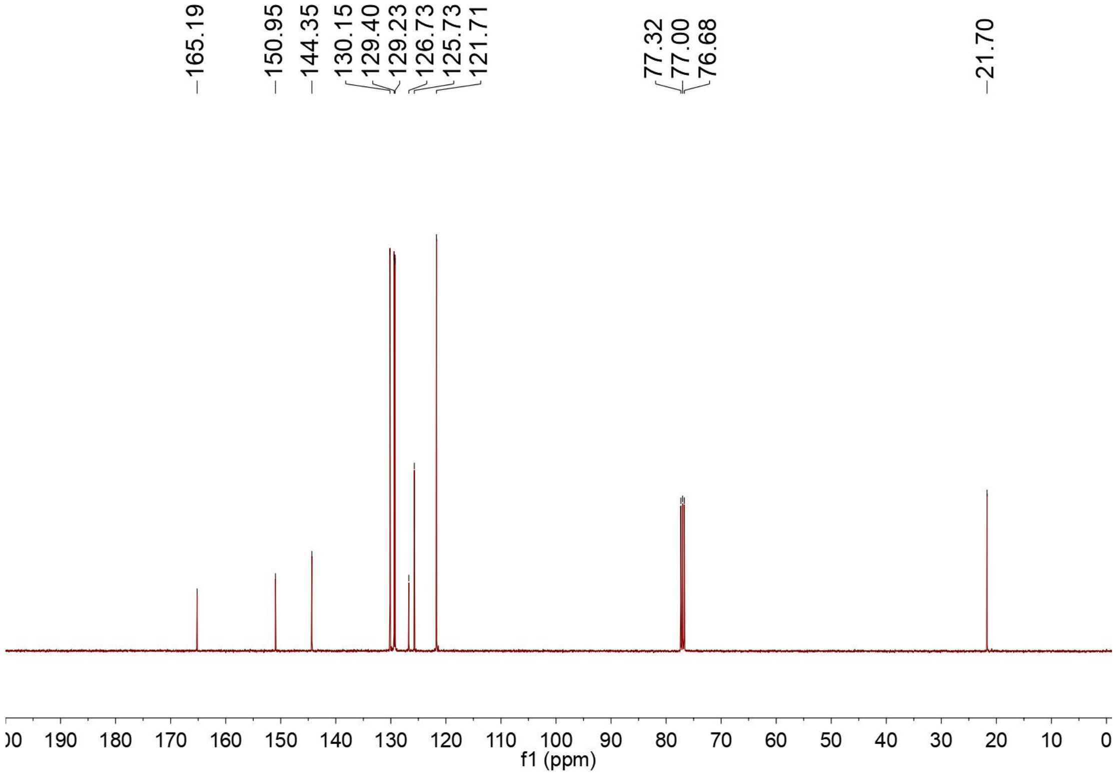

165.19
150.95
144.35
130.15
129.40
129.23
126.73
125.73
121.71
77.32
77.00
76.68
21.70
190 180 170 160 150 140 130 120 110 100 90 80 70 60 50 40 30 20 10 0
f1 (ppm)

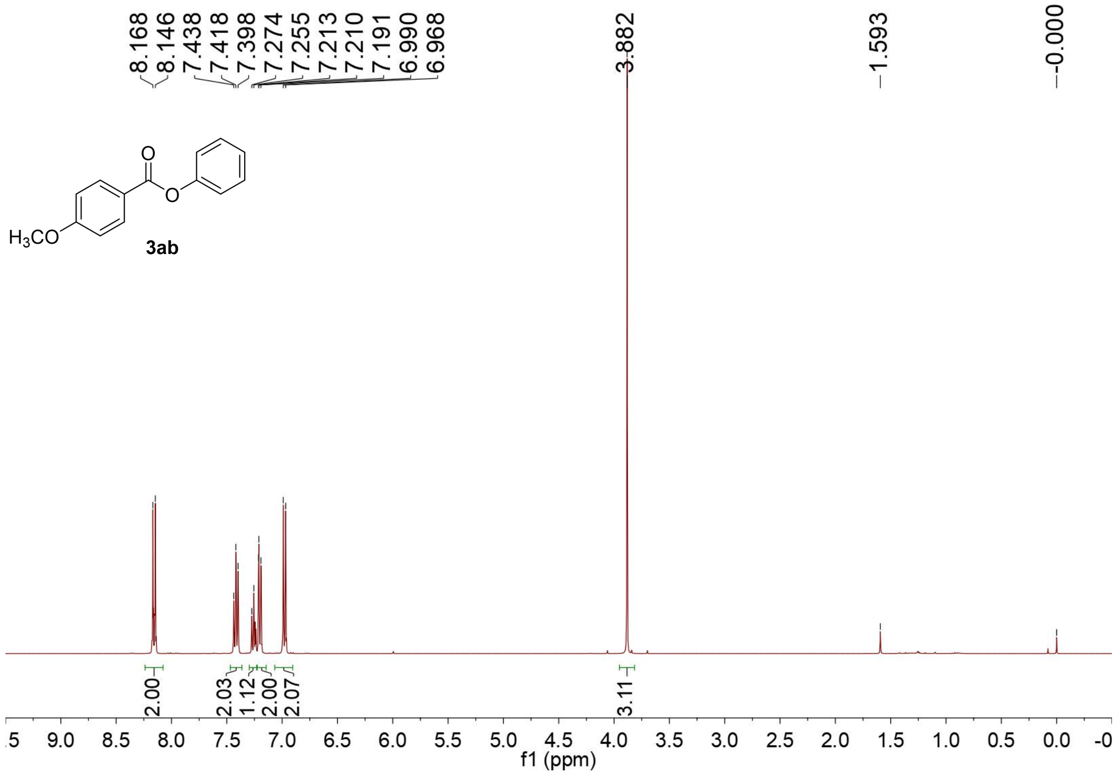
8.168
8.146
7.438
7.418
7.398
7.274
7.255
7.213
7.210
7.191
6.990
6.968
3.882
1.593
0.000
H_3CO
O
O
3ab
2.00
2.03
1.12
2.00
2.07
3.11
f1 (ppm)

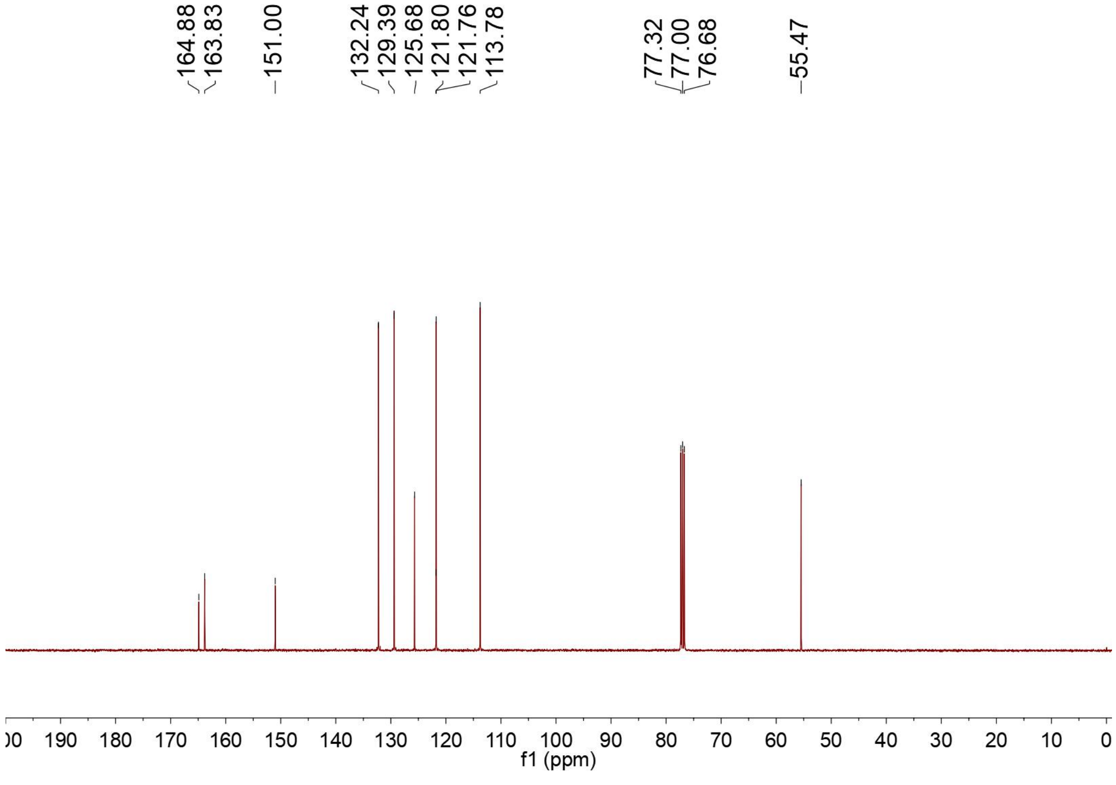
164.88
163.83
151.00
132.24
129.39
125.68
121.80
121.76
113.78
77.32
77.00
76.68
55.47
f1 (ppm)

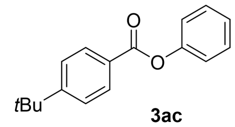
O
O
tBu

3ac

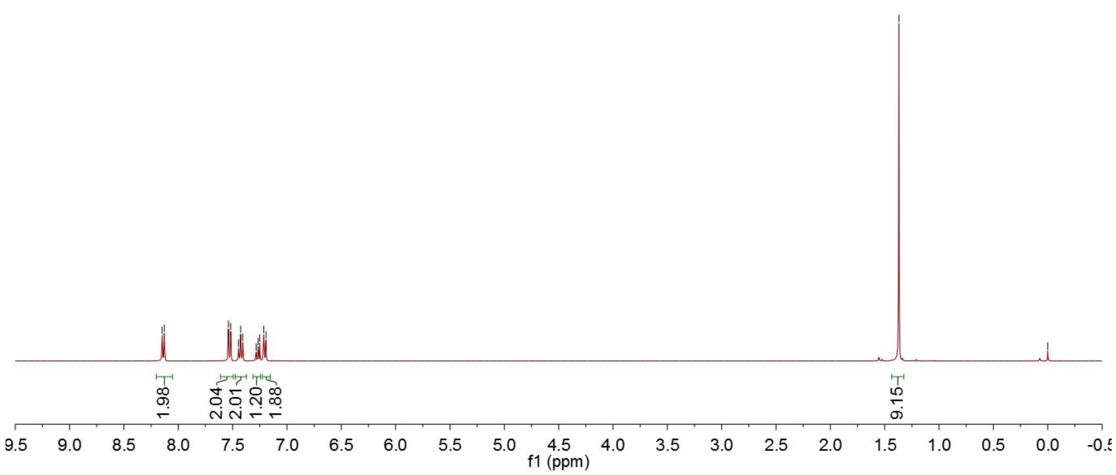
8.149
8.128
7.539
7.518
7.446
7.426
7.407
7.283
7.265
7.251
7.214
7.193
1.369
-0.000
1.98
2.04
2.01
1.20
1.88
9.15
9.5 9.0 8.5 8.0 7.5 7.0 6.5 6.0 5.5 5.0 4.5 4.0 3.5 3.0 2.5 2.0 1.5 1.0 0.5 0.0
f1 (ppm)

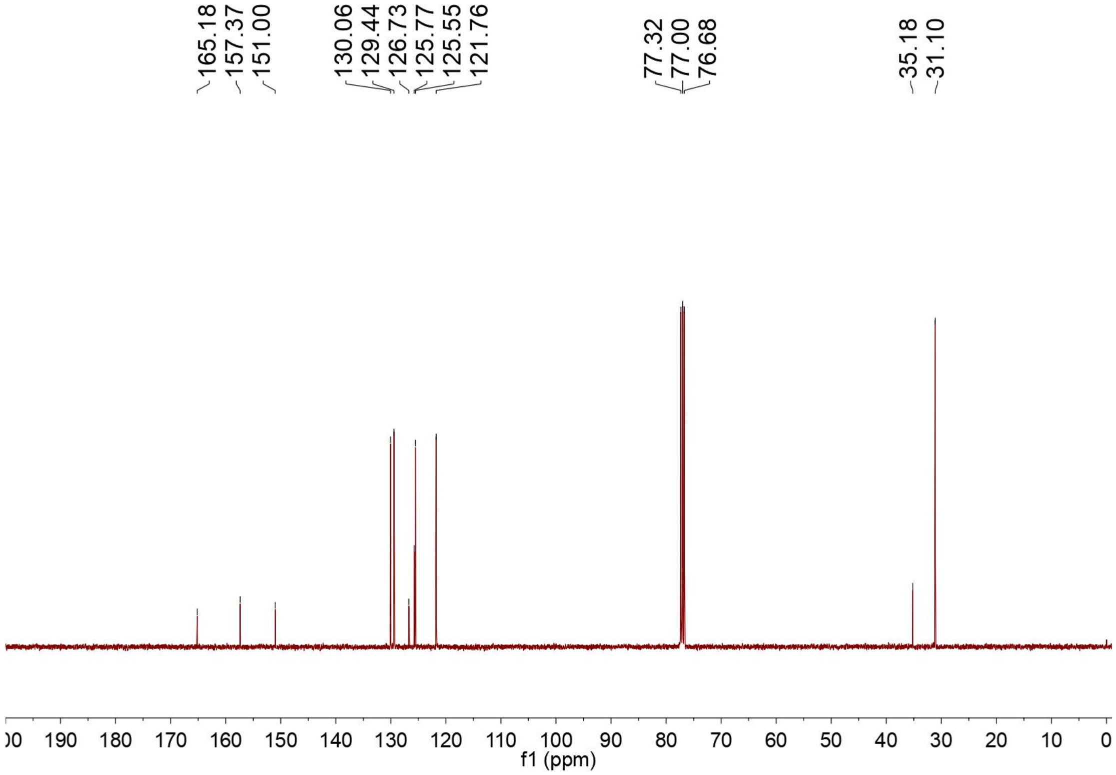
165.18
157.37
151.00
130.06
129.44
126.73
125.77
125.55
121.76
77.32
77.00
76.68
35.18
31.10
190 180 170 160 150 140 130 120 110 100 90 80 70 60 50 40 30 20 10 0
f1 (ppm)

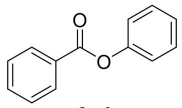

3ad

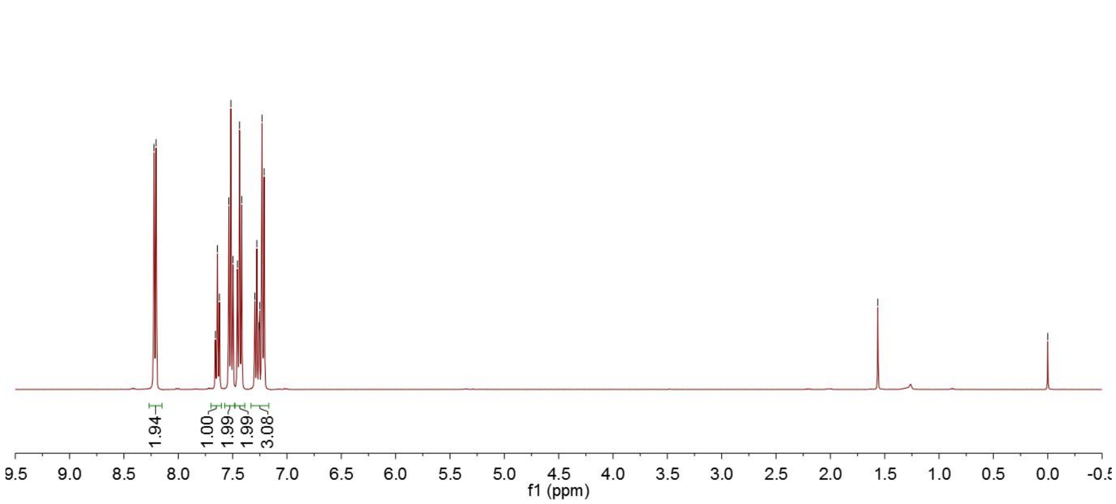
8.223
8.205
7.658
7.640
7.621
7.535
7.516
7.497
7.456
7.436
7.417
7.296
7.277
7.258
7.250
7.229
7.210
1.564
0.000
1.94
1.00
1.99
1.99
3.08
9.5 9.0 8.5 8.0 7.5 7.0 6.5 6.0 5.5 5.0 4.5 4.0 3.5 3.0 2.5 2.0 1.5 1.0 0.5 0.0 -0.5
f1 (ppm)

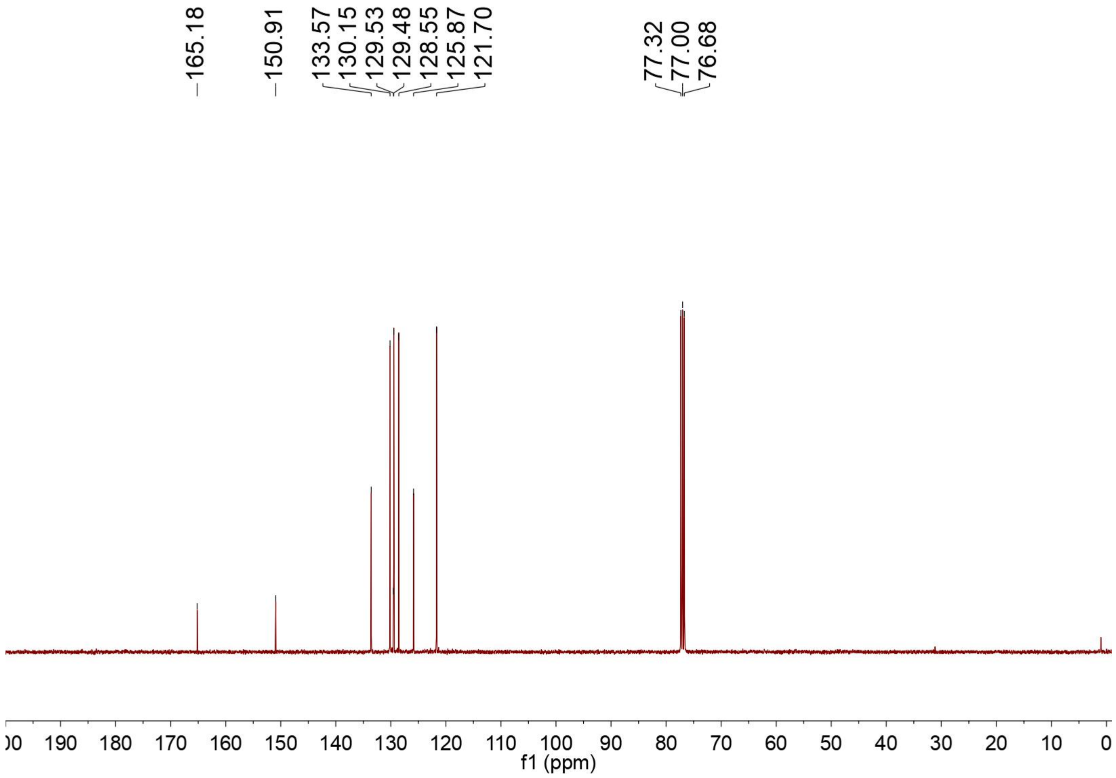
165.18
150.91
133.57
130.15
129.53
129.48
128.55
125.87
121.70
77.32
77.00
76.68
190 180 170 160 150 140 130 120 110 100 90 80 70 60 50 40 30 20 10 0
f1 (ppm)

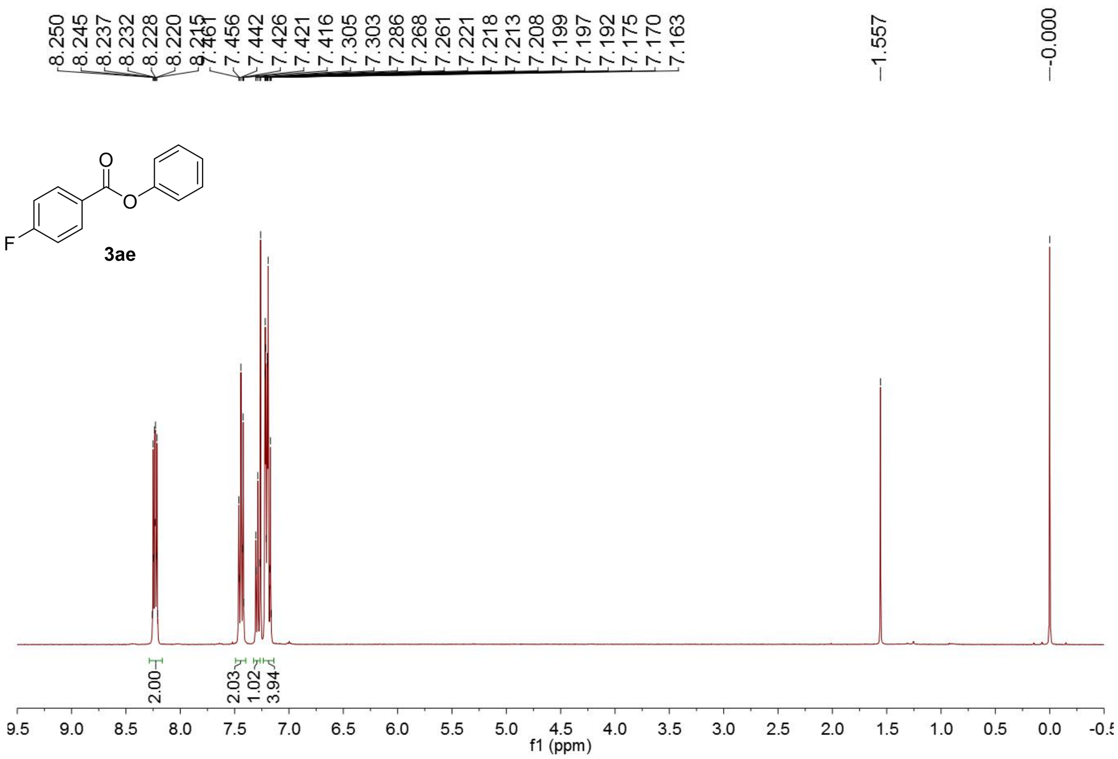

8.250
8.245
8.237
8.232
8.228
8.220
8.215
7.461
7.456
7.442
7.426
7.421
7.416
7.305
7.303
7.286
7.268
7.261
7.221
7.218
7.213
7.208
7.199
7.197
7.192
7.175
7.170
7.163
1.557
0.000
O
O
F
3ae
2.00
2.03
1.02
3.94
9.5 9.0 8.5 8.0 7.5 7.0 6.5 6.0 5.5 5.0 4.5 4.0 3.5 3.0 2.5 2.0 1.5 1.0 0.5 0.0
f1 (ppm)

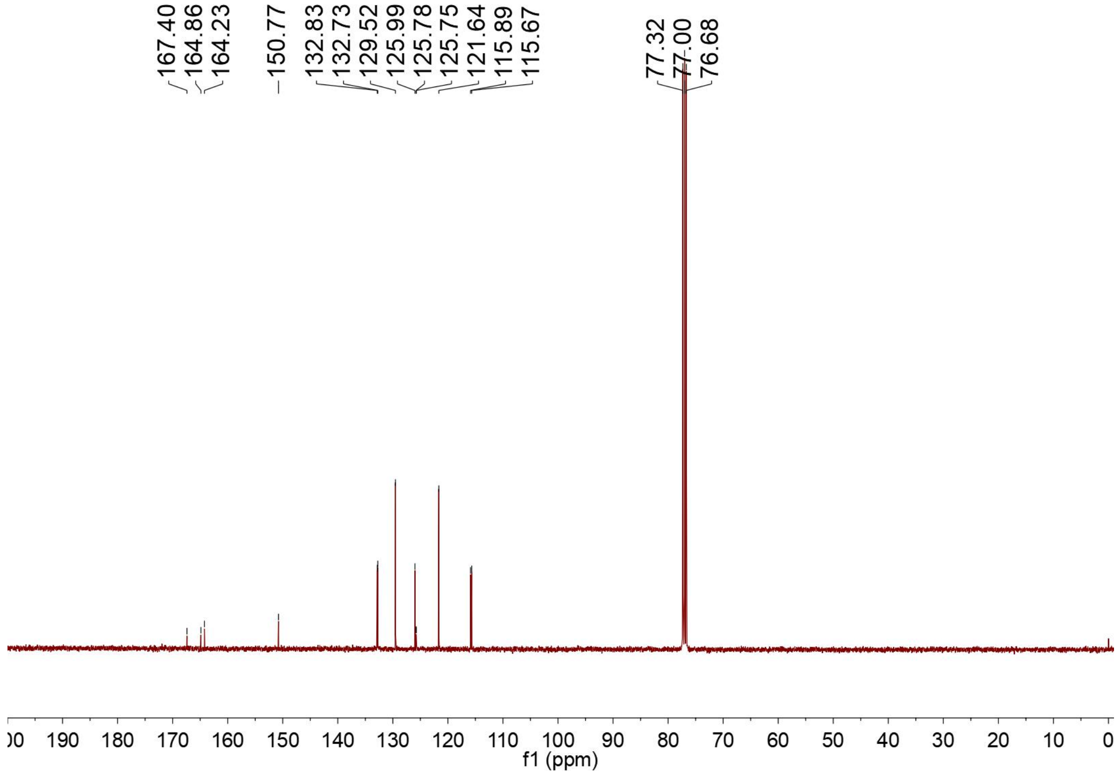

167.40
164.86
164.23
150.77
132.83
132.73
129.52
125.99
125.78
125.75
121.64
115.89
115.67
77.32
77.00
76.68
190 180 170 160 150 140 130 120 110 100 90 80 70 60 50 40 30 20 10 0
f1 (ppm)

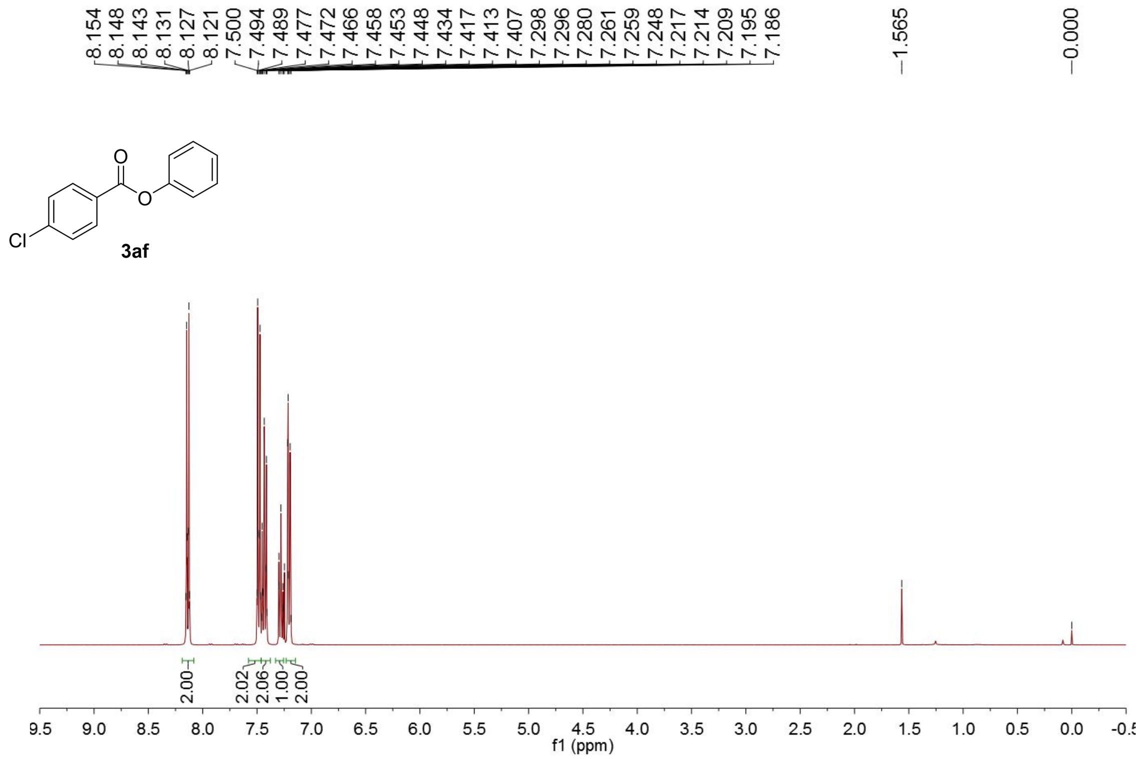
8.154
8.148
8.143
8.131
8.127
8.121
7.500
7.494
7.489
7.477
7.472
7.466
7.458
7.453
7.448
7.434
7.417
7.413
7.407
7.298
7.296
7.280
7.261
7.259
7.248
7.217
7.214
7.209
7.195
7.186
1.565
0.000
O
O
Cl
3af
2.00
2.02
2.06
1.00
2.00
9.5 9.0 8.5 8.0 7.5 7.0 6.5 6.0 5.5 5.0 4.5 4.0 3.5 3.0 2.5 2.0 1.5 1.0 0.5 0.0
f1 (ppm)

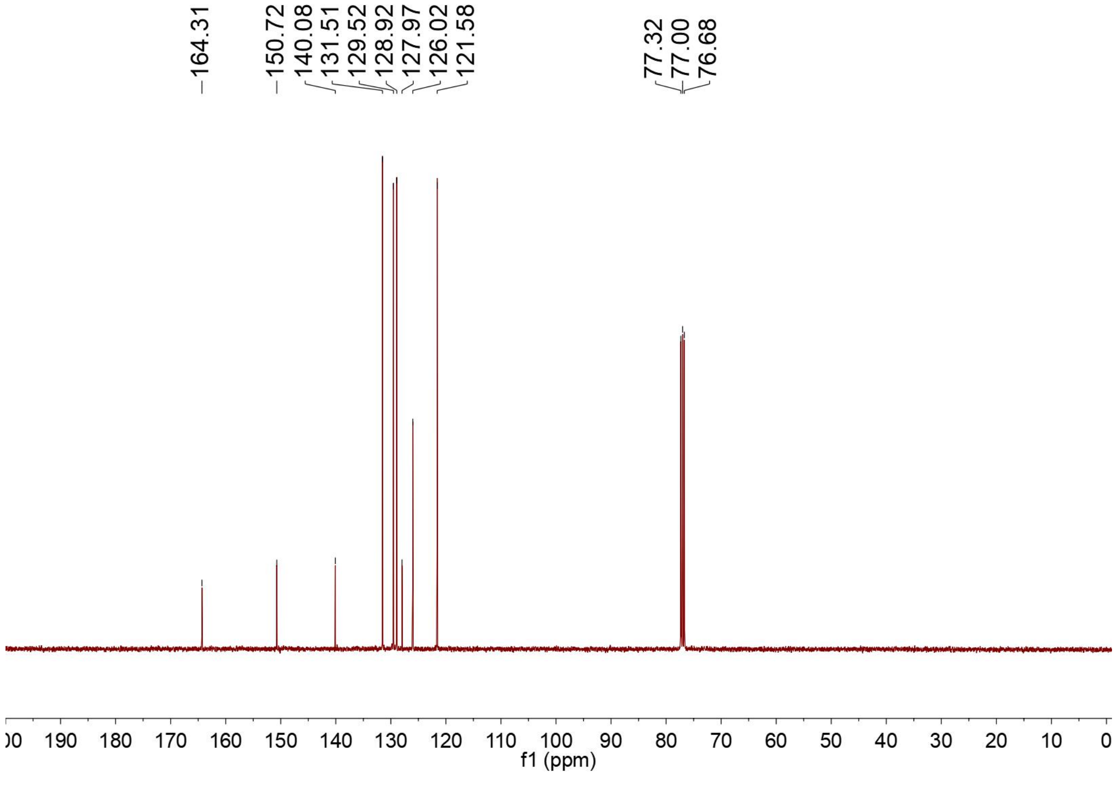
164.31
150.72
140.08
131.51
129.52
128.92
127.97
126.02
121.58
77.32
77.00
76.68
190 180 170 160 150 140 130 120 110 100 90 80 70 60 50 40 30 20 10 0
f1 (ppm)

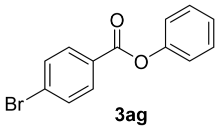
O
O
Br

3ag

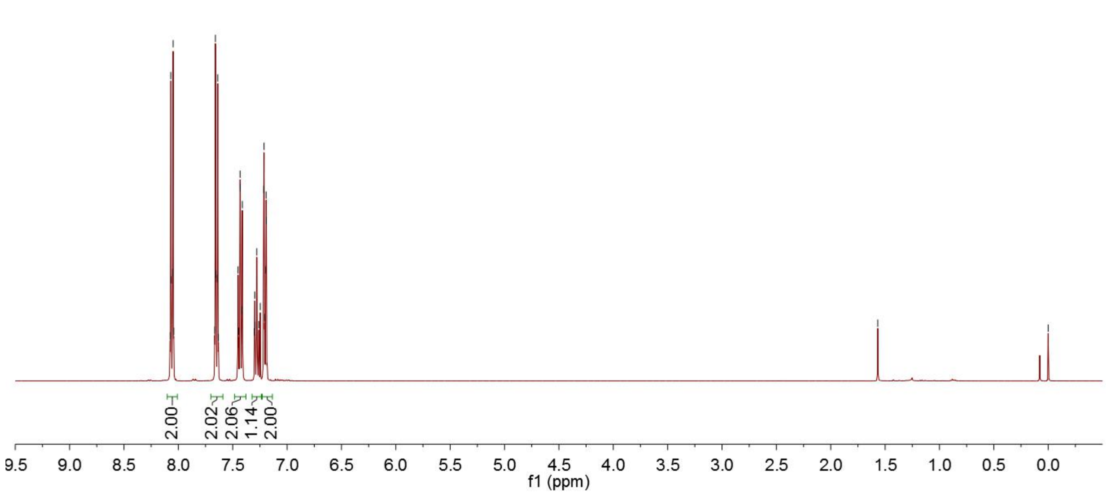
8.075
8.069
8.065
8.053
8.048
8.042
7.665
7.659
7.655
7.642
7.638
7.632
7.451
7.446
7.432
7.431
7.416
7.411
7.300
7.297
7.295
7.279
7.260
7.247
7.215
7.212
7.207
7.196
7.193
7.191
1.568
0.000
2.00
2.02
2.06
1.14
2.00
9.5 9.0 8.5 8.0 7.5 7.0 6.5 6.0 5.5 5.0 4.5 4.0 3.5 3.0 2.5 2.0 1.5 1.0 0.5 0.0
f1 (ppm)

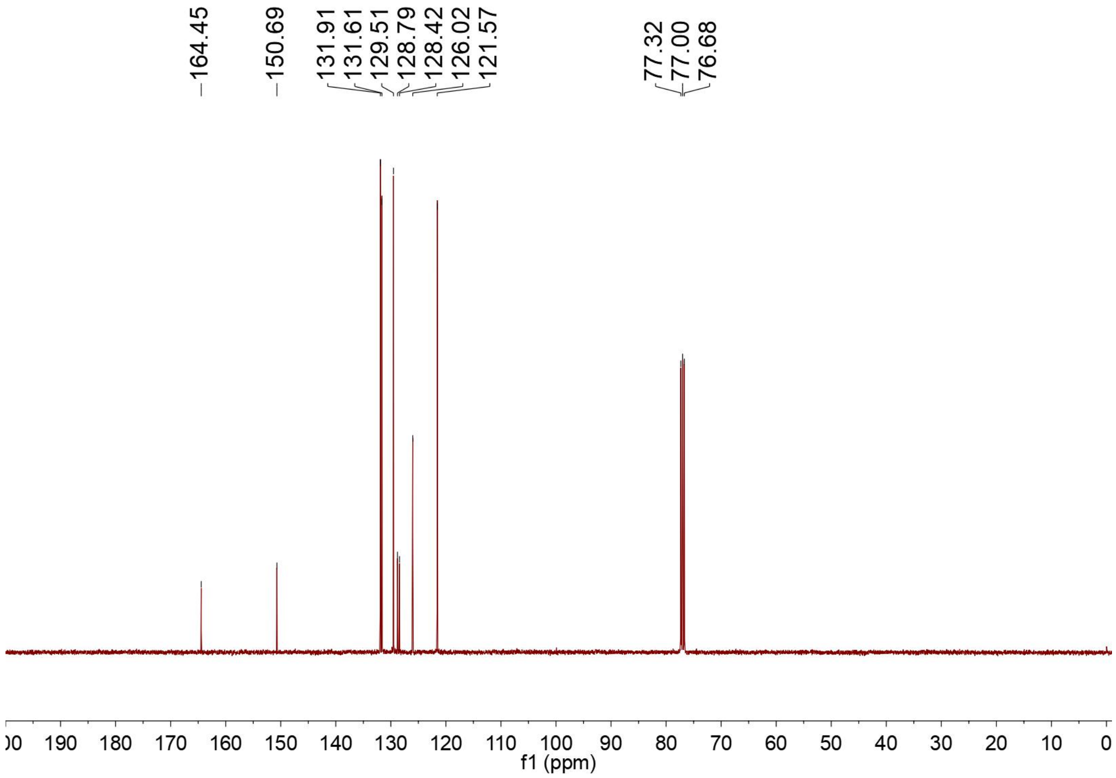
164.45
150.69
131.91
131.61
129.51
128.79
128.42
126.02
121.57
77.32
77.00
76.68
190 180 170 160 150 140 130 120 110 100 90 80 70 60 50 40 30 20 10 0
f1 (ppm)

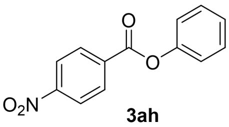

3ah

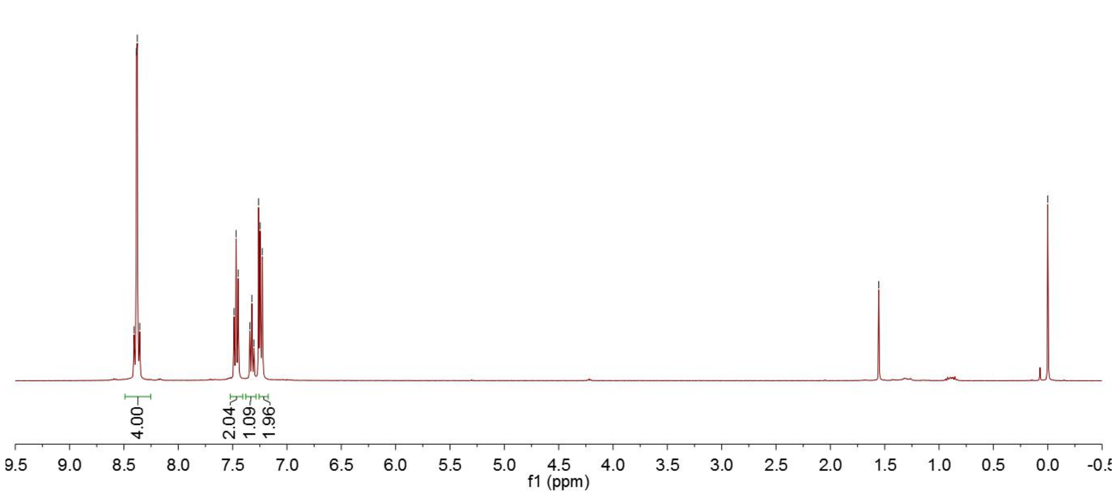
8.407
8.385
8.377
8.354
7.487
7.468
7.448
7.341
7.323
7.304
7.261
7.247
7.227
1.555
0.000
4.00
2.04
1.09
1.96
f1 (ppm)

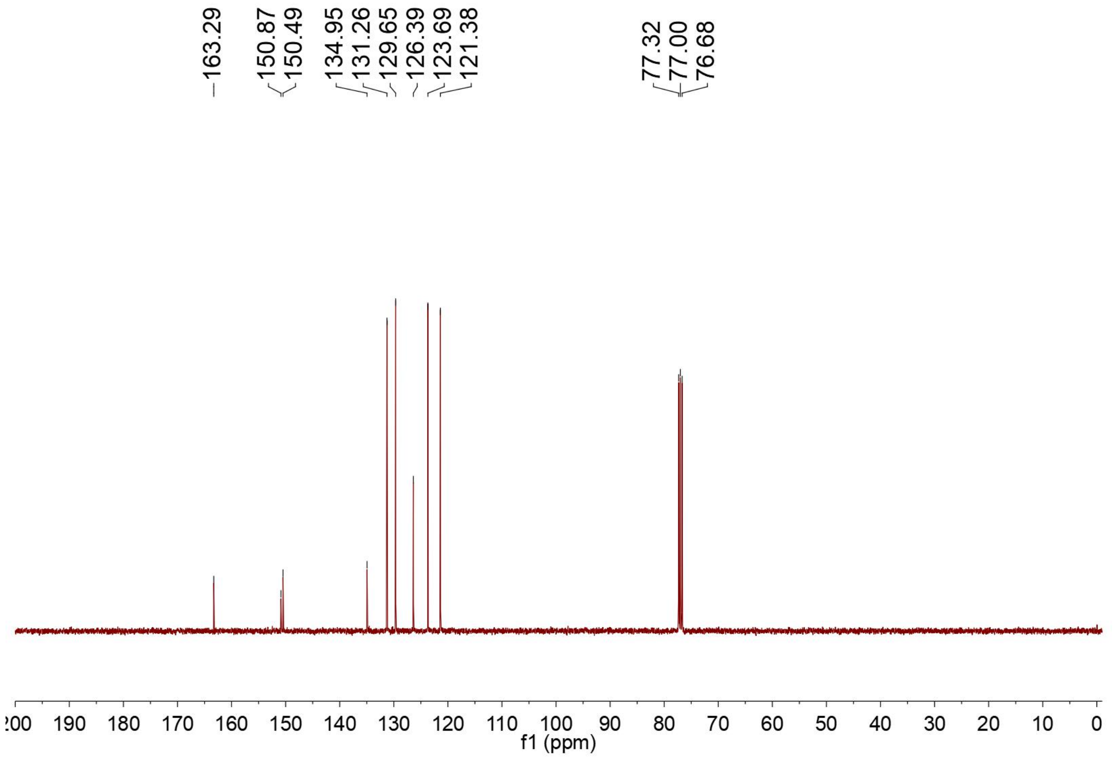
163.29
150.87
150.49
134.95
131.26
129.65
126.39
123.69
121.38
77.32
77.00
76.68
f1 (ppm)

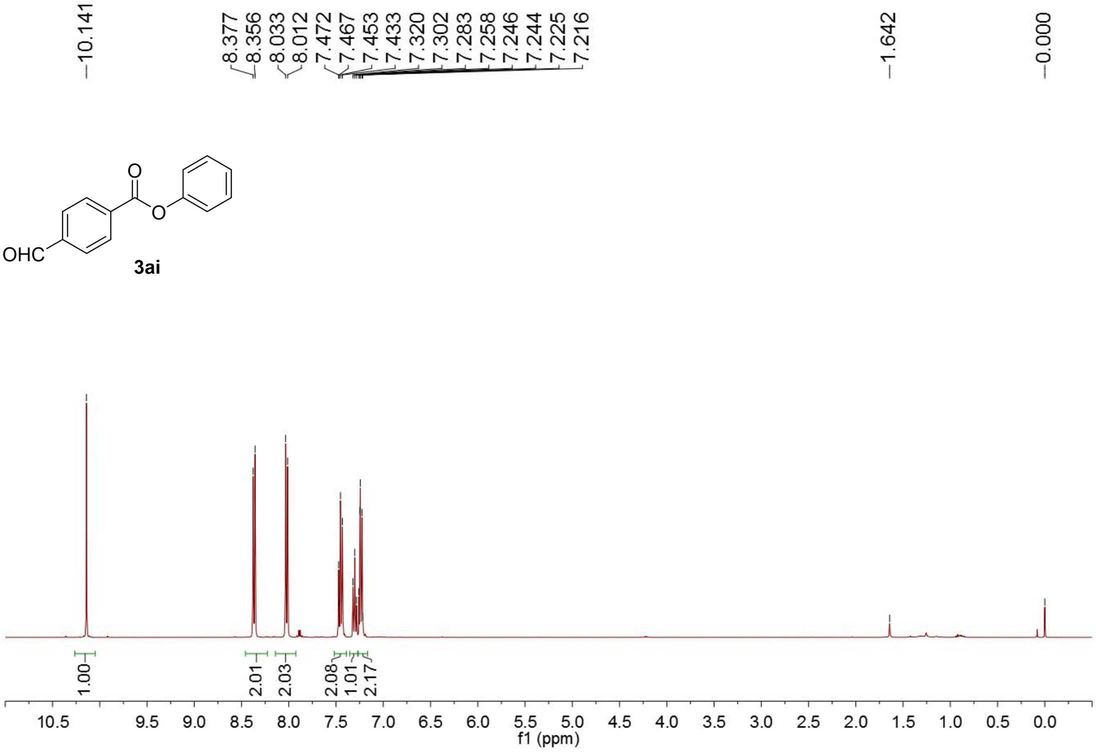
10.141
8.377
8.356
8.033
8.012
7.472
7.467
7.453
7.433
7.320
7.302
7.283
7.258
7.246
7.244
7.225
7.216
1.642
0.000
OHC
O
O
3ai
1.00
2.01
2.03
2.08
1.01
2.17
10.5 9.5 9.0 8.5 8.0 7.5 7.0 6.5 6.0 5.5 5.0 4.5 4.0 3.5 3.0 2.5 2.0 1.5 1.0 0.5 0.0
f1 (ppm)

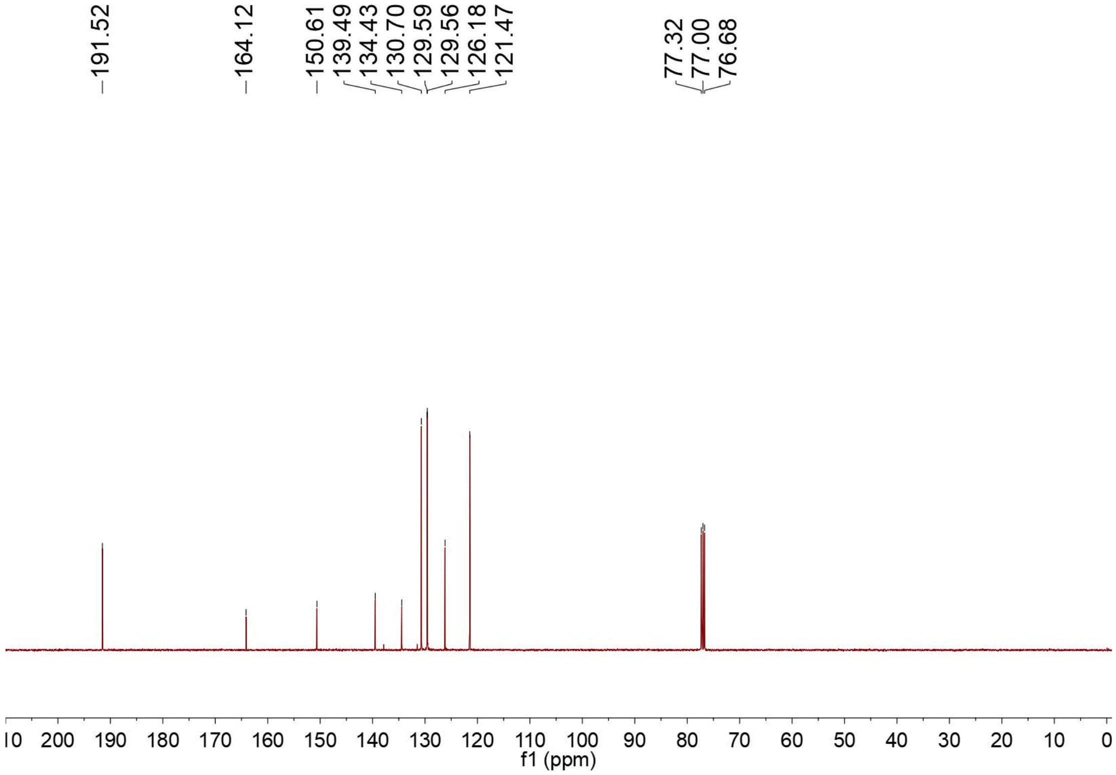
191.52
164.12
150.61
139.49
134.43
130.70
129.59
129.56
126.18
121.47
77.32
77.00
76.68
10 200 190 180 170 160 150 140 130 120 110 100 90 80 70 60 50 40 30 20 10 0
f1 (ppm)

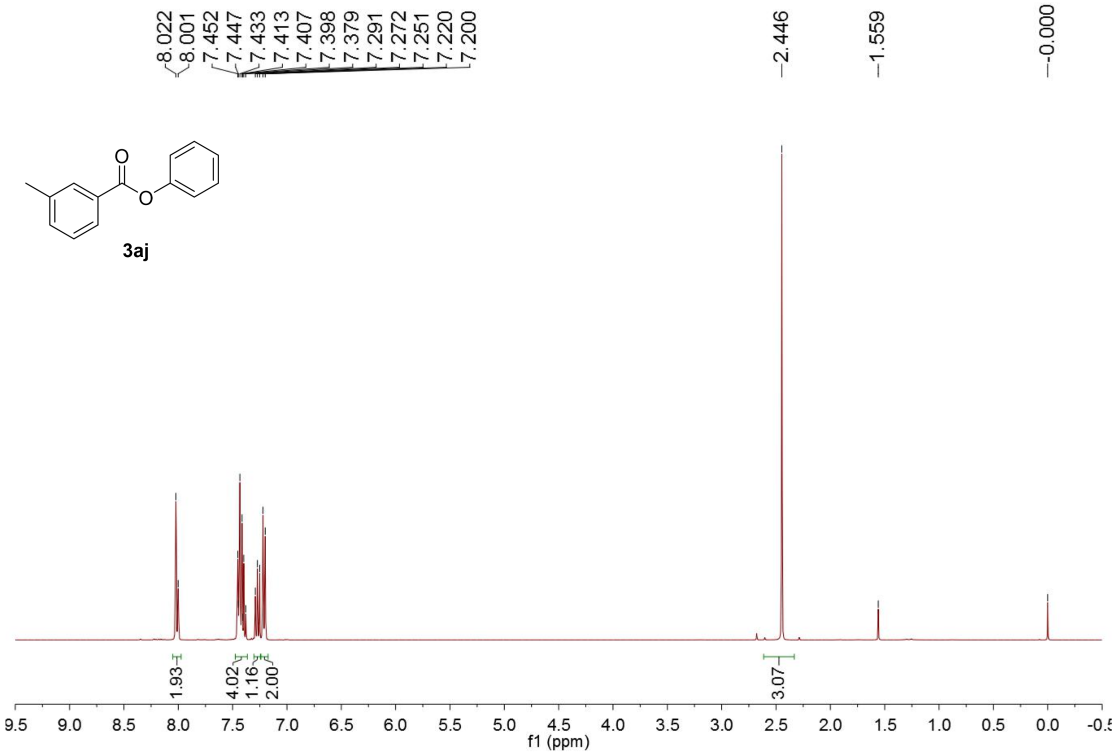
8.022
8.001
7.452
7.447
7.433
7.413
7.407
7.398
7.379
7.291
7.272
7.251
7.220
7.200
2.446
1.559
-0.000
3aj
1.93
4.02
1.16
2.00
3.07
f1 (ppm)

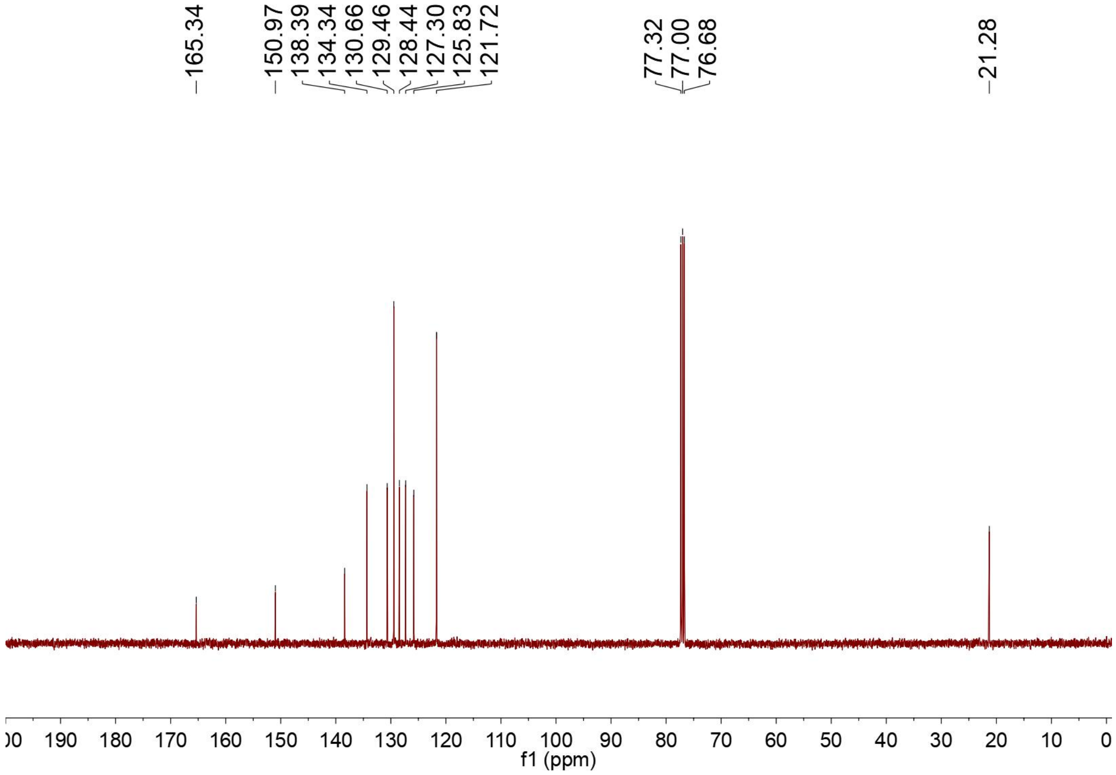
165.34
150.97
138.39
134.34
130.66
129.46
128.44
127.30
125.83
121.72
77.32
77.00
76.68
21.28
f1 (ppm)

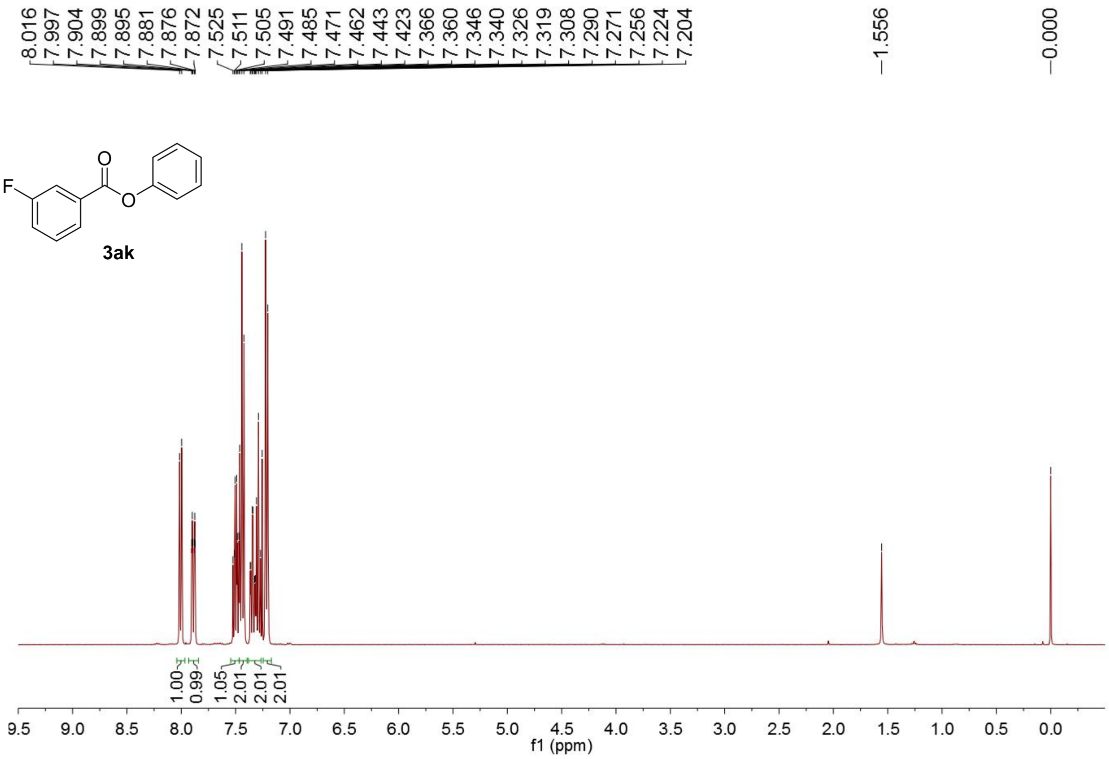

3ak
8.016
7.997
7.904
7.899
7.895
7.881
7.876
7.872
7.525
7.511
7.505
7.491
7.485
7.471
7.462
7.443
7.423
7.366
7.360
7.346
7.340
7.326
7.319
7.308
7.290
7.271
7.256
7.224
7.204
1.556
0.000
1.00
0.99
1.05
2.01
2.01
2.01
9.5 9.0 8.5 8.0 7.5 7.0 6.5 6.0 5.5 5.0 4.5 4.0 3.5 3.0 2.5 2.0 1.5 1.0 0.5 0.0
f1 (ppm)

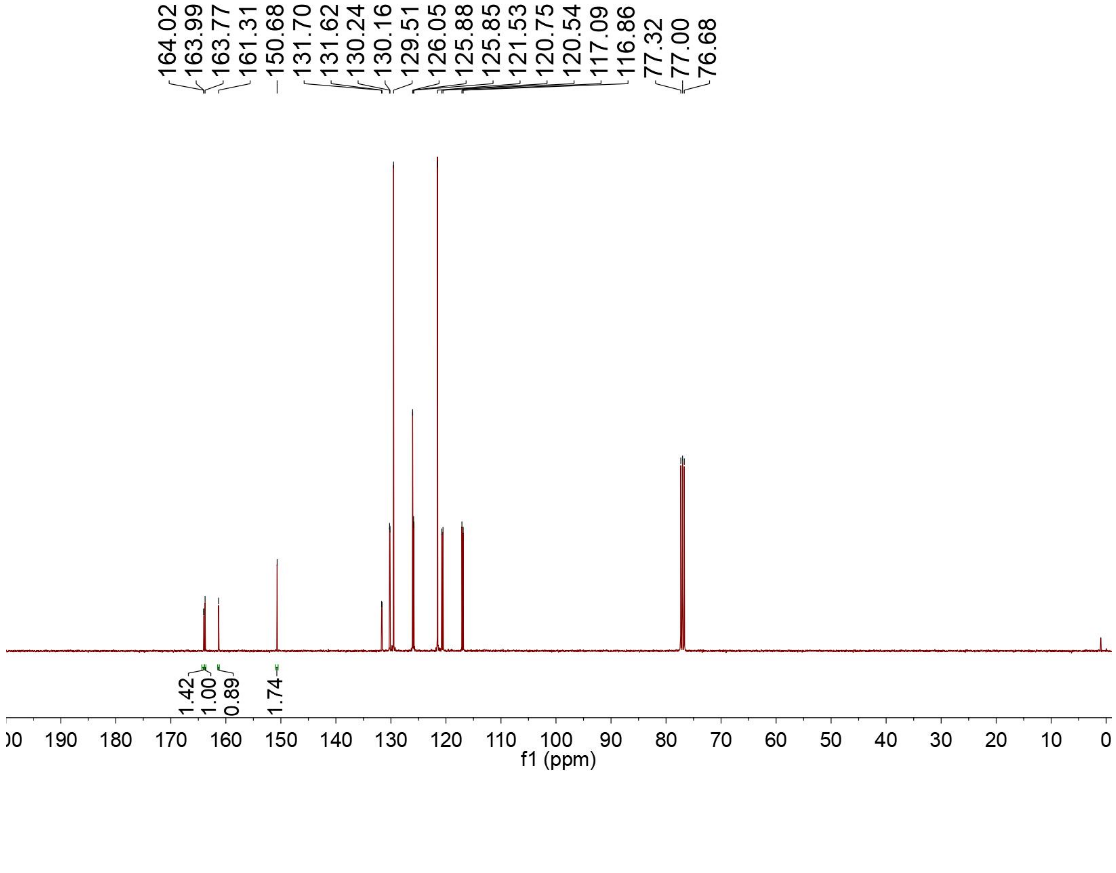
164.02
163.99
163.77
161.31
150.68
131.70
131.62
130.24
130.16
129.51
126.05
125.88
125.85
121.53
120.75
120.54
117.09
116.86
77.32
77.00
76.68
1.42
1.00
0.89
1.74
190 180 170 160 150 140 130 120 110 100 90 80 70 60 50 40 30 20 10 0
f1 (ppm)

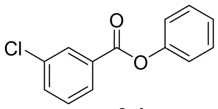

3al

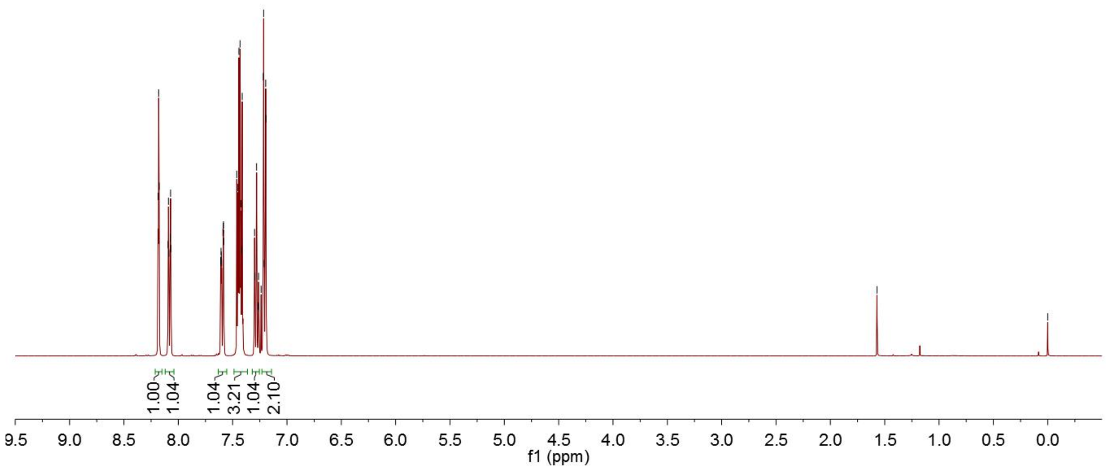

8.185
8.180
8.176
8.094
8.091
8.074
8.071
7.609
7.607
7.604
7.601
7.589
7.587
7.584
7.581
7.462
7.451
7.442
7.432
7.422
7.416
7.411
7.298
7.295
7.280
7.263
7.261
7.258
7.238
7.217
7.215
7.209
7.196
7.194
1.571
0.000
1.00
1.04
1.04
3.21
1.04
2.10
f1 (ppm)

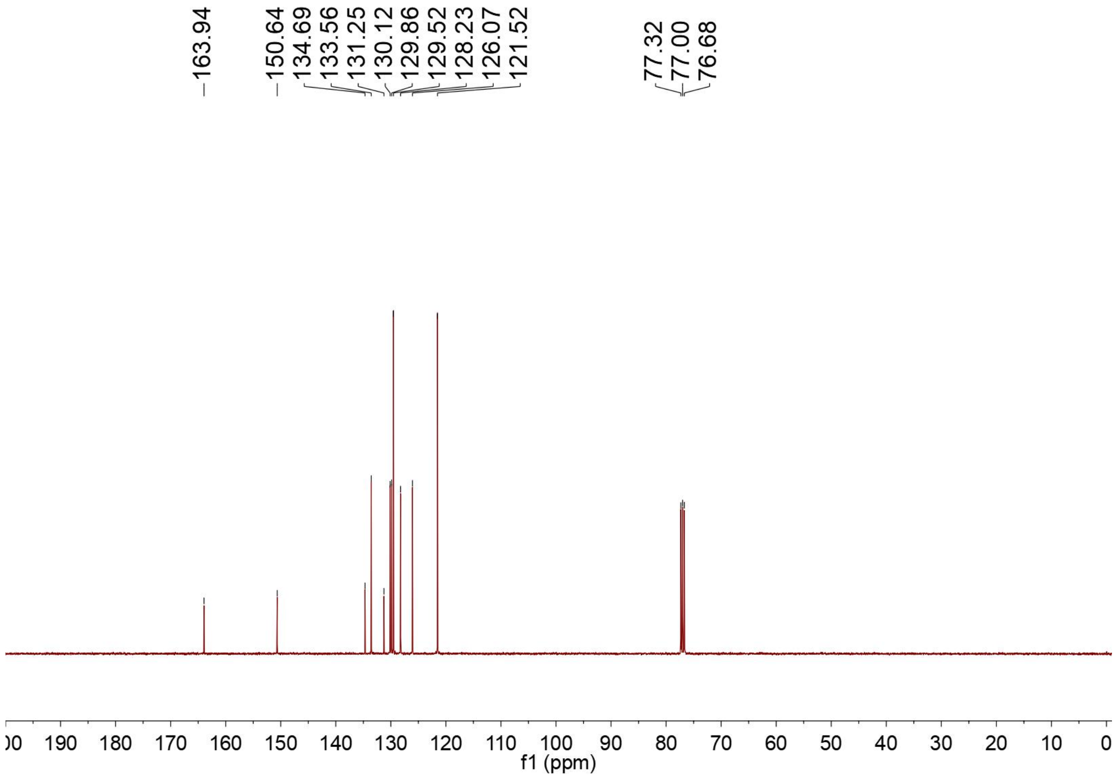

163.94
150.64
134.69
133.56
131.25
130.12
129.86
129.52
128.23
126.07
121.52
77.32
77.00
76.68
f1 (ppm)

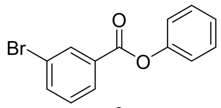
Br
O
O

3am

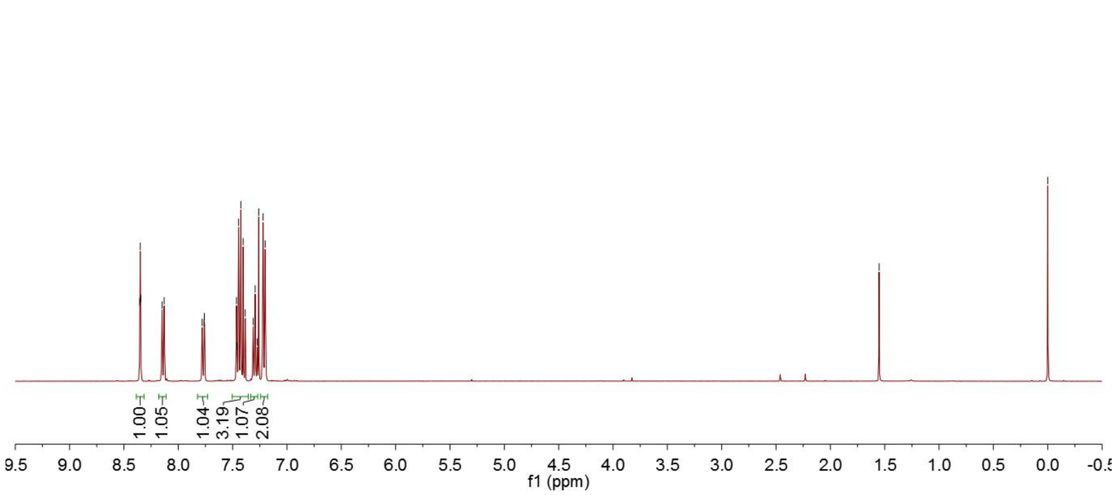
8.354
8.350
8.345
8.149
8.130
7.780
7.763
7.760
7.464
7.460
7.445
7.424
7.404
7.384
7.311
7.293
7.274
7.260
7.220
7.200
1.552
0.000
1.00
1.05
1.04
3.19
1.07
2.08
9.5 9.0 8.5 8.0 7.5 7.0 6.5 6.0 5.5 5.0 4.5 4.0 3.5 3.0 2.5 2.0 1.5 1.0 0.5 0.0
f1 (ppm)

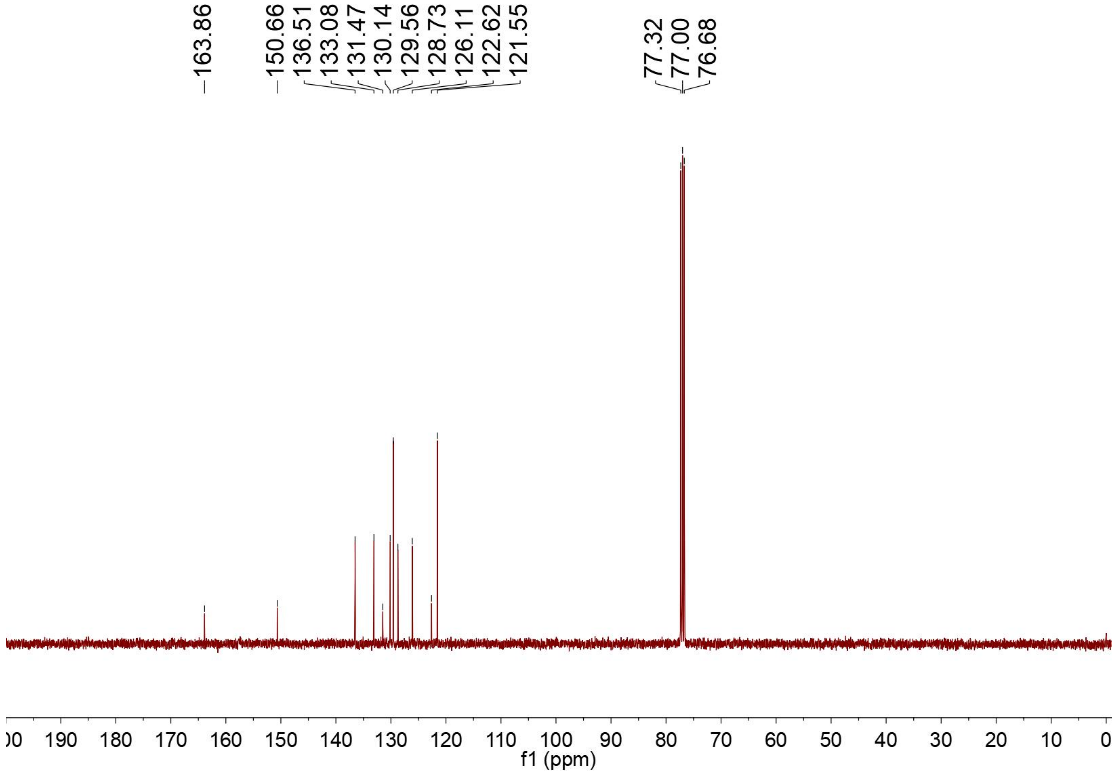
163.86
150.66
136.51
133.08
131.47
130.14
129.56
128.73
126.11
122.62
121.55
77.32
77.00
76.68
190 180 170 160 150 140 130 120 110 100 90 80 70 60 50 40 30 20 10 0
f1 (ppm)

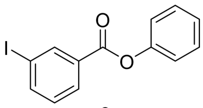

3an

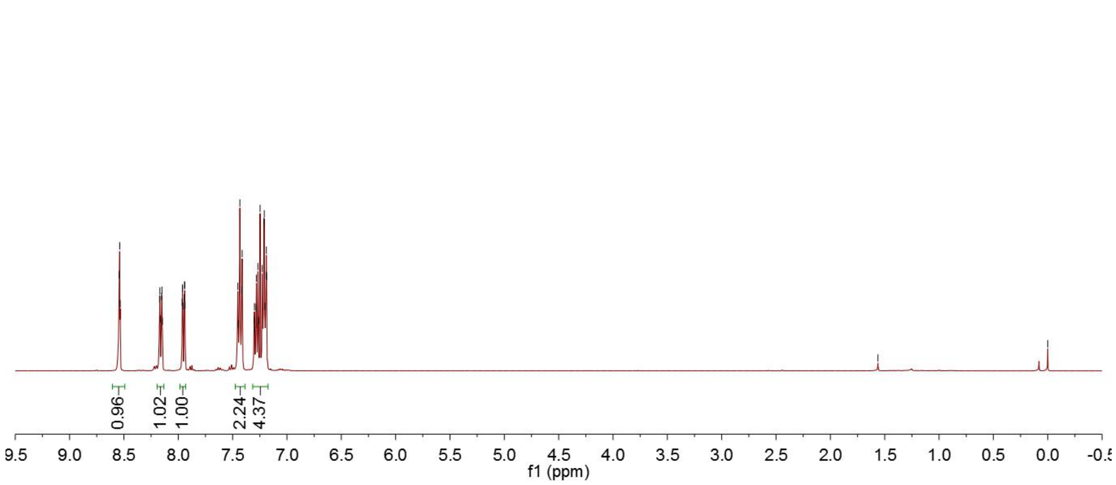
8.543
8.539
8.170
8.150
7.962
7.961
7.942
7.941
7.452
7.447
7.433
7.417
7.412
7.302
7.299
7.296
7.284
7.281
7.267
7.262
7.247
7.228
7.211
7.209
7.203
7.192
7.190
7.188
1.562
0.000
0.96
1.02
1.00
2.24
4.37
9.5 9.0 8.5 8.0 7.5 7.0 6.5 6.0 5.5 5.0 4.5 4.0 3.5 3.0 2.5 2.0 1.5 1.0 0.5 0.0 -0.5
f1 (ppm)

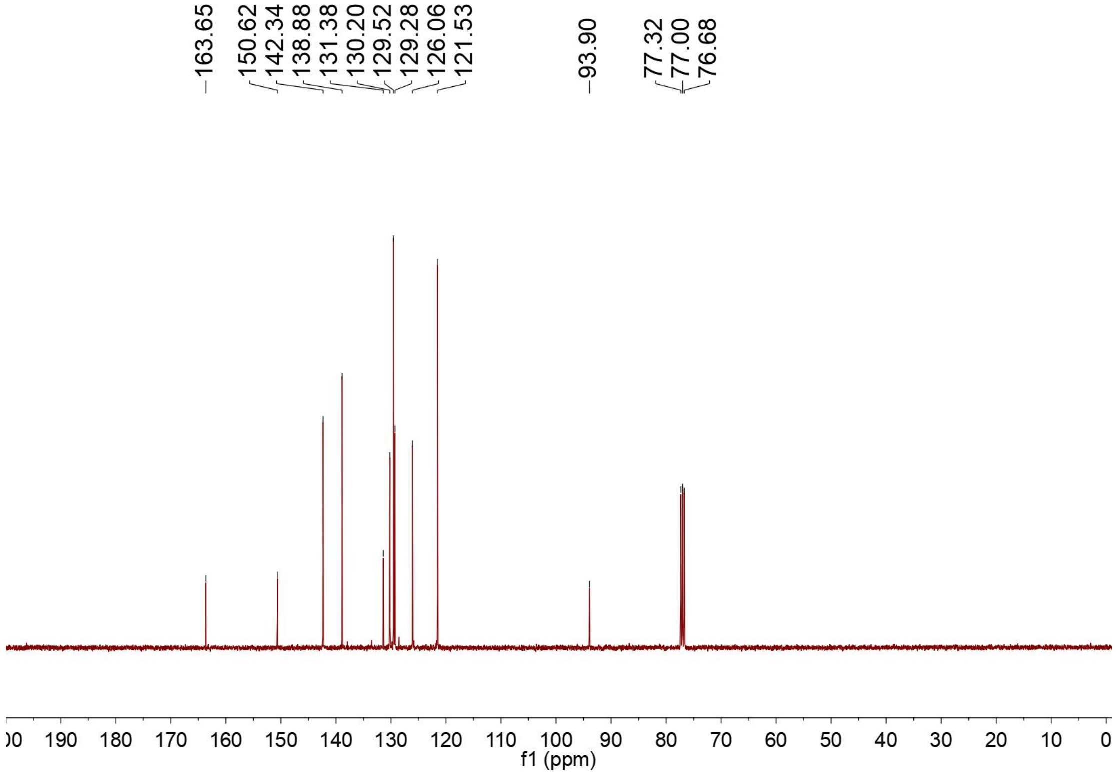
163.65
150.62
142.34
138.88
131.38
130.20
129.52
129.28
126.06
121.53
93.90
77.32
77.00
76.68
190 180 170 160 150 140 130 120 110 100 90 80 70 60 50 40 30 20 10 0
f1 (ppm)

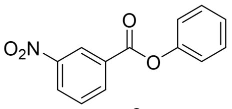
O
O_2N
O

3ao

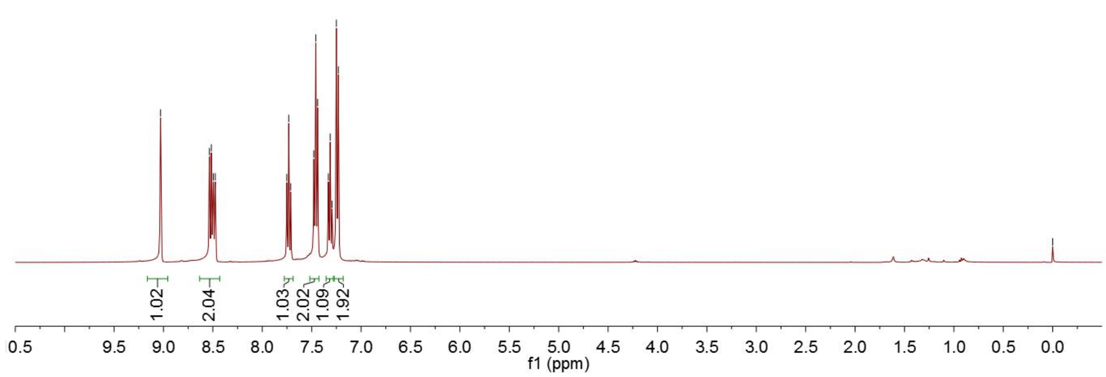
9.029
8.535
8.515
8.476
7.752
7.732
7.712
7.478
7.459
7.439
7.331
7.313
7.294
7.250
7.230
0.000
1.02
2.04
1.03
2.02
1.09
1.92
f1 (ppm)

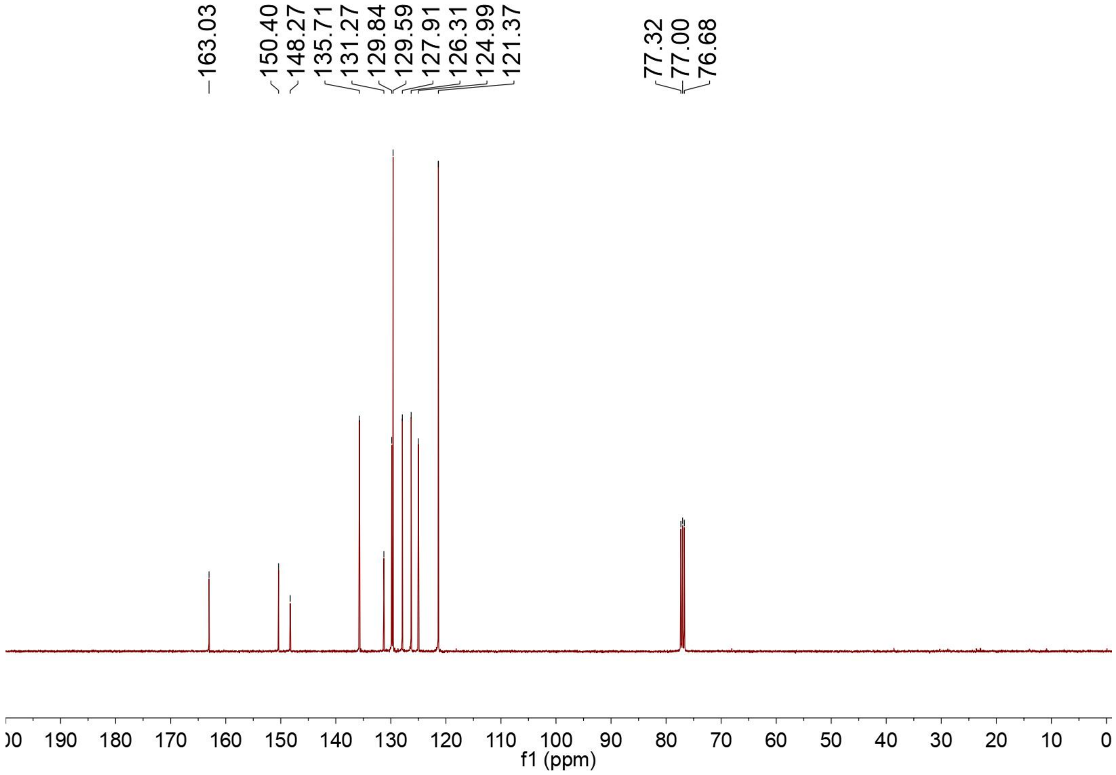
163.03
150.40
148.27
135.71
131.27
129.84
129.59
127.91
126.31
124.99
121.37
77.32
77.00
76.68
f1 (ppm)

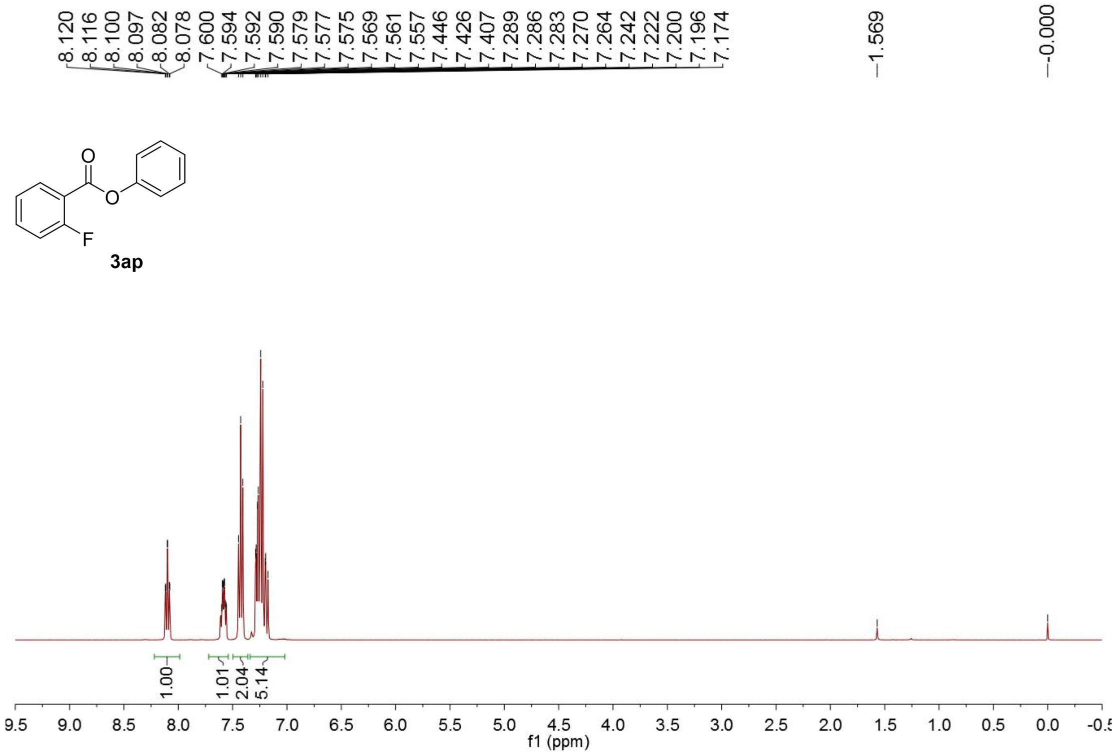
O
O
F
3ap
8.120
8.116
8.100
8.097
8.082
8.078
7.600
7.594
7.592
7.590
7.579
7.577
7.575
7.569
7.561
7.557
7.446
7.426
7.407
7.289
7.286
7.283
7.270
7.264
7.242
7.222
7.200
7.196
7.174
1.569
0.000
1.00
1.01
2.04
5.14
9.5 9.0 8.5 8.0 7.5 7.0 6.5 6.0 5.5 5.0 4.5 4.0 3.5 3.0 2.5 2.0 1.5 1.0 0.5 0.0
f1 (ppm)

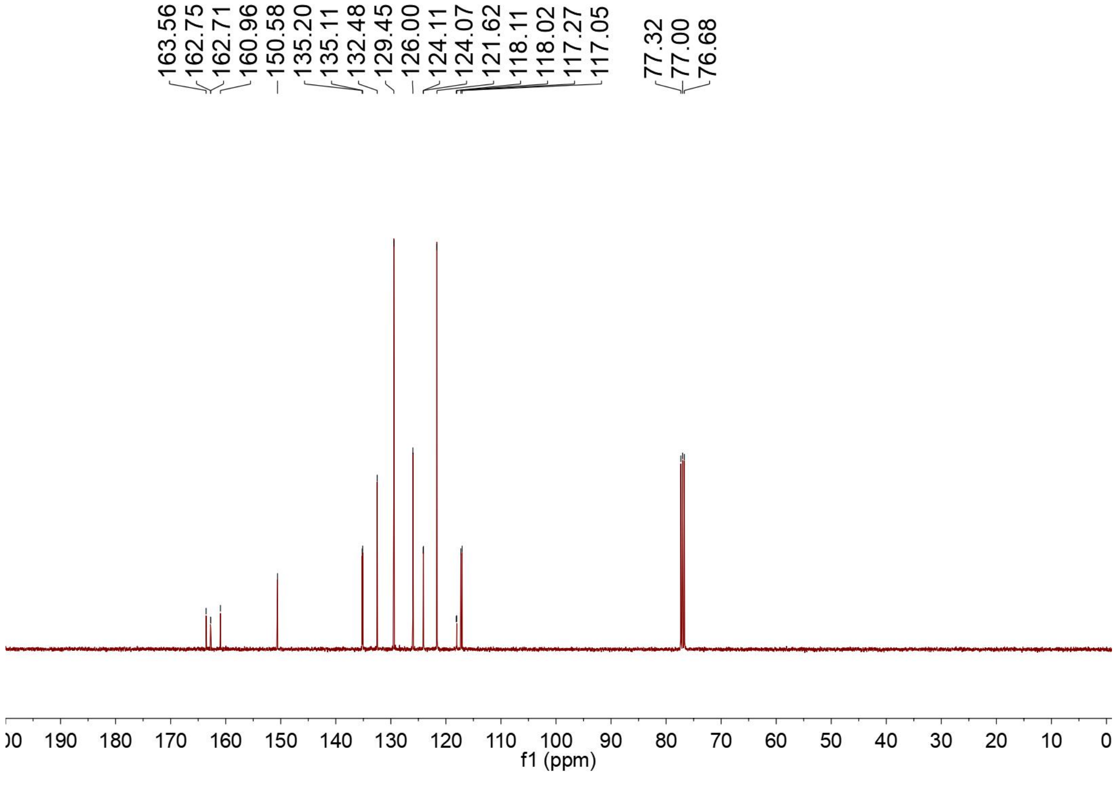
163.56
162.75
162.71
160.96
150.58
135.20
135.11
132.48
129.45
126.00
124.11
124.07
121.62
118.11
118.02
117.27
117.05
77.32
77.00
76.68
190 180 170 160 150 140 130 120 110 100 90 80 70 60 50 40 30 20 10 0
f1 (ppm)

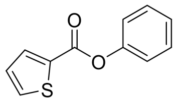

3aq

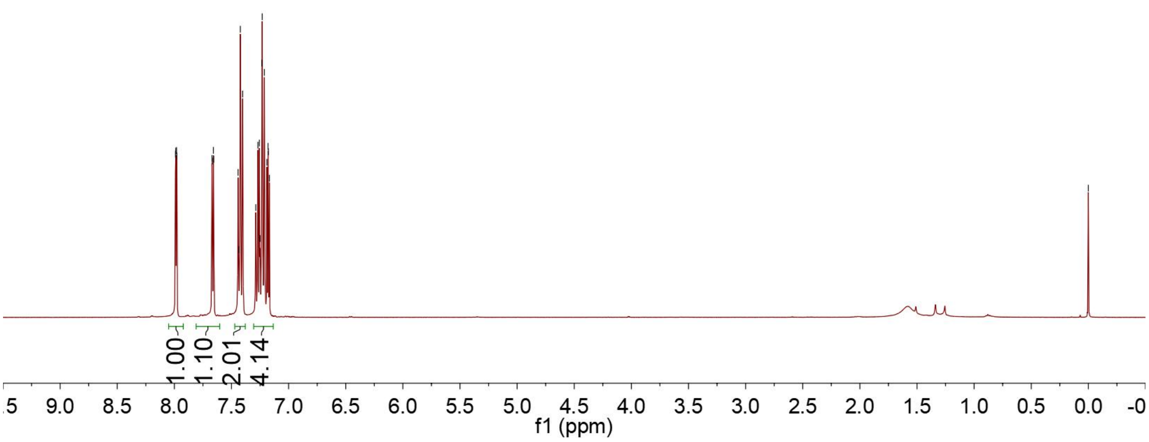
7.990
7.988
7.981
7.978
7.671
7.668
7.658
7.656
7.442
7.438
7.423
7.403
7.287
7.269
7.256
7.250
7.234
7.231
7.212
7.190
7.180
7.178
7.168
0.000
1.00
1.10
2.01
4.14
f1 (ppm)

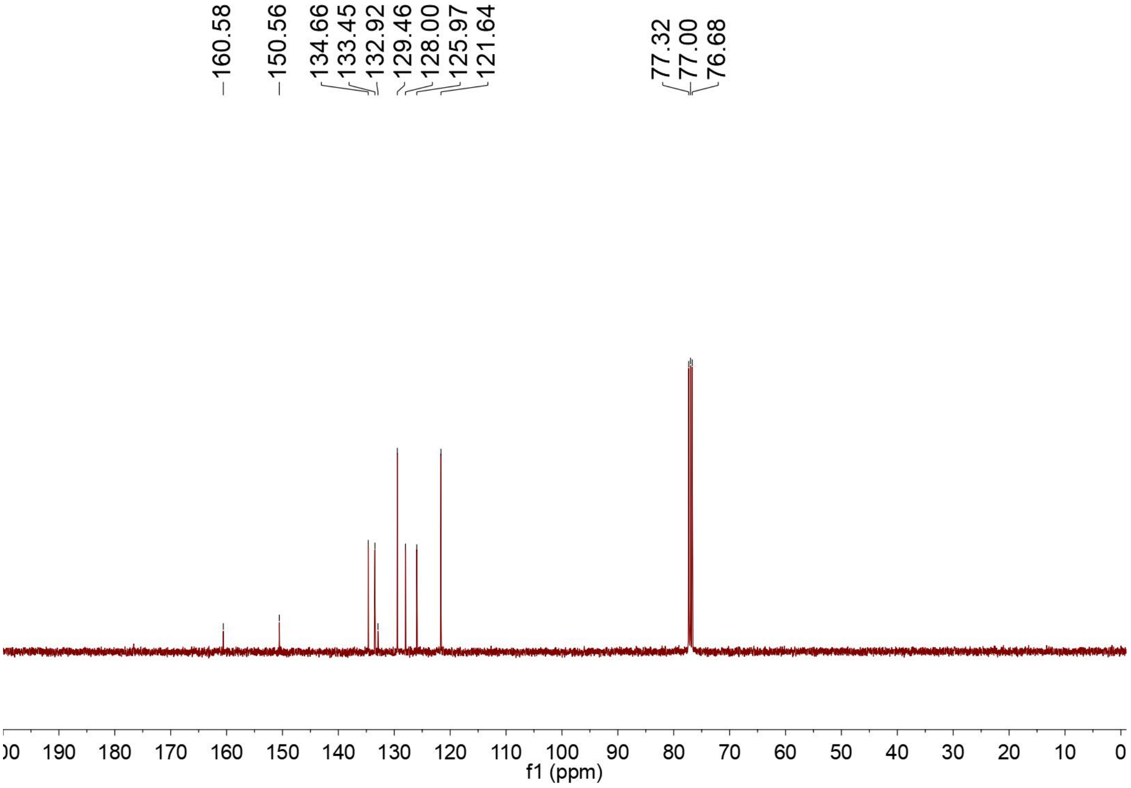
160.58
150.56
134.66
133.45
132.92
129.46
128.00
125.97
121.64
77.32
77.00
76.68
f1 (ppm)

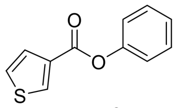

3ar

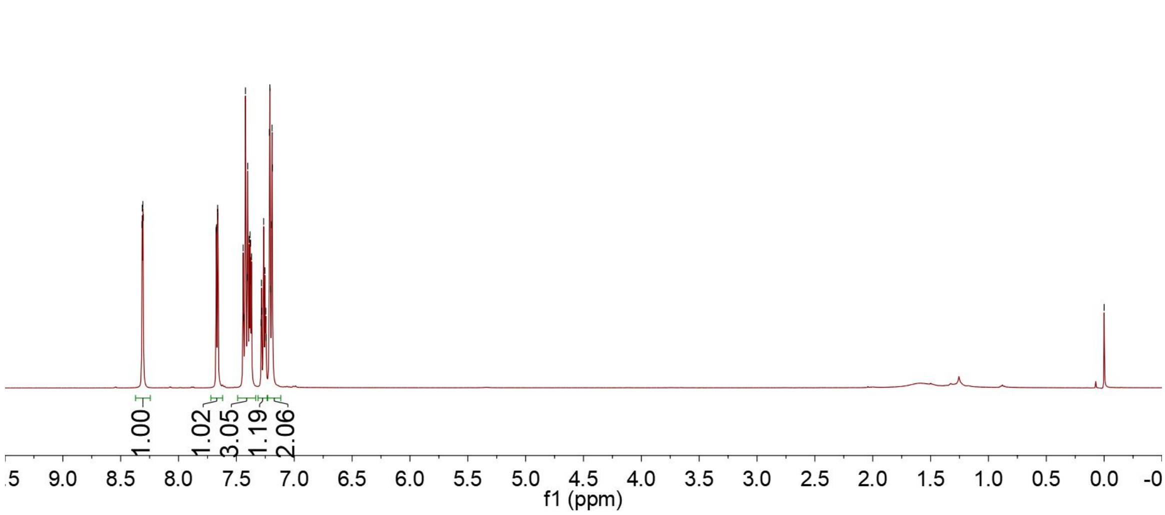
8.316
8.313
8.308
8.305
7.674
7.672
7.662
7.659
7.441
7.437
7.422
7.406
7.402
7.390
7.382
7.377
7.369
7.286
7.283
7.280
7.264
7.253
7.246
7.243
7.214
7.211
7.205
7.195
7.192
7.189
-0.000
1.00
1.02
3.05
1.19
2.06
f1 (ppm)

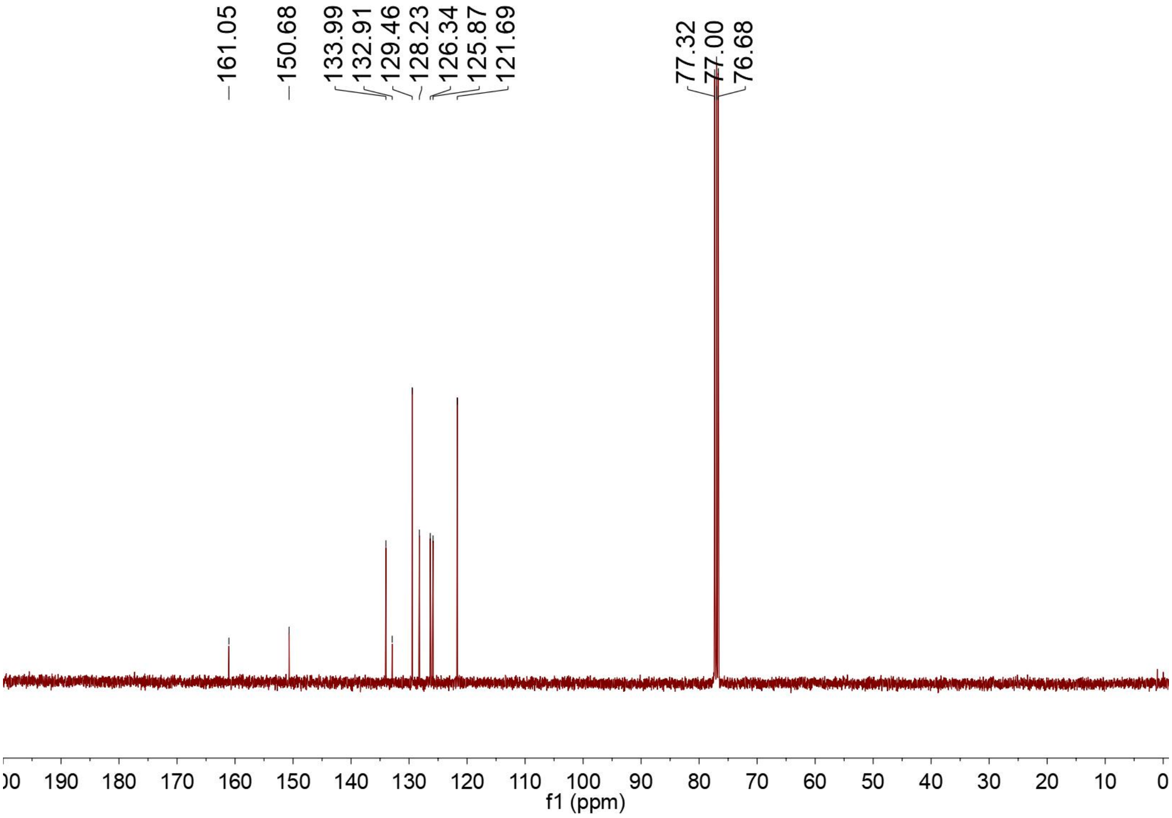
161.05
150.68
133.99
132.91
129.46
128.23
126.34
125.87
121.69
77.32
77.00
76.68
f1 (ppm)

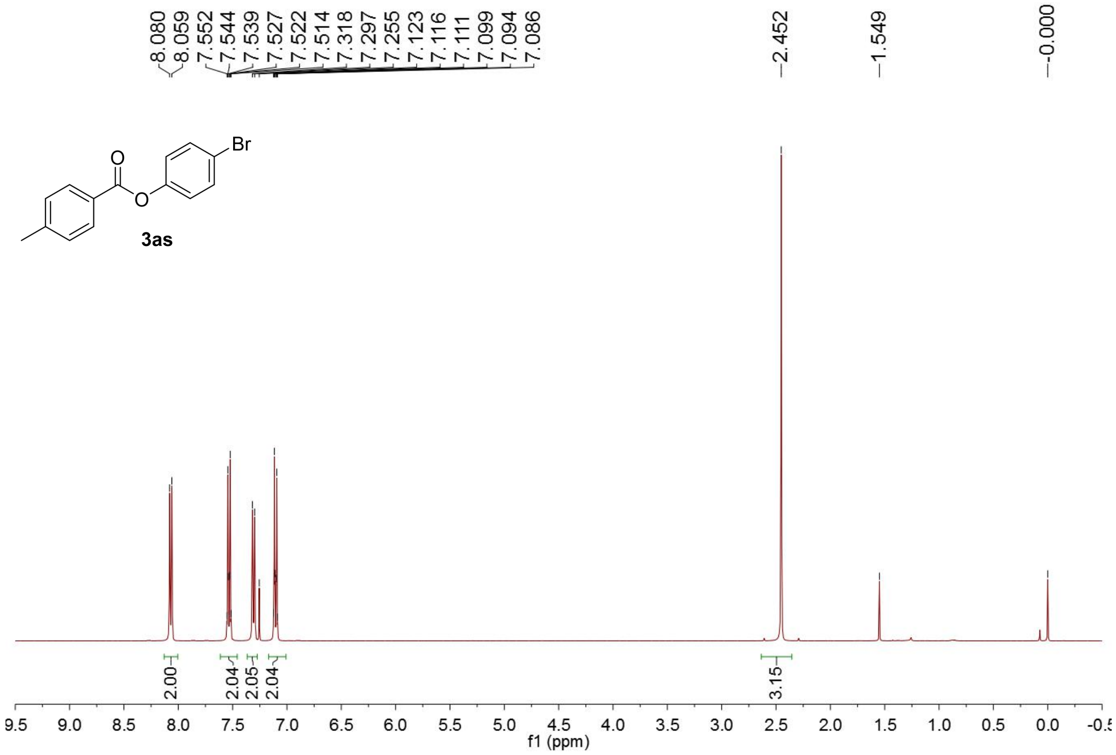
8.080
8.059
7.552
7.544
7.539
7.527
7.522
7.514
7.318
7.297
7.255
7.123
7.116
7.111
7.099
7.094
7.086
2.452
1.549
-0.000
O
O
Br
3as
2.00
2.04
2.05
2.04
3.15
9.5 9.0 8.5 8.0 7.5 7.0 6.5 6.0 5.5 5.0 4.5 4.0 3.5 3.0 2.5 2.0 1.5 1.0 0.5 0.0
f1 (ppm)

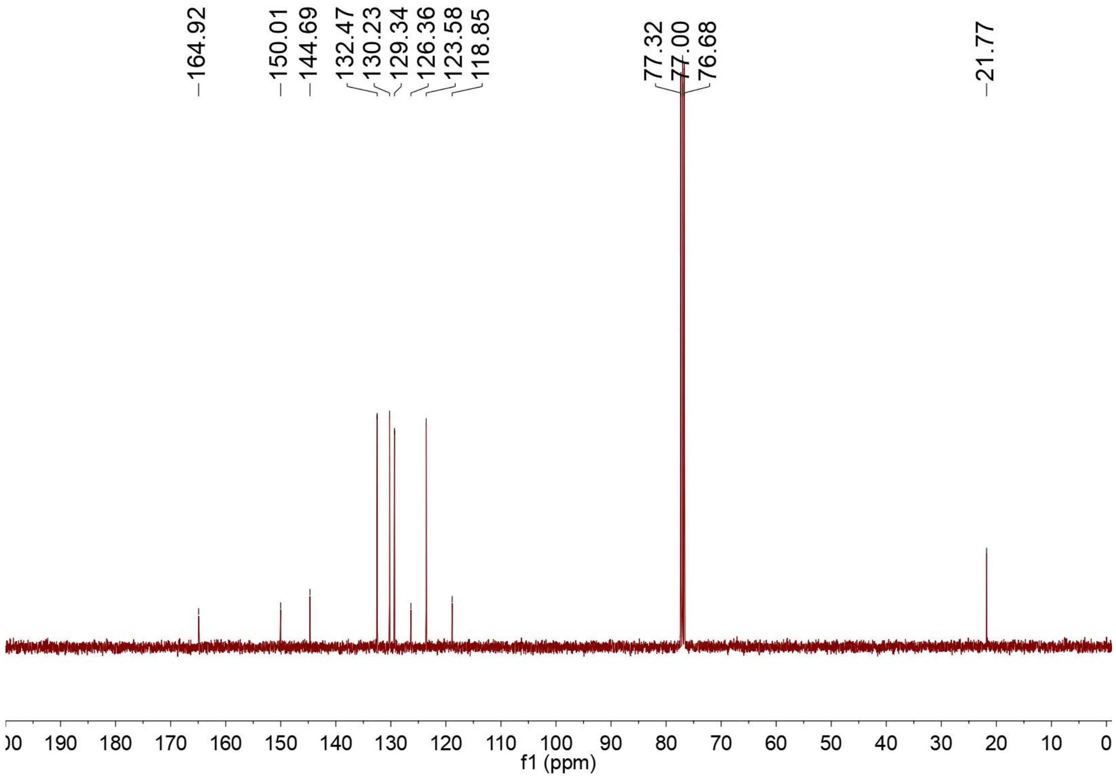
164.92
150.01
144.69
132.47
130.23
129.34
126.36
123.58
118.85
77.32
77.00
76.68
21.77
190 180 170 160 150 140 130 120 110 100 90 80 70 60 50 40 30 20 10 0
f1 (ppm)

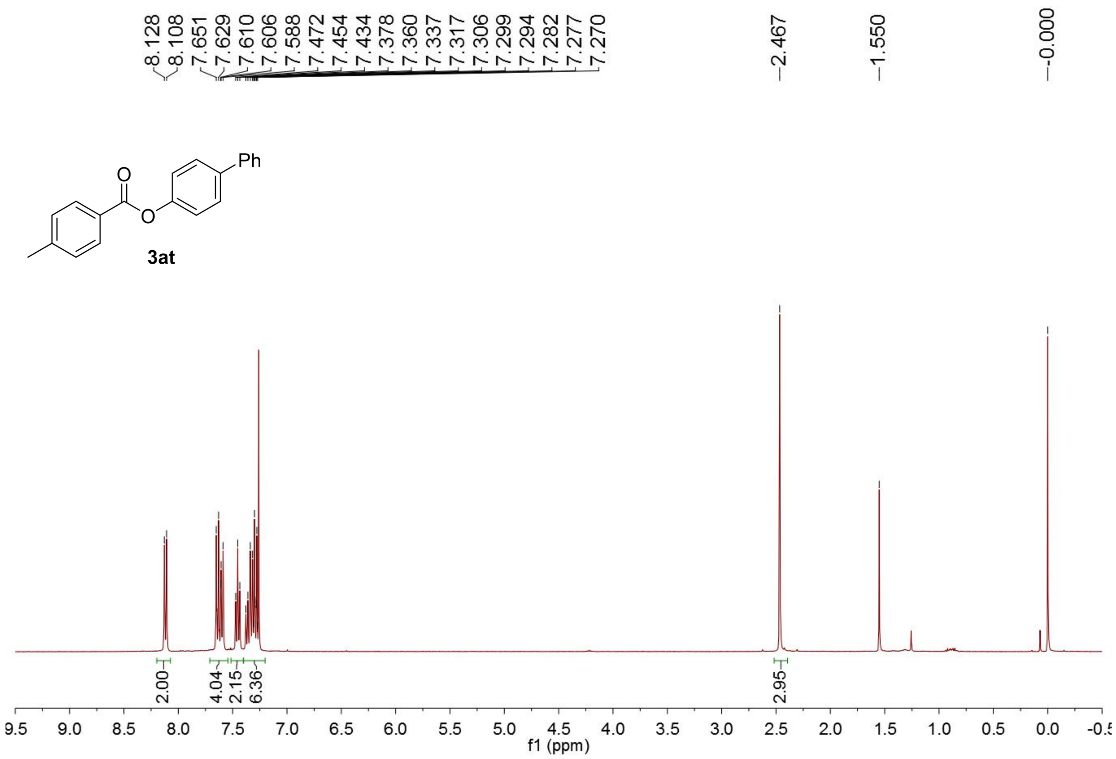
8.128
8.108
7.651
7.629
7.610
7.606
7.588
7.472
7.454
7.434
7.378
7.360
7.337
7.317
7.306
7.299
7.294
7.282
7.277
7.270
2.467
1.550
-0.000
Ph
O
O
3at
2.00
4.04
2.15
6.36
2.95
9.5 9.0 8.5 8.0 7.5 7.0 6.5 6.0 5.5 5.0 4.5 4.0 3.5 3.0 2.5 2.0 1.5 1.0 0.5 0.0 -0.5
f1 (ppm)

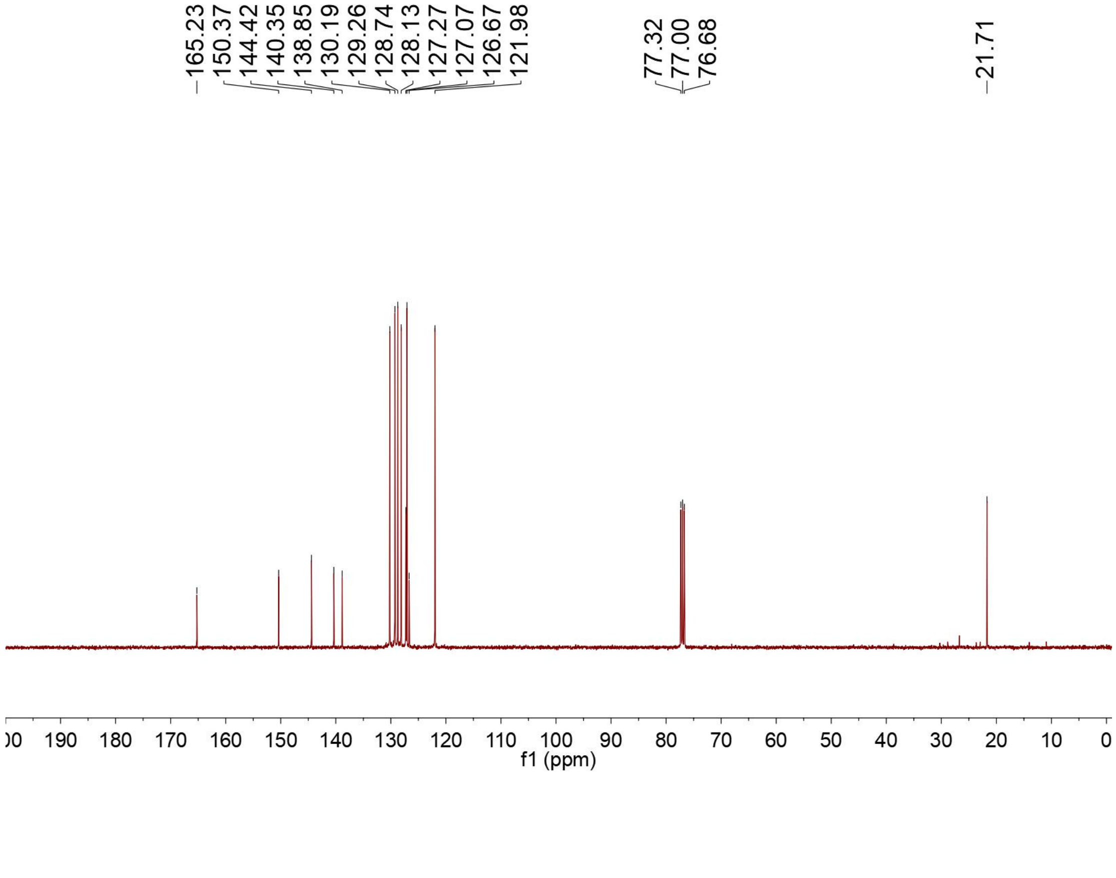
165.23
150.37
144.42
140.35
138.85
130.19
129.26
128.74
128.13
127.27
127.07
126.67
121.98
77.32
77.00
76.68
21.71
200 190 180 170 160 150 140 130 120 110 100 90 80 70 60 50 40 30 20 10 0
f1 (ppm)

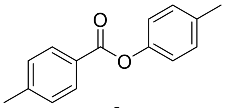

3au

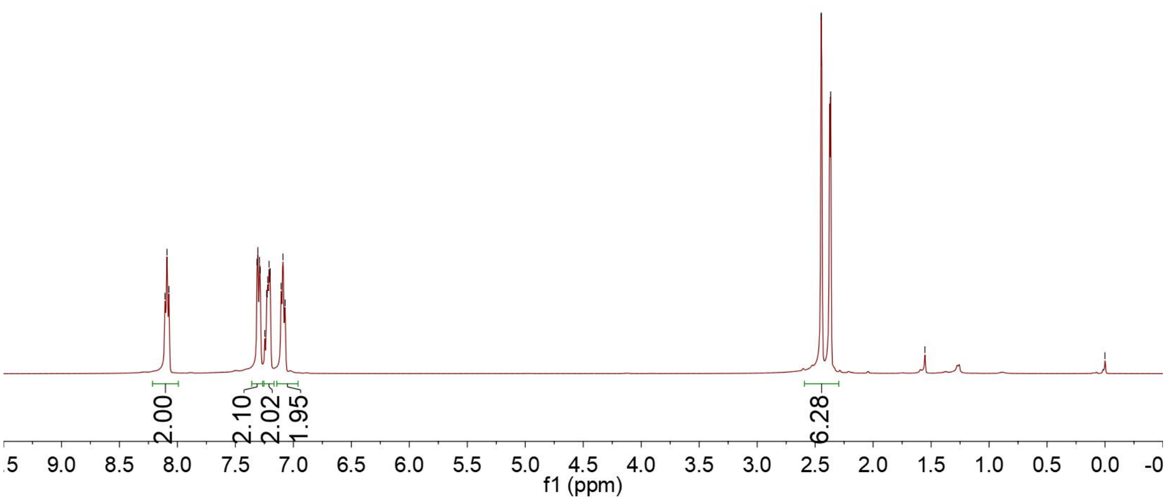

8.107
8.090
8.073
7.313
7.307
7.292
7.286
7.247
7.230
7.221
7.209
7.200
7.106
7.090
7.071
2.449
2.366
1.553
-0.001
2.00
2.10
2.02
1.95
6.28
f1 (ppm)

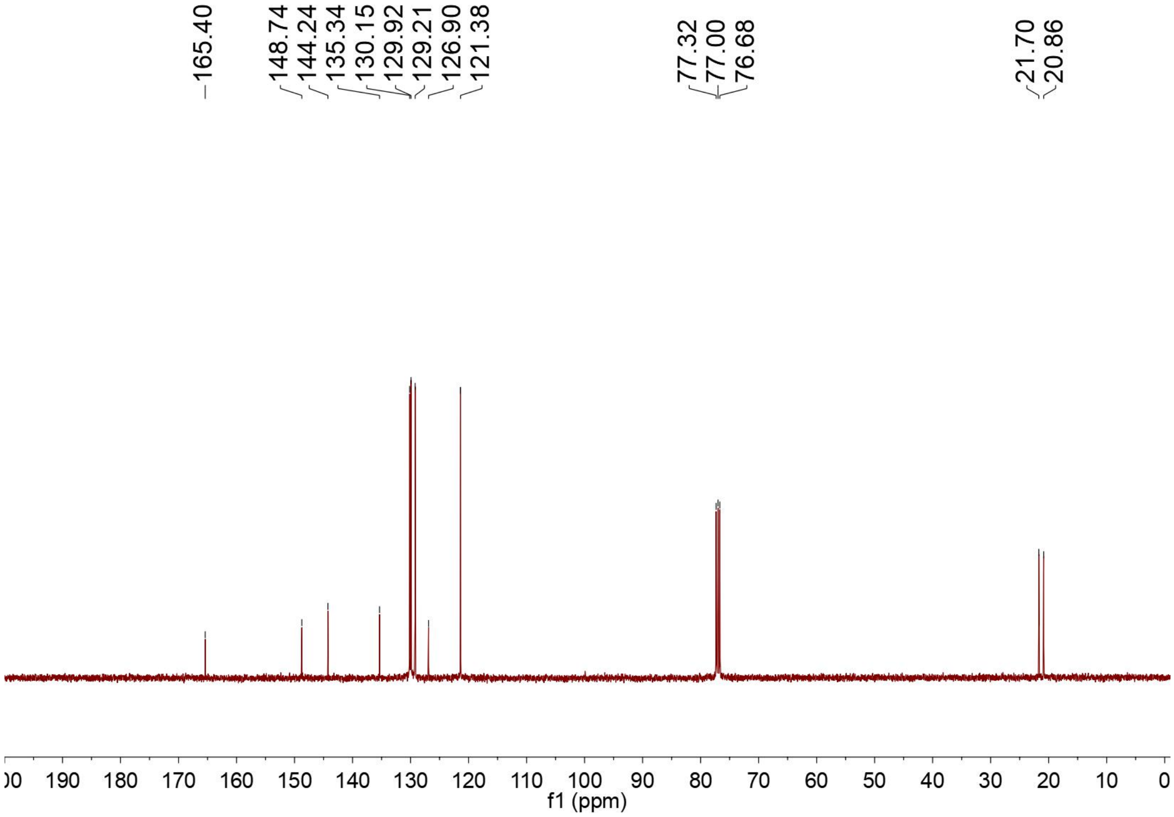

165.40
148.74
144.24
135.34
130.15
129.92
129.21
126.90
121.38
77.32
77.00
76.68
21.70
20.86
f1 (ppm)

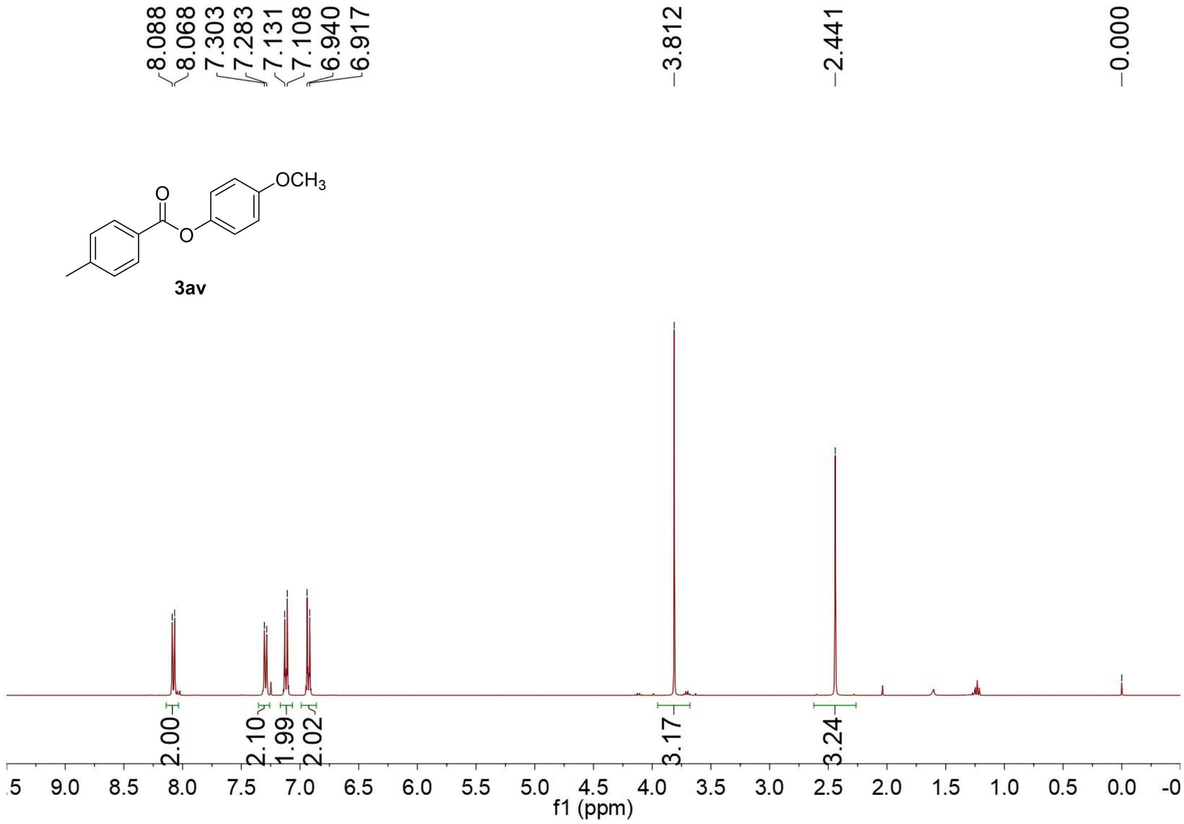
OCH3
O
O
3av
8.088
8.068
7.303
7.283
7.131
7.108
6.940
6.917
3.812
2.441
0.000
2.00
2.10
1.99
2.02
3.17
3.24
f1 (ppm)

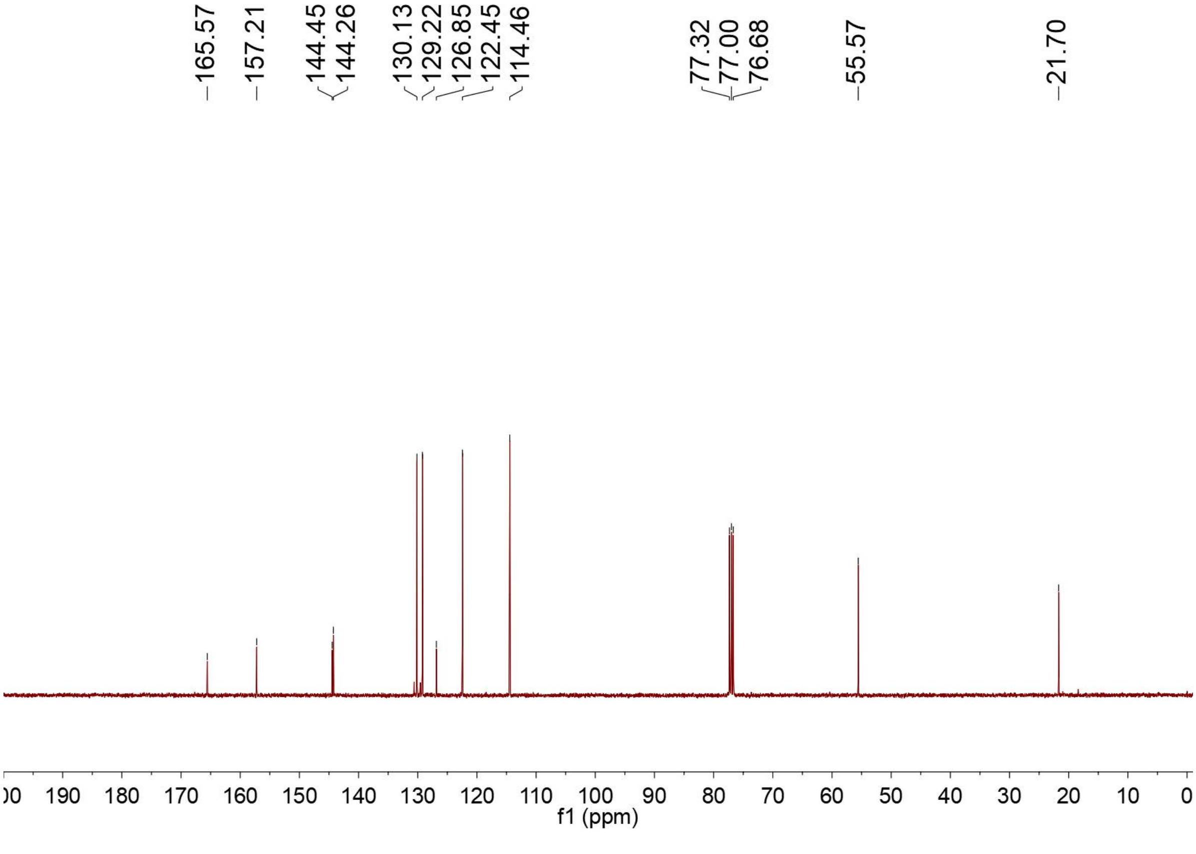
165.57
157.21
144.45
144.26
130.13
129.22
126.85
122.45
114.46
77.32
77.00
76.68
55.57
21.70
f1 (ppm)

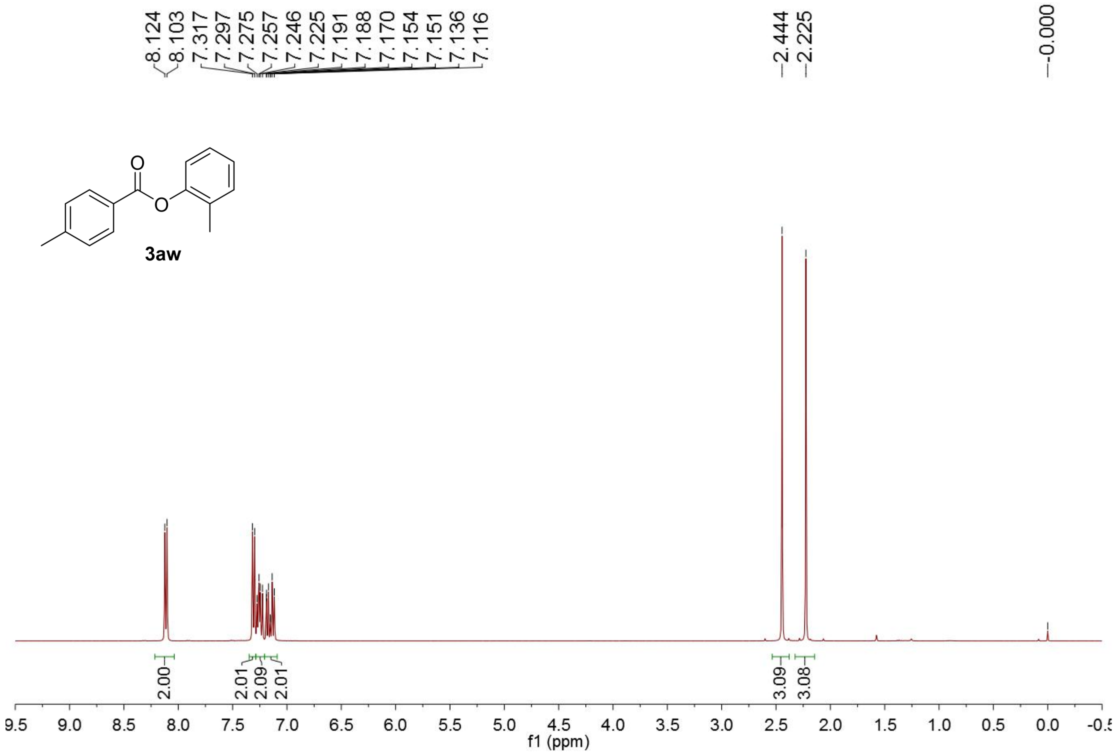
8.124
8.103
7.317
7.297
7.275
7.257
7.246
7.225
7.191
7.188
7.170
7.154
7.151
7.136
7.116
2.444
2.225
-0.000
3aw
2.00
2.01
2.09
2.01
3.09
3.08
f1 (ppm)

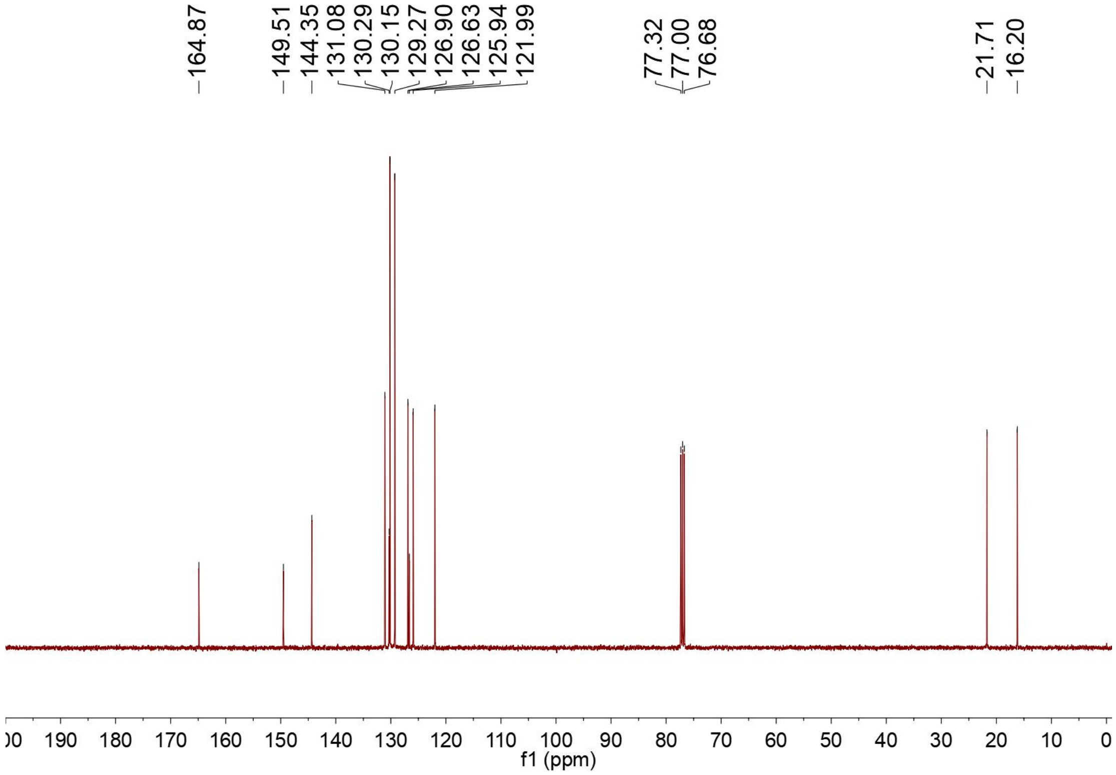
164.87
149.51
144.35
131.08
130.29
130.15
129.27
126.90
126.63
125.94
121.99
77.32
77.00
76.68
21.71
16.20
f1 (ppm)

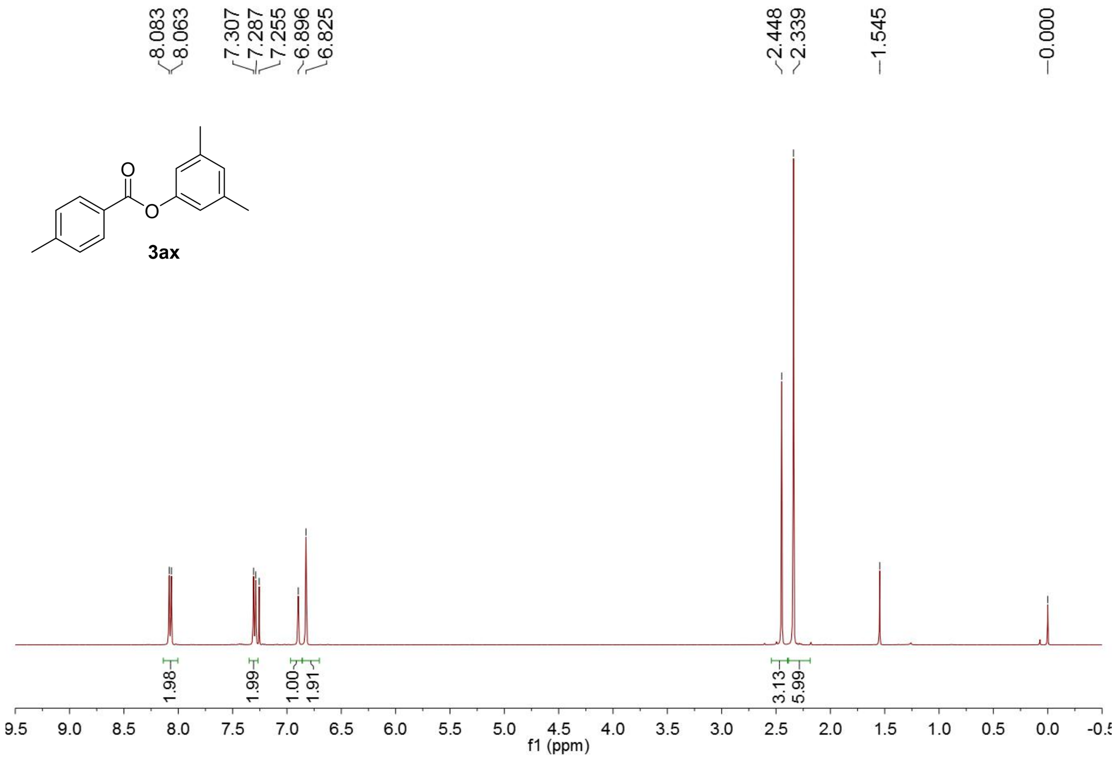
8.083
8.063
7.307
7.287
7.255
6.896
6.825
2.448
2.339
1.545
0.000
3ax
1.98
1.99
1.00
1.91
3.13
5.99
9.5 9.0 8.5 8.0 7.5 7.0 6.5 6.0 5.5 5.0 4.5 4.0 3.5 3.0 2.5 2.0 1.5 1.0 0.5 0.0 -0.5
f1 (ppm)

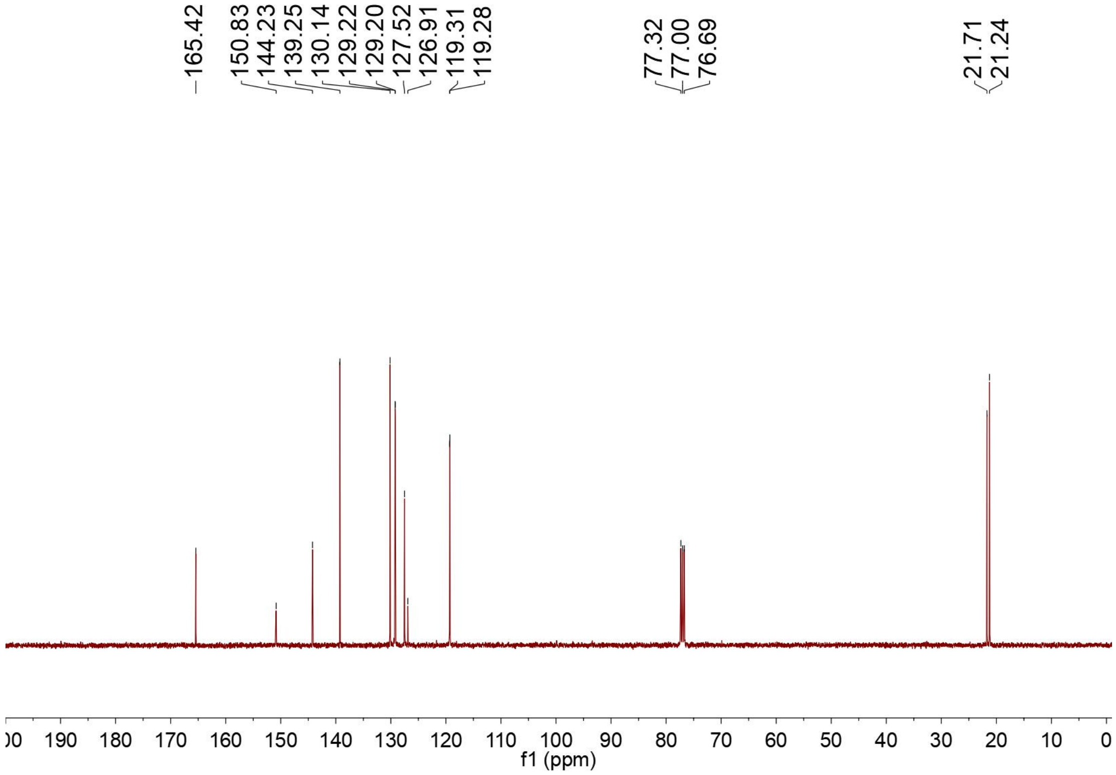
165.42
150.83
144.23
139.25
130.14
129.22
129.20
127.52
126.91
119.31
119.28
77.32
77.00
76.69
21.71
21.24
00 190 180 170 160 150 140 130 120 110 100 90 80 70 60 50 40 30 20 10 0
f1 (ppm)

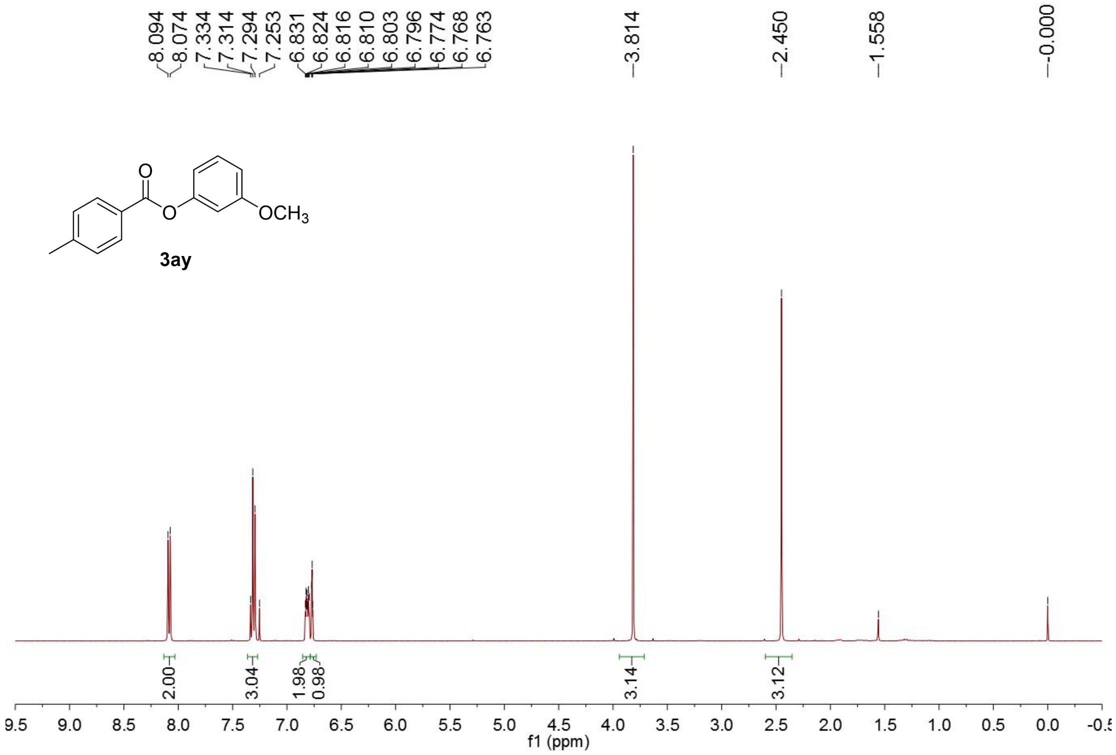
8.094
8.074
7.334
7.314
7.294
7.253
6.831
6.824
6.816
6.810
6.803
6.796
6.774
6.768
6.763
3.814
2.450
1.558
-0.000
O
O
OCH3
3ay
2.00
3.04
1.98
0.98
3.14
3.12
f1 (ppm)

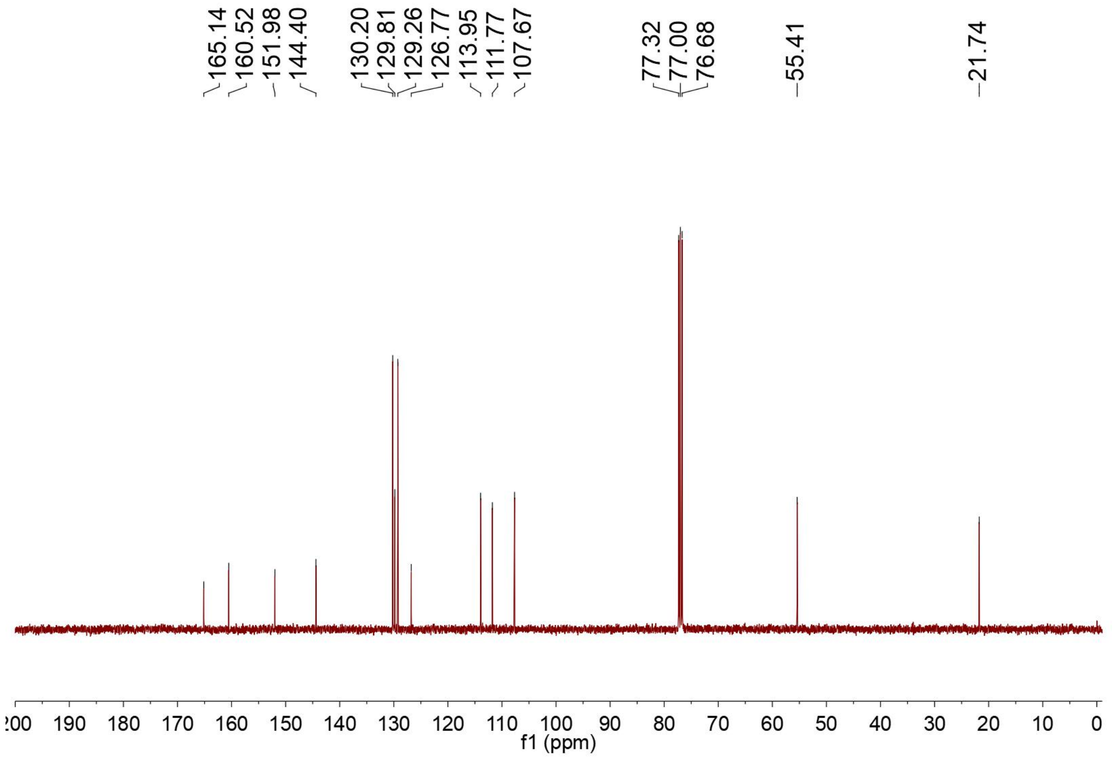
165.14
160.52
151.98
144.40
130.20
129.81
129.26
126.77
113.95
111.77
107.67
77.32
77.00
76.68
55.41
21.74
f1 (ppm)

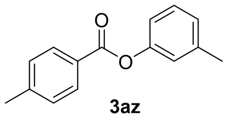

3az

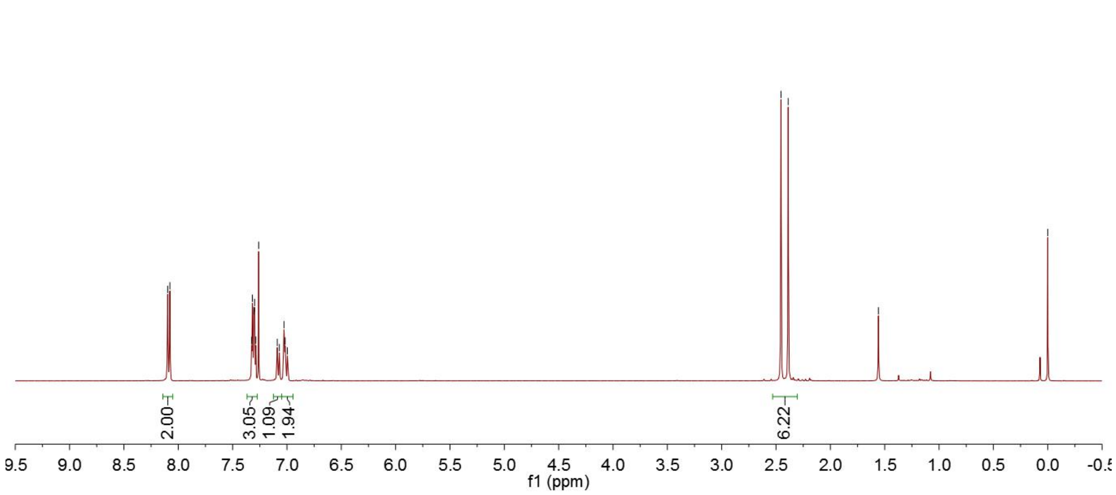
8.098
8.077
7.325
7.318
7.306
7.298
7.287
7.260
7.089
7.070
7.027
7.017
6.997
2.455
2.388
1.558
-0.000
2.00
3.05
1.09
1.94
6.22
f1 (ppm)

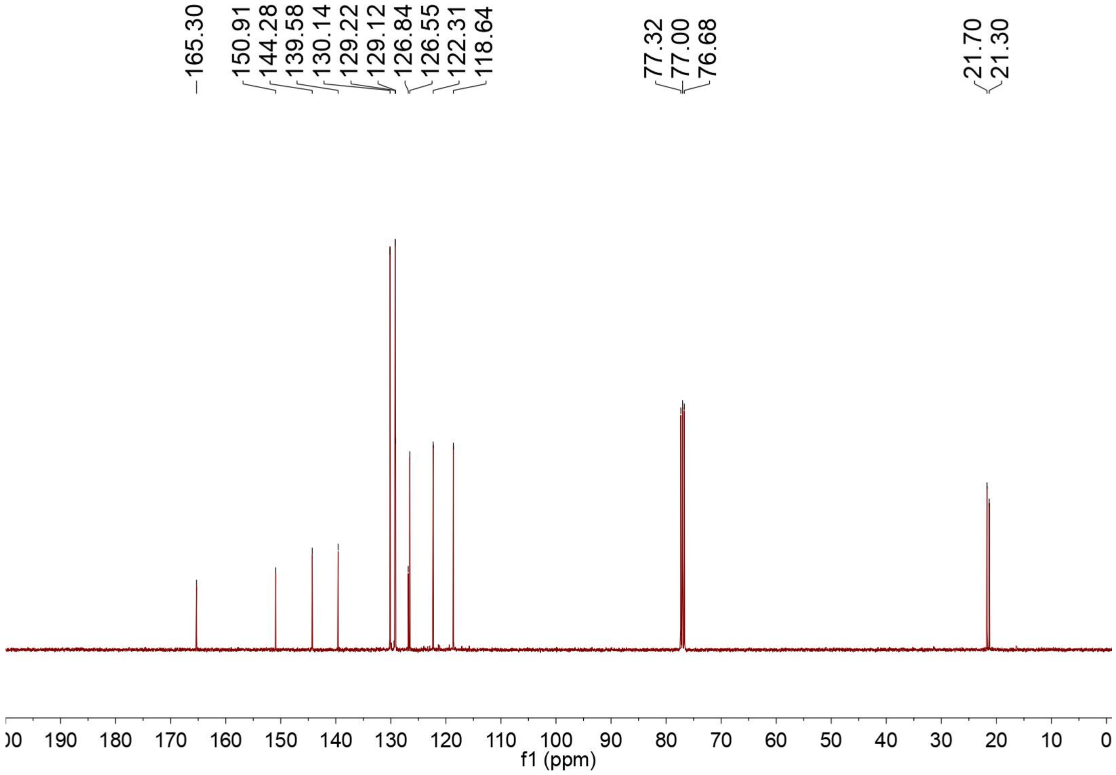
165.30
150.91
144.28
139.58
130.14
129.22
129.12
126.84
126.55
122.31
118.64
77.32
77.00
76.68
21.70
21.30
f1 (ppm)

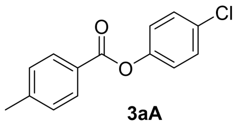
Cl
O
O

3aA

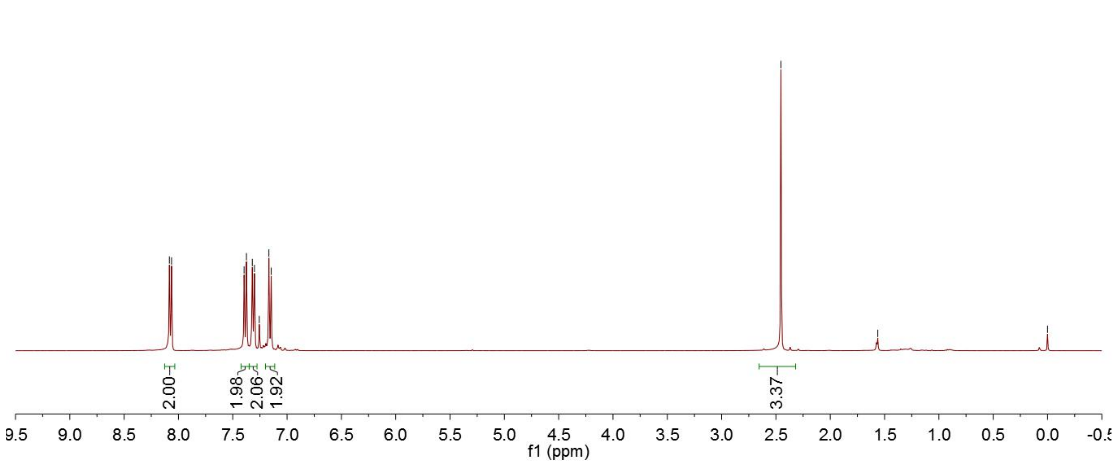
8.084
8.064
7.396
7.374
7.320
7.300
7.256
7.167
7.146
2.454
1.562
0.000
2.00
1.98
2.06
1.92
3.37
9.5 9.0 8.5 8.0 7.5 7.0 6.5 6.0 5.5 5.0 4.5 4.0 3.5 3.0 2.5 2.0 1.5 1.0 0.5 0.0 -0.5
f1 (ppm)

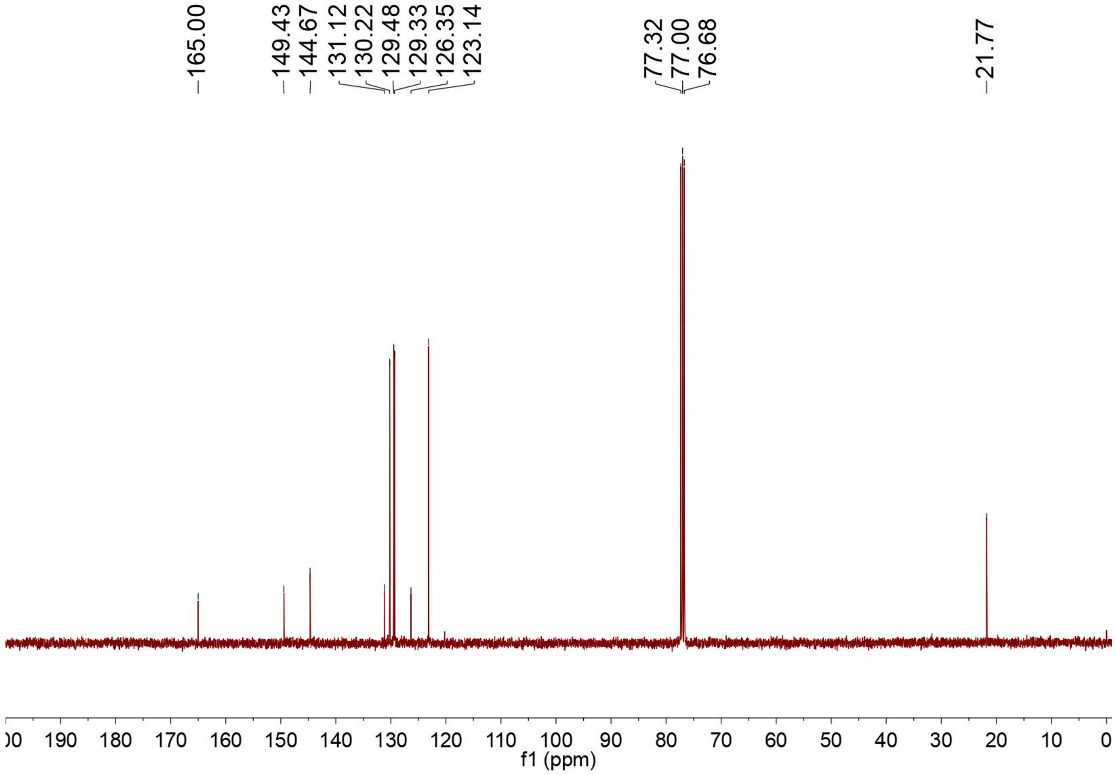
165.00
149.43
144.67
131.12
130.22
129.48
129.33
126.35
123.14
77.32
77.00
76.68
21.77
190 180 170 160 150 140 130 120 110 100 90 80 70 60 50 40 30 20 10 0
f1 (ppm)

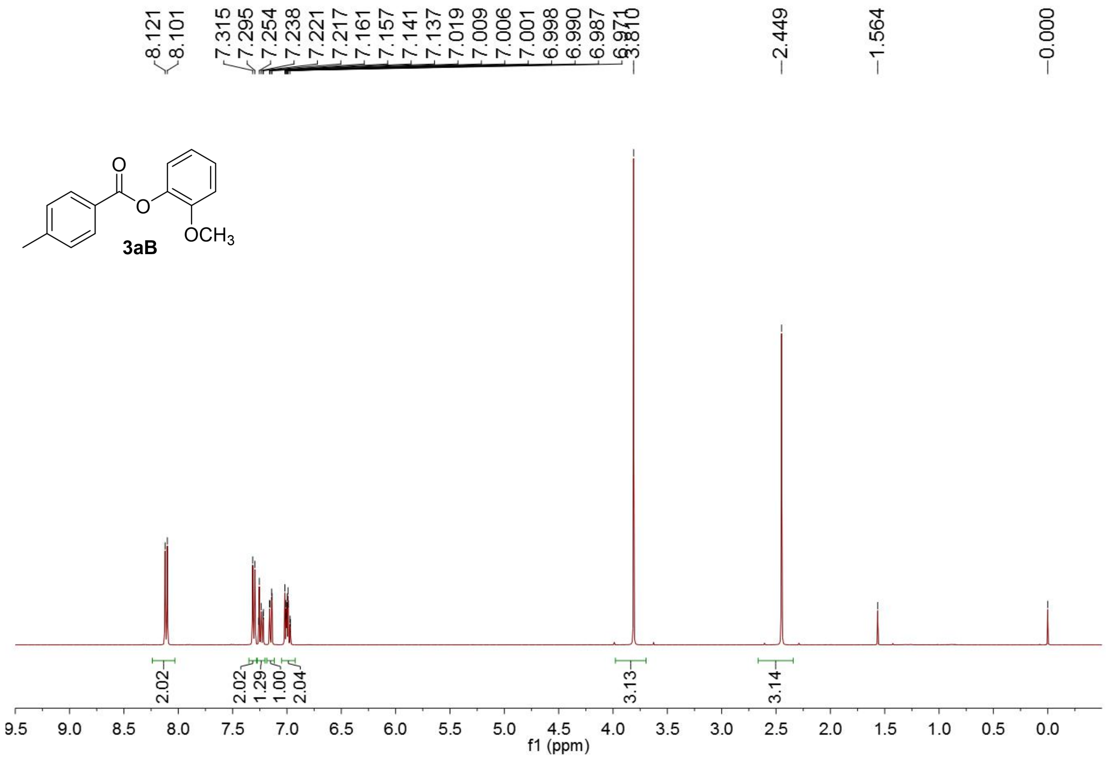
8.121
8.101
7.315
7.295
7.254
7.238
7.221
7.217
7.161
7.157
7.141
7.137
7.019
7.009
7.006
7.001
6.998
6.990
6.987
6.971
3.810
2.449
1.564
0.000
O
O
OCH3
3aB
2.02
2.02
1.29
1.00
2.04
3.13
3.14
9.5
9.0
8.5
8.0
7.5
7.0
6.5
6.0
5.5
5.0
4.5
4.0
3.5
3.0
2.5
2.0
1.5
1.0
0.5
0.0
f1 (ppm)

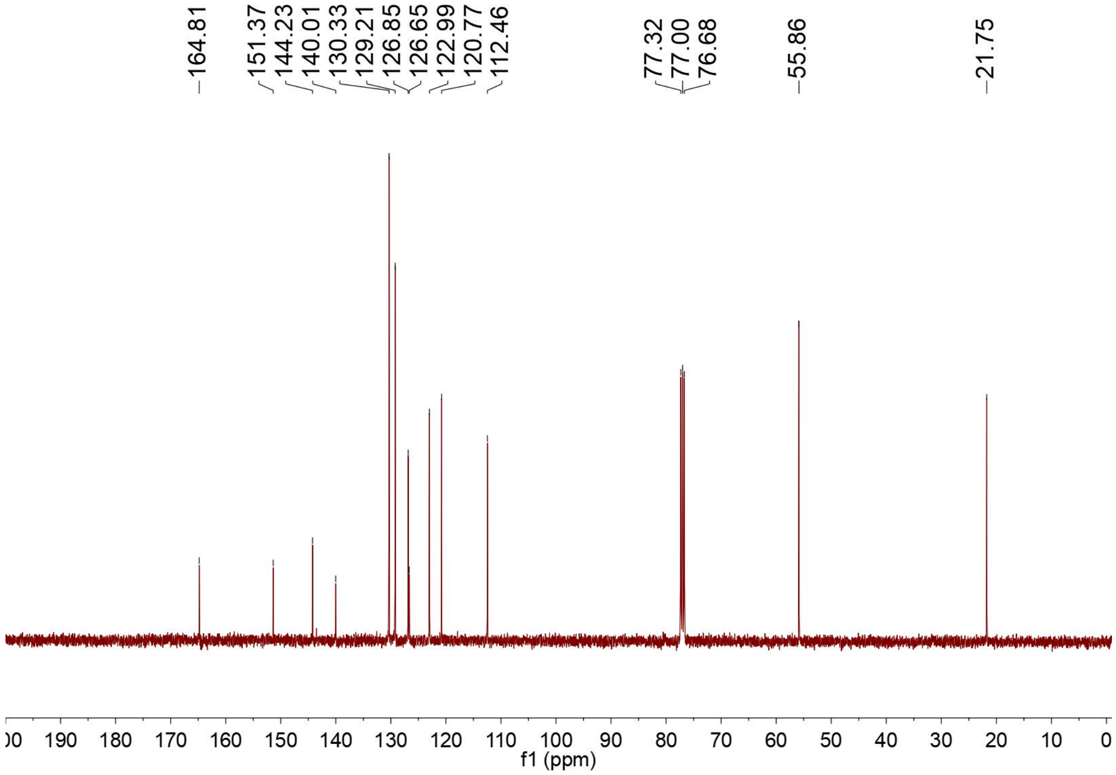
164.81
151.37
144.23
140.01
130.33
129.21
126.85
126.65
122.99
120.77
112.46
77.32
77.00
76.68
55.86
21.75
190
180
170
160
150
140
130
120
110
100
90
80
70
60
50
40
30
20
10
0
f1 (ppm)

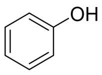
OH

4a

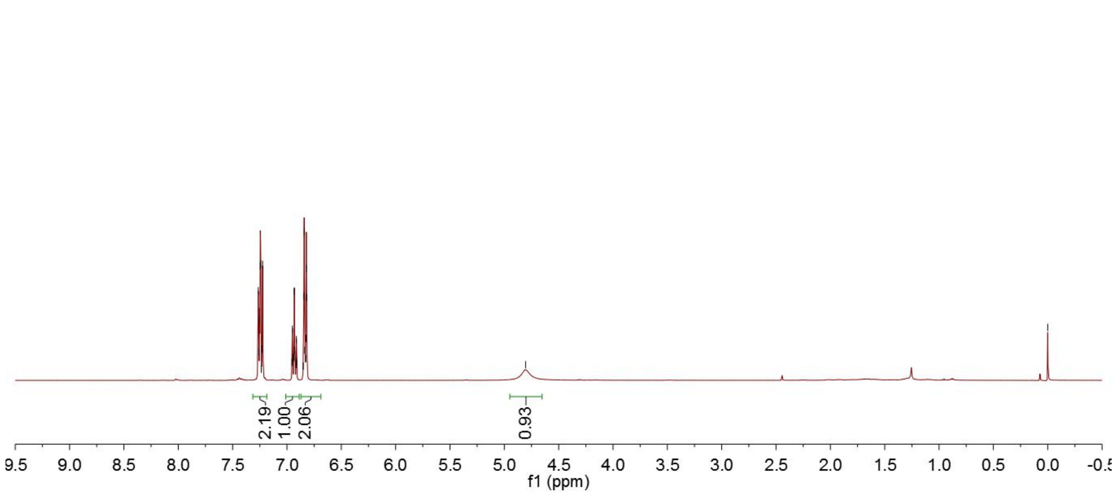
7.264
7.259
7.252
7.245
7.242
7.229
7.224
6.950
6.948
6.935
6.932
6.929
6.913
6.911
6.844
6.841
6.836
6.828
6.825
6.822
6.820
4.804
-0.000
2.19
1.00
2.06
0.93
9.5 9.0 8.5 8.0 7.5 7.0 6.5 6.0 5.5 5.0 4.5 4.0 3.5 3.0 2.5 2.0 1.5 1.0 0.5 0.0
f1 (ppm)

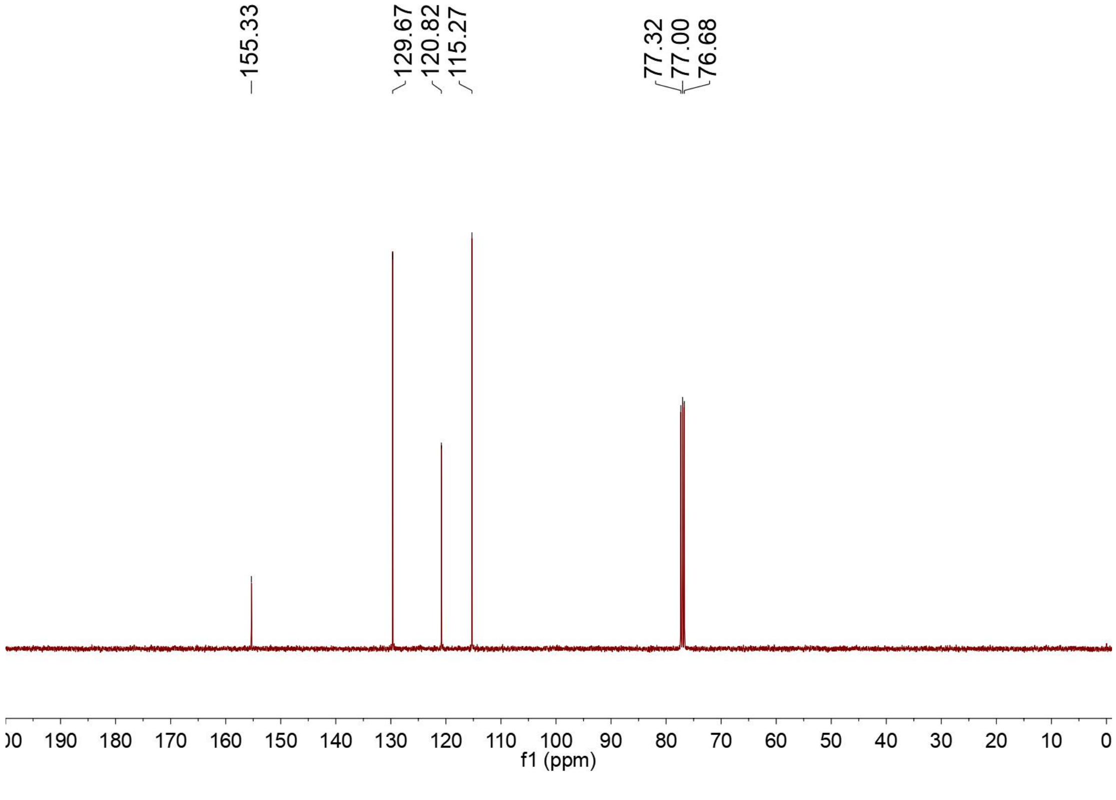
155.33
129.67
120.82
115.27
77.32
77.00
76.68
190 180 170 160 150 140 130 120 110 100 90 80 70 60 50 40 30 20 10 0
f1 (ppm)

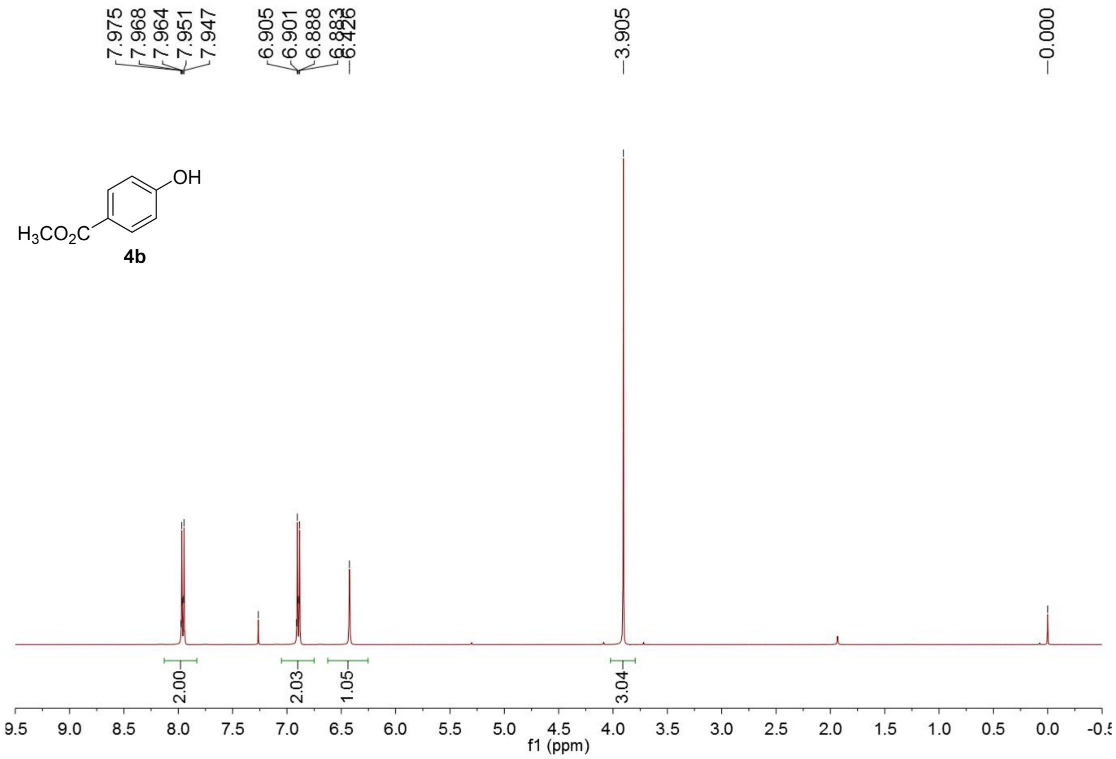
7.975
7.968
7.964
7.951
7.947
6.905
6.901
6.888
6.883
6.426
3.905
0.000
OH
H_3CO_2C
4b
2.00
2.03
1.05
3.04
9.5 9.0 8.5 8.0 7.5 7.0 6.5 6.0 5.5 5.0 4.5 4.0 3.5 3.0 2.5 2.0 1.5 1.0 0.5 0.0 -0.5
f1 (ppm)

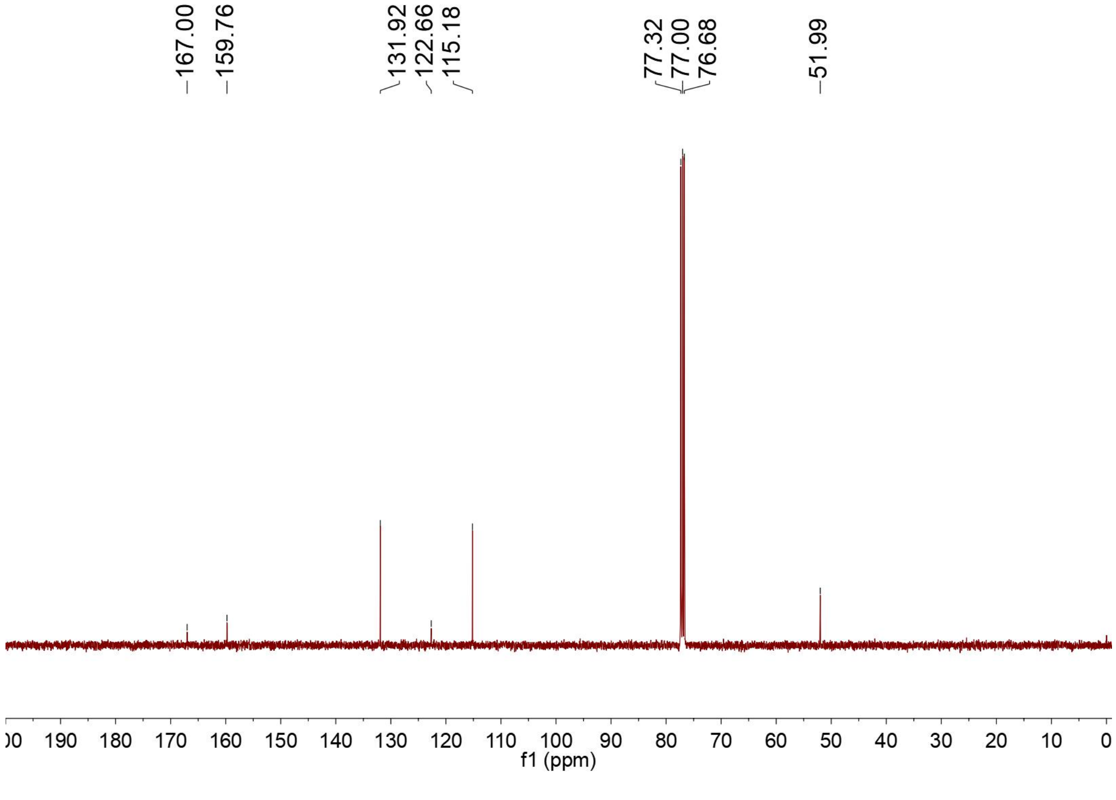
167.00
159.76
131.92
122.66
115.18
77.32
77.00
76.68
51.99
190 180 170 160 150 140 130 120 110 100 90 80 70 60 50 40 30 20 10 0
f1 (ppm)

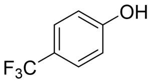
OH
F3C

4c

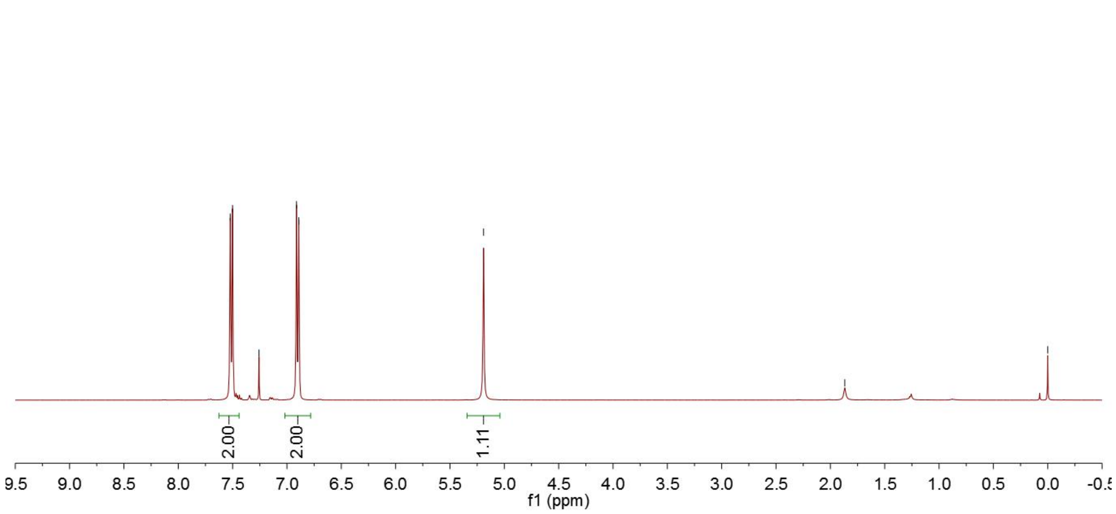
7.522
7.501
7.257
6.912
6.891
5.191
1.867
0.000
2.00
2.00
1.11
9.5 9.0 8.5 8.0 7.5 7.0 6.5 6.0 5.5 5.0 4.5 4.0 3.5 3.0 2.5 2.0 1.5 1.0 0.5 0.0 -0.5
f1 (ppm)

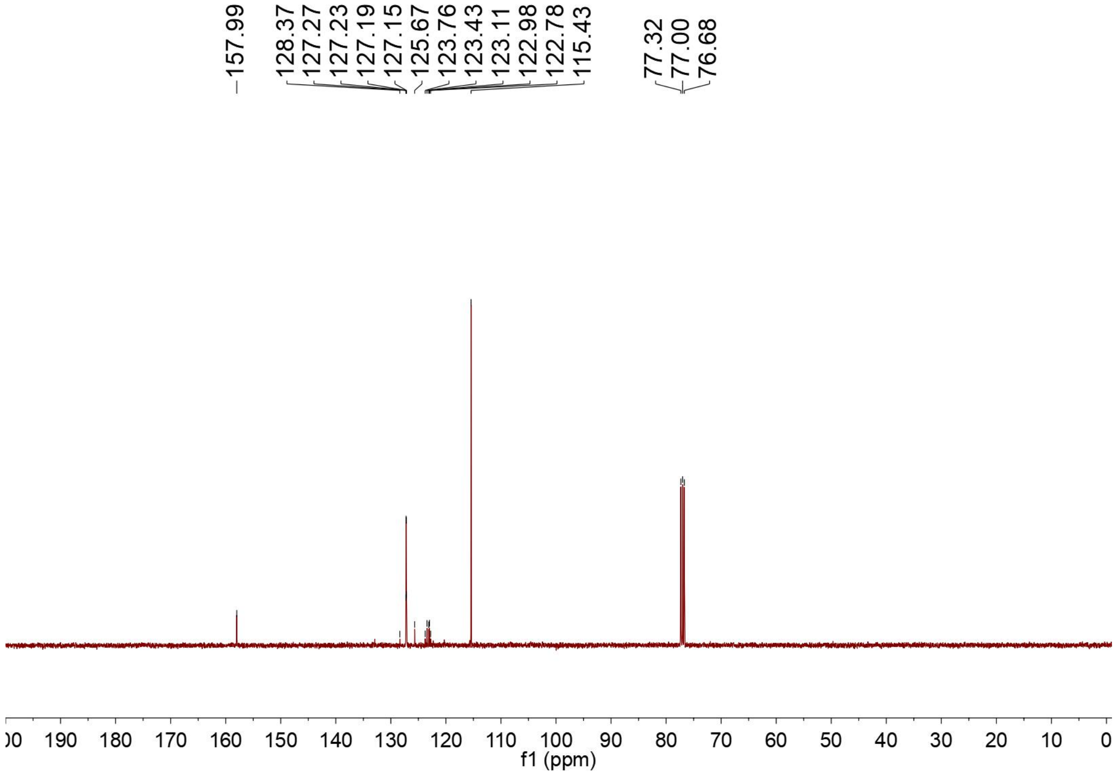
157.99
128.37
127.27
127.23
127.19
127.15
125.67
123.76
123.43
123.11
122.98
122.78
115.43
77.32
77.00
76.68
190 180 170 160 150 140 130 120 110 100 90 80 70 60 50 40 30 20 10 0
f1 (ppm)

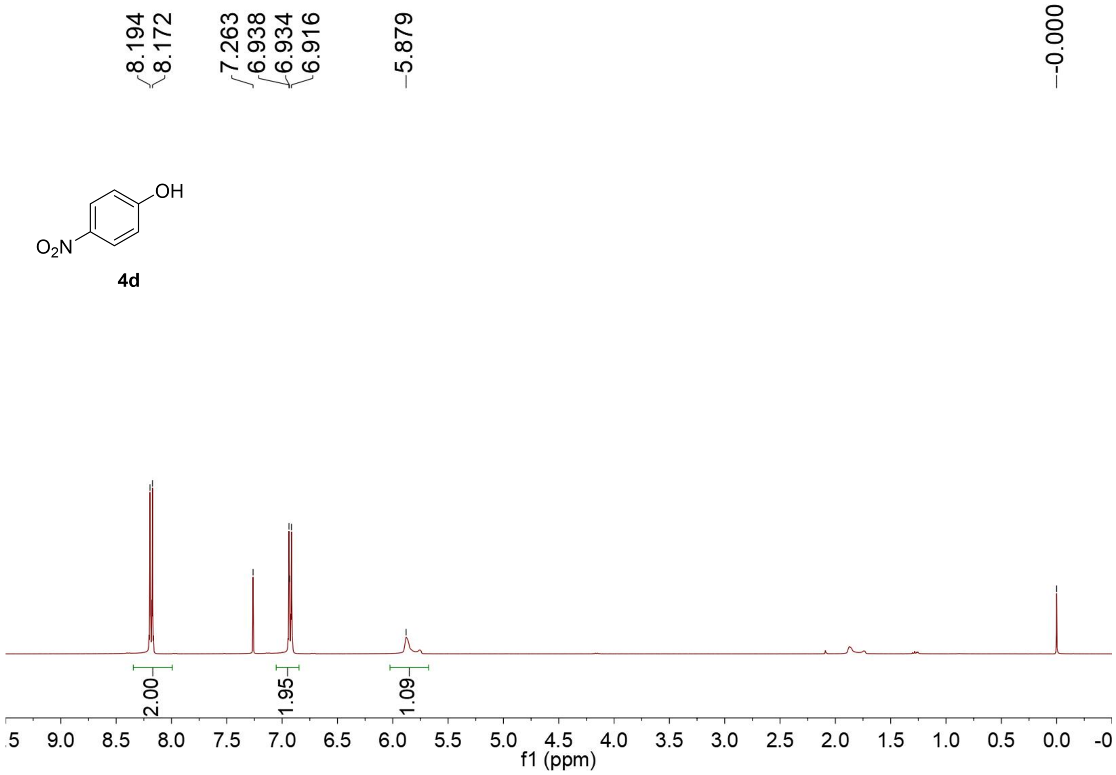
OH
O2N
4d
8.194
8.172
7.263
6.938
6.934
6.916
5.879
0.000
2.00
1.95
1.09
f1 (ppm)

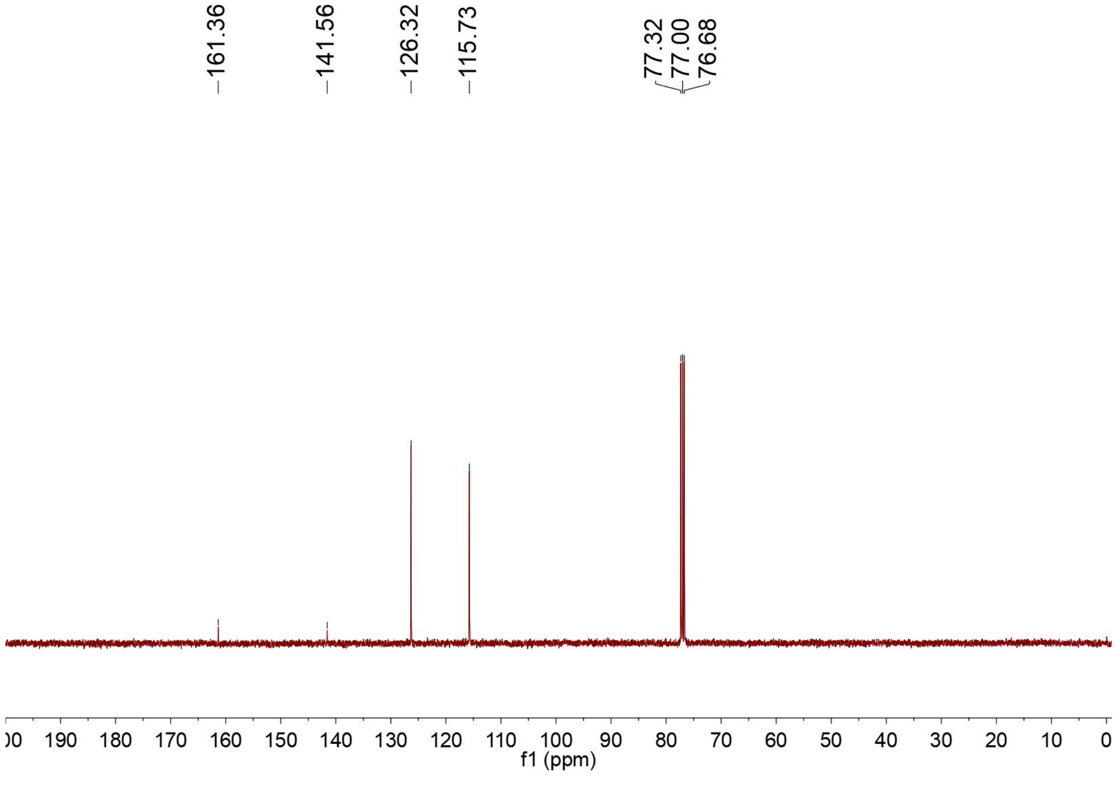
161.36
141.56
126.32
115.73
77.32
77.00
76.68
f1 (ppm)

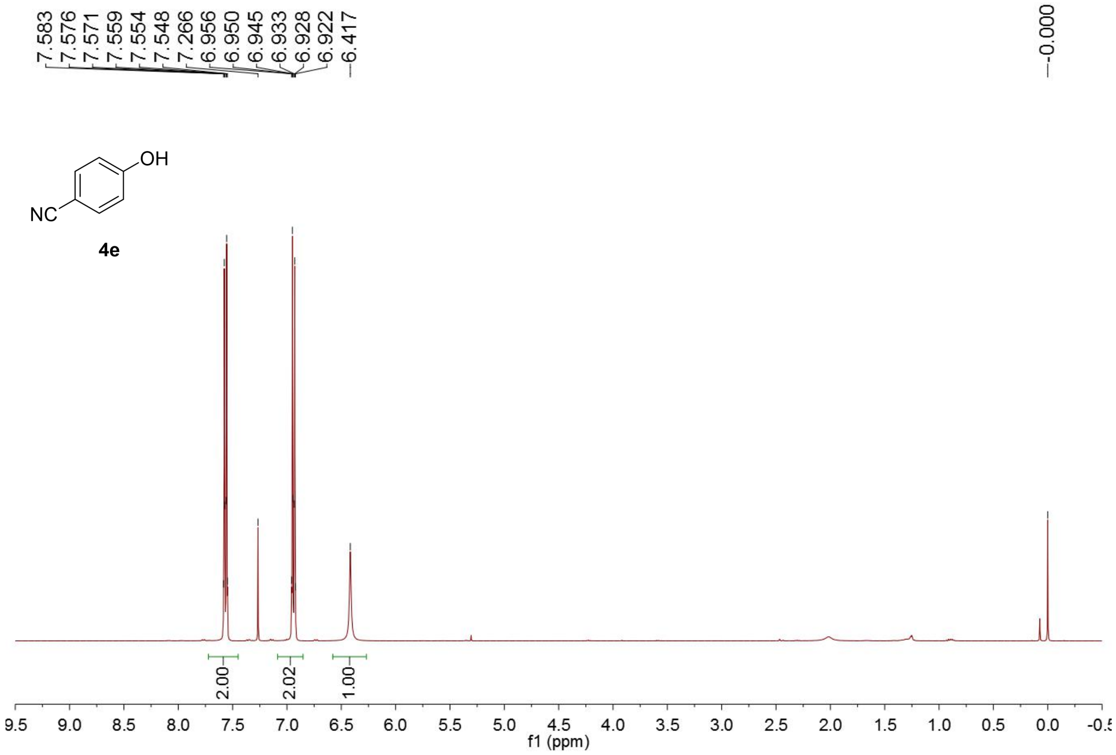
7.583
7.576
7.571
7.559
7.554
7.548
7.266
6.956
6.950
6.945
6.933
6.928
6.922
6.417
-0.000
OH
NC
4e
2.00
2.02
1.00
9.5 9.0 8.5 8.0 7.5 7.0 6.5 6.0 5.5 5.0 4.5 4.0 3.5 3.0 2.5 2.0 1.5 1.0 0.5 0.0 -0.[illegible]
f1 (ppm)

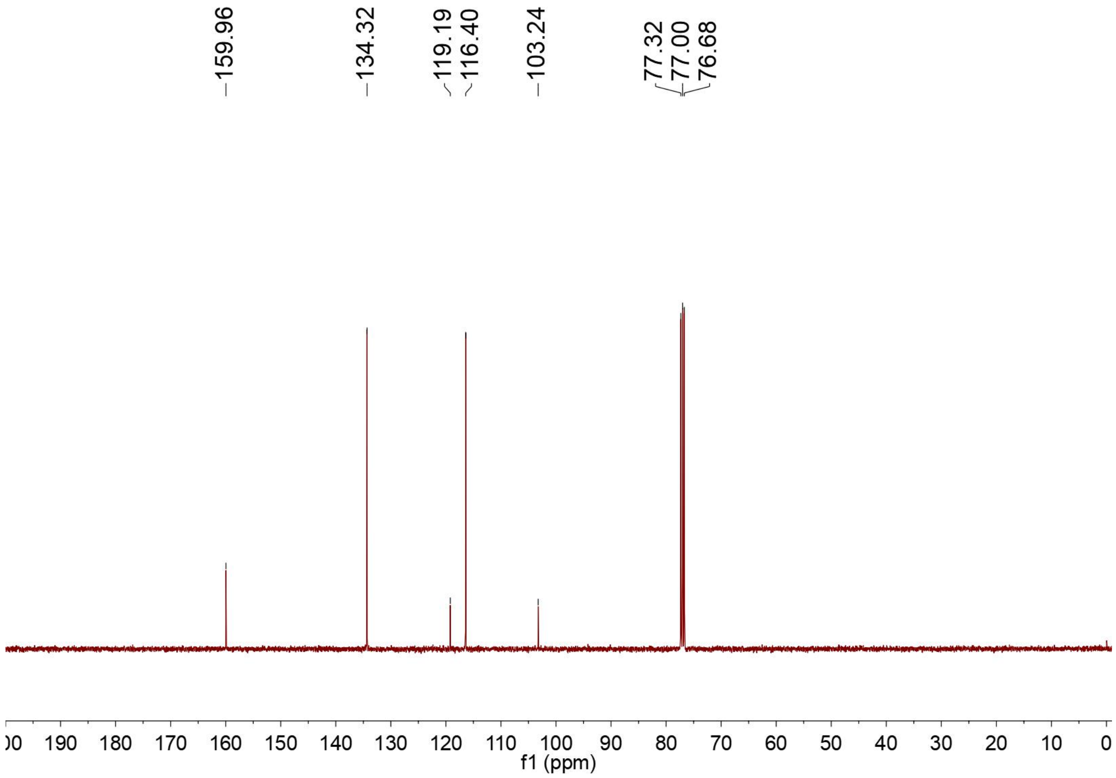
159.96
134.32
119.19
116.40
103.24
77.32
77.00
76.68
190 180 170 160 150 140 130 120 110 100 90 80 70 60 50 40 30 20 10 0
f1 (ppm)

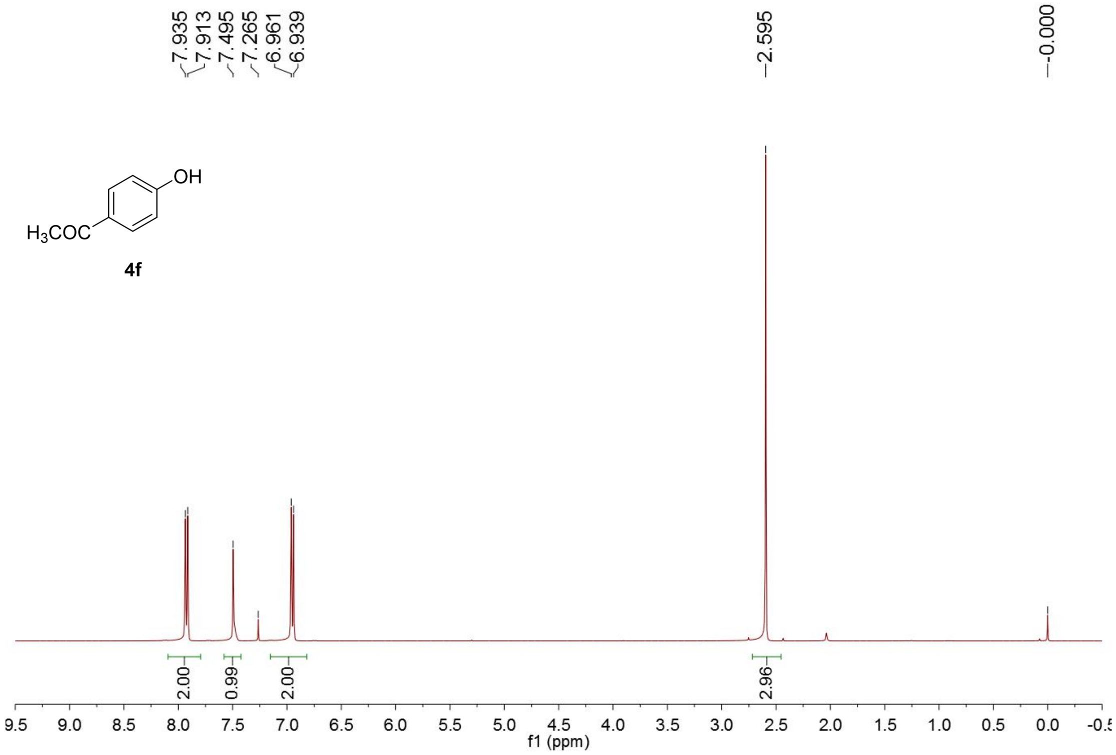
7.935
7.913
7.495
7.265
6.961
6.939
2.595
-0.000
OH
H_3COC
4f
2.00
0.99
2.00
2.96
f1 (ppm)

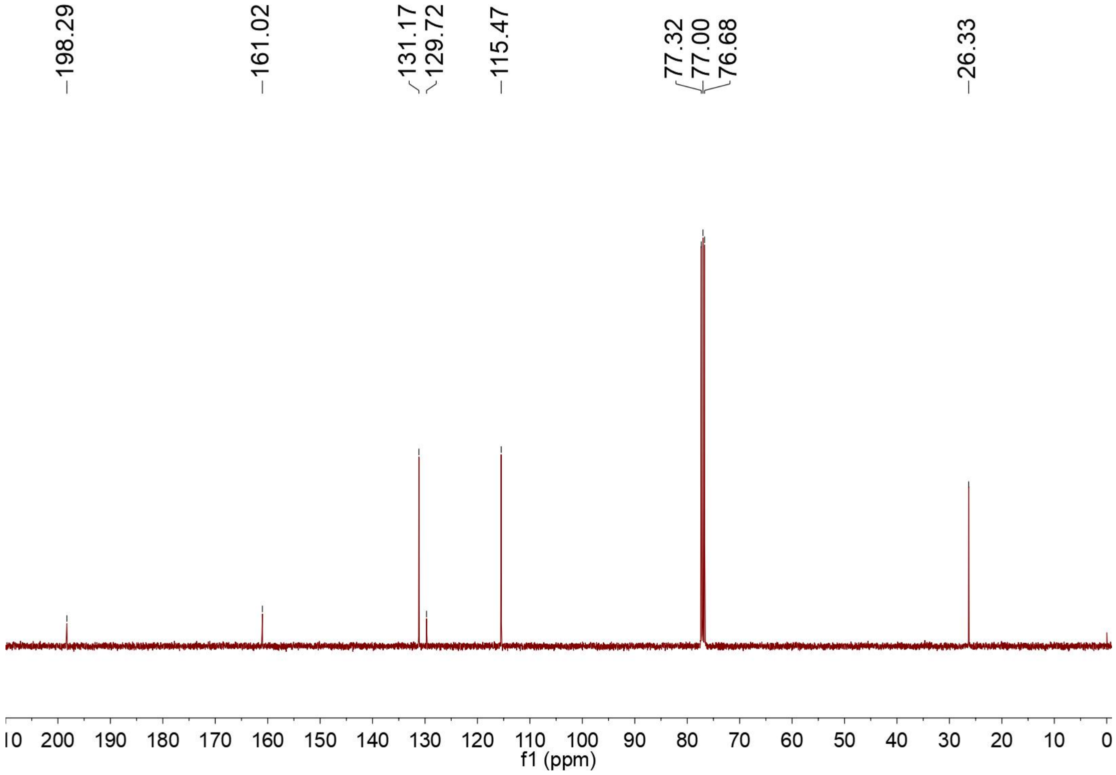
198.29
161.02
131.17
129.72
115.47
77.32
77.00
76.68
26.33
f1 (ppm)

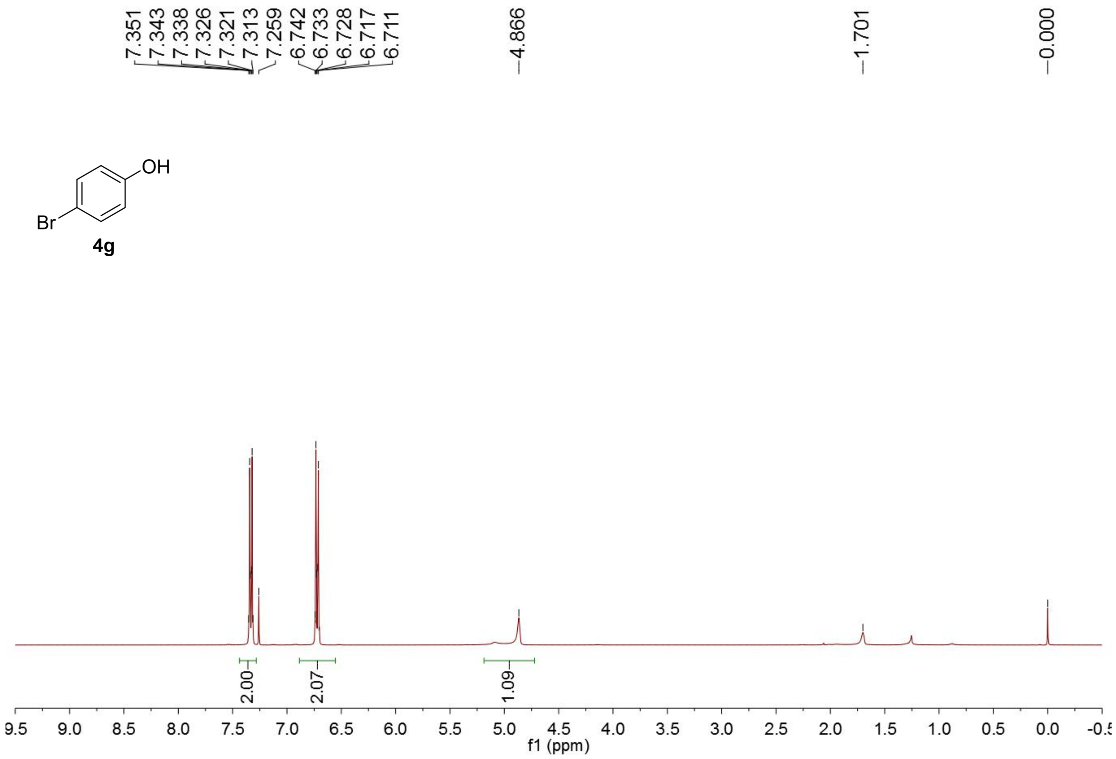
7.351
7.343
7.338
7.326
7.321
7.313
7.259
6.742
6.733
6.728
6.717
6.711
4.866
1.701
0.000
OH
Br
4g
2.00
2.07
1.09
f1 (ppm)

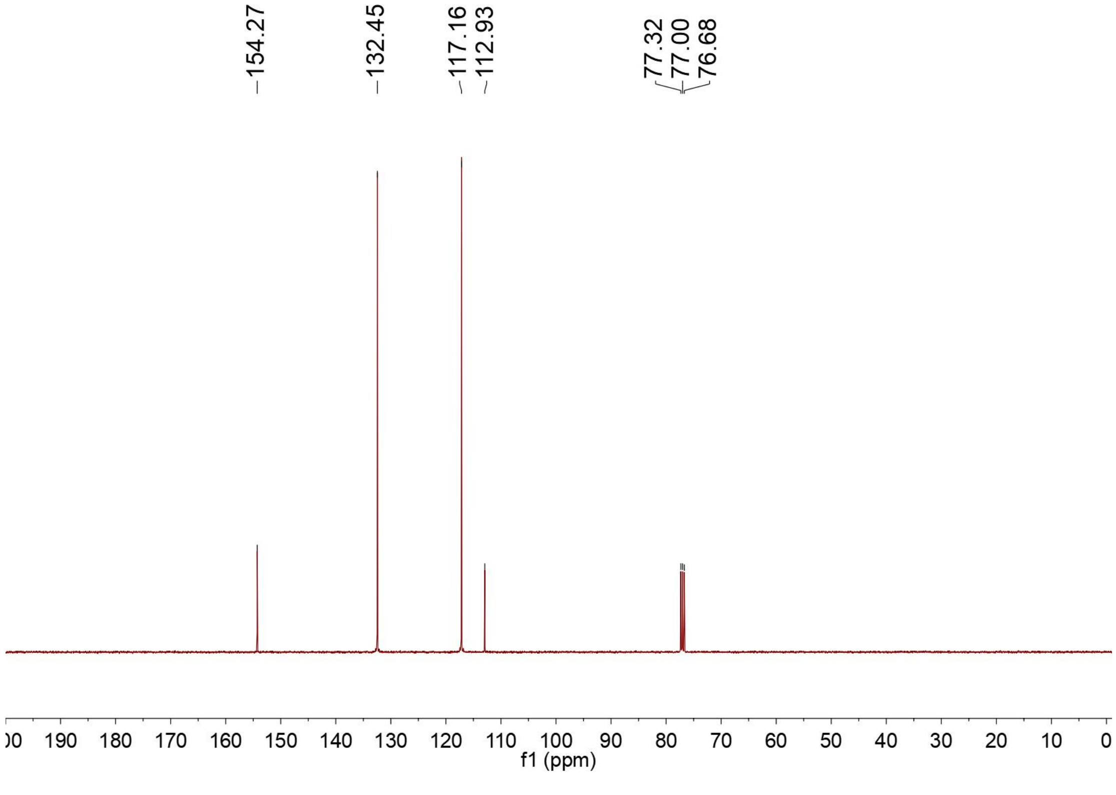
154.27
132.45
117.16
112.93
77.32
77.00
76.68
f1 (ppm)

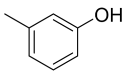

4h

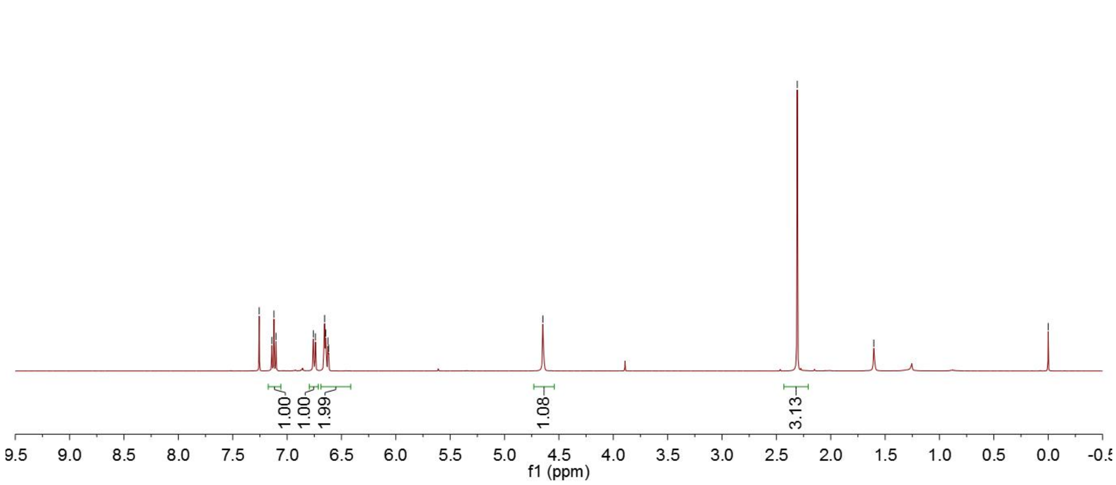
7.256
7.140
7.121
7.102
6.758
6.740
6.655
6.644
6.624
6.618
4.648
2.308
1.604
0.000
1.00
1.00
1.99
1.08
3.13
9.5 9.0 8.5 8.0 7.5 7.0 6.5 6.0 5.5 5.0 4.5 4.0 3.5 3.0 2.5 2.0 1.5 1.0 0.5 0.0
f1 (ppm)

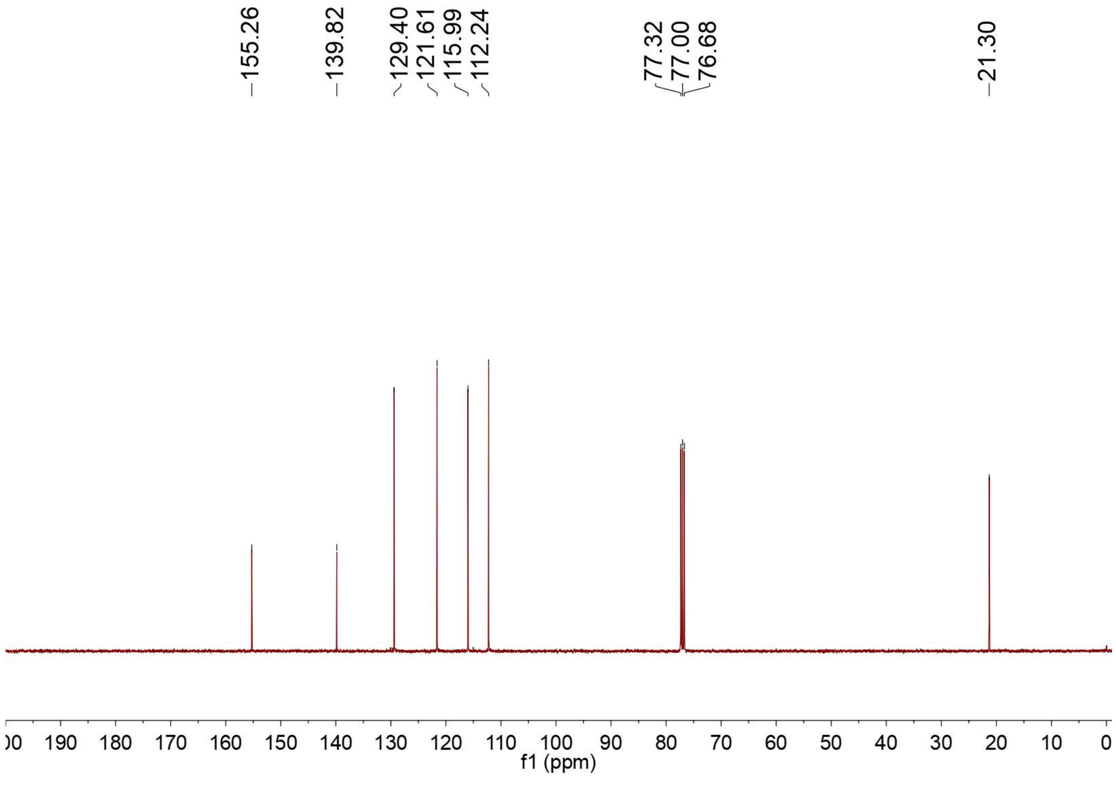
155.26
139.82
129.40
121.61
115.99
112.24
77.32
77.00
76.68
21.30
190 180 170 160 150 140 130 120 110 100 90 80 70 60 50 40 30 20 10 0
f1 (ppm)

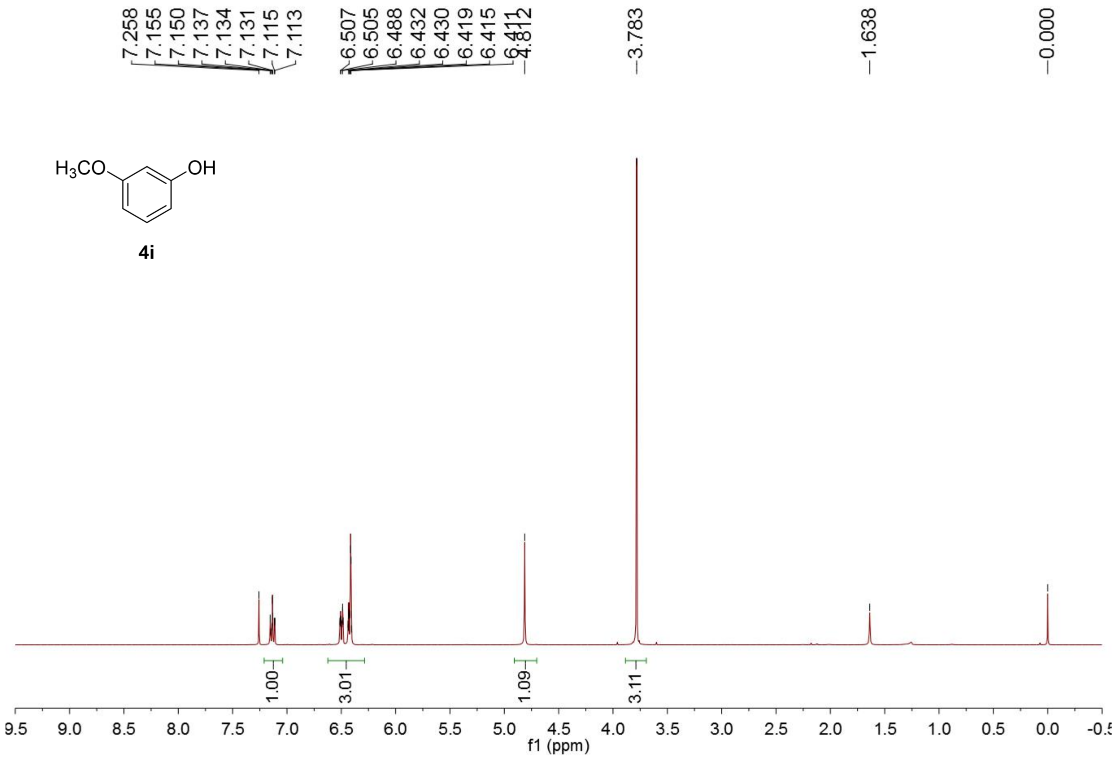
7.258
7.155
7.150
7.137
7.134
7.131
7.115
7.113
6.507
6.505
6.488
6.432
6.430
6.419
6.415
6.411
4.812
3.783
1.638
0.000
H_3CO
OH
4i
1.00
3.01
1.09
3.11
9.5 9.0 8.5 8.0 7.5 7.0 6.5 6.0 5.5 5.0 4.5 4.0 3.5 3.0 2.5 2.0 1.5 1.0 0.5 0.0
f1 (ppm)

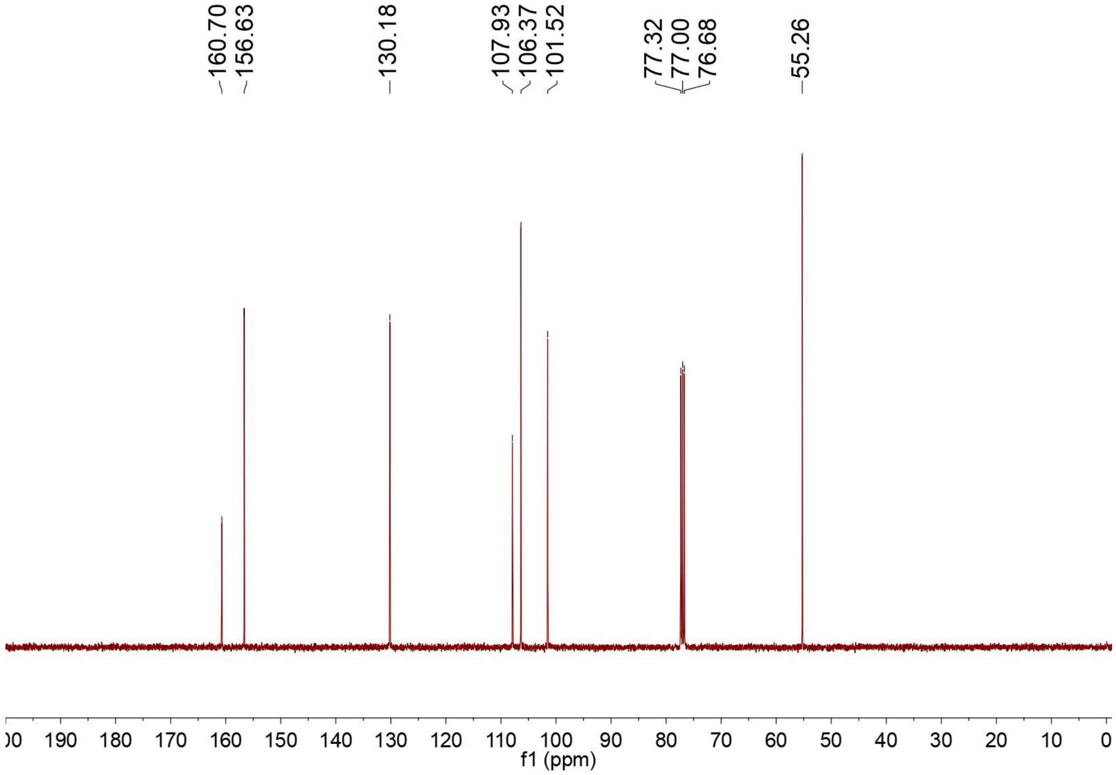
160.70
156.63
130.18
107.93
106.37
101.52
77.32
77.00
76.68
55.26
190 180 170 160 150 140 130 120 110 100 90 80 70 60 50 40 30 20 10 0
f1 (ppm)

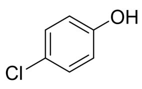

4j

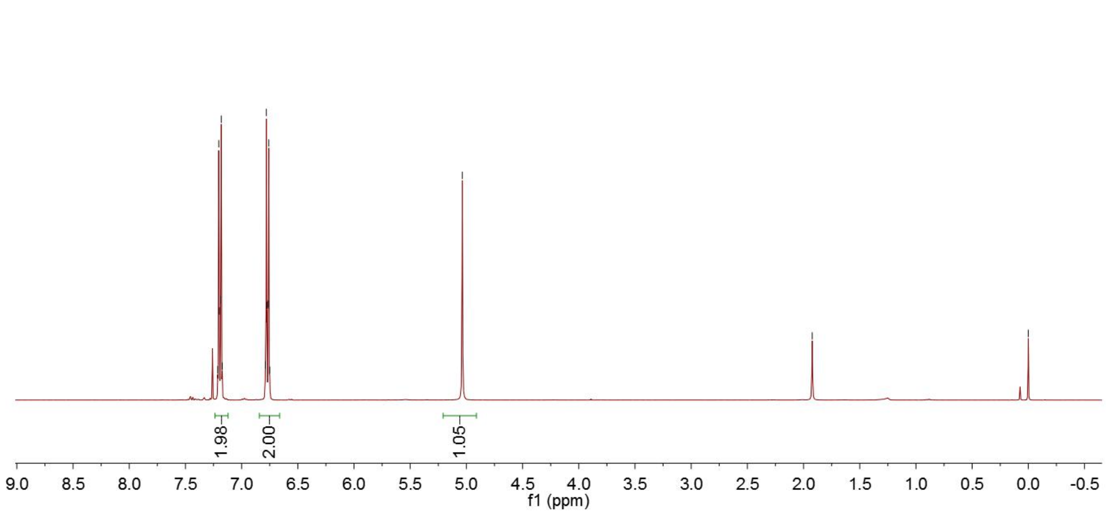
7.212
7.203
7.198
7.187
7.181
7.173
6.789
6.780
6.775
6.764
6.758
6.750
5.036
1.923
-0.000
1.98
2.00
1.05
9.0 8.5 8.0 7.5 7.0 6.5 6.0 5.5 5.0 4.5 4.0 3.5 3.0 2.5 2.0 1.5 1.0 0.5 0.0 -0.5
f1 (ppm)

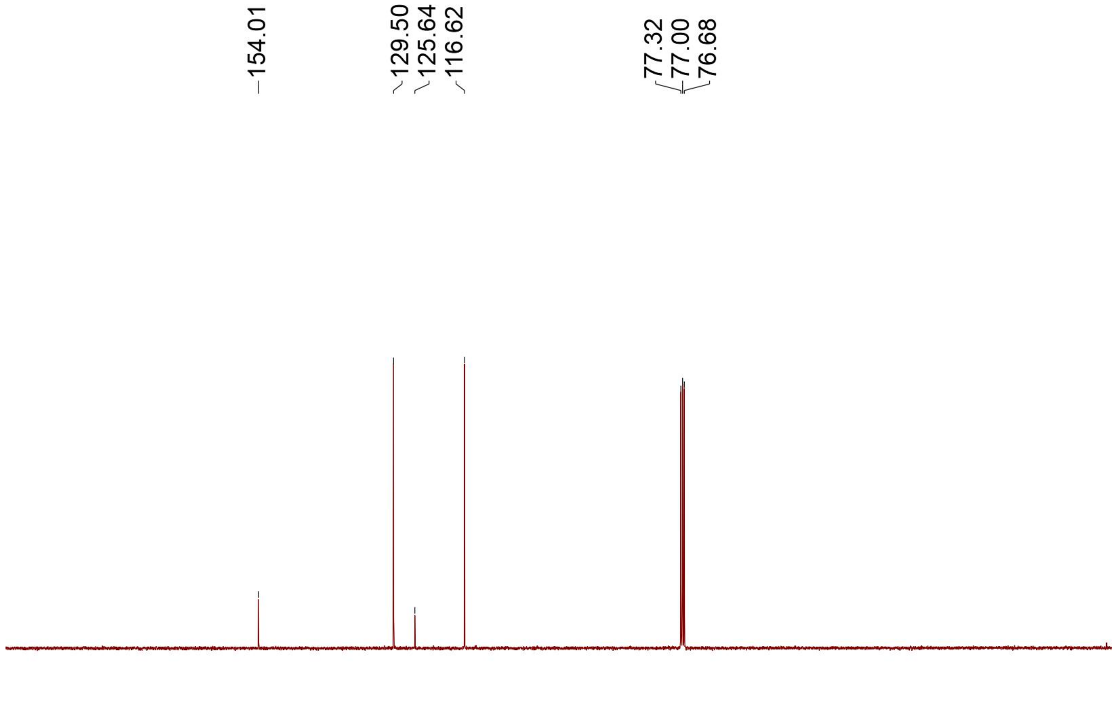
154.01
129.50
125.64
116.62
77.32
77.00
76.68
190 180 170 160 150 140 130 120 110 100 90 80 70 60 50 40 30 20 10 0
f1 (ppm)

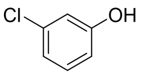

Cl
OH

4k

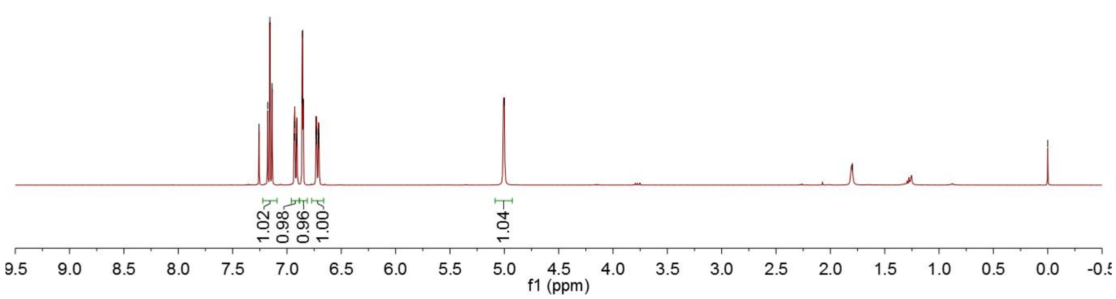

7.257
7.177
7.157
7.137
6.931
6.928
6.911
6.909
6.862
6.856
6.851
6.733
6.731
6.727
6.725
6.713
6.710
6.707
6.704
5.000
1.798
0.000
1.02
0.98
0.96
1.00
1.04
9.5 9.0 8.5 8.0 7.5 7.0 6.5 6.0 5.5 5.0 4.5 4.0 3.5 3.0 2.5 2.0 1.5 1.0 0.5 0.0 -0.5
f1 (ppm)

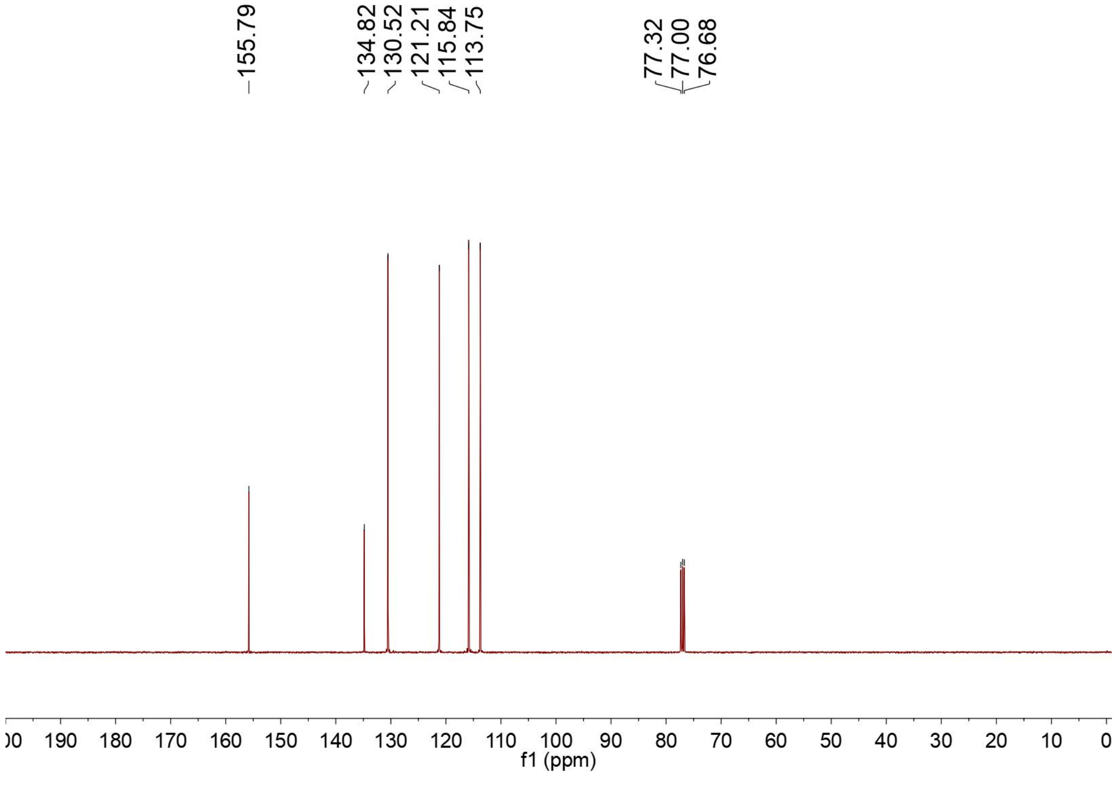

155.79
134.82
130.52
121.21
115.84
113.75
77.32
77.00
76.68
190 180 170 160 150 140 130 120 110 100 90 80 70 60 50 40 30 20 10 0
f1 (ppm)

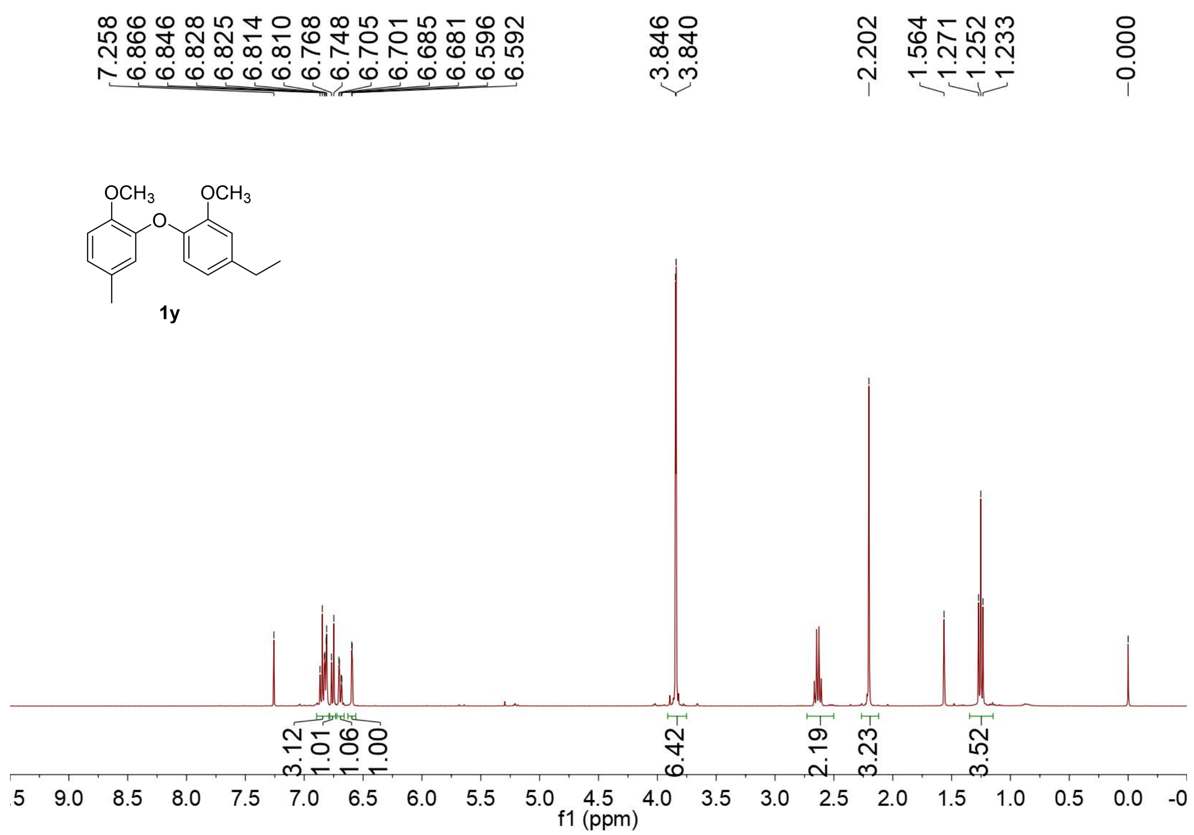
7.258
6.866
6.846
6.828
6.825
6.814
6.810
6.768
6.748
6.705
6.701
6.685
6.681
6.596
6.592
3.846
3.840
2.202
1.564
1.271
1.252
1.233
0.000
OCH3
OCH3
O
1y
3.12
1.01
1.06
1.00
6.42
2.19
3.23
3.52
f1 (ppm)

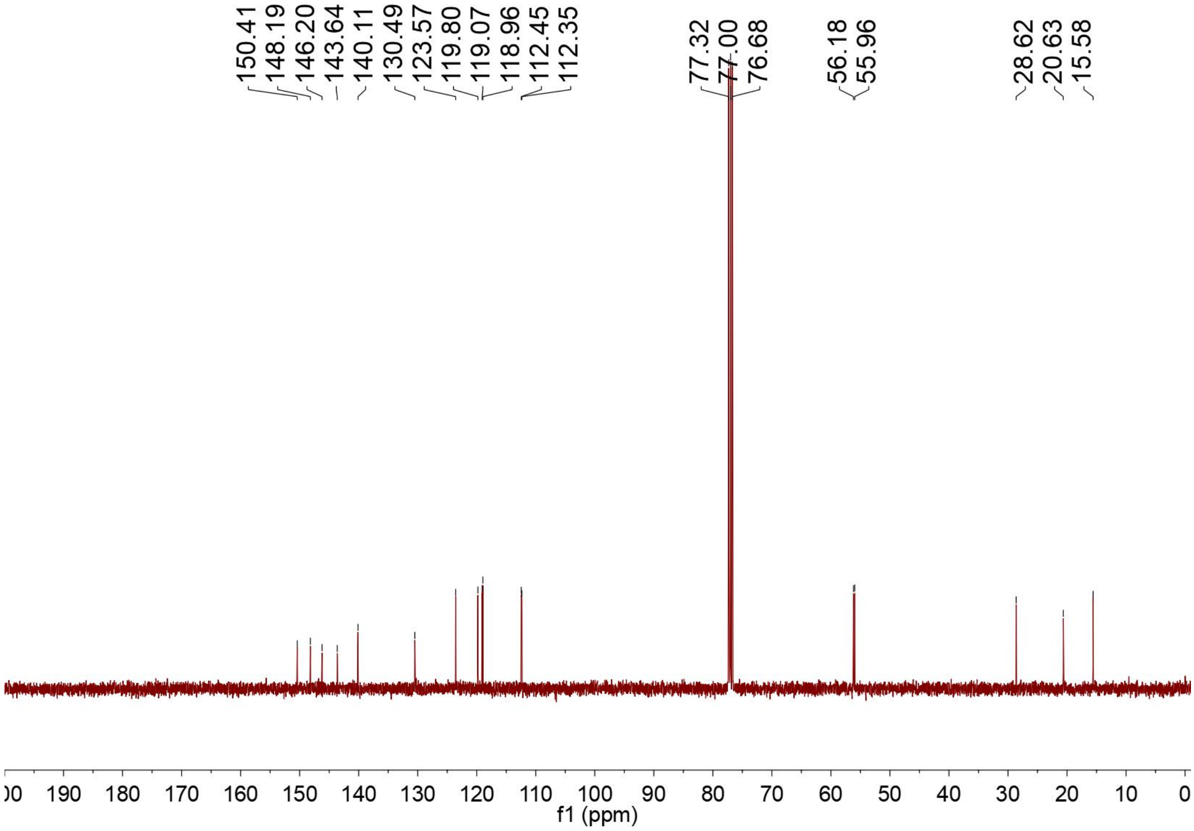
150.41
148.19
146.20
143.64
140.11
130.49
123.57
119.80
119.07
118.96
112.45
112.35
77.32
77.00
76.68
56.18
55.96
28.62
20.63
15.58
f1 (ppm)

Supplementary References

1. Romero, N. A., Margrey, K. A., Tay, N. E. & Nicewicz, D. A. Site-selective arene C-H amination via photoredox catalysis. *Science* **349**, 1326-1330 (2015).
2. Dressler, J. J., Miller, S. A., Meeuwsen, B. T., Riel, A. M. S. & Dahl, B. J. Synthesis of dilactone bridged terphenyls with crankshaft architectures. *Tetrahedron* **71**, 283-292 (2015).
3. Hartwig, J. F., Kawatsura, M., Hauck, S. I., Shaughnessy, K. H. & Alcazar-Roman, L. M. Room-temperature palladium-catalyzed amination of aryl bromides and chlorides and extended scope of aromatic C-N bond formation with a commercial ligand. *J. Org. Chem*. **64**, 5575-5580 (1999).
4. Joshi-Pangu, A. *et al.* Acridinium-Based Photocatalysts: A sustainable option in photoredox catalysis. *J. Org. Chem.* **81**, 7244-7249 (2016).
5. Sergeev, A. G. & Hartwig, J. F. Selective, Nickel-catalyzed hydrogenolysis of aryl ethers. *Science* **332**, 439-443 (2011).
6. Zhai, Y. *et al.* Copper-catalyzed diaryl ether formation from (hetero)aryl halides at low catalytic loadings. *J. Org. Chem.* **82**, 4964-4969 (2017).
7. Hu, T. *et al.* Efficient palladium-catalyzed coupling reactions of aryl bromides and chlorides with phenols. *Chem. Commun*. 7330-7332 (2009).
8. Naidu, A. B., Jaseer, E. A. & Sekar, G. General, mild, and intermolecular Ullmann-type synthesis of diaryl and alkyl aryl ethers catalyzed by diol-Copper(I) complex. *J. Org. Chem*. **74**, 3675-3679 (2009).
9. Cristau, H. J., Cellier, P. P., Hamada, S., Spindler, J. F. & Taillefer, M. A general and mild Ullmann-type synthesis of diaryl ethers. *Org. Lett.* **6**, 913-916 (2004).
10. Kakinuma, Y., Moriyama, K. & Togo, H. Facile preparation of unsymmetrical diaryl ethers from unsymmetrical diaryliodonium tosylates and phenols with high regioselectivity. *Synthesis* **45**, 183-188 (2013).
11. Maity, T. *et al*. A family of ligand and anion dependent structurally diverse Cu(II) Schiff-base complexes and their catalytic efficacy in an O-arylation reaction in ethanolic media. *RSC Adv.* **5**, 82179-82191 (2015).
12. Chen, J. *et al*. Palladium-catalyzed aerobic oxidative coupling of acyl chlorides with arylboronic acids. *Adv. Synth. Catal.* **354**, 2117-2122 (2012).
13. Shih, B.-H., Basha, R. S. & Lee, C. F. Nickel-catalyzed cross-coupling of aryl redoxactive esters with aryl Zinc reagents. *ACS Catal*. **9**, 8862-8866 (2019).
14. Arde, P., Ramanjaneyulu, B. T., Reddy, V., Saxena, A. & Anand, R. V. N-Heterocyclic carbene catalysed aerobic oxidation of aromatic aldehydes to aryl esters using boronic acids. *Org. Biomol. Chem.* **10**, 848-851 (2012).
15. Fujihara, T. *et al.* Palladium-catalyzed esterification of aryl halides using aryl formates without the use of external carbon monoxide. *Chem. Commun.* **48**, 8012-8014 (2012).
16. Gaikwad, V. V. & Bhanage, B. M. Oxime palladacycle in PEG as a highly efficient and recyclable catalytic system for phenoxycarbonylation of aryl iodides with phenols. *Appl. Organometal. Chem*. **33**, e4741 (2019).
17. Chun, S. & Chung, Y. K. Transition-Metal-Free poly(thiazolium) iodide/1,8-diazabicyclo 5.4.0 undec-7-ene/phenazine-catalyzed esterification of aldehydes with alcohols. *Org. Lett.* **19**, 3787-3790 (2017).
18. Zabiulla, Z. *et al.* Synthesis, molecular docking, and apoptogenic efficacy of novel N-heterocycle analogs to target B-cell lymphoma 2/X-linked inhibitors of apoptosis proteins to regress melanoma. *Med. Chem. Res.* **28**, 1132-1160 (2019).
19. Fitzjarrald, V. P. & Pongdee, R. A convenient procedure for the esterification of benzoic acids with phenols: a new application for the Mitsunobu reaction. *Tetrahedron Lett.* **48**, 3553-3557 (2007).
20. Chiriac, C. I. & Tanasa, F. Novel boron reagents for designed organic syntheses. 1. Direct synthesis of aromatic esters in the presence of boric acid. *Des. Monomers Poly.* **6**, 277-281 (2003).
21 Chen, D. *et al.* Formates plus triazabicyclodecene (TBD): an efficient platform for non-gaseous carbonylation and unexpected hydrogenation. *Org. Chem. Front.* **6**, 1403-1408 (2019).
22. Cismesia, M. A. & Yoon, T. P. Characterizing chain processes in visible light photoredox catalysis. *Chem. Sci.* **6**, 5426-5434 (2015).

23. Hatchard, C. G. & Parker, C. A. A new sensitive actinometer. *Proc. Roy. Soc. (London), A235*, 518-536 (1956).

24 Kuhn, H. J., Braslavsky, S. E. & Schmidt, R. Chemical actinometry. *Pure Appl. Chem.* **61**, 187-210 (1989).

25. Fattahi, N., Ayubi, M. & Ramazani, A. Amidation and esterification of carboxylic acids with amines and phenols by N,N'-diisopropylcarbodiimide: A new approach for amide and ester bond formation in water. *Tetrahedron* **74**, 4351-4356 (2018).
